# Exploring the Phytochemical Landscape of the Early-Diverging Flowering Plant *Amborella trichopoda* Baill.

**DOI:** 10.3390/molecules24213814

**Published:** 2019-10-23

**Authors:** Sheng Wu, Alexander E. Wilson, Lijing Chang, Li Tian

**Affiliations:** 1 Shanghai Key Laboratory of Plant Functional Genomics and Resources, Shanghai Chenshan Botanical Garden, Shanghai 201602, China; wusheng@sioc.ac.cn (S.W.); changlijing@csnbgsh.cn (L.C.); 2 Shanghai Chenshan Plant Science Research Center, Chinese Academy of Sciences, Shanghai 201602, China; 3 Department of Plant Sciences, University of California, Davis, Davis, CA 95616, USA; aewilson@ucdavis.edu; 4 Department of Chemistry, Northern Michigan University, Marquette, MI 49855, USA

**Keywords:** *Amborella trichopoda*, metabolome, specialized metabolites, *O*-methyltransferase, OMT, UDP-dependent glycosyltransferase, UGT

## Abstract

Although the evolutionary significance of the early-diverging flowering plant *Amborella* (*Amborella trichopoda* Baill.) is widely recognized, its metabolic landscape, particularly specialized metabolites, is currently underexplored. In this work, we analyzed the metabolomes of *Amborella* tissues using liquid chromatography high-resolution electrospray ionization mass spectrometry (LC-HR-ESI-MS). By matching the mass spectra of *Amborella* metabolites with those of authentic phytochemical standards in the publicly accessible libraries, 63, 39, and 21 compounds were tentatively identified in leaves, stems, and roots, respectively. Free amino acids, organic acids, simple sugars, cofactors, as well as abundant glycosylated and/or methylated phenolic specialized metabolites were observed in *Amborella* leaves. Diverse metabolites were also detected in stems and roots, including those that were not identified in leaves. To understand the biosynthesis of specialized metabolites with glycosyl and methyl modifications, families of small molecule UDP-dependent glycosyltransferases (UGTs) and *O*-methyltransferases (OMTs) were identified in the *Amborella* genome and the InterPro database based on conserved functional domains. Of the 17 phylogenetic groups of plant UGTs (A–Q) defined to date, *Amborella* UGTs are absent from groups B, N, and P, but they are highly abundant in group L. Among the 25 *Amborella* OMTs, 7 cluster with caffeoyl-coenzyme A (CCoA) OMTs involved in lignin and phenolic metabolism, whereas 18 form a clade with plant OMTs that methylate hydroxycinnamic acids, flavonoids, or alkaloids. Overall, this first report of metabolomes and candidate metabolic genes in *Amborella* provides a starting point to a better understanding of specialized metabolites and biosynthetic enzymes in this basal lineage of flowering plants.

## 1. Introduction

*Amborella* (*Amborella trichopoda* Baill.), a short shrub native to the tropical rainforests of New Caledonia, is the only living species in the Amborellales, the earliest diverging order of flowering plants (angiosperms) [1]. Despite its widely recognized importance in understanding flowering plant phylogeny and land plant evolution, little is known about the metabolomes, in particular specialized metabolites (secondary metabolites), of *Amborella*. To date, only three specialized metabolites, procyanidin, kaempferol-3-*O*-glucoside, and kaempferol-3-*O*-rutinoside, have been tentatively identified from *Amborella* leaves according to the R*_f_* values of the compounds measured using paper and column chromatography [2].

Plant specialized metabolites participate in beneficial and defensive interactions between plants and the environment [3,4,5]. In addition, their bioactivities in humans have been exploited as a source of nutraceuticals (e.g., phytonutrients) and pharmaceuticals (e.g., anticancer agents) [6]. Plant specialized metabolites can be classified into three major groups: terpenoids (isoprenoids), phenolics, and alkaloids. Terpenoids are divided into mono- (C_10_), sesqui- (C_15_), di- (C_20_), sester- (C_25_), tri- (C_30_), tetra- (C_40_), and poly- (C_n_) terpenoids based on the number of C_5_ isoprene units in the carbon skeleton [7]. A majority of plant phenolics are produced initially by the general phenylpropanoid pathway and subsequently branch into groups of flavonoids, isoflavonoids, anthocyanins, and proanthocyanidins [8]. Unlike the hydrophobic nature of most terpenoid and phenolic aglycones, alkaloids are water-soluble, alkaline molecules that contain heterocyclic (true alkaloids) or exocyclic (proto-alkaloids) nitrogen atoms. Alkaloids can be categorized based on either the biosynthetic precursors (e.g., phenylalanine, tyrosine, lysine, arginine, etc.) or the N-containing heterocyclic/exocyclic ring structures (e.g., monoterpenoid indole alkaloids, benzylisoquinoline alkaloids, tropane alkaloids, pyrrolizidine alkaloids, etc.) [9].

There are estimated over 200,000 specialized metabolites produced by plants [10]. The rich diversity of plant specialized metabolites is conferred by enzymes that modify the core compound structures (i.e., modification enzymes). Glycosylation and *O*-methylation, catalyzed by UDP-dependent glycosyltransferases (UGTs) and *O*-methyltransferases (OMTs), respectively, represent common modifications of plant specialized metabolites [11]. Conserved functional domains have been identified in plant UGT and OMT proteins. Plant small molecule UGTs contain a 44 amino acid plant secondary product glycosyltransferase (PSPG) motif for interacting with the sugar donor [12]. *S*-adenosyl-l-methionine (SAM)-dependent OMTs contain a GxG/GxGxG motif for binding the adenosyl part of SAM and an acid reside for hydrogen bonding with the ribose part of SAM. Plant small-molecule OMTs can be divided into three groups. Class I OMTs require divalent ions (e.g., Mg^2+^) for activity and include caffeoyl-coenzyme A OMTs (CCoA OMTs). Class II OMTs do not require divalent ions for catalysis and encompass OMTs that methylate substrates with diverse structures. A third group, the SABATH (salicylic acid carboxyl methyltransferase, benzoic acid carboxyl methyltransferase, and theobromine synthase) OMTs, catalyzes the formation of volatile esters and does not share significant sequence homology with the classes I and II OMTs [13].

To better understand the evolutionary roles of phytochemicals in plant–environment interactions, we performed metabolite profiling in an early diverging lineage of flowering plants *Amborella*. The metabolomes of *Amborella* leaves, stems, and roots were analyzed using liquid chromatography high-resolution electrospray ionization mass spectrometry (LC-HR-ESI-MS), which revealed, for the first time, the accumulation of diverse primary and specialized metabolites in these tissues. In addition, the fully sequenced *Amborella* genome allowed us to explore candidate biosynthetic enzymes that give rise to these structurally diverse phytochemicals, particularly those with glycosyl and methyl modifications. Families of small molecule UGTs and OMTs were identified in *Amborella,* and their phylogenetic association with other plant UGTs and OMTs were examined. These metabolic and molecular data from *Amborella* provide a reference point for interrogating the evolution, function, and environmental interactions of phytochemicals and their biosynthetic genes.

## 2. Results

### 2.1. Abundant Phenolic Compounds with Glycosylation and Methylation Modifications in Amborella Leaves

**Figure 1:**
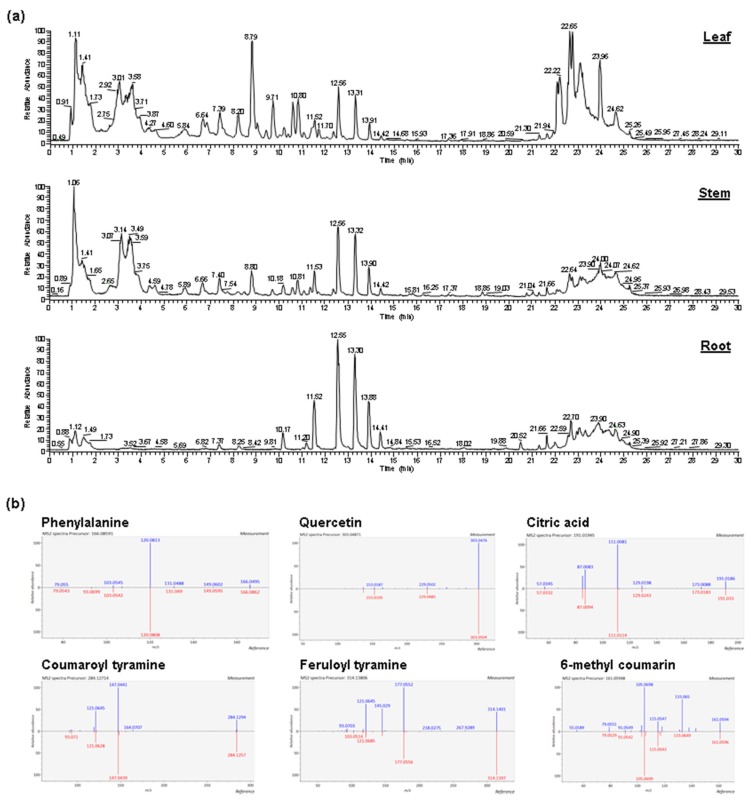
(**a**) Total ion chromatograms (TICs) of metabolites extracted from the leaf, stem, and root tissues of *Amborella trichopoda*. (**b**) Representative MS/MS spectral comparisons of the *A. trichopoda* metabolites (measurement) with the corresponding phytochemical standards in the publicly available mass spectral libraries (reference).

To examine the metabolomes of Amborella, methanolic extracts of leaf, stem, and root tissues were analyzed using LC-HR-ESI-MS (Figure 1). The Amborella metabolites were tentatively identified by matching their MS and MS/MS spectra with those of authentic phytochemical standards in the publicly available mass spectral libraries, followed by manual inspection. After data processing, 63, 39, and 21 compounds were tentatively identified in leaves, stems, and roots of *Amborella*, respectively, with peak areas ranging from 5.4 × 10^6^ to 2.7 × 10^10^ (Tables S1–S3).

In *Amborella* leaves, the detectable free amino acids include isoleucine, arginine, aspartic acid, glutamine, and glutamic acid as well as the three aromatic amino acids, phenylalanine, tyrosine, and tryptophan, and their acetylated derivatives acetylphenylalanine and acetyltryptophan (Table S1). A group of organic acids including gluconate, citrate, malic acid, citramalate, 2-isopropylmalic acid, glutaric acid, 2-hydroxyisocaproic acid, and azelaic acid was found. Several cofactors, such as oxidized glutathione, flavin adenine dinucleotide, and riboflavin, as well as the nucleobase adenine and its nucleoside derivative adenosine, were also detected. In addition, *Amborella* leaves contain simple sugars (sucrose and raffinose), nucleotide sugars (UDP-glucose and UDP-xylose), and phosphate sugars (mannose 6-phosphate) (Table S1).

**Figure 2:**
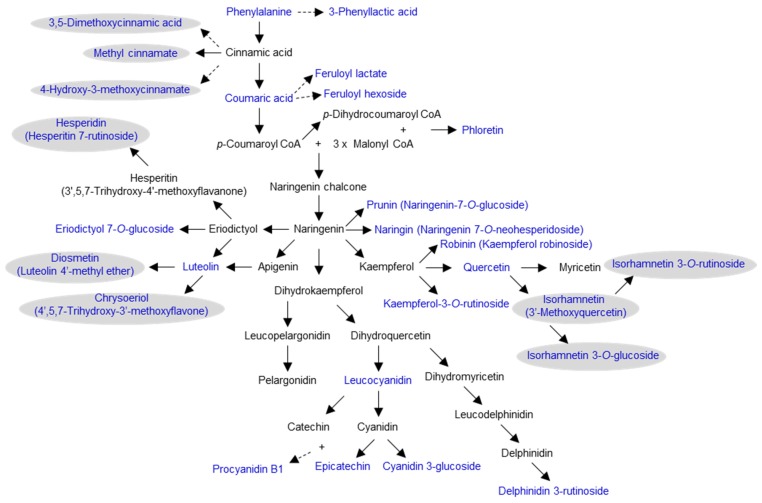
A simplified scheme of the general phenylpropanoid, flavonoid, anthocyanin, and proanthocyanidin biosynthetic pathways in *Amborella trichopoda*. These aglycones lead to the formation of diverse glycosidic derivatives (Tables S1–S3). Metabolites present in *Amborella* leaves are shown in blue, and methylated compounds are shaded in gray. Dotted arrows denote multiple enzymatic steps.

Phenylalanine serves as the biosynthetic precursor of phenylpropanoids. In addition to phenylalanine (in the free amino acid form), metabolites in the general phenylpropanoid pathway and their derivatives were found in high abundance in *Amborella* leaves, including coumaric acid, methyl cinnamate, 4-hydroxy-3-methoxycinnamate, 3,5-dimethoxycinnamic acid, 3-phenyllactic acid, feruloyl lactate, and feruloyl hexoside (Figure 2; Table S1). Products of the general phenylpropanoid pathway led to the biosynthesis of several groups of phenolic compounds detected in *Amborella* leaves, including coumarins (6-methylcoumarin, 6,8-dimethyl-4-hydroxycoumarin), isoflavonoids (genistein), anthocyanins (leucocyanidin, cyanidin 3-glucoside, delphinidin 3-rutinoside), proanthocyanidins (procyanidin B1), and flavonoids (Table S1).

A wide range of flavonoid aglycones and glycosides are present in *Amborella* leaves (Figure 2; Table S1). In particular, there are 6 flavonol aglycones and glycosides, robinin (kaempferol robinoside), kaempferol-3-*O*-rutinoside, quercetin, isorhamnetin (3′-methoxyquercetin), isorhamnetin 3-*O*-glucoside, and isorhamnetin 3-*O*-rutinoside. In addition, chalcones (phloretin (dihydronaringenin)), glycosides of flavanones (prunin (naringenin-7-*O*-glucoside), naringin (naringenin 7-*O*-neohesperidoside), eriodictyol-7-*O*-glucoside, hesperidin (hesperitin-7-rutinoside)), flavones and flavone glucosides (luteolin, diosmetin (luteolin 4′-methyl ether), chrysoeriol (4′,5,7-trihydroxy-3′-methoxyflavone)), and flavan-3-ols (epicatechin) were also found (Table S1). Along with glycosylation, methylation is another modification commonly observed for many phenolic compounds in *Amborella* leaves (Figure 2; Table S1).

### 2.2. Diverse Metabolites in Amborella Stems and Roots

*Amborella* stems contain the free amino acids lysine, 5-oxo-proline, and 2-aminoadipic acid, in addition to arginine, aspartic acid, glutamic acid, glutamine, tyrosine, phenylalanine, and acetyltryptophan that are also identified in leaves (Table S2). Three organic acids, malic acid, saccharic acid, and 2-hydroxyisocaproic acid, are present in stems. Besides sucrose, raffinose, mannose 6-phosphate, and UDP-glucose that are detectable in both leaves and stems, stems also accumulate trehalose (Table S2).

Like leaves, phenolic compounds are the most abundant specialized metabolites in stems (Table S2). These include coumaric acid, feruloyl hexoside, 6-methylcoumarin, kaempferol 3-*O*-glucoside, kaempferol 3-*O*-rutinoside, quercetin 7-*O*-rhamnoside, rutin (quercetin rutinoside), isorhamnetin (3′-methoxyquercetin), isorhamnetin 3-*O*-glucoside, isorhamnetin 3-*O*-rutinoside, naringenin, naringin (naringenin 7-*O*-neohesperidoside), prunin (naringenin-7-*O*-glucoside), and epicatechin. Several phenolics were found in stems but not leaves, such as coumaroyl putrescin, coumaroyl hexoside, sinapyl aldehyde, feruloyl tyramine, and catechin. A cyclohexenone glucoside, roseoside, was also detected in stems (Table S2).

Only a few metabolites were identified in *Amborella* roots, of which arginine, glutamic acid, citric acid, glutaric acid, malic acid, azaleic acid, adenine, UDP-glucose, trehalose, 3-phenyllactic acid, 3,5-dimethoxycinnamic acid, naringenin, and feruloyl tyramine are in common with leaves and/or stems (Tables S1–S3). However, several metabolites were found only in roots, but not leaves and stems, including citraconic acid, galactarate, 4-hydroxy-proline, 2-deoxy-d-glucose, pantothenic acid, coumarol tyramine, 2-propanamidoacetic acid, and heptadecanoic acid (Table S3).

### 2.3. Large Families of UDP-Dependent Glycosyltransferases (UGTs) and O-Methyltransferases (OMTs) in the Amborella Genome

**Figure 3:**
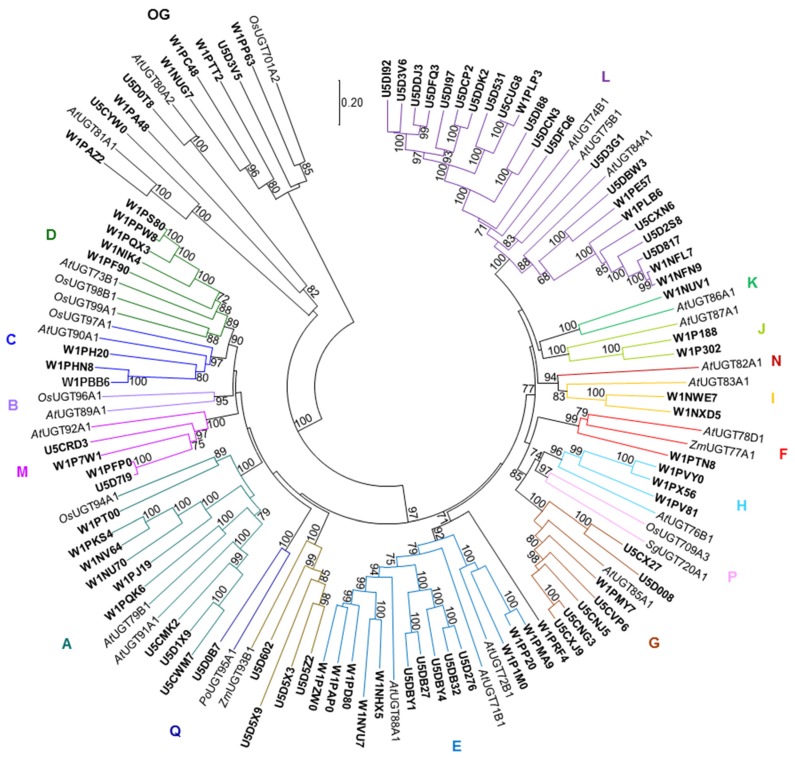
A neighbor-joining tree of *Amborella trichopoda* UGTs and selected UGTs representing different plant UGT phylogenetic groups. The *Amborella* UGTs are highlighted in bold. Bootstrap values greater than 60 are shown next to the branches. OG, outgroup.

To explore the UGTs and OMTs that generate the diverse glycosylated and/or methylated specialized metabolites in *Amborella* tissues, the fully sequenced *Amborella* genome [14] and the InterPro database for protein sequence analysis and classification were searched using functional domains common to plant small molecule UGTs or OMTs. There are 87 putative UGTs in *Amborella* that contain the classic PSPG motif conserved in plant UGTs and are over 340 aa in length (the minimum size of a functionally characterized plant UGT) (Figure S1). To understand the evolutionary relationship among the UGTs, a neighbor-joining tree was built with the *Amborella* UGTs and selected UGTs representing different plant UGT phylogenetic groups (Figure 3). Of the 17 UGT phylogenetic groups delineated in plants to date (A–Q), *Amborella* UGTs occupy 14 groups (A, C–M, O, and Q), but they are absent in groups B, N, and P (Figure 3). While groups C, D, H, I, J, M, and O contain 2 to 5 *Amborella* UGTs per group, only one *Amborella* UGT is present each in groups F, K, and Q. On the other hand, groups G, A, and E are relatively abundant with *Amborella* UGTs, containing 7, 9, and 13 UGTs, respectively. Mostly notably, 25% of the *Amborella* UGTs (22 out of 87) belong to group L (Figure 3). There are also 9 *Amborella* UGTs in the outgroup clade of plant UGTs that glycosylate sterols and lipids [15]. Interestingly, the *Amborella* UGT W1PRF4 is not associated with any of the currently defined plant UGT clades (Figure 3).

**Figure 4:**
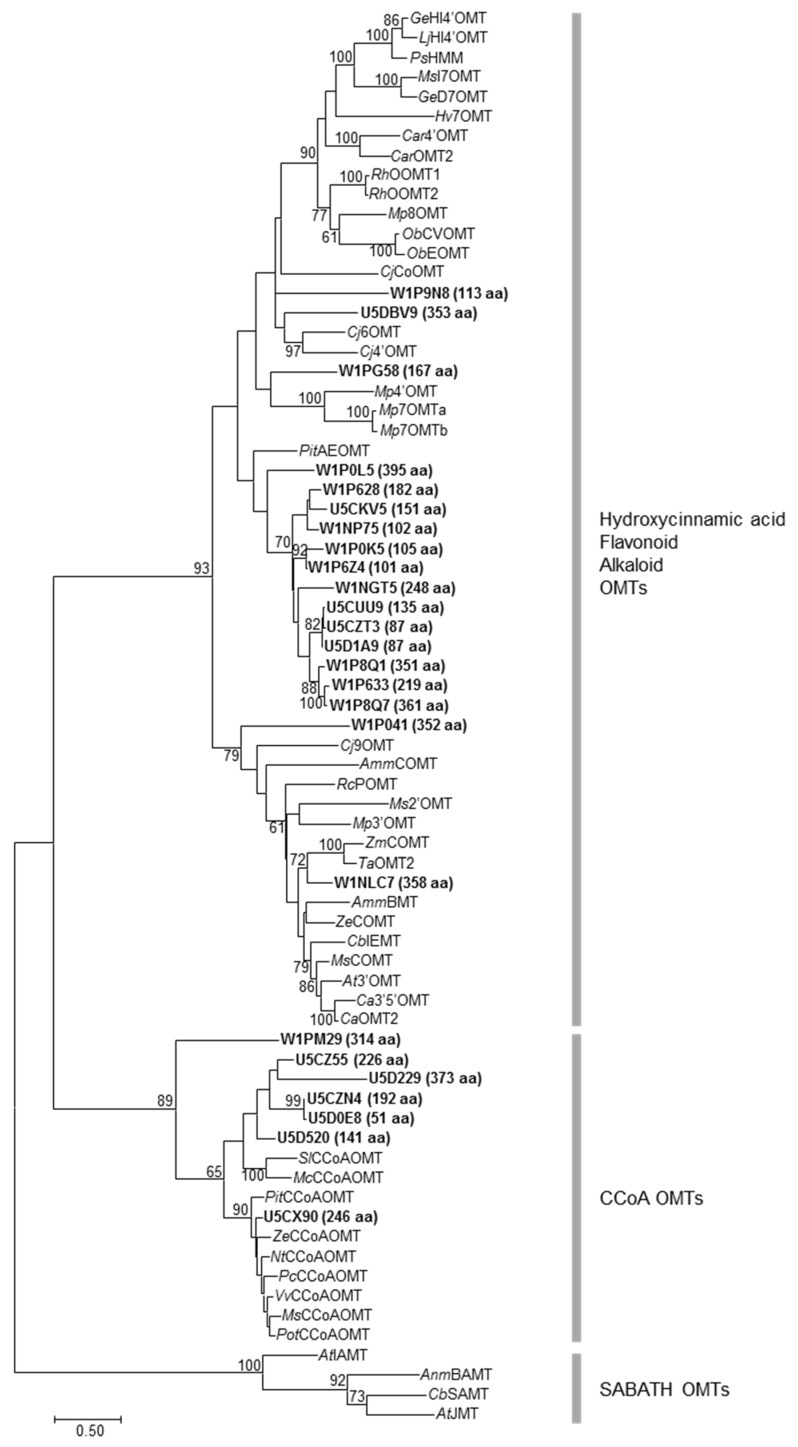
A maximum likelihood tree of *Amborella trichopoda O*-methyltransferases (OMTs) and selected functionally characterized plant small-molecule OMTs. The size of the predicted *Amborella* OMT is shown next to the identifier (highlighted in bold). Bootstrap values greater than 60 are shown next to the branches.

Twenty-five *Amborella* proteins (51 to 395 aa, average size 216 ± 111 aa) were predicted to contain the *O*-methyltransferase, class I-like SAM-dependent *O*-methyltransferase, O-methyltransferase COMT-type, or plant methyltransferase dimerization domain (Figure 4). The protein sequences of *Amborella* OMTs and selected functionally characterized plant small-molecule OMTs were aligned (Figure S2). Because some of the pairwise distances could not be estimated from the multiple sequence alignment, a character-based method, the maximum likelihood method, was used for building the OMT phylogeny instead of neighbor-joining, which requires a distance matrix (Figure 4). Eighteen *Amborella* OMTs (including a tight cluster of 13 OMTs) were associated with plant OMTs functioning towards hydroxycinnamic acids, flavonoids, or alkaloids (Figure 4). Interestingly, within the same clade, W1NLC7 (358 aa) grouped closely (bootstrap value 72) with two monocot OMTs, *Zm*COMT (maize) and *Ta*OMT2 (wheat), which utilize caffeic acid as substrate. Seven *Amborella* OMTs clustered with plant CCoA OMTs, including U5CZ55, U5D229, U5CZN4, U5D0E8, and U5D520 that fall in the same group as *Sl*CCoAOMT and *Mc*CCoAOMT, U5CX90 that is associated with other plant CCoA OMTs, and W1PM29 that is more distant from the other *Amborella* CCoA OMTs (Figure 4). It should be noted that the SABATH OMTs from *Arabidopsis thaliana*, *Antirrhinum majus*, and *Clarkia breweri* were used as the outgroup for the phylogenetic analysis. Thirteen *Amborella* proteins contain the SAM-dependent carboxyl methyltransferase domain (present in SABATH OMTs) but were not included in the phylogenetic analysis.

## 3. Discussion

Overall, this nontargeted, metabolite-profiling study revealed the presence of diverse groups of phytochemicals in *Amborella* tissues. Putative metabolite identification was accomplished by querying the *Amborella* metabolites against authentic standards in multiple mass spectral libraries followed by manual inspection of the matched spectra. The accumulation of phenolic specialized metabolites in *Amborella* tissues suggests a major role of these compounds in *Amborella* interactions with the environment. On the other hand, the low abundance of terpenoids and alkaloids in these tissues could be due to either the lack of biosynthetic genes and enzymes or inducible biosynthesis that only occurs under stress conditions.

An interesting observation was that feruloyl tyramine and coumaroyl tyramine were identified in stems and/or roots, but not leaves (Tables S1–S3). Hydroxycinnamoyl tyramines are reportedly phytoalexins with increased production in response to wounding [16] and inoculation of pathogens [17] in other plant species. This poses the question of whether feruloyl tyramine and coumaroyl tyramine constitute a form of chemical defense against abiotic and biotic stresses in *Amborella* stems and roots. If this is the case, it remains to be answered whether these compounds in *Amborella* have coevolved with pathogens in the environment.

Notably, various glycosylated and methylated phenolics are produced by *Amborella* tissues, particularly leaves (Tables S1–S3). The diverse glycosylated flavonoids, anthocyanins, and proanthocyanidins present in *Amborella* leaves may protect the plants from UV radiation, as have been demonstrated in other plants [18]. Although the role of methylated flavonoids in foliar tissues has not been well characterized, methylated isoflavonoids were shown to act as phytoalexins in different plants [19], suggesting that methylated flavonoids in *Amborella* may also be involved in defense against biotic stress. In addition, the phenylpropanoid pathway derivative methylcinnamate acts as a signaling molecule in plant–insect interactions [20]. Methylcinnamate and its associated compounds, 3,5-dimethoxycinnamic acid and 4-hydroxy-3-methoxycinnamate, were identified in *Amborella* leaves, suggesting that they could be mediators of *Amborella* and insect relations.

Intrigued by the occurrence of multiple glycosylated and methylated specialized metabolites, the fully sequenced *Amborella* genome was explored to identify candidate genes encoding modification enzymes of these compounds [14] (Figure 3 and Figure 4). Phylogenetic analysis showed that a large number of *Amborella* UGTs (22 out of 87) belonged to group L (Figure 3). Retention of duplicated genes after whole-genome duplication (WGD) may have led to the large group L UGTs in *Amborella*. On the other hand, recent gene duplications after the divergence of *Amborella* from other flowering plants may have also contributed to the expansion of group L UGTs, as suggested by W1NFN9 and W1NFL7 that share 97.1% identity and 97.5% similarity (Figure 3). Group L UGTs from different plants have shown to form glycosides and glucose esters of a wide range of compounds, such as flavonoids, isoflavonoids, benzoic acid derivatives, cinnamic acid derivatives, lignans, hydroxy coumarins, phenylethanoids, hydroquinones, diterpenes, triterpenes, glucosinolates, epoxy sesquiterpenoids, auxins, and xenobiotics [21,22,23,24,25,26,27]. Functional characterization of the group L UGTs in *Amborella* will help understand whether they are responsible for generating the diverse glycosylated specialized metabolites reported here. *Amborella* UGTs are absent in groups B, N, and P (Figure 3). Although the activity of group N UGTs has not been elucidated, group B UGTs are active towards flavonoids, benzoic acid derivatives, and xenobiotics [23,25], whereas group P UGTs glycosylate monoterpenes and triterpenes [26,28]. The lack of groups B and P UGTs in *Amborella* suggests that glycosylation of flavonoids and terpenoids relies on UGTs in other phylogenetic groups (e.g., group L).

Seven *Amborella* OMTs are clustered with plant CCoA OMTs (bootstrap value 89) and may be involved in monolignol biosynthesis (Figure 4). Interestingly, of the seven *Amborella* CCoA OMTs, five group with two CCoA OMTs from the Caryophyllales, *Sl*CCoAOMT and *Mc*CCoAOMT, which exhibited activities towards caffeoyl esters and a broad range of flavonoid substrates [29]. U5CX90 is located in the branch of CCoA OMTs from various plant species for lignin biosynthesis. On the other hand, W1PM29 is more distantly related to the other six *Amborella* CCoA OMTs within the same clade (Figure 4). Thirteen *Amborella* OMTs are clustered together and group with plant OMTs that use hydroxycinnamic acids, flavonoids or alkaloids as substrates. Notably, another OMT of this clade, W1NLC7, is strongly associated (bootstrap value 72) with two monocot COMTs, *Zm*COMT and *Ta*OMT2, which are able to methylate caffeic acid (Figure 4). The recombinant *Ta*OMT2 protein also carried out sequential methylations of the flavone tricetin [30]. These phylogenetic associations of *Amborella* OMTs with plant OMTs of various activities instigate an exciting next step of characterization of their biochemical properties.

Overall, *Amborella* tissues are rich in phenolic specialized metabolites, and its genome is abundant in enzymes that modify the core structure of compounds. In the future, the role of these specialized metabolites may be investigated within the context of *Amborella* interacting with the environment. Functional characterization of the candidate UGTs and OMTs in *Amborella* will allow for comparative analysis with UGTs and OMTs from other plant lineages for convergence or divergence in enzyme evolution. Glycosylation and methylation have shown to improve the bioavailability and bioactivity of the core molecules (e.g., flavonoids) [31,32]. Elucidating the catalytic features of *Amborella* enzymes capable of producing specialized metabolites with unique structures (e.g., regiospecific) will enable valuable pharmaceutical biotechnology applications.

## 4. Materials and Methods

### 4.1. Plant Materials

An *Amborella* plant was obtained from UC Santa Cruz in 2014 by the UC Davis Botanical Conservatory; this plant was propagated vegetatively from an *Amborella* plant that was originally collected from New Caledonia by Dr. Ray Collett (*Amborella* is endemic to New Caledonia). A voucher specimen was deposited at the UC Davis herbarium (No. b.2014.123). Leaf and stem tissues were collected from the *Amborella* plant growing at the UC Davis Botanical Conservatory in April 2017. Cuttings were previously made from this plant in August 2016 for vegetative propagation. Root tissues were obtained from one of the rooted cuttings in April 2017. The plant tissues were harvested using a razor blade and immediately frozen in liquid nitrogen. The leaf, stem, and root tissues were taken and analyzed in triplicate.

### 4.2. Metabolite Analysis

*Amborella* leaves, stems, and roots were ground into fine powder in liquid nitrogen using a mortar and pestle and then freeze-dried. Fifty milligrams of the lyophilized tissue was extracted using 1 mL of 70% methanol with sonication, followed by centrifugation at 13,000 rpm for 10 min. The supernatant was filtered through a syringe filter (MilliporeSigma, Burlington, MA, USA) and subjected to LC-HR-ESI-MS analysis on an ultra-performance liquid chromatography (UPLC) (Waters, Milford, MA, USA) coupled to a Q Exactive mass spectrometer (Thermo Scientific, Waltham, MA, USA) as previously described [33]. Briefly, metabolite separation was conducted using a BEH C_18_ column (Acquity UPLC^®^, 100 mm × 2.1 mm, particle size 1.7 μm; Waters, Milford, MA, USA) at a column temperature of 30 °C and with gradient elution between solvents (A) 0.1% formic acid in water and (B) acetonitrile. The injection volume was 5 μL, and the total run time was 30 min. The gradient was as follows: 0–2 min, 3% B; 2–19 min, 3%–35% B; 19–22 min, 35%–90% B; 22–24 min, 90% B; 24–24.1 min, 90%–3% B; and 24.1–30 min, 3% B. The flow rate was maintained at 0.25 mL min^−1^. The UPLC chromatograms were monitored at 254, 280, and 320 nm.

Mass spectra were obtained in the positive and negative ion modes over the mass range of m/z 120–1500 at an ion spray voltage of 4 and 3 kV, respectively. For both types of analysis, the capillary temperature was kept at 320 °C and source temperature at 200 °C. Sheath gas and auxiliary gas were nitrogen at a flow rate of 35 arbitrary units (arb) and 8 arb, respectively. The stepped normalized collision energy (NCE) for MS/MS analysis was at 15% and 40%.

### 4.3. Metabolite Identification

The raw data obtained from the LC-HR-ESI-MS analysis were analyzed using MS-DIAL version 3.20 [34]. Multiple reference mass spectral libraries were used for querying the unknown metabolites, including MassBank [35], RIKEN tandem mass spectral database for phytochemicals (ReSpect) (http://spectra.psc.riken.jp/), the Global Natural Product Social Molecular Networking system (GNPS) (https://gnps.ucsd.edu/ProteoSAFe/libraries.jsp), the Critical Assessment of Small Molecule Identification (CASMI) 2016 library [36], the Fiehn lab hydrophilic interaction liquid chromatography (HILIC) MS/MS library (available at http://prime.psc.riken.jp/Metabolomics_Software/MS-DIAL/index.html), the Bruker MetaboBASE plant library (https://www.bruker.com/products/mass-spectrometry-and-separations/ms-oftware/metabolomics-spectral-libraries/overview.html), RIKEN PlaSMA (http://plasma.riken.jp/), and Karolinska Institute and Gunma (GIAR) zic-HILIC deconvoluted MS2 spectra in data independent acquisition [37]. Tentative compound identification was based on the weighted similarity score of accurate mass, isotope ratio, and MS/MS spectra with a cut-off value set at 80. The accurate mass tolerance for MS was set at 0.01 Da and MS/MS at 0.05 Da. The tentatively identified metabolites were further examined by careful manual inspection of the matching MS/MS spectra between the *Amborella* metabolites and the authentic standards.

### 4.4. Phylogenetic Analysis

The *Amborella* UGT sequences were obtained from the Ensembl Plants database (https://plants.ensembl.org/info/website/ftp/index.html). There are 87 *Amborella* proteins that contain the conserved PSPG motif and are longer than 340 amino acids, which is the shortest length reported for a functionally characterized plant UGT. The accession numbers are: U5CMK2 (467), U5CNG3 (472), U5CNJ5 (477), U5CRD3 (382), U5CUG8 (447), U5CVP6 (486), U5CWM7 (431), U5CX27 (363), U5CXJ9 (482), U5CXN6 (489), U5CYW0 (453), U5D008 (374), U5D0B7 (562), U5D0T8 (551), U5D1 × 9 (469), U5D276 (470), U5D2S8 (473), U5D3G1 (450), U5D3V5 (434), U5D3V6 (463), U5D531 (408), U5D5 × 3 (411), U5D5 × 9 (438), U5D5Z2 (488), U5D602 (452), U5D7I9 (490), U5D817 (481), U5DB27 (427), U5DB32 (469), U5DBW3 (470), U5DBY1 (482), U5DBY4 (478), U5DCN3 (473), U5DCP2 (463), U5DDJ3 (467), U5DDK2 (467), U5DFQ3 (467), U5DFQ6 (467), U5DI88 (510), U5DI92 (419), U5DI97 (469), W1NFL7 (481), W1NFN9 (481), W1NHX5 (466), W1NIK4 (481), W1NU70 (500), W1NUG7 (539), W1NUV1 (607), W1NV64 (484), W1NVU7 (388), W1NWE7 (342), W1NXD5 (462), W1P188 (462), W1P1M0 (474), W1P302 (465), W1P7W1 (486), W1PA48 (541), W1PAP0 (479), W1PAZ2 (518), W1PBB6 (485), W1PC48 (514), W1PD80 (476), W1PE57 (469), W1PF90 (490), W1PFP0 (486), W1PH20 (465), W1PHN8 (448), W1PJ19 (438), W1PKS4 (481), W1PLB6 (487), W1PLP3 (363), W1PMA9 (471), W1PMY7 (390), W1PP20 (470), W1PP63 (543), W1PPW8 (500), W1PQK6 (475), W1PQX3 (486), W1PRF4 (436), W1PS80 (500), W1PT00 (452), W1PTN8 (451), W1PTT2 (531), W1PV81 (398), W1PVY0 (447), W1PX56 (479), and W1PZW0 (469). The size of the protein is indicated in parenthesis next to the gene identifier.

The *Amborella* OMT sequences were retrieved from the InterPro database for protein sequence analysis and classification (http://www.ebi.ac.uk/interpro/) [38]. Seventeen *Amborella* proteins were found for both domain IPR016461 (*O*-methyltransferase COMT-type) and domain IPR001077 (*O*-methyltransferase) searches, including U5CKV5 (151), U5CUU9 (135), U5CZT3 (87), U5D1A9 (87), U5DBV9 (353), W1NGT5 (248), W1NLC7 (358), W1NP75 (102), W1P041 (352), W1P0K5 (105), W1P0L5 (395), W1P628 (182), W1P633 (219), W1P6Z4 (101), W1P8Q1 (351), W1P8Q7 (361), and W1PG58 (167). An additional protein, W1P9N8 (113), possesses the plant methyltransferase dimerization domain (IPR012967). Furthermore, the class I-like SAM-dependent *O*-methyltransferase (domain IPR002935) family contains seven *Amborella* proteins, including U5CX90 (246), U5CZ55 (226), U5CZN4 (192), U5D0E8 (51), U5D229 (373), U5D520 (141), and W1PM29 (314). A search of SAM-dependent carboxyl methyltransferase (domain IPR005299; putative SABATH OMTs) identified 13 proteins, including U5D0V3 (357), U5DA47 (359), U5DCV1 (358), W1NFR3 (363), W1NGL4 (349), W1NHF8 (358), W1NQ15 (406), W1P226 (347), W1PFG6 (116), W1PG85 (109), W1PGE4 (367), W1PNB8 (387), and W1PZL5 (318). The size of the protein is indicated in parenthesis next to the gene identifier.

For phylogenetic analysis of UGTs, the *Amborella* UGTs and selected UGTs representing different plant UGT phylogenetic groups were aligned using multiple sequence comparison by log-expectation (MUSCLE) [39]. A neighbor-joining tree was constructed in Molecular Evolutionary Genetics Analysis (MEGA7) with 1000 bootstrap replicates [40]. The AGI (*Arabidopsis* sequences) and GenBank (other sequences) accession numbers of the selected plant UGTs are: *At*UGT71B1 (AT3G21750), *At*UGT72B1 (AT4G01070), *At*UGT73B1 (AT4G34138), *At*UGT74B1 (AT1G24100), *At*UGT75B1 (AT1G05560), *At*UGT76B1 (AT3G11340), *At*UGT78D1 (AT1G30530), *At*UGT79B1 (AT5G54060), *At*UGT80A2 (AT3G07020), *At*UGT81A1 (AT4G31780), *At*UGT82A1 (AT3G22250), *At*UGT83A1 (AT3G02100), *At*UGT84A1 (AT4G15480), *At*UGT85A1 (AT1G22400), *At*UGT86A1 (AT2G36970), *At*UGT87A1 (AT2G30150), *At*UGT88A1 (AT3G16520), *At*UGT89A1 (AT1G51210), *At*UGT90A1 (AT2G16890), *At*UGT91A1 (AT2G22590), *At*UGT92A1 (AT5G12890), *Os*UGT94A1 (BAC15998), *Os*UGT96A1 (BAD09654), *Os*UGT97A1 (BAD36519), *Os*UGT98B1 (BAB17059), *Os*UGT99A1 (ABF96059), *Os*UGT701A2 (CAE05712), *Os*UGT709A3 (BAC80059), *Po*UGT95A1 (ACB56927), *Zm*UGT77A1 (CAA31855), and *Zm*UGT93B1 (AAK53551). The *Sg*UGT720A1 sequence was obtained from the supplementary data file of [26].

For phylogenetic analysis of OMTs, the *Amborella* OMTs and selected functionally characterized plant small-molecule OMTs were aligned using MUSCLE. A maximum likelihood tree was constructed in MEGA7 with 100 bootstrap replicates. The GenBank accession numbers of the selected plant OMTs are: *Amm*BMT (AAR24096), *Amm*COMT (AAR24095), *Anm*BAMT (AF198492), *At*3′OMT (AAB96879), *At*IAMT (NP_200336), *At*JMT (AAG23343), *Ca*3′5′OMT (AAA80579), *Ca*OMT2 (AAA86982), *Car*4′OMT (AAR02419), *Car*OMT2 (AAM97497), *Cb*IEMT (AAC01533), *Cb*SAMT (AF133053), *Cj*4′OMT (BAB08005), *Cj*6OMT (BAB08004), *Cj*9OMT (BAA06192), *Cj*CoOMT (BAC22084), *Ge*D7OMT (BAC58012), *Ge*HI4′OMT (BAC58011), *Hv*7OMT (CAA54616), *Lj*HI4′OMT (BAC58013), *Mc*CCoAOMT (AAN61072), *Mp*7OMTa (AAR09598), *Mp*7OMTb (AAR09599), *Mp*8OMT (AAR09600), *Mp*3′OMT (AAR09601), *Mp*4′OMT (AAR09602), *Ms*2′OMT (AAB48059), *Ms*CCoAOMT (AAC28973), *Ms*COMT (AAB46623), *Ms*I7OMT (AAC49928), *Nt*CCoAOMT (AAC49913), *Ob*CVOMT (AAL30423), *Ob*EOMT (AAL30424), *Pc*CCoAOMT (AAA33851), *Pit*AEOMT (AAC49708), *Pit*CCoAOMT (AAD02050), *Pot*CCoAOMT (CAA12198), *Ps*HMM (AAC49856), *Rc*POMT (BAD18975), *Rh*OOMT1 (AAM23004), *Rh*OOMT2 (AAM23005), *Sl*CCoAOMT (AAB61680), *Ta*OMT2 (ABB03907), *Vv*CCoAOMT (CAA90969), *Ze*CCoAOMT (AAA59389), *Ze*COMT (AAA86718), and *Zm*COMT (AAB03364). (*Amm*, *Ammi majus*; *Anm*, *Antirrhinum majus*; *At*, *Arabidopsis thaliana*; *Ca*, *Chrysosplenium americanum*; *Car*, *Catharanthus roseus*; *Cb*, *Clarkia breweri*; *Cj*, *Coptis japonica*; *Ge*, *Glycyrrhiza echinata*; *Hv*, *Hordeum vulgare*; *Lj*, *Lotus japonicas*; *Mc*, *Mesembryanthemum crystallinum*; *Mp*, *Mentha x piperita*; *Ms*, *Medicago sativa*; *Nt*, *Nicotiana tobacum*; *Ob*, *Ocimum basilicum*; *Os*, *Oryza sativa*; *Pc*, *Petroselium crispum*; *Pit*, *Pinus taeda*; *Po*, *Pilosella officinarum*; *Pot*, *Populus trichocarpa*; *Rc*, *Rosa chinensis*; *Rh*, *Rosa hybrida*; *Sg*, *Siraitia grosvenorii*; *Sl*, *Stellaria longipes*; *Ta*, *Triticum aestivum*; *Vv*, *Vitis vinifera*; *Ze*, *Zinnia elegans*; *Zm*, *Zea mays.*)

## Supplementary Materials

The following are available online. Table S1: High-resolution electrospray ionization mass spectrometry (HR-ESI-MS) data of metabolites tentatively identified in leaves of *Amborella trichopoda*. The MS/MS fragments that match those of authentic standards are highlighted in bold. Table S2: High-resolution electrospray ionization mass spectrometry (HR-ESI-MS) data of metabolites tentatively identified in stems of *Amborella trichopoda*. The MS/MS fragments that match those of authentic standards are highlighted in bold. Table S3: High-resolution electrospray ionization mass spectrometry (HR-ESI-MS) data of metabolites tentatively identified in roots of *Amborella trichopoda*. The MS/MS fragments that match those of authentic standards are highlighted in bold. Figure S1: Alignment of *Amborella trichopoda* UDP-dependent glycosyltransferases (UGTs) and selected UGTs representing different plant UGT phylogenetic groups. Figure S2: Alignment of *Amborella trichopoda O*-methyltransferases (OMTs) and selected functionally characterized plant small molecule OMTs.
