# Supplementary material for: Exploring the Phytochemical Landscape of the Early-Diverging Flowering Plant Amborella trichopoda Baill
Source: Molecules. 2019 Oct 23;24(21):3814. doi: 10.3390/molecules24213814 (PMC6864642; doi:10.3390/molecules24213814)
Supplement: Supplementary file 1 [file molecules-24-03814-s001.zip › Figure S1.pdf]

```
U5CYW0       1 ------------------------------------------------------------
W1PAZ2       1 ------------------------------------------------------------
AtUGT81A1    1 ------------------------------------------------------------
W1PTT2       1 ------------------------------------------------------------
U5D3V5       1 ------------------------------------------------------------
W1NUG7       1 ------------------------------------------------------------
W1PC48       1 ------------------------------------------------------------
W1PP63       1 ------------------------------------------------------------
OsUGT701A2   1 ------------------------------------------------------------
W1PA48       1 ------------------------------------------------------------
U5D0T8       1 ------------------------------------------------------------
AtUGT80A2    1 -------------------MPEISPAELAKVSSSSSSSSSSSSGRASVKIEEIEGGAAAS
W1PJ19       1 ------------------------------------------------------------
W1NU70       1 ------------------------------------------------------------
W1NV64       1 ------------------------------------------------------------
W1PKS4       1 ------------------------------------------------------------
AtUGT91A1    1 ------------------------------------------------------------
U5CMK2       1 ------------------------------------------------------------
U5CWM7       1 ------------------------------------------------------------
U5D1X9       1 ------------------------------------------------------------
W1PT00       1 ------------------------------------------------------------
OsUGT94A1    1 ------------------------------------------------------------
AtUGT79B1    1 ------------------------------------------------------------
W1PQK6       1 ------------------------------------------------------------
U5D602       1 ------------------------------------------------------------
U5D5X9       1 ------------------------------------------------------------
U5D5Z2       1 ------------------------------------------------------------
U5D5X3       1 ------------------------------------------------------------
ZmUGT93B1    1 ------------------------------------------------------------
PoUGT95A1    1 ------------------------------------------------------------
U5D0B7       1 ------------------------------------------------------------
AtUGT89A1    1 ------------------------------------------------------------
OsUGT96A1    1 ------------------------------------------------------------
AtUGT92A1    1 ------------------------------------------------------------
U5CRD3       1 ------------------------------------------------------------
W1P7W1       1 ------------------------------------------------------------
U5D7I9       1 ------------------------------------------------------------
W1PFP0       1 ------------------------------------------------------------
OsUGT99A1    1 ------------------------------------------------------------
AtUGT73B1    1 ------------------------------------------------------------
OsUGT98B1    1 ------------------------------------------------------------
W1PF90       1 -----------------------------------------------------------M
W1NIK4       1 ------------------------------------------------------------
W1PQX3       1 ------------------------------------------------------------
W1PPW8       1 ------------------------------------------------------------
W1PS80       1 ------------------------------------------------------------
AtUGT90A1    1 ------------------------------------------------------------
OsUGT97A1    1 ------------------------------------------------------------
W1PH20       1 ------------------------------------------------------------
W1PBB6       1 ------------------------------------------------------------
W1PHN8       1 ------------------------------------------------------------
W1PRF4       1 ------------------------------------------------------------
AtUGT71B1    1 ------------------------------------------------------------
W1P1M0       1 ------------------------------------------------------------
AtUGT72B1    1 ------------------------------------------------------------
W1PMA9       1 ------------------------------------------------------------
W1PP20       1 ------------------------------------------------------------
AtUGT88A1    1 ------------------------------------------------------------
U5DB27       1 ------------------------------------------------------------
U5DBY1       1 ------------------------------------------------------------
U5DBY4       1 ------------------------------------------------------------
U5D276       1 ------------------------------------------------------------
U5DB32       1 ------------------------------------------------------------
W1NHX5       1 ------------------------------------------------------------
W1NVU7       1 ------------------------------------------------------------
W1PAP0       1 ------------------------------------------------------------
W1PD80       1 ------------------------------------------------------------
W1PZW0       1 ------------------------------------------------------------
AtUGT82A1    1 ------------------------------------------------------------
W1NUV1       1 MRGATLVAVTCTVLHILHRHARRTSISHVALPHYTRGDAPFHVQQCSISCTTLLHFTCSN
AtUGT86A1    1 ------------------------------------------------------------
AtUGT87A1    1 ------------------------------------------------------------
W1P188       1 ------------------------------------------------------------
```

```
W1P302        1 ------------------------------------------------------------
AtUGT78D1     1 ------------------------------------------------------------
W1PTN8        1 ------------------------------------------------------------
ZmUGT77A1     1 ------------------------------------------------------------
AtUGT83A1     1 ------------------------------------------------------------
W1NWE7        1 ------------------------------------------------------------
W1NXD5        1 ------------------------------------------------------------
AtUGT75B1     1 ------------------------------------------------------------
AtUGT84A1     1 ------------------------------------------------------------
W1PLB6        1 ------------------------------------------------------------
U5CXN6        1 ------------------------------------------------------------
U5D2S8        1 ------------------------------------------------------------
U5D817        1 ------------------------------------------------------------
W1NFL7        1 ------------------------------------------------------------
W1NFN9        1 ------------------------------------------------------------
U5DBW3        1 ------------------------------------------------------------
W1PE57        1 ------------------------------------------------------------
U5D3G1        1 ------------------------------------------------------------
AtUGT74B1     1 ------------------------------------------------------------
U5DFQ6        1 ------------------------------------------------------------
U5DI88        1 --------------------------------------------------------MCEP
U5DCN3        1 ------------------------------------------------------------
U5D531        1 ------------------------------------------------------------
U5CUG8        1 ------------------------------------------------------------
W1PLP3        1 ------------------------------------------------------------
U5DI92        1 ------------------------------------------------------------
U5D3V6        1 ------------------------------------------------------------
U5DDJ3        1 ------------------------------------------------------------
U5DFQ3        1 ------------------------------------------------------------
U5DI97        1 ------------------------------------------------------------
U5DCP2        1 ------------------------------------------------------------
U5DDK2        1 ------------------------------------------------------------
OsUGT709A3    1 ------------------------------------------------------------
SgUGT720A1    1 ------------------------------------------------------------
U5CX27        1 ------------------------------------------------------------
U5D008        1 ------------------------------------------------------------
W1PMY7        1 ------------------------------------------------------------
AtUGT85A1     1 ------------------------------------------------------------
U5CVP6        1 ------------------------------------------------------------
U5CNJ5        1 ------------------------------------------------------------
U5CNG3        1 ------------------------------------------------------------
U5CXJ9        1 ------------------------------------------------------------
AtUGT76B1     1 ------------------------------------------------------------
W1PV81        1 ------------------------------------------------------------
W1PVY0        1 ------------------------------------------------------------
W1PX56        1 ----------------------------------------------------MDKNLKLQ
W1PS80        1 ------------------------------------------------------------
AtUGT90A1     1 ------------------------------------------------------------
OsUGT97A1     1 ------------------------------------------------------------
W1PH20        1 ------------------------------------------------------------
W1PBB6        1 ------------------------------------------------------------
W1PHN8        1 ------------------------------------------------------------
W1PRF4        1 ------------------------------------------------------------
AtUGT71B1     1 ------------------------------------------------------------
W1P1M0        1 ------------------------------------------------------------
AtUGT72B1     1 ------------------------------------------------------------
W1PMA9        1 ------------------------------------------------------------
W1PP20        1 ------------------------------------------------------------
AtUGT88A1     1 ------------------------------------------------------------
U5DB27        1 ------------------------------------------------------------
U5DBY1        1 ------------------------------------------------------------
U5DBY4        1 ------------------------------------------------------------
U5D276        1 ------------------------------------------------------------
U5DB32        1 ------------------------------------------------------------
W1NHX5        1 ------------------------------------------------------------
W1NVU7        1 ------------------------------------------------------------
W1PAP0        1 ------------------------------------------------------------
W1PD80        1 ------------------------------------------------------------
W1PZW0        1 ------------------------------------------------------------
AtUGT82A1     1 ------------------------------------------------------------
W1NUV1        1 MRGATLVAVTCTVLHILHRHARRTSISHVALPHYTRGDAPFHVQQCSISCTTLLHFTCSN
AtUGT86A1     1 ------------------------------------------------------------
AtUGT87A1     1 ------------------------------------------------------------
W1P188        1 ------------------------------------------------------------
```

```
U5CYW0       1 ---------------------------------------------MVMTVISP-------
W1PAZ2       1 -------------------------MQASAVTQEPSSG-------LDLLC----------
AtUGT81A1    1 -----------------------MQNPSTVTQESAAPV-------FDFFPRLRGLTSRNR
W1PTT2       1 -----------------MIVFSDRFPQPHHQQQDPGQD-------GSSQPDPP-------
U5D3V5       1 --------------------------MALLSSLSLSKH-------LLMLVLLM-------
W1NUG7       1 ------------------------MGLTPQATKPGDER-------ATFLPNYM-------
W1PC48       1 -----------------------------------MKL-------LILLAALI-------
W1PP63       1 ----------------------------------MSVL-------FLLIALCS-------
OsUGT701A2   1 ----------------MELKAMYLYAAVLAVLLCSSVN-------FIQSP-TD-------
W1PA48       1 ---------------------------MEGNRRRRRPN-------AIFMA-FG-------
U5D0T8       1 -----------------------------------MPS-------FVTLH-EV-------
AtUGT80A2   42 GVVIVSEELETNPKTVVASIADETVAESSGTGNKSFSR-------VWTMP-LEGSSSSDR
W1PJ19       1 ------------------------------MEAPPKQH-------FLLMP-WL-------
W1NU70       1 -----------------------------MDNPDNRRH-------FLLFP-WL-------
W1NV64       1 ----------------------------MEAPPPPKQH-------FLLFP-WL-------
W1PKS4       1 ------------------------------MEAPPKHH-------FLLFP-WL-------
AtUGT91A1    1 -----------------------MTNFKDNDGDGTKLH-------VVMFP-WL-------
U5CMK2       1 -------------------------------MTDEVLH-------VAVLS-FS-------
U5CWM7       1 ------------------------------------------------------------
U5D1X9       1 -------------------------------MERENVH-------VVMVP-WL-------
W1PT00       1 ------------------------------MEENEELH-------VLMFP-WL-------
OsUGT94A1    1 ----------------------------MAQAERERLR-------VLMFP-WL-------
AtUGT79B1    1 -------------------------MGVFGSNESSSMS-------IVMYP-WL-------
W1PQK6       1 ---------------------METAAVVGVGHEVRPLH-------VAMFP-WL-------
U5D602       1 ----------------------MQPTKKGLDGRRSRVV-------VLMVP-FP-------
U5D5X9       1 ------------------------------------------------------------
U5D5Z2       1 ----MNEIVKEPLEAHQATFSTSQMNSPTHLNSLSAQHAPSGGVVVLVVP-FP-------
U5D5X3       1 ---------------------------------------------MGWWCYWP-------
ZmUGT93B1    1 ----------------------------MAVDTMESVA-------VVAVP-FP-------
PoUGT95A1    1 --------------MDNETQIKKRKLQTATMEAKATGE-------IFVVP-FF-------
U5D0B7       1 ----------------------------------METE-------IVVIP-FH-------
AtUGT89A1    1 ------------------MNRAMPQPRPETIRGSLKPH-------IMVFP-YP-------
OsUGT96A1    1 --------------------MAATTTNGVPSRRRRRPH-------VLVLP-LP-------
AtUGT92A1    1 ----------------------------MAEAKPRNLR-------IVMFP-FM-------
U5CRD3       1 ------------------------------MEPTKLLH----------------------
W1P7W1       1 -------------------MEARHGNATGDVSCGEPKR-------IVMLP-FL-------
U5D7I9       1 ---------------------------------MVVEH-------VVMMP-FM-------
W1PFP0       1 ---------------------------------MVVEH-------VVMMP-FM-------
OsUGT99A1    1 ---------------------------------MAAAH-------FVFVP-LM-------
AtUGT73B1    1 ---------------------------MGTPVEVSKLH-------FLLFP-FM-------
OsUGT98B1    1 ------------------MDDDTAGHASGGYTTTAAAH-------FVLVP-MM-------
W1PF90       2 EFTDHNPNSTSIASQHSTNGTTIVTENRNPNPNGAQPY-------VLMFP-VM-------
W1NIK4       1 --------------------------MFGVGDSDQRHH-------VVVFP-LM-------
W1PQX3       1 ------------------MVMEGEGEGEGEGESEQRHN-------VVLFP-FM-------
W1PPW8       1 ------------------------MVMEGEGESEQSHN-------VVLFP-FM-------
W1PS80       1 ------------------------MVMEGEGESEQSHN-------VVLFP-FM-------
AtUGT90A1    1 -----------------------------MSVSTHHHH-------VVLFP-FM-------
OsUGT97A1    1 ---------MPASAAAATRPAAEGVGGNDGATLAGRDH-------VVVFP-LM-------
W1PH20       1 --------------------------MPEEAPLTSSHH-------IVVFP-FM-------
W1PBB6       1 -------MAAAKTEPEFTRGLAPVESSGLSIERTSAPH-------IAVFP-FM-------
W1PHN8       1 -------MATTTTEPEIIRRLTPVEPTRLSMERTSAPH-------IAVFP-FM-------
W1PRF4       1 ----------------------------MAGQEHRRPH-------VAMLP-SS-------
AtUGT71B1    1 ----------------------------------MKVE-------LVFIP-SP-------
W1P1M0       1 -------------------------------METQRPH-------VAIFP-SP-------
AtUGT72B1    1 ------------------------------MEESKTPH-------VAIIP-SP-------
W1PMA9       1 ----------------------------MAMEAQTGTH-------VAILP-SP-------
W1PP20       1 ------------------------------MEAKTGAH-------VAILP-SP-------
AtUGT88A1    1 ---------------------------------MGEEA-------IVLYP-AP-------
U5DB27       1 ----------------------------------MATQ-------LVLFP-SP-------
U5DBY1       1 ----------------------------------MATQ-------LVFFP-SP-------
U5DBY4       1 ------------------------------MDEAAKVQ-------VVVFP-LP-------
U5D276       1 ------------------------------MENVTKVQ-------VLMFP-LP-------
U5DB32       1 ------------------------------MENAREVQ-------VVMFP-LP-------
W1NHX5       1 ---------------------------------MKEMV-------VVLYP-SP-------
W1NVU7       1 ----------------------------MASREMKDRV-------VVLYP-SP-------
W1PAP0       1 ---------------------------MCLHSFKMSES-------IVLYP-SP-------
W1PD80       1 ---------------------------------------------MVFFP-SP-------
W1PZW0       1 ----------------------------------MKQE-------VVLYP-SP-------
AtUGT82A1    1 ------------------------------MKVTQKPK-------IIFIP-YP-------
W1NUV1      61 ASFHVRRYFTSCVTMLHFTHGVTPLHTSVGTPAMDWCR-------LLVAPTSSMRRS---
AtUGT86A1    1 ----------------------------MERAKSRKPH-------IMMIP-YP-------
AtUGT87A1    1 -------------------------MNPIKPQPLGVRH-------VVAMP-WP-------
W1P188       1 ------------------MAEQKIPVVQEPDPGHAEPM-------IVAIP-YP-------
W1P188       1 ------------------------------------------------------------
```

```
W1P302        1  -----------------MLGIGKSDTRNFESLCSGVRH-------VVAVP-FP-------
AtUGT78D1     1  --------------------------MTKFSEPIRDSH-------VAVLAFFP-------
W1PTN8        1  -----------------------------MAEAATRAH-------VALLA-FP-------
ZmUGT77A1     1  -------------------------MAPADGESSPPPH-------VAVVA-FP-------
AtUGT83A1     1  -------------------------MDNNSNKRMGRPH-------VVVIP-YP-------
W1NWE7        1  ---------------------------------------------MEFFYTYK-------
W1NXD5        1  ---------------------------------MGVQH-------ALMLP-YP-------
AtUGT75B1     1  ---------------------------------MAPPH-------FLLVT-FP-------
AtUGT84A1     1  -------------------------MVFETCPSPNPIH-------VMLVS-FQ-------
W1PLB6        1  -----------------------------MGAIKERAH-------ILLVT-FP-------
U5CXN6        1  -----------------------------MVGIEEKVH-------VLVVT-FP-------
U5D2S8        1  ---------------------------------------------MGAIS-YW-------
U5D817        1  -----------------------------MVTLEVNIH-------ILLVT-FP-------
W1NFL7        1  -----------------------------MSAIEVNIH-------ILLVT-FP-------
W1NFN9        1  -----------------------------MSAIEVNIH-------ILLVT-FP-------
U5DBW3        1  -----------------------------MEMGSETKH-------VMLVC-YA-------
W1PE57        1  -----------------------------MGEIREPTH-------VMLVF-FS-------
U5D3G1        1  ----------------------------------MAHH-------VLMVC-SP-------
AtUGT74B1     1  ---------------------------MAETTPKVKGH-------VVILP-YP-------
U5DFQ6        1  ---------------------------MEKGKQTRRPH-------VLVFP-YP-------
U5DI88        5  GFVVDEEEHAPSYKRHTLLFQTSKKPEEMEVEKATRGH-------VLVLP-YT-------
U5DCN3        1  ----------------------------MEVEKGRGGH-------VLVLP-YP-------
U5D531        1  ----------------------------MEEEKDKSAH-------VLVIP-SP-------
U5CUG8        1  ----------------------------MEEEKGKSAH-------VLVLL-SL-------
W1PLP3        1  ----------------------------MEEEKGKSAH-------VLVLL-SL-------
U5DI92        1  ----------------------------MEEETAKSAH-------VLLLP-YP-------
U5D3V6        1  ------------------------------MEKDKSVH-------VLLLT-YP-------
U5DDJ3        1  ----------------------------MEEERGKSAH-------VLLLP-YP-------
U5DFQ3        1  ----------------------------MEVGKAKSAH-------VLLLP-YP-------
U5DI97        1  ----------------------------MEEDKSKSAH-------VLLLP-YP-------
U5DCP2        1  ----------------------------MEEGKAKSSH-------VLVLP-YP-------
U5DDK2        1  ----------------------------MEEGKAKSSH-------VLVLP-YP-------
OsUGT709A3    1  -----------------------------MGTAPAPAH-------VLVFP-WP-------
SgUGT720A1    1  ----------------------------MAEQAHDLLH-------VLLFP-FP-------
U5CX27        1  -----------------------------------MCY----------------------
U5D008        1  ---------------------------------------------------ML-------
W1PMY7        1  ------------------------------------------------------------
AtUGT85A1     1  -------------------------MGSQIIHNSQKPH-------VVCVP-YP-------
U5CVP6        1  ----------------------------MGAQLEVKPH-------AVCIP-YP-------
U5CNJ5        1  ----------------------------MASTVSTKPH-------AVCVP-FP-------
U5CNG3        1  ----------------------------MGSENENQAH-------AVCVP-YP-------
U5CXJ9        1  ----------------------------MGSDYVQKPH-------AVCVP-FP-------
AtUGT76B1     1  -----------------------------METRETKPV-------IFLFP-FP-------
W1PV81        1  -----------------------------MDLDHRPTQ-------LVFFP-YP-------
W1PVY0        1  -------------------------MAENRVSTPYTKH-------LVLFS-PP-------
W1PX56        9  TLVLHFLNSRLTLPIKPSTPLPNSLMAENRVSMPYTGH-------LVLFS-YP-------
AtUGT90A1     1  -----------------------------MSVSTHHHH-------VVLFP-FM-------
OsUGT97A1     1  ---------MPASAAAATRPAAEGVGGNDGATLAGRDH-------VVVFP-LM-------
W1PH20        1  --------------------------MPEEAPLTSSHH-------IVVFP-FM-------
W1PBB6        1  -------MAAAKTEPEFTRGLAPVESSGLSIERTSAPH-------IAVFP-FM-------
W1PHN8        1  -------MATTTTEPEIIRRLTPVEPTRLSMERTSAPH-------IAVFP-FM-------
W1PRF4        1  ----------------------------MAGQEHRRPH-------VAMLP-SS-------
AtUGT71B1     1  ----------------------------------MKVE-------LVFIP-SP-------
W1P1M0        1  -------------------------------METQRPH-------VAIFP-SP-------
AtUGT72B1     1  ------------------------------MEESKTPH-------VAIIP-SP-------
W1PMA9        1  ----------------------------MAMEAQTGTH-------VAILP-SP-------
W1PP20        1  ------------------------------MEAKTGAH-------VAILP-SP-------
AtUGT88A1     1  ---------------------------------MGEEA-------IVLYP-AP-------
U5DB27        1  ----------------------------------MATQ-------LVLFP-SP-------
U5DBY1        1  ----------------------------------MATQ-------LVFFP-SP-------
U5DBY4        1  ------------------------------MDEAAKVQ-------VVVFP-LP-------
U5D276        1  ------------------------------MENVTKVQ-------VLMFP-LP-------
U5DB32        1  ------------------------------MENAREVQ-------VVMFP-LP-------
W1NHX5        1  ---------------------------------MKEMV-------VVLYP-SP-------
W1NVU7        1  ----------------------------MASREMKDRV-------VVLYP-SP-------
W1PAP0        1  ---------------------------MCLHSFKMSES-------IVLYP-SP-------
W1PD80        1  ---------------------------------------------MVFFP-SP-------
W1PZW0        1  ----------------------------------MKQE-------VVLYP-SP-------
AtUGT82A1     1  ------------------------------MKVTQKPK-------IIFIP-YP-------
W1NUV1       61  ASFHVRRYFTSCVTMLHFTHGVTPLHTSVGTPAMDWCR-------LLVAPTSSMRRS---
AtUGT86A1     1  ----------------------------MERAKSRKPH-------IMMIP-YP-------
AtUGT87A1     1  -------------------------MNPIKPQPLGVRH-------VVAMP-WP-------
W1P188        1  ------------------MAEQKIPVVQEPDPGHAEPM-------IVAIP-YP-------
W1P188        1  ------------------------------------------------------------
```

```
U5CYW0        9 -----RRS-------------------------ITEK---VLQSL------SLSPRSSSR-
W1PAZ2       19 -----KFG---------------YLSF--------DSNFSSRGFGQLCLPHSSSSCGAKF
AtUGT81A1    31 SPCSNSDG---------------YALSSSNALYFNG---FRTLP------SRRMGKTLAS
W1PTT2       31 -----AAS---------------GLEP-----TP-----LHLRL-----AAARDFGGLR-
U5D3V5       21 -----EEN---------------EVHC-----KNSQ---PCDLS------SHDYMSLTSL
W1NUG7       23 -----ASN---------------LSFD--------A---SRTRL------ASRDFGGLTG
W1PC48       12 -----SSH---------------HVSA-----TLEL---DSADL------SSLKTLNLEG
W1PP63       13 -----PFN---------------FIQS-----PM-D---FGPLN------FLQTSDSAA-
OsUGT701A2   30 -----VLGPVA------------LLEP-----TPSSARDFGAVV------SDA---PFA-
W1PA48       19 -----TKG---------------DVLP-----IA-A---IATAL------ASDQQ-HYN-
U5D0T8       11 -----KKC---------------SEIC-----FL-K---MLARM------ATVKNDGT--
AtUGT80A2    94 AESSSTNQPRLDKSKTERQQKVTHILAEDAAKIFDDKISAGKKLKLLNRIATVKHDGT--
W1PJ19       16 -----AMG---------------HILP-----FF-Q---LAKRL------ADA---GIS-
W1NU70       17 -----AFG---------------HILP-----FF-H---LAKRL------ADA---KIS-
W1NV64       18 -----AFG---------------HIIP-----FF-Q---LAKRL------ADA---GIS-
W1PKS4       16 -----ALG---------------HIIP-----FF-R---LAKRL------ADA---GIS-
AtUGT91A1    23 -----AFG---------------HMVP-----YL-E---LSKLI------AQK---GHK-
U5CMK2       15 -----AFG---------------HLIP-----FL-E---LSKIL------AQK---GHK-
U5CWM7        1 -----MEGQCR------------SILP-----FF-E---LSKAL------ALK---GIR-
U5D1X9       15 -----AFG---------------HMIP-----FF-E---LSKAL------AQK---GIT-
W1PT00       16 -----AHG---------------HITP-----FF-E---LAKKL------VER---EIT-
OsUGT94A1    18 -----AHG---------------HINP-----YL-E---LATRL------TTTSSSQIDV
AtUGT79B1    21 -----AFG---------------HMTP-----FL-H---LSNKL------AEK---GHK-
W1PQK6       25 -----AFG---------------HINP-----YV-Y---LANKL------AGDA--ATR-
U5D602       24 -----IQG---------------HLNA-----LL-N---FARSL------AEC---GLR-
U5D5X9        1 -----MIN---------------QLKP-----TL-S---SSSTM------SGPPN-----
U5D5Z2       49 -----AAS---------------HLNQ-----LL-N---FARVL------AEH---GLP-
U5D5X3        9 --------------------------------LTAS------------------------
ZmUGT93B1    18 -----AQG---------------HLNQ-----LL-H---LSLLL------ASR---GLS-
PoUGT95A1    32 -----GQG---------------HLFP-----AM-E---LCKNI------SAH---NYN-
U5D0B7       12 -----GQG---------------HIFP-----CI-E---LCNQL------ARL---NLK-
AtUGT89A1    28 -----AQG---------------HLLP-----LL-D---LTHQL------CLR---GLT-
OsUGT96A1    26 -----SRG---------------HLLP-----LL-D---FAHRL------STRH--GVA-
AtUGT92A1    18 -----GQG---------------HIIP-----FVALALRLEKIM------IMNRANKTT-
U5CRD3        9 -----AVS---------------HYRQ--------H------------------------
W1P7W1       27 -----ARG---------------HLIH-----FV-A---LAELL------AART--PFA-
U5D7I9       13 -----AQG---------------HLIP-----FA-A---LAELL------SELH--SLK-
W1PFP0       13 -----AQG---------------HLIP-----FA-A---LAELL------SELH--PLK-
OsUGT99A1    13 -----AQG---------------HLIP-----AV-D---TALLL------ATH---GAF-
AtUGT73B1    19 -----AHG---------------HMIP-----TL-D---MAKLF------ATK---GAK-
OsUGT98B1    28 -----AQG---------------HAIP-----MT-D---MARLL------AEHG--AAR-
W1PF90       47 -----SQG---------------HMIP-----MI-D---LARMI------AAR---GLA-
W1NIK4       20 -----AQG---------------HMLP-----MI-D---LARML------AKR---GLA-
W1PQX3       28 -----AQG---------------HMIP-----ML-D---IARML------ADH---RLA-
W1PPW8       22 -----AQG---------------HMIP-----ML-D---IARML------ANH---GLA-
W1PS80       22 -----AQG---------------HMIP-----VL-D---IARML------ADH---GLA-
AtUGT90A1    17 -----SKG---------------HIIP-----LL-Q---FGRLL----LRHHRKEPTIT-
OsUGT97A1    37 -----SKG---------------HMIP-----LL-H---FAAAL------AVHHGDHLR-
W1PH20       20 -----AQG---------------HTIP-----LL-Y---LCNAL------SSM---GHR-
W1PBB6       39 -----SRG---------------HAIP-----LL-H---LCATF------LRR---GLE-
W1PHN8       39 -----SRG---------------HAIP-----LL-H---LCATL------LRR---GLE-
W1PRF4       18 -----GMG---------------HLAP-----FT-R---LATLL------AKDH--GFT-
AtUGT71B1    12 -----GVG---------------HIRA-----TT-A---LAKLL------VASDN-RLS-
W1P1M0       15 -----GLG---------------HLIP-----LA-A---FAQQL------ATIH--GFS-
AtUGT72B1    16 -----GMG---------------HLIP-----LV-E---FAKRL------VHLH--GLT-
W1PMA9       18 -----GMG---------------HLIP-----LV-A---FSKRL------VATH--SFS-
W1PP20       16 -----GMG---------------HLIP-----LV-V---FAKHL------AATH--SFS-
AtUGT88A1    13 -----PIG---------------HLVS-----MV-E---LGKTI-----LSKNPSLSIH-
U5DB27       12 -----AVG---------------HLVS-----MV-E---LAKRL------LHHNPTAFS-
U5DBY1       12 -----GVG---------------HLVS-----MV-E---LAKRL------LHHNPTAFS-
U5DBY4       16 -----LVG---------------HLVP-----TV-E---LAKLL------LGHRS-LLS-
U5D276       16 -----PIG---------------HLVT-----TV-E---LAKIL------IHNHS-LLS-
U5DB32       16 -----PIG---------------HLVT-----MV-E---LAKTL------IRNHS-LLS-
W1NHX5       13 -----CSG---------------HLMS-----MV-E---LAKLI-----LHRHPNLSTT-
W1NVU7       18 -----AIR---------------HLLS-----MV-E---LAKLI----L-HHSPKLYIT-
W1PAP0       19 -----GMG---------------HLVS-----MM-E---LGKLI----L-HHYPTFSIH-
W1PD80        8 -----GIG---------------HLIP-----MV-E---LGKLI----L-RHHPSFSIT-
W1PZW0       12 -----GMG---------------HLVS-----LV-E---LGKLI----L-RHHPTFSIT-
AtUGT82A1    16 -----AQG---------------HVTP-----ML-H---LASAF------LSR---GFS-
W1NUV1      111 -----LKGRCSLSATCWKLEFGSHIIP-----SV-Q---LAKKL------ASK---GLT-
AtUGT86A1    18 -----LQG---------------HVIP-----FV-H---LAIKL------ASH---GFT-
AtUGT87A1    21 -----GRG---------------HINP-----ML-N---LCKSL------VRRDP-NLT-
W1P188       28 -----GQG---------------HINP-----MM-R---LCMRL------ASK---GAL-
W1P188        1 ------------------------------------------------------------
```

```
W1P302       29 -----GQG---------------HINP-----MM-H---LCKRL------ASK---GAA-
AtUGT78D1    21 -----VGA---------------HAGP-----LL-A---VTRRL------AAASPSTI--
W1PTN8       17 -----FGT---------------HAAP-----LL-A---LALNL------ATTSPSTSL-
ZmUGT77A1    21 -----FSS---------------HAAV-----LL-S---IARAL---A--AAAAPSGAT-
AtUGT83A1    21 -----AQG---------------HVLP-----LI-S---FSRYL------AKQ---GIQ-
W1NWE7        9 -----GNN-------------------------VL-D---FKRPL-----------DGLK-
W1NXD5       13 -----AQG---------------HIIP-----MM-E---LSRRM------EEH---GFI-
AtUGT75B1    13 -----AQG---------------HVNP-----SL-R---FARRL------IKRT--GAR-
AtUGT84A1    21 -----GQG---------------HVNP-----LL-R---LGKLI------ASK---GLL-
W1PLB6       17 -----AQG---------------HVKP-----AI-R---LARRL------ASDN--RLR-
U5CXN6       17 -----AQG---------------HVNL-----AI-R---LARCL------AASNT-SLL-
U5D2S8        8 -----SPS---------------QDKA-----MLSQ---QSDLL------AASNA-SLL-
U5D817       17 -----AQG---------------HVKP-----AI-R---FARRL------TASNA-SLL-
W1NFL7       17 -----AQG---------------HVKP-----AI-R---FARRL------TASNA-SLL-
W1NFN9       17 -----AQG---------------HVKP-----AI-R---FARRL------TASNA-SLL-
U5DBW3       17 -----AQG---------------HINP-----VL-Q---FAGRL------ASR---GIS-
W1PE57       17 -----AQG---------------HLNP-----ML-Q---FAGHL------SSR---GIT-
U5D3G1       12 -----AQG---------------HINP-----AL-E---LAKRL------VKK---DLR-
AtUGT74B1    19 -----VQG---------------HLNP-----MV-Q---FAKRL------VSK---NVK-
U5DFQ6       19 -----TQG---------------HINP-----ML-Q---FAKCL------TRE---LLS-
U5DI88       50 -----TQG---------------HINP-----ML-Q---FSRRL------THK---GLR-
U5DCN3       18 -----TQG---------------HINP-----ML-Q---FSRRL------THK---GLR-
U5D531       18 -----FPS---------------HINP-----ML-H---FADRL------ASK---GLR-
U5CUG8       18 -----CRG---------------HINP-----ML-H---FADRL------AIN---GLR-
W1PLP3       18 -----CRG---------------HINP-----ML-H---FADRL------ASN---GLR-
U5DI92       18 -----LLG---------------HINP-----LL-Q---FADRL------ASH---GFR-
U5D3V6       16 -----TQG---------------HINP-----LL-Q---FAGRL------ASN---GLR-
U5DDJ3       18 -----SQG---------------HINP-----LL-Y---FADRL------ASN---GLR-
U5DFQ3       18 -----SQG---------------HINP-----LL-Q---FADRL------ASN---CLC-
U5DI97       18 -----IQG---------------HINP-----LL-Q---FADRL------VGK---GLK-
U5DCP2       18 -----IQG---------------HINP-----LL-Q---FADGL------ASK---GVK-
U5DDK2       18 -----IQG---------------HINP-----LL-Q---FADRL------ASK---GVK-
OsUGT709A3   17 -----IQG---------------HLNV-----ML-H---LAVAL------AGA---GVH-
SgUGT720A1   18 -----AEG---------------HIKP-----FL-C---LAELL------CNA---GFH-
U5CX27        4 -----GHG---------------SFEP---------------------------------
U5D008        3 -----GTG------------------P-----------------------------GRV-
W1PMY7        1 ---------------------------------------MGKNL----------------
AtUGT85A1    21 -----AQG---------------HINP-----MM-R---VAKLL------HAR---GFY-
U5CVP6       18 -----AQG---------------HVSP-----MM-Q---LAKLL------HSK---GFH-
U5CNJ5       18 -----AQS---------------HINA-----MV-Q---LAKLL------HTR---GFH-
U5CNG3       18 -----AQG---------------HINP-----MM-Q---LAKLL------HAR---GFH-
U5CXJ9       18 -----AQG---------------HITP-----MM-Q---LAKLL------HAR---GFH-
AtUGT76B1    17 -----LQG---------------HLNP-----MF-Q---LANIF------FNR---GFS-
W1PV81       17 -----LQG---------------HLTP-----ML-Q---LAQSL------LSLNP-SFK-
W1PVY0       21 -----FQG---------------HINP-----MV-Q---LAHIL------HSR---GFF-
W1PX56       54 -----YQG---------------HINP-----MI-Q---LARTL------HSR---GFF-
AtUGT90A1    17 -----SKG---------------HIIP-----LL-Q---FGRLL----LRHHRKEPTIT-
OsUGT97A1    37 -----SKG---------------HMIP-----LL-H---FAAAL------AVHHGDHLR-
W1PH20       20 -----AQG---------------HTIP-----LL-Y---LCNAL------SSM---GHR-
W1PBB6       39 -----SRG---------------HAIP-----LL-H---LCATF------LRR---GLE-
W1PHN8       39 -----SRG---------------HAIP-----LL-H---LCATL------LRR---GLE-
W1PRF4       18 -----GMG---------------HLAP-----FT-R---LATLL------AKDH--GFT-
AtUGT71B1    12 -----GVG---------------HIRA-----TT-A---LAKLL------VASDN-RLS-
W1P1M0       15 -----GLG---------------HLIP-----LA-A---FAQQL------ATIH--GFS-
AtUGT72B1    16 -----GMG---------------HLIP-----LV-E---FAKRL------VHLH--GLT-
W1PMA9       18 -----GMG---------------HLIP-----LV-A---FSKRL------VATH--SFS-
W1PP20       16 -----GMG---------------HLIP-----LV-V---FAKHL------AATH--SFS-
AtUGT88A1    13 -----PIG---------------HLVS-----MV-E---LGKTI-----LSKNPSLSIH-
U5DB27       12 -----AVG---------------HLVS-----MV-E---LAKRL------LHHNPTAFS-
U5DBY1       12 -----GVG---------------HLVS-----MV-E---LAKRL------LHHNPTAFS-
U5DBY4       16 -----LVG---------------HLVP-----TV-E---LAKLL------LGHRS-LLS-
U5D276       16 -----PIG---------------HLVT-----TV-E---LAKIL------IHNHS-LLS-
U5DB32       16 -----PIG---------------HLVT-----MV-E---LAKTL------IRNHS-LLS-
W1NHX5       13 -----CSG---------------HLMS-----MV-E---LAKLI-----LHRHPNLSTT-
W1NVU7       18 -----AIR---------------HLLS-----MV-E---LAKLI-----L-HHSPKLYIT-
W1PAP0       19 -----GMG---------------HLVS-----MM-E---LGKLI-----L-HHYPTFSIH-
W1PD80        8 -----GIG---------------HLIP-----MV-E---LGKLI-----L-RHHPSFSIT-
W1PZW0       12 -----GMG---------------HLVS-----LV-E---LGKLI-----L-RHHPTFSIT-
AtUGT82A1    16 -----AQG---------------HVTP-----ML-H---LASAF------LSR---GFS-
W1NUV1      111 -----LKGRCSLSATCWKLEFGSHIIP-----SV-Q---LALKL------ASK---GLT-
AtUGT86A1    18 -----LQG---------------HVIP-----FV-H---LAIKL------ASH---GFT-
AtUGT87A1    21 -----GRG---------------HINP-----ML-N---LCKSL------VRRDP-NLT-
W1P188       28 -----GQG---------------HINP-----MM-R---LCMRL------ASK---GAL-
W1P188        1 ------------------------------------------------------------
```

```
U5CYW0        30 -----CSYGVL---EEERGT-----ELTQL----------------GAERTKTVLI---
W1PAZ2        51 G----VTFPGEPKFVGSLKIGKTAAKVQGIGNQFNRFIRLNCERIPIGFASVSSVQKPFD
AtUGT81A1     67 -----LSFNTKSSAGSSLRR--------FISDFNSFIRFHCDKVVPESFASVGGVGLSSD
W1PTT2        55 -----ADPPPA---TVALPS-----------------TASDVTRAIRAALSHQTLTLSAR
U5D3V5        47 NLHGSLSFNETLRASKDFGG----------------FRVRKPGAVVEPGSVQDIVDVIKV
W1NUG7        46 APPHGVLVPSSVHEIANLVR-------------------VAYEADAHFRVAAKGHAHSIQ
W1PC48        38 Y----LSFHDTHLASRDFGD-------------CYHSLPSAILRPKSPQDIAKTLQHVFD
W1PP63        37 -----IDFGRI---FFNSPS--------------AILQPNSVDEISLLLKFISLSSYSKV
OsUGT701A2    58 -----VMRPES---PDDIAL-----------------LLGALSSTAPSPRATVAAVGAG
W1PA48        42 -----VVLITHLAHKYLEAH---------------LVAKGASIVPISTPPVLSMEDAHRT
U5D0T8        34 -----VEFEVTENLPHDDVD--------------HGSEAHTGAVDEETDELIDLQDIQPL
AtUGT80A2    152 -----VEFEVP---ADAIPQ----------PIVVDRGESKNGVCADESIDGVDLQYIPPM
W1PJ19        37 -----ITFLTP---------------------------------PSTPPLSSLSL---
W1NU70        38 -----VTFLAP---ASVLPR-----------------LPSLHLPHHLSLISFHPFSLP
W1NV64        39 -----VTFLTL---PSVFSH--------------LPPLH-----VPHHLSLISFHPVSLP
W1PKS4        37 -----VTYLSP---PSVFPR-----------------LPPLQLPHHQSLITFHPLSLP
AtUGT91A1     44 -----VSFIST---PRNIDR-----------------LLPWLPENLSSVINFVKLSLP
U5CMK2        36 -----VSFIST---PRNIDR------------------LPKIPPNLATLINLVKFELP
U5CWM7        25 -----VSLIST---PRNLQR------------------LPPIPQELAPQINLVGFPLP
U5D1X9        36 -----VSFIST---PRNLQR------------------LPPTPPELAPQINLVGFPLP
W1PT00        37 -----VTICST---ASNLTS------------------IKDQLRETHSSIKTAEISLP
OsUGT94A1     43 V----VHLVST---PVNLAA---------------------VAHRRTDRISLVELHLP
AtUGT79B1     42 -----IVFLLP---KKALNQ-------------------LEPLNLYPNLITFHTISIP
W1PQK6        47 -----VTIVSA---PGNLPQ-------------------ISPSILLPSLITLLPLPLP
U5D602        45 -----VVFACS---DTHMRQ----------------ATQRAEGWDPFAIANLSFHLLPLP
U5D5X9        21 -----LNFAFR---PRGPKS-------------------GGASGAAAGAEPPKPAAHLRLR
U5D5Z2        70 -----VVFACS---ETHARQ----------------TTHRSGGFNCASNPNLRFHILPFP
U5D5X3        13 -----VAWIAP---PFPTSG------------------------FTSSPSLNFILPLDP
ZmUGT93B1     39 -----VHYAAP---PPHVRQ----------------ARARVHGWDPRALGSIRFHDLDVP
PoUGT95A1     53 -----VTLIIPSHLSSSIPS-----------------TFSNHSSFIHVTEISVAASPPP
U5D0B7        33 -----TTALIP-----------------------------SPISSSLRLHPLVQIMETSTH
AtUGT89A1     49 -----VSIIVT---PKNLPY------------------LSPLLSAHPSAVSVVTLPFP
OsUGT96A1     48 -----LTVAVT---ASDLPL-------------------LSAFLASTPLAAALPF---
AtUGT92A1     46 -----ISMINT---PSNIPK-------------------IRSNLPPESSISLIELPFN
U5CRD3        17 ------------PSQHCH------------------------TPIHATPILAALPFN
W1P7W1        49 -----ITLVST---PKNAQM-----------------MRSILDTATAAAITVVELPFC
U5D7I9        35 -----ITLVNT---PHNASK-----------------LRSMMNPKAAGAIDVAELPFS
W1PFP0        35 -----ITLVNT---PHNASK-----------------LRSMMNPKAAGAIDVAEIPFS
OsUGT99A1     34 -----CTVVAT---PATAAR-----------------VRPTVDSARRSGLPVRLAEFPLD
AtUGT73B1     40 -----STILTT---PLNAKL-------------FFEKPIKSFNQDNPGLEDITIQILNFP
OsUGT98B1     50 -----VSLVVT---PVNAAR-----------------MAGFAAGVEEAGLPVQLVEL---
W1PF90        68 -----VAVATT---PLNAAR-------------------------IPSVPLLRVLELPFP
W1NIK4        41 -----VSIVTT---PLNASR-----------------IRSILDRAAASGHRMQILEIEFP
W1PQX3        49 -----VTLVTT---PLNAVR---------------------IRAAAWSPRVRVLEMEFP
W1PPW8        43 -----VTLVTT---PLNAVW-----------------IRTILDRAASSSCRVRVLDIEFP
W1PS80        43 -----VTLVTT---PLNAVW-----------------IRPILDRAASSSCRVRVLDIEFP
AtUGT90A1     43 -----VTVFTT---PKNQPF---------------------ISDFLSDTPEIKVISLPFP
OsUGT97A1     61 -----VTLVTT---PANLAF---------------------ARRRLPPSPSVRVVAIPFP
W1PH20        41 -----STIITT---PLNAIS-------------------MRRTLDCQNPKIRLIELPFP
W1PBB6        60 -----LTIFST---PSNTPF---------------------YRQCLPQDPKIRIIDLPFP
W1PHN8        60 -----LTIFST---PSNSPF---------------------YRQSLPKDSGIRIIDLPFP
W1PRF4        40 -----VTILVP---TSLVSV-----------------PESRTLDALFSSGLDIHRL-PY
AtUGT71B1     35 -----VTLIVI---PSRVSD-----------------DASSSVYTNSEDRLRYILLPAR
W1P1M0        37 -----VTFINVSTGSSPMQD-----------------SILAGLEQTCKGFVKSVELPMM
AtUGT72B1     38 -----VTFVIAGEGPPSKAQ---------------------RTVLDSLPSSISSVFLPPV
W1PMA9        40 -----ITFINLITDSSAAQS-----------------SVLSTLNDVGLSSLQSVDLPPA
W1PP20        38 -----ITFINL---TTDSSA--------------AQSSVLSNLNDVGLSSVQFVDLPPA
AtUGT88A1     38 -----IILVPP---PYQPES-----------------TATYISSVSSSFPSITFHHLPAV
U5DB27        36 -----ITILTT------------------------------QSPFGPGAAATYIAN---
U5DBY1        36 -----ITMLTIES-PFGAAT----------------AAAYMAKVSASAPPALQFLPLPPP
U5DBY4        39 -----ITILTPHDHPFNPSS--------------TAPYIERTSSSSFPLNRLRFLQLPRV
U5D276        39 -----ITILTPHDHPFDAPT--------------TAPYIARISASYSPTRLRFLNLPPV
U5DB32        39 -----ITILTPHDHPFDAAT--------------TDPYISRISSSYSPTRLRFLKLPPV
W1NHX5        38 -----ILTVNP---PFYVGN-----------------TAPYIAYISNEFPDIAFLELSPP
W1NVU7        43 -----ILNVNP---PFDVGN-----------------TAPYIGRISSEFPAIAFLELEPP
W1PAP0        44 -----VLTFNP---PFHTGA-----------------TAPYMSRIAAEYPAISFHEL---
W1PD80        33 -----ILITTP---PFETGA-----------------TAPYMSRVASEIQAITFIELPPV
W1PZW0        37 -----VLITKP---PFDTGA-----------------TAEYVARVASQVSAITFHELPPA
AtUGT82A1     37 -----PVVMTP---ESIHRR---------------------ISATNEDLGITFLALSD-
W1NUV1       147 -----ITFVNT---EAVHAQ----------TVKAYNNSLSKTLNDSIDCSNSTDDIFFG
AtUGT86A1     39 -----ITFVNTDSIHHHIST----------------AHQDDAGDIFSAARSSGQHDIRYT
AtUGT87A1     44 -----VTFVVT---EEWLGF---------------------IGSDPKPNRIHFATL-PN
W1P188        49 -----IRIVVT---EEWLSF-------------------MTSAQEPPPNVRLLSIPN-
W1P188         1 ------------------------------------------------------------
```

```
W1P302        50 -----ISFVVT---KEWVGF----------------------MSGQPIPPNVRLCPIPN-
AtUGT78D1     44 -----FSFFNT---ARSNAS----------------------LFSSDHPENIKVHDV-SD
W1PTN8        41 -----FTFFST---PQSLSK-----------------LSSSITICSSKATNLRFLPLPEN
ZmUGT77A1     46 -----LSFLST---ASSLAQ---------------LRKASSASAGHGLPGNLRFVEV-PD
AtUGT83A1     42 -----ITFINT---EFNHNR--------------IISSLPNSPHEDYVGDQINLVSI-PD
W1NWE7        24 -----QVLLGP---NIGKRN----------------------------------------
W1NXD5        34 -----VTFVNT---DFTQKR----------------VLDASMKAHTESHPNKGTGCIRLV
AtUGT75B1     35 -----VTFVTC---VSVFHN--------------------SMIANHNKVENLSFLTF-SD
AtUGT84A1     42 -----VTFVTT---ELWGKK-----------------MRQANKIVDGELKPVGSGSIRFE
W1PLB6        39 -----VSFATN---EAIGKQ----------------LHDAGTTTTSGTDAHVEFLTF-FD
U5CXN6        40 -----VSLAAI---EAIAQQ----------------MHDAGGITTSGSDGHISFETF-SD
U5D2S8        32 -----VSLATN---KTVGQQ-------------------MHDASAAGPVGPISFETF-SD
U5D817        40 -----VSLATN---EAIGQQ-------------------MRDSGATGSDEHINFETF-SD
W1NFL7        40 -----VSLATN---EAIGQQ-------------------MRDSGAAGSDEHISFETF-SD
W1NFN9        40 -----VSLATN---EAIGQQ-------------------MRDSGAAGSDEHISFETF-SD
U5DBW3        38 -----ITVATA---AIGRQR---------------ILKDGDKATAFEADGNIKFEFF-SD
W1PE57        38 -----TTLATT---SIGQEG----------------ILKGGTRAVLEAEGTIKFEFY-SD
U5D3G1        33 -----VTFATT---AHGLSR------------------------IHQAIDIVSFASY-SD
AtUGT74B1     40 -----VTIATT---TYTASS--------------------------ITTPSLSVEPI-SD
U5DFQ6        40 -----VTFVTS---TFVTQS----------------------LRNTSSAKPLLLQSIRFE
U5DI88        71 -----VTLVTT---IQNLKD---------------------MRKANPSDPDFVSIELISD
U5DCN3        39 -----VTLVIT---IQNLQE---------------------MRASNPSDPAFLDIEHISD
U5D531        39 -----ATLVVA---IQNRGD---------------------IPKANPPADASASLEYISD
U5CUG8        39 -----ATLVVP---IQNRGD---------------------IGKADPRAVAAACLEYISD
W1PLP3        39 -----ATLVVP---IQNRGD---------------------IGKADPPAVAAAFLEYISD
U5DI92        39 -----ATIVAP---IRSRGD---------------------FRKADPSALASVNLEFFSD
U5D3V6        37 -----ATIVAP---TRCKDE---------------------FCKADPSAVASTNLEFFSD
U5DDJ3        39 -----VTFVTP---IQSKKY---------------------FHKADPAVLASTHLEFFSD
U5DFQ3        39 -----ATIVAP---IRNRSD---------------------FHKADPSALATTHLEFFSD
U5DI97        39 -----ATLVAP---VRSRDD---------------------IRRADRSALTSTNTEYISD
U5DCP2        39 -----ASLVVP---LRIHGD--------------------ILHKVDTSSLATIPLEYISD
U5DDK2        39 -----ASLVVP---LRIHGD--------------------ILHKVDTSTLATTHLEYISD
OsUGT709A3    38 -----VTFLHT---DYNLRR------------------LGAAAAAAVASPWLRFMSV-TD
SgUGT720A1    39 -----VTFLNT---DYNHRR------------------LHNLHLLAARFPSLHFESI-SD
U5CX27        11 -----IRNLDL---DSDSDR----------------------------------------
U5D008        10 -----RSFWPQ---NRLNSK------------------------VFDPNPTQRIKNF---
W1PMY7         6 ------------------------------------------------------------
AtUGT85A1     42 -----VTFVNT---VYNHNR---------------------FLRSRGSNALDGLPSFRFE
U5CVP6        39 -----ITYVNT---EFNHKR---------------------LLKSRGPDLSNGVPDFRFE
U5CNJ5        39 -----ITFVNT---EYNHQR---------------------LLKSRGSDPSMGVDGFVFD
U5CNG3        39 -----ITFVNN---EYNHKR---------------------LLKSRGPDPSMGVQGFAFE
U5CXJ9        39 -----ITFVNN---EYNHKR---------------------LLKSRGSDPSMGVQGFVFE
AtUGT76B1     38 -----ITVIHT---EFNSPN-------------------------SSNFPHFTFVSIPDS
W1PV81        40 -----ITIAHT---HFNSPK-------------------------IESPLKFTLIPI-DD
W1PVY0        42 -----ITIVHT---DYNAPH-------------------------LEPRPGLTIEPI-YL
W1PX56        75 -----ITIVHT---DYNAPH-------------------------LEPSPGFSIESI-CL
AtUGT90A1     43 -----VTVFTT---PKNQPF---------------------ISDFLSDTPEIKVISLPFP
OsUGT97A1     61 -----VTLVTT---PANLAF---------------------ARRRLPPSPSVRVVAIPFP
W1PH20        41 -----STIITT---PLNAIS--------------------MRRTLDCQNPKIRLIELPFP
W1PBB6        60 -----LTIFST---PSNTPF---------------------YRQCLPQDPKIRIIDLPFP
W1PHN8        60 -----LTIFST---PSNSPF---------------------YRQSLPKDSGIRIIDLPFP
W1PRF4        40 -----VTILVP---TSLVSV------------------PESRTLDALFSSGLDIHRL-PY
AtUGT71B1     35 -----VTLIVI---PSRVSD------------------DASSSVYTNSEDRLRYILLPAR
W1P1M0        37 -----VTFINVSTGSSPMQD------------------SILAGLEQTCKGFVKSVELPMM
AtUGT72B1     38 -----VTFVIAGEGPPSKAQ---------------------RTVLDSLPSSISSVFLPPV
W1PMA9        40 -----ITFINLITDSSAAQS------------------SVLSTLNDVGLSSLQSVDLPPA
W1PP20        38 -----ITFINL---TTDSSA---------------AQSSVLSNLNDVGLSSVQFVDLPPA
AtUGT88A1     38 -----IILVPP---PYQPES-----------------TATYISSVSSSFPSITFHHLPAV
U5DB27        36 -----ITILTT-------------------------------QSPFGPGAAATYIAN---
U5DBY1        36 -----ITMLTIES-PFGAAT----------------AAAYMAKVSASAPPALQFLPLPPP
U5DBY4        39 -----ITILTPHDHPFNPSS--------------TAPYIERTSSSSFPLNRLRFLQLPRV
U5D276        39 -----ITILTPHDHPFDAPT---------------TAPYIARISASYSPTRLRFLNLPPV
U5DB32        39 -----ITILTPHDHPFDAAT---------------TDPYISRISSSYSPTRLRFLKLPPV
W1NHX5        38 -----ILTVNP---PFYVGN-----------------TAPYIAYISNEFPDIAFLELSPP
W1NVU7        43 -----ILNVNP---PFDVGN-----------------TAPYIGRISSEFPAIAFLELEPP
W1PAP0        44 -----VLTFNP---PFHTGA-----------------TAPYMSRIAAEYPAISFHEL---
W1PD80        33 -----ILITTP---PFETGA-----------------TAPYMSRVASEIQAITFIELPPV
W1PZW0        37 -----VLITKP---PFDTGA-----------------TAEYVARVASQVSAITFHELPPA
AtUGT82A1     37 -----PVVMTP---ESIHRR----------------------ISATNEDLGITFLALSD-
W1NUV1       147 -----ITFVNT---EAVHAQ-----------TVKAYNNSLSKTLNDSIDCSNSTDDIFFG
AtUGT86A1     39 -----ITFVNTDSIHHHIST----------------AHQDDAGDIFSAARSSGQHDIRYT
AtUGT87A1     44 -----VTFVVT---EEWLGF----------------------IGSDPKPNRIHFATL-PN
W1P188        49 -----IRIVVT---EEWLSF---------------------MTSAQEPPPNVRLLSIPN-
W1P188         1 ------------------------------------------------------------
```

```
U5CYW0        57 ------------------------------------LMS-DTGGGHRASAEAIRD----AFY
W1PAZ2       107 ECEFQCGESHGLRLLGAETPKKVL-----------ILMSDTGGGHRASAEAIKA----AFN
AtUGT81A1    114 ENGIRENGTGGVLGEE-GLPLNGVEADRPKKVLILMS-DTGGGHRASAEAIRA----AFN
W1PTT2        90 GCGH------------SVHGQANAP--------HGI-VLDMSSMRGSMAVIP----RVA
U5D3V5        91 GLSSVS----------GLTVAARGNGHCTNGQAQAH-RGVVLEMRGIKGIEV----SLS
W1NUG7        87 GQAQAPG---------GVVIEMEALKGCLFNVGVLE-NESCSFSSSYSVERE----SVE
W1PC48        81 LGPSKLTIAAR------GHAHSLR-----------GQA-QAPHGIVVNMESLAD----PKK
W1PP63        75 TVAARGA---------GHSIHGQ-----------AQA-PNGIVIEMDSLPLSL----KIH
OsUGT701A2    92 HSLH------------GQAQARD---------------GIVVETRALPRDVHV----VSA
W1PA48        82 SGLE------------DFSFSRC-----------KEA-IKQHHREECLSAFEN----IFG
U5D0T8        75 QIVMLIV---------GTQGDVQPFVAIGKR--LQV-YGHRVRLATHSNFEE----FVL
AtUGT80A2    194 QIVMLIV---------GTRGDVQPFVAIAKR--LQD-YGHRVRLATHANFKE----FVL
W1PJ19        54 ----------------FHPPTFT-------------------------------------LLS
W1NU70        71 HVP-------------DLPPEAATTSD------LPP-HLQSLLKSSLDLLQP----LLH
W1NV64        72 SIP-------------ELPEAAA---TTSD---LPP-HLQDHLKAAFDALQP----LFH
W1PKS4        70 SIP-------------ELPEAATTYD-------LLP-HLQTPFKAAFDALQP----LFH
AtUGT91A1     77 VGDN------------KLPEDGEATTD------VPF-ELIPYLKIAYDGLKV----PVT
U5CMK2        68 PTH-------------GLPENAEATMD------LPI-EKLQFLTAAYDKLHE----PIE
U5CWM7        57 LVE-------------GLPKNAKATIEN-----ISL-EKVQYLKKAYDGLRA----PFE
U5D1X9        68 SVE-------------GLPKNAEATTD------IPL-KKVPYLKKAYDGLRA----PFE
W1PT00        69 QVA-------------GLSPHHQTTKD------LPP-HLMPHLKRAFHLSQN----QFK
OsUGT94A1     73 ELP-------------GLPPALHTTKH------LPP-RLMPALKRACDLAAP----AFG
AtUGT79B1     73 QVK-------------GLPPGAE---TNSD---VPF-FLTHLLAVAMDQTRP----EVE
W1PQK6        78 HID-------------GLPPGLECTTD------ATF-LQAELLKDALDATQP----QVV
U5D602        81 HLTPGP----------RLRPQLPSA--------PPV-YLGFHAVPSRPPGRP--C-PLA
U5D5X9        55 EFP-------------TLPPDPE----------SSN-KFPAHLQPVFDAIEH----LLA
U5D5Z2       106 SFP-------------ALLPDPESSNK------FPG-HLQPLFDATEHLLSP----LTT
U5D5X3        40 ELPN------------KFPG---------------HLQPVFDATEQLLAP----LTT
ZmUGT93B1     75 PYDS------------PAPDLAAPSP-------FPN-HLMPMFEAFAAAARA----PLA
PoUGT95A1     90 EAADEP----------GSGTEVR----------SSG-PRGNPLQDQNLQMGK----GIK
U5D0B7        60 SPP-------------PPPPQW-----------PEN-SPSPSFPAMLDPLES--I-LLE
AtUGT89A1     81 HHPLIPS---------GVENVKD----------LGG-YGNPLIMASLRQLRE----PIV
OsUGT96A1     76 ----------------HLPDASL----------PEN-SNHALLAVHLSGIRA----PLL
AtUGT92A1     77 SSDH------------GLPHDGENFDS------LPY-SLVISLLEASRSLREPFRDFMT
U5CRD3        38 GPNH------------GLPPNLEITDS------LPY-SLFPAFFKTTETLLPSFQNLIS
W1P7W1        82 STDH------------GLPDGVESSDA------VPP-ALTLHVFHASESLQP----AFE
U5D7I9        68 SADH------------GLPPDAENTDA------LPY-PLILRLFEASETLEPAFESLIS
W1PFP0        68 SADH------------GLPPDAENTDA------LPY-PLILRLCEASETLEPAFESLIS
OsUGT99A1     69 HAGA------------GLPEGVDNMDN------VPS-EFMARYFAAVARLRE----PVE
AtUGT73B1     79 CTEL------------GLPDGCENTDFIFSTPDLNVGDLSQKFLLAMKYFEE----PLE
OsUGT98B1     82 PFPAAEF---------GLPDGCE---NVDML--PSK-DLFSNFLLACGALRE----PFA
W1PF90        95 GTEV------------DLPRGCESLDAL-----PQL-SLFPEFMKATRLMKP----KLD
W1NIK4        76 CMEA------------GLPEGCENLDSL-----PST-HLLMNFFEATKMLRD----PFE
W1PQX3        79 CAEV------------GLPKGCESFEDV-----PSY-DLMSNFLRATEMLRE----PFE
W1PPW8        78 CAEV------------GLPEGCESVDLL-----PSN-DLMINFFRATELLQE----PFE
W1PS80        78 CAEV------------GLPEGCESMDLL-----PSN-DLKINFLRATELLQE----PFE
AtUGT90A1     74 ENIT------------GIPPGVENTEKL-----PSM-SLFVPFTRATKLLQP----FFE
OsUGT97A1     92 AHP-------------QIPPGVESTDAL-----PSQ-SLFPAFLRATALLRE----PFA
W1PH20        73 EEP-------------AVPRNCENTDQ------LPSVDLFVPFLKATQKLRP----QFD
W1PBB6        91 SYP-------------DLPKGCESTEQL-----PSM-DMFPTFLNAVKELRD----SFE
W1PHN8        91 SHP-------------DLPKGCESTEQL-----PSM-DMFLPFLNAVKELRD----SFE
W1PRF4        73 SLPP------------PLSDNID----------PFF-KQWGLISRSIDELKP----ILE
AtUGT71B1     69 D---------------QTTDLVS----------YID-SQKPQVRAVVSKVAG----DVS
W1P1M0        74 DMS-------------DIPPDTG----------KVV-QISLTIRRSLGSLRG----LLG
AtUGT72B1     72 DLT-------------DLSSSTR----------IES-RISLTVTRSNPELRK----VFD
W1PMA9        77 DLS-------------DLPISAK----------IET-RISLTVRRSLSSLRT----LLC
W1PP20        75 DLS-------------DLPSSAK----------IET-RISLTMFRSLSSLRA----LLG
AtUGT88A1     73 T---------------PYSSSST----------SRH-HHESLLLEILCFSNP----SVH
U5DB27        57 ---------------------------------ISALFIGAHKQSIAEALKS-----LS
U5DBY1        74 LNF-------------SVSPETR----------GET-LISLFIGAHKQSVAE----ALK
U5DBY4        80 DF--------------SPAPDMR-----------PQ-TLFSLFAAAHKPIVT----ETL
U5D276        79 HF--------------SPTPDMR----------SQT-IRSLFVAAHKPLVAE----ALK
U5DB32        79 NFS---------------PTSDM----------RPQ-TIRSLFAAAHKPFVT----EAL
W1NHX5        73 EF--------------SVDHSLN----------LET-LVFEFIHDSNPSVSK----ALE
W1NVU7        78 EF--------------SVDDSAH----------LET-LIFEYIHLANNPRVS----EAL
W1PAP0        76 ----------------SLPPNQC-------------A-TFMDWFLVLKPLVRE----ALE
W1PD80        68 EL--------------SVTQIDH----------SLT-FMFEWLLLCKPAVAQ----ALQ
W1PZW0        72 DLN-------------VLNPN------------LEL-LIFEWLSLCKPHVSE----ALL
AtUGT82A1     66 ----------------GQDRPDA----------PPS-DFFSIENSMENIMPP----QLE
W1NUV1       188 AREAGLDIRYRVVSD--GLPLEFDRSLN------HNQ-FMFSLLHVFSAHVEK----IIR
AtUGT86A1     78 TVSD------------GFPLDFDRSLN------HDQ-FFEGILHVFSAHVDD----LIA
AtUGT87A1     73 IIPS------------ELVRAND----------FIA-FIDAVLTRLEEPFEQ----LLD
W1P188        79 ----------------VIPSELS----------RAA-DWVGFVQAVNSTMEE----PVD
W1P188         1 -----------------------------------------------------------
```

```
W1P302       79 -----------------VIPSELN----------RSA-DWTGFIEAVSTKMEE----PFE
AtUGT78D1    73 GVPE-------------GTMLGN-----------PLE-MVELFLEAAPRIFRS----EIA
W1PTN8       76 EN---------------YSPENLT----------PMA-AIEAFLRGAPSAVRE----ALG
ZmUGT77A1    82 GAPAAEE----------TVPVPRQ----------MQL-FMEAAEAGGVKAWLE----AAR
AtUGT83A1    79 GLED-------------SPEERNI----------PGK-LSESVLRFMPKKVEE----LIE
W1NWE7       36 -----------------RMMLRDD---------------------------------KIS
W1NXD5       70 SIPD-------------GLEPDADRNK-------LGA-LCEAMLSSMPNALET----LIK
AtUGT75B1    66 GFDDG------------GISTYED----------RQK-RSVNLKVNGDKALSD----FIE
AtUGT84A1    77 FFDE-------------EWAEDDDRRAD------FSL-YIAHLESVGIREVSK----LVR
W1PLB6       74 GLA--------------VDEDRRD----------LDW-FMAHLNRAGPNGLAA----LIS
U5CXN6       75 GH---------------GIDDDRN-----------DA-FMAQLSTAGPGGLLA----LID
U5D2S8       64 GL---------------GMPDNNQHD--------LDK-FMAQLNCAGPEGLAA----LIN
U5D817       72 GLSV--------------DGNQHD----------VDK-FMAQLNRAGPEGLAT----LIN
W1NFL7       72 GLSV-------------VGNQLD-----------ADK-FMAQLNRAGPEGLAT----LIN
W1NFN9       72 GLSV--------------DSNQHD----------IDK-FMAQLNRAGPEGLAT----LIN
U5DBW3       74 GSD--------------PLMPIKD----------LDD-CLGRLEREGPESLAG----LVE
W1PE57       73 GWDS-------------RVPIVH-----------LDD-YMDKLEIVGSESLAS----LVE
U5D3G1       60 GFDQ-------------GFDNSIA----------FAD-YVSQFKRLGSETLAA----LIR
AtUGT74B1    65 GFDFIPI----------GIPGFS-----------VDT-YSESFKLNGSETLTL----LIE
U5DFQ6       70 TISD-------------GFDNGRSRKDS------IAF-YLEKFKITGQVALTE----LIA
U5DI88      102 GFDD-------------GLPSEFT----------VDA-YLATLERVGSLTLDS----LLA
U5DCN3       70 GFDD-------------GLPVDFK----------LDT-YVDSLKRVGSQTLDS----LLT
U5D531       70 GYDD-------------ALPSNFD----------VEI-YKTTLQRVGSEKLLE----LIA
U5CUG8       70 GYDD-------------GLPSNFD----------VEI-YRTTFQCVGSKKLLE----LIA
W1PLP3       70 GYDD-------------GLPSNFD----------VEI-YRTTFQRVGSKKLLE----LIA
U5DI92       70 GHDN-------------GFPLDLS----------LKA-QDSNFSRVGSRAVGD----LID
U5D3V6       68 GHDS-------------GLPPDHD----------REA-YYSNRRSVGSRAVGE----FID
U5DDJ3       70 GHDD-------------GLPPDLD----------LEA-YQSNLRRLGSRAVGD----LID
U5DFQ3       70 GHDD-------------GLPPDFT----------FET-YEPNLRRTGSRAVGD----LID
U5DI97       70 GHDD-------------GLPSDYS----------SEA-YWATLRRVGSQTLGD----LIA
U5DCP2       71 GYDD-------------GVPLTFD----------PGA-YDISLQRVGSQTLGE----LIA
U5DDK2       71 GCDD-------------GLPLTFD----------PEA-YDITLQRVGSQTLGE----LIG
OsUGT709A3   71 GLPD-------------DHPRTVAN---------LGE-ISRSLHTAGRAAYRA----LLA
SgUGT720A1   72 GLPP-------------DQPRDIL----------DPK-FFISICQVTKPLFRE----LLL
U5CX27       23 -----------------GDCPGRQ---------------------VGPNRLPP-----LE
U5D008       35 -----------------DPNPNSD---------------------------------RVH
W1PMY7        6 -----------------------------------------------TTPFKE----LLV
AtUGT85A1    73 SIAD-------------GLPETDMDATQD-----ITA-LCESTMKNCLAPFRE----LLQ
U5CVP6       70 SIPD-------------GMPETDLDATQD-----VPE-LSDHIRTCFLAPFRE----LVL
U5CNJ5       70 CIPD-------------GLPPVEIEATQS-----VPD-LCYYTRFSCVAPFRE----LLA
U5CNG3       70 SIPD-------------GLPPIEIEATQS-----IPE-LCYYTRNCCLAPFRE----LVA
U5CXJ9       70 SIPD-------------GVPPIEIEATQS-----IPE-LCYYTRFSCLDPFRE----LVA
AtUGT76B1    65 LSEPE------------SYPDVIE--------------ILHDLNSKCVAPFGD----CLK
W1PV81       66 GLPVNW-----------QPKGGAD----------LLK-LVDTISSNCRAPLFS----LLS
W1PVY0       68 GLSN-------------EEAREMD----------IVE-LILTLNKKCELPFRA----LIE
W1PX56      101 GLSN-------------QEAREMD----------KVE-LVLVLNEKCRPPFRA----LME
AtUGT90A1    74 ENIT-------------GIPPGVENTEKL-----PSM-SLFVPFTRATKLLQP----FFE
OsUGT97A1    92 AHP--------------QIPPGVESTDAL-----PSQ-SLFPAFLRATALLRE----PFA
W1PH20       73 EEP--------------AVPRNCENTDQ------LPSVDLFVPFLKATQKLRP----QFD
W1PBB6       91 SYP--------------DLPKGCESTEQL-----PSM-DMFPTFLNAVKELRD----SFE
W1PHN8       91 SHP--------------DLPKGCESTEQL-----PSM-DMFLPFLNAVKELRD----SFE
W1PRF4       73 SLPP-------------PLSDNID----------PFF-KQWGLISRSIDELKP----ILE
AtUGT71B1    69 D----------------QTTDLVS----------YID-SQKPQVRAVVSKVAG----DVS
W1P1M0       74 DMS--------------DIPPDTG----------KVV-QISLTIRRSLGSLRG----LLG
AtUGT72B1    72 DLT--------------DLSSSTR----------IES-RISLTVTRSNPELRK----VFD
W1PMA9       77 DLS--------------DLPISAK----------IET-RISLTVRRSLSSLRT----LLC
W1PP20       75 DLS--------------DLPSSAK----------IET-RISLTMFRSLSSLRA----LLG
AtUGT88A1    73 T----------------PYSSSST----------SRH-HHESLLLEILCFSNP----SVH
U5DB27       57 ----------------------------------ISALFIGAHKQSIAEALKS-----LS
U5DBY1       74 LNF--------------SVSPETR----------GET-LISLFIGAHKQSVAE----ALK
U5DBY4       80 DF---------------SPAPDMR-----------PQ-TLFSLFAAAHKPIVT----ETL
U5D276       79 HF---------------SPTPDMR----------SQT-IRSLFVAAHKPLVAE----ALK
U5DB32       79 NFS----------------PTSDM----------RPQ-TIRSLFAAAHKPFVT----EAL
W1NHX5       73 EF---------------SVDHSLN----------LET-LVFEFIHDSNPSVSK----ALE
W1NVU7       78 EF---------------SVDDSAH----------LET-LIFEYIHLANNPRVS----EAL
W1PAP0       76 -----------------SLPPNQC------------A-TFMDWFLVLKPLVRE----ALE
W1PD80       68 EL---------------SVTQIDH----------SLT-FMFEWLLLCKPAVAQ----ALQ
W1PZW0       72 DLN--------------VLNPN------------LEL-LIFEWLSLCKPHVSE----ALL
AtUGT82A1    66 -----------------GQDRPDA----------PPS-DFFSIENSMENIMPP----QLE
W1NUV1      188 AREAGLDIRYRVVSD--GLPLEFDRSLN------HNQ-FMFSLLHVFSAHVEK----IIR
AtUGT86A1    78 TVSD-------------GFPLDFDRSLN------HDQ-FFEGILHVFSAHVDD----LIA
AtUGT87A1    73 IIPS-------------ELVRAND----------FIA-FIDAVLTRLEEPFEQ----LLD
W1P188       79 -----------------VIPSELS----------RAA-DWVGFVQAVNSTMEE----PVD
W1P188        1 ------------------------------------------------------------
```

```
U5CYW0        78 H--------EYGDD-YRVLVKDVWKE-HTGWPLNDMERSYKFMVKHVQLWKVAFHGTSPS
W1PAZ2       153 L--------EFGDEYQVYVTDLWSD--HTPWPFNQLPRTYNFLVKHGTLWKMTFYGTAPR
AtUGT81A1    168 Q--------EFGDEYQVFITDLWTD--HTPWPFNQLPRSYNFLVKHGTLW----------
W1PTT2       124 N-----TDPEQAGSTGSSYFCVDVKG-GEVWE-DVLRFCLQFGMAPRSWT------DFLS
U5D3V5       135 R----------------DNPYVEAGAGELWI-DVLKATLKLGFAPVSWT------DYLS
W1NUG7       132 R---------ESGGSLYYADVWGGEL----WI-DVLHETLKHGLAPRSWT------DYLY
W1PC48       120 R---------RVHEGGKSPYVEVSGGELWIDV---LRQTLKCGLAPRSWT------DYLH
W1PP63       110 R----------KGKRD-EGYSYADVSGGALWI-EVLRESLKHGLAPRSWT------DYLY
OsUGT701A2   121 R--------AHGGDDDATVRAYADVGAGALWV-EVLEECLKLGLAPPSWT------DYLY
W1PA48       114 E-DSSIEGDFVAINFFALVVSLYYQFVYFTTKLFDAKSHVKEGWHLAELF------RVRC
U5D0T8       118 T--AGLEFYPLGGDPKVLAGYMVKNKGFLPSG--ASEIPIQRKQIKEIIN------SLLP
AtUGT80A2    237 T--AGLEFYPLGGDPKVLAGYMVKNKGFLPSG--PSEIPIQRNQMKDIIY------SLLP
W1PJ19        64 ------------------PNVVVHDF-NLYWL---PKTAAEFGVPSVFFS------IFTA
W1NU70       106 S--------ILSKL-P--LNLVIQDF-APYWL---PKIAAKFGVPTAYFS------IFNA
W1NV64       107 T--------LLPKL-S--PNLVIHDF-TSYWL---PEIATTFGIPTVFFS------IFNA
W1PKS4       104 T--------LLPKLS---PNLIIHDF-TSYWL---PQIATTFGIPTVFFS------IFNA
AtUGT91A1    113 E--------FLESSK---PDWVLQDF-AGFWL---PPISRRLGIKTGFFS------AFNG
U5CMK2       103 N--------FVSEES---PDWLIHDL-FAPWA---PCLAAKFGIPSCFCS------VFTA
U5CWM7        93 E--------LVAKVS---PDWILVDF-APYWA---PAIAFKYGIPCCFFI------VFSA
U5D1X9       103 E-------LVAKVS----PDWIVVDF-APYWA---TSIASKYGVPCCWF-------NAVS
W1PT00       104 D--------LLRSLK---PNFLLHDF-IQPWA---PEAALELKIPSSVFL------TFSV
OsUGT94A1    108 A--------LLDELS---PDVVLYDF-IQPWA---PLEAAARGVPAVHFS------TCSA
AtUGT79B1    108 T--------IFRTI-K--PDLVFYDS--AHWI---PEIAKPIGAKTVCFN------IVSA
W1PQK6       113 S--------ILRDLA---PDLVVYDF-SYCWL---PTVAHKLGIRAVYFS------VFSA
U5D602       118 R--------SLARNYG--RVAVVHDP-LVSIA-----AAAASRTPNAEAY------GYRC
U5D5X9        86 P--------LTVLVRDLASVVVIHDM-GRSVA---AGAAAESPNSEAYMY------RCPS
U5D5Z2       141 L--------VHELASIHRRVAVIHDALMSVAA---GAAVECLNAEAYSYN------CGST
U5D5X3        66 L--------VCEPAGSHRRVVVVHDMLMSVAA---RAAAESLNSESYSYR------CSSA
ZmUGT93B1    110 A-------LLQRLSTSYRRVAVVFDR-LNPFA-----ATEAARLANADAF------GLQC
PoUGT95A1    124 S--------FLSARSGTRPTCVVIDV-MMSWS---KEIFVDHEIPVVSFS------TSGA
U5D0B7        91 R--------VAAGKSL--PTVVIVDT-MMSRL---LETCRKFKIPAVSFF------TSGA
AtUGT89A1    116 N-------WLSSHPNP--PVALISDF-FLGWT-------KDLGIPRFAFF------SSGA
OsUGT96A1    104 S--------WARSRPDDPPTVVVSDF-FLGWA---QLLADDLGVPRVVFY------ASGA
AtUGT92A1    117 K---------ILKEEGQSSVIVIGDF-FLGWI---GKVCKEVGVYSVIFS------ASGA
U5CRD3        78 K---------ISKQDNLPPLCLVSDI-FLGWT---VEVANNLNIFHTVLC------TSGA
W1P7W1       118 R--------LISEGPS--PVCVIGDI-FTGWT---IGVAEKLGVKHVTFL------KSSA
U5D7I9       108 D---------VARKDGRLPLCIVADV-FCGWT---VLVAERLGIHHVVFT------TCGA
W1PFP0       108 D---------IARKDGRLPLCIVADV-FCGWT---VLVAERLGIHHVVFT------TCGA
OsUGT99A1    105 R----HLLLRADEGGAPPPTCVVADF-CHPWA---SELAAGLAVPRLTFF--------SM
AtUGT73B1    122 E--------LLVTMR---PDCLVGNM-FFPWS---TKVAEKFGVPRLVFH------GTGY
OsUGT98B1    122 A--------RLRQQ-RPPASCIISDM-IHSWA---GDIARELGVPWLTFN--------GS
W1PF90       132 E-------ILAIDR----PLAIVSDF-FLPWT---LDTAERIGVPRLVFQ------GISC
W1NIK4       113 K--------LIRQTKN--PLCIISDT-FLPWT---AEAAQALGIPRLAFH------TSNC
W1PQX3       116 K--------LIRQE-VENPLCIISDT-FFWWT---VETGKVFGIPRLVFH------GTSC
W1PPW8       115 K---------LIRQEVENPLCIISDM-FLGWT---VETAKVLGIPRIVFH------GTSC
W1PS80       115 K--------LIRQE-VENPLCIISDM-FLGWT---VETAKVLGIPRIVFH------GTSC
AtUGT90A1    111 E--------TLKTLPK--VSFMVSDG-FLWWT---SESAAKFNIPRFVSY------GMNS
OsUGT97A1    128 E--------FLASLPSPPPLVLVSDF-FLGFT---QRVADDAGVRRLTFN------GMSP
W1PH20       109 Q-------ILEELTVIDPPICIVSDF-FHGWT---LDSAKRLGIPRLVFH------GMGA
W1PBB6       127 Q-------SLSEIAAVNPPICLISDT-FLYWT---QYVAEKLSIPRLTFM------GMSA
W1PHN8       127 Q-------SLSEIAAVNPPICLISDT-FLYWT---QYVAEKLSIPRLTFM------GTSA
W1PRF4       105 N--------------LNGLSALITDISLAGAM---LPLTNQLGIPNYIFF------TSSA
AtUGT71B1     98 T--------RSDSR----LAGIVVDM-FCTSM---IDIADEFNLSAYIFY------TSNA
W1P1M0       105 D---LFSGLPTATGRFPAISGLIADP-FCADV---IELTHELQLPTYLFF------TSTA
AtUGT72B1    103 S--------FVEGGRL--PTALVVDL-FGTDA---FDVAVEFHVPPYIFY------PTTA
W1PMA9       108 D---------LTSKFP--NTVMVADI-FGTDA---FDVADELSLPKYLFF------PANA
W1PP20       106 D---------LTSKFP--NTVLVADI-FGTDA---FDVADELSLPKYLFF------PSNA
AtUGT88A1    102 R-------TLFSLSRNFNVRAMIIDF-FCTAV---LDITADFTFPVYFFY------TSGA
U5DB27        78 P-----------------VRAFITDL-FCTTI---LDTAAEAGVPAYNFF------TSGA
U5DBY1       105 S--------------LSPVRAFIIDL-FCTTF---LETAAEAGVPAYIFF------TSGA
U5DBY4       109 K-------SLSSTSPP--VCAFIIDF-FCTAL---LDAAMESGVPAYIFF------TSSA
U5D276       109 S--------LSTPSSP--VRAFITDF-FCTNL---LEAAEEADVPCYLFF------TSGA
U5DB32       108 K--------SLSSP-SFPVHAFVTDF-FCTTL---LDAAKEADVPSYIFF------TSGA
W1NHX5       103 S---------ICEQSN--IVAFIMDL-FCMTA---LDVANDMHIPAHVFY------TSSA
W1NVU7       108 K--------SITEHST--VVAFVMDL-FCATA---LDRAKDMHIPTYLFF------TSSA
W1PAP0       102 S---------FSRRTP--VRAFIMDF-FCTSV---LEDAEELCIPAYFFF------TSAA
W1PD80        98 S---------ISEKTV--IRAFVMDS-FCSTA---LDVATDLGVPAYFFY------ASAA
W1PZW0       101 S---------ISEKAR--IKAFIMDF-FCCTA---LEVARGMGIASYIFF------TSGA
AtUGT82A1     94 R-------LLLEEDLD--VACVVVDL-LASWA---IGVADRCGVPVAGFW------PVMF
W1NUV1       235 E--------VALDGPP--ISCIIADT-FFVWV---APMAEKFGVPHISFW------TEPA
AtUGT86A1    114 K---------LSRRDDPPVTCLIADT-FYVWS---SMICDKHNLVNVSFW------TEPA
AtUGT87A1    105 R---------LNSP----PTAIIADT-YIIWA---VRVGTKRNIPVASFW------TTSA
W1P188       107 R---------LIDSIEPMVSVIVADV-YMPWV---ADMAKRRGIPVTWLF------PMAA
W1P188         1 ------------------------------------------------------------
```

```
W1P302      107 R---LLVGLLETEERP--VSVVIVDV-FMPWA---ADVAERRGIPVAVLF------PMAS
AtUGT78D1   104 A-----AEIEVGKK----VTCMLTDA-FFWFA---ADIAAELNATWVAFW------AGGA
W1PTN8      106 Q-----------SEPP--VSCVIGDS-FLVFA---EEVAREIGAKWVSFW------TAGP
ZmUGT77A1   117 A--------AAGGA-R--VTCVVGDA-FVWPA---ADAAASAGAPWVPVW------TAAS
AtUGT83A1   111 R-------MMAETSGGTIISCVVADQ-SLGWA---IEVAAKFGIRRTAFC------PAAA
W1NWE7       46 A-----------------IIGLLHNRSMIQLT----------------------------
W1NXD5      105 D---------INGLEGDSVTCIVADV-SMGWA---LDVAKKLGIPRAAFW------PAAA
AtUGT75B1    99 A--------TKNGDSP--VTCLIYTI-LLNWA---PKVARRFQLPSALLW------IQPA
AtUGT84A1   113 R----------YEEANEPVSCLINNP-FIPWV---CHVAEEFNIPCAVLW------VQSC
W1PLB6      105 D--------LEARGRR--VHCVVSNP-FVPWV---VDVAEGFSIPCALLW------VQSC
U5CXN6      104 D--------LRASGRP--VHCLVSNP-FVPWV---VDVAERFSIPCAMLW------VQSC
U5D2S8       96 D--------LQARGRP--VHYVVSNP-FMPFV---VDVAEGFGIPCAMLW------IQSC
U5D817      103 D--------LQVRGRP--VHCIVSNP-FVPWV---VDVAEGFGIPCAMLW------VQSC
W1NFL7      103 D--------LQVRGRP--VHCIVSNP-FVPWV---VDVAEGFGIPCAMLW------VQSC
W1NFN9      103 D--------LQVRGRP--VHCIVSNP-FVPWV---VDVAEGFGIPCAMLW------VQSC
U5DBW3      105 D--------LRVQGRK--VTYMVTTP-FTPWV---IDLAKGLGVPCSLLW------IQCC
W1PE57      104 E--------LAANGRR--VSYMISSP-FTPWV---VDVAKKLEIPCSVLW------IQSS
U5D3G1       92 N--------LASQGCP--VTFIIYNS-VVPWS---ADVADELGIPSALHW------IQSA
AtUGT74B1    99 K--------FKSTDSP--IDCLIYDS-FLPWG---LEVARSMELSAASFF------TNNL
U5DFQ6      106 R--------LVAEGYP--VNCIVYDP-LLPWI---ADVSENLEIPSAAFF------TQSC
U5DI88      134 R--------LSVEGSR--PLCIIYDS-FLPWV---LDVAHAHGILGATYH------TQSC
U5DCN3      102 R--------LGAEGVR--PLCVIYDS-FLPWA---LDVAHAHGILGAAFH------TQSI
U5D531      102 R--------LTVEGMR--PVCLIYDL-ALSWV---LEIAHAHGLLGATFI------TQPV
U5CUG8      102 R--------LTVEGPR--PVCLIYDS-FLSWA---LELAHAHGLLGATFI------TQPL
W1PLP3      102 R--------LTVEGPR--PVCLIYDS-FLSWA---LELAHAHGLLGATFI------TQPL
U5DI92      102 R--------LTLEGAR--PVCLIYNS-FLPWA---LDVAHHHGILGVTFL------TQPC
U5D3V6      100 R--------LTLEGER--PVSLIYDS-FLPWA---LDVAHDHGMLGVSFL------TQSC
U5DDJ3      102 R--------LTVQGVR--PVSLVYDS-FYLWA---LDVAHDHGILGVSFL------TQSC
U5DFQ3      102 R--------LTVEGER--PVCLVYDS-FLTWA---LDVAHDHGILGVSFL------TQSC
U5DI97      102 R--------LNEEGPR--PVCLIYDS-FLTWA---LDVAHAYGILGVTFL------TQSY
U5DCP2      103 R--------LAVDGLQ--PVCLIYDS-ILQWA---LDIAHAHGILGVAFL------TQSY
U5DDK2      103 R--------LSVDGQQ--PVCLIYDS-ILPWA---LDIAHAHGILGVTFL------TQSY
OsUGT709A3  104 SSSQLVPADAAGGGAFPPVTTVVADA-LLPFA---IDVAEELGVPALAFR------TASA
SgUGT720A1  104 S--YKRISSVQTGRPP--ITCVITDV-IFRFP---IDVAEELDIPVFSFC------TFSA
U5CX27       40 K-------------------THIVDVVVLANA-------------NENFM------TNGD
U5D008       45 -------------------------FPGWP----------YPPTRWP------PQEK
W1PMY7       15 R--------LNGSEGIPRVSYIVSAG-MMSFT---REVAEEIGIPRVSFW------TSGG
AtUGT85A1   110 R--------INAGDNVPPVSCIVSDG-CMSFT---LDVAEELGVPEVLFW------TTSG
U5CVP6      107 R--------LNGSGEVPKVTCFVIDG-VTNFA---QPVADELCVPKVIFW------TASA
U5CNJ5      107 R---------LTDSDVPPVSCVISDT-AMNFT---QQVAIGFGIRRIAFY------PASA
U5CNG3      107 R--------LNSTEDLPPVSCVISDG-AMNFT---QTVADELGIPRVTFW------TTSA
U5CXJ9      107 R--------LNSSEDLPPVSCVISDG-VMNFT---QTLADELGIPRVAFW------TTSA
AtUGT76B1    95 K--------LISEEPT--AACVIVDA-LWYFT---HDLTEKFNFPRIVLR------TVNL
W1PV81      100 R---------LNEEDP--VAGFFYDG-IMGFA---GEVGTELGLKAMAFR------TSSA
W1PVY0      100 D--------LQSKKGGLRVSYLITDA-VMYFT---QDLAEELGIKRMAFR------TSSS
W1PX56      133 D--------LQSRKGGVPVSCLITDA-VMYFT---QDLAEELGIKRMAFR------TSSS
AtUGT90A1   111 E--------TLKTLPK--VSFMVSDG-FLWWT---SESAAKFNIPRFVSY------GMNS
OsUGT97A1   128 E--------FLASLPSPPPLVLVSDF-FLGFT---QRVADDAGVRRLTFN------GMSP
W1PH20      109 Q-------ILEELTVIDPPICIVSDF-FHGWT---LDSAKRLGIPRLVFH------GMGA
W1PBB6      127 Q-------SLSEIAAVNPPICLISDT-FLYWT---QYVAEKLSIPRLTFM------GMSA
W1PHN8      127 Q-------SLSEIAAVNPPICLISDT-FLYWT---QYVAEKLSIPRLTFM------GTSA
W1PRF4      105 N--------------LNGLSALITDISLAGAM---LPLTNQLGIPNYIFF------TSSA
AtUGT71B1    98 T--------RSDSR----LAGIVVDM-FCTSM---IDIADEFNLSAYIFY------TSNA
W1P1M0      105 D---LFSGLPTATGRFPAISGLIADP-FCADV---IELTHELQLPTYLFF------TSTA
AtUGT72B1   103 S--------FVEGGRL--PTALVVDL-FGTDA---FDVAVEFHVPPYIFY------PTTA
W1PMA9      108 D---------LTSKFP--NTVMVADI-FGTDA---FDVADELSLPKYLFF------PANA
W1PP20      106 D---------LTSKFP--NTVLVADI-FGTDA---FDVADELSLPKYLFF------PSNA
AtUGT88A1   102 R-------TLFSLSRNFNVRAMIIDF-FCTAV---LDITADFTFPVYFFY------TSGA
U5DB27       78 P-----------------VRAFITDL-FCTTI---LDTAAEAGVPAYNFF------TSGA
U5DBY1      105 S--------------LSPVRAFIIDL-FCTTF---LETAAEAGVPAYIFF------TSGA
U5DBY4      109 K-------SLSSTSPP--VCAFIIDF-FCTAL---LDAAMESGVPAYIFF------TSSA
U5D276      109 S--------LSTPSSP--VRAFITDF-FCTNL---LEAAEEADVPCYLFF------TSGA
U5DB32      108 K--------SLSSP-SFPVHAFVTDF-FCTTL---LDAAKEADVPSYIFF------TSGA
W1NHX5      103 S---------ICEQSN--IVAFIMDL-FCMTA---LDVANDMHIPAHVFY------TSSA
W1NVU7      108 K--------SITEHST--VVAFVMDL-FCATA---LDRAKDMHIPTYLFF------TSSA
W1PAP0      102 S---------FSRRTP--VRAFIMDF-FCTSV---LEDAEELCIPAYFFF------TSAA
W1PD80       98 S---------ISEKTV--IRAFVMDS-FCSTA---LDVATDLGVPAYFFY------ASAA
W1PZW0      101 S---------ISEKAR--IKAFIMDF-FCCTA---LEVARGMGIASYIFF------TSGA
AtUGT82A1    94 R-------LLLEEDLD--VACVVVDL-LASWA---IGVADRCGVPVAGFW------PVMF
W1NUV1      235 E--------VALDGPP--ISCIIADT-FFVWV---APMAEKFGVPHISFW------TEPA
AtUGT86A1   114 K----------LSRRDDPPVTCLIADT-FYVWS---SMICDKHNLVNVSFW------TEPA
AtUGT87A1   105 R---------LNSP----PTAIIADT-YIIWA---VRVGTKRNIPVASFW------TTSA
W1P188      107 R---------LIDSIEPMVSVIVADV-YMPWV---ADMAKRRGIPVTWLF------PMAA
W1P188        1 ------------------------------------------------------------
```

```
U5CYW0       128 WV---HCVYLAALAA---------------------------YYAKKVEAGLMKYKPDII
W1PAZ2       203 LV---HQPHFAATST---------------------------FIAREVAKGLMKYQPD--
AtUGT81A1    208 -----KMTYYGTSPR---------------------------IVHQSNFAATSTFIAR--
W1PTT2       171 LS---VGGTLS--NG---------------------------GVSGQSFRYGPQVVNV--
U5D3V5       171 LS---IGGTLS--QA---------------------------GISGQAFRHGPQISTV--
W1NUG7       172 LT---VGGTLS--NA---------------------------GISGQAFNHGPQISNV--
W1PC48       162 LT---VGGTLS--NA---------------------------GISGQAFRYGPQISNV--
W1PP63       152 LS---VGGTLS--NA---------------------------GISGQTFKHGPQISNV--
OsUGT701A2   166 LT---VGGTLS--NG---------------------------GISGQTFKHGPQISNV--
W1PA48       167 VV---VAPYVV--PY---------------------------SMPSSFE---CRFKST--
U5D0T8       168 AC---REPYGSSGVP---------------------------FKADAIISNPPAYGHTHV
AtUGT80A2    287 AC---KEPDPDSGIS---------------------------FKADAIIANPPAYGHTHV
W1PJ19        96 SV---AAYFLSHTRK---------------------------EGNPHVPTAAEITTPP--
W1NU70       145 AS---VAYFMV--PS---------------------------RRVPETP---PPSASD--
W1NV64       146 AS---FAYAA---VP---------------------------YRVSGTP---TDLTSA--
W1PKS4       143 AS---MAYLMV--PY---------------------------RFTGKAF---PPTAAD--
AtUGT91A1    152 AT---LGILKP-------------------------------PGFEEYRTSPADFMKP--
U5CMK2       142 ST---LVFFGP--PD---------------------------GLVGAHQRLTPESYAK--
U5CWM7       132 AC---CAFGGA--PE---------------------------SLNGVNERKKVEDFTV--
U5D1X9       141 TA---FTVFLG--PP---------------------------HSLKGLCRTTTVDELM--
W1PT00       143 SC---SSFLYHLVLG---------------------------KKPNEFPFETMELHPH--
OsUGT94A1    147 AA---TAFFLH-------------------------------FLDGGGGGGGRGAFPF--
AtUGT79B1    146 AS---IALSLV--PS---------------------------AEREVIDGKEMSGEEL--
W1PQK6       152 ASDGYTMVCER-------------------------------RANGTFP---TSEELE--
U5D602       156 VS---ACGLVH--------------------------------LLGHGE-----------
U5D5X9       128 AC---FSLCFQSEGE---------------------------GRPEPIP-----------
U5D5Z2       184 CA---TICFQR-------------------------------EAEGQQE-----------
U5D5X3       109 CS---AICFRR-------------------------------EEDDHPE-----------
ZmUGT93B1    151 VA-----------IS---------------------------YNVGWLD-----------
PoUGT95A1    166 TA---SAVGYG--MW----------------------------KAEVGNMKPGEIREI--
U5D0B7       131 CA---AAMEHA--LWMNSPDKVTQGQVLTLNGLPGEMALSSYELKRFFFRPPHPPPNPTS
AtUGT89A1    153 FL---ASILHF--VS---------------------------DKPHLFE-----------
OsUGT96A1    146 FA---VAALEQ-------------------------------LWNGALP---LDPKIS--
AtUGT92A1    158 FG---LGCYRS--IW---------------------------LNLPHKETKQDQFLLD--
U5CRD3       119 YG---TAVYNS--VW---------------------------THMPHIYTE---------
W1P7W1       158 FG---DLAAGT--MW---------------------------HHLPHRK-------TD--
U5D7I9       149 FG---TLGYAS--TW---------------------------LHLPHVG-------TD--
W1PFP0       149 FG---TLGYAS--TW---------------------------LHLPHVG-------TD--
OsUGT99A1    149 CA---FCLLCQ-------------------------------HNVERFGAYDGVADDN--
AtUGT73B1    161 FS---LCASHCIRLP---------------------------KNVATSS-----------
OsUGT98B1    161 CT---FSSFAR--DI---------------------------IYRKNLL---ENLTDD--
W1PF90       171 SA---HCCLELVKKF---------------------------RLWDAVD-------SD--
W1NIK4       153 FS---QCCWHS--LY---------------------------VHQPQKR-----VGSD--
W1PQX3       157 FS---LCCLRC--IR---------------------------EHKPHNN-----VGSD--
W1PPW8       156 FS---CCCSSC--IH---------------------------EKKPHIN-----VGSD--
W1PS80       156 FS---CCCSSC--IH---------------------------EKKPHIN-----VGSD--
AtUGT90A1    151 YS---AAVSIS-------------------------------VFKHELFTEPESKSDT--
OsUGT97A1    170 FS---LALCFT--LA---------------------------SRRPHVGVEG--------
W1PH20       152 YS---MSICKS--VC---------------------------VHYPQKGLTE--------
W1PBB6       170 YA---TCLSMA--IA---------------------------TYGSPIP-------KD--
W1PHN8       170 YA---TCLSLA--IA---------------------------AYGSPIP-------KD--
W1PRF4       142 LM---LAFVAH--FP---------------------------SLSTDFA-----------
AtUGT71B1    136 SY---LGLQFH--VQ---------------------------SLYDEKELDVSEFKDT--
W1P1M0       152 TL---LSLMLH--LP---------------------------TLDQEIS---GEYRDL--
AtUGT72B1    143 NV---LSFFLH--LP---------------------------KLDETVS---CEFREL--
W1PMA9       147 TA---LCLMSQ--LP---------------------------ALDAQVS---GEFRDL--
W1PP20       145 TF---LSLVCQ--LP---------------------------ALDTKVS---GEYRDL--
AtUGT88A1    145 AC---LAFSFY--LP---------------------------TIDETTP---GKNLKD--
U5DB27       111 SL---LSLMLY--LP---------------------------TLHDQIA---SEFTDL--
U5DBY1       141 SL---LSLMLY--LP---------------------------TLHDQIT---SEFRDL--
U5DBY4       150 NS---LALMLH--LP---------------------------TLHDQIP---CEFKDC--
U5D276       149 NL---LSLMLY--LP---------------------------TLHDQIP---CEFRDC--
U5DB32       149 NF---LSLMLF--LP---------------------------TLHEQIP---CEFRDW--
W1NHX5       142 AD---LAIFLY--TP---------------------------TLHSRIP---ISFRDQ--
W1NVU7       148 AD---LVVFLY--LP---------------------------ILHFQIP-----------
W1PAP0       141 AF---LVLFLY--FP---------------------------ELHKQNP---GTLKDL--
W1PD80       137 AF---LTVFLY--FP---------------------------ELNKQNP---GKFRDL--
W1PZW0       140 GF---LDIFFY--FP---------------------------ELHKENP---GSFTDL--
AtUGT82A1    135 AA---YRLIQA--IP---------------------------ELVRTGLVSQKGCPRQ--
W1NUV1       275 LV---FSLYHHAHLL---------------------------RQHGHFDSEGKNRKDP--
AtUGT86A1    155 LV---LNLYYH--MD---------------------------LLISNGHFKSLDNRKD--
AtUGT87A1    142 TI---LSLFINSDLL---------------------------ASHGHFPIEPSESKLD--
W1P188       148 TV---FSVFYHCEVL---------------------------LSKGHFPANSSANKEE--
W1P188         1 ------------------------------------------------------------
```

```
W1P302       152 AI---FSIYYHFDLL---------------------------FSKGHFSGSSTVSQEE--
AtUGT78D1    145 NS---LCAHLY--TD---------------------------LIRETIGLKDVSMEET--
W1PTN8       143 CS---LAAHFY--TK---------------------------KIREEVKLGDNGIKDE--
ZmUGT77A1    156 CA---LLAHIR--TD---------------------------ALREDVGDQAANRVDG--
AtUGT83A1    154 AS---MVLGFS--IQ---------------------------KLIDDGLIDSDGTVRV--
W1NWE7        61 ------------------------------------------------------------
W1NXD5       146 GC---LTVFLK--LQ---------------------------ELIQKGV---ITKEGE--
AtUGT75B1    139 LV---FNIYYT--HF---------------------------MGNKSVF-----------
AtUGT84A1    153 AC---FSAYYH-------------------------------YQDGSVSFPTETEPEL--
W1PLB6       145 AI---YALYYR--SV---------------------------YRLSEFP---TPEKPD--
U5CXN6       144 SV---YALYYR--SF---------------------------YRLGEFP---TPERPD--
U5D2S8       136 AV---YAVYYR--SL---------------------------YRLGEFP---TPERPN--
U5D817       143 AV---YAIYYQ--SL---------------------------YRLGEFP---TPERPN--
W1NFL7       143 AV---YAVCYQ--SL---------------------------YRLDEFP---TPERLN--
W1NFN9       143 AV---YAVYYQ--SL---------------------------CRLDEFP---TPERLN--
U5DBW3       145 AV---YGYYYR--VL---------------------------HRNRAEM-----------
W1PE57       144 LV---YSYYYR--VY---------------------------GGLGANG-----------
U5D3G1       132 TV---FVIYWN-------------------------------HFRGSKS---SSYLIS--
AtUGT74B1    139 TV---CSVLRK-------------------------------FSNGDFP---LPADPN--
U5DFQ6       146 AV---FALYFR-------------------------------YHEGSLSICDEKAETM--
U5DI88       174 AV---NTIYRH--------------------------------LHGKFS---GKETKL--
U5DCN3       142 AI---NTLYLL--------------------------------HHDKFS---DKKTDI--
U5D531       142 AV---CLTYCY--YG---------------------------------------------
U5CUG8       142 AV---CLMYCY--YG---------------------------CGLQINP-----------
W1PLP3       142 AV---CLMYCY--YG---------------------------CGLQINP-----------
U5DI92       142 AV---CTIYIR--YI---------------------------RGFPVLP------KEP--
U5D3V6       140 AV---CSIYNR--YR---------------------------RGLPVMP------KEL--
U5DDJ3       142 AV---GSIYNR--YL---------------------------HGHPVRP------KEP--
U5DFQ3       142 AV---CSIYDR--YL---------------------------RDLPVTP------KEP--
U5DI97       142 AV---SSIYSL--CH---------------------------RSILVVQ------REP--
U5DCP2       143 AVSSMCSLYHR--CA---------------------------LVAQAEP-----------
U5DDK2       143 AV---SSMYSL--YH---------------------------RGALVMQ------AEP--
OsUGT709A3   154 CS---FLAYMS--VP---------------------------RLVELGELPFPPGGDL--
SgUGT720A1   150 RF---MFLYFW--IP---------------------------KLIEDGQ---LPYPNG--
U5CX27        62 LE----------------------------------------------------------
U5D008        61 TH---MVDVVL--LA---------------------------NADENFM---TNGELE--
W1PMY7        57 CG---YWAFLQ--IP---------------------------ELIRRGYTPLKDMACL--
AtUGT85A1    152 CA---FLAYLH--FY---------------------------LFIEKGL---CPLKDESY
U5CVP6       149 SG---LWGYLQ--YT---------------------------ELIERGYVPLKEESYL--
U5CNJ5       148 SS---FLAFLQ--YP---------------------------ELQERGYIPLKDESQL--
U5CNG3       149 CG---FWAYLH--YP---------------------------ELVERGYTPLKDASQL--
U5CXJ9       149 CG---LWAYLQ--YP---------------------------ELIERGYTPLKDASQL--
AtUGT76B1    135 SA---FVAFSK--FH---------------------------VLREKGYLSLQETKAD--
W1PV81       139 TS---FAVFAN--IE---------------------------KLHEMGY---LPIRDF--
W1PVY0       142 TS---TLGFQA--------------------------------IRDKGY---LDNRGH--
W1PX56       175 TS---TLGFQA--------------------------------IREKGY---LDERAD--
AtUGT90A1    151 YS---AAVSIS-------------------------------VFKHELFTEPESKSDT--
OsUGT97A1    170 FS---LALCFT--LA---------------------------SRRPHVGVEG--------
W1PH20       152 YS---MSICKS--VC---------------------------VHYPQKGLTE--------
W1PBB6       170 YA---TCLSMA--IA---------------------------TYGSPIP-------KD--
W1PHN8       170 YA---TCLSLA--IA---------------------------AYGSPIP-------KD--
W1PRF4       142 LM---LAFVAH--FP---------------------------SLSTDFA-----------
AtUGT71B1    136 SY---LGLQFH--VQ---------------------------SLYDEKELDVSEFKDT--
W1P1M0       152 TL---LSLMLH--LP---------------------------TLDQEIS---GEYRDL--
AtUGT72B1    143 NV---LSFFLH--LP---------------------------KLDETVS---CEFREL--
W1PMA9       147 TA---LCLMSQ--LP---------------------------ALDAQVS---GEFRDL--
W1PP20       145 TF---LSLVCQ--LP---------------------------ALDTKVS---GEYRDL--
AtUGT88A1    145 AC---LAFSFY--LP---------------------------TIDETTP---GKNLKD--
U5DB27       111 SL---LSLMLY--LP---------------------------TLHDQIA---SEFTDL--
U5DBY1       141 SL---LSLMLY--LP---------------------------TLHDQIT---SEFRDL--
U5DBY4       150 NS---LALMLH--LP---------------------------TLHDQIP---CEFKDC--
U5D276       149 NL---LSLMLY--LP---------------------------TLHDQIP---CEFRDC--
U5DB32       149 NF---LSLMLF--LP---------------------------TLHEQIP---CEFRDW--
W1NHX5       142 AD---LAIFLY--TP---------------------------TLHSRIP---ISFRDQ--
W1NVU7       148 AD---LVVFLY--LP---------------------------ILHFQIP-----------
W1PAP0       141 AF---LVLFLY--FP---------------------------ELHKQNP---GTLKDL--
W1PD80       137 AF---LTVFLY--FP---------------------------ELNKQNP---GKFRDL--
W1PZW0       140 GF---LDIFFY--FP---------------------------ELHKENP---GSFTDL--
AtUGT82A1    135 AA---YRLIQA--IP---------------------------ELVRTGLVSQKGCPRQ--
W1NUV1       275 LV---FSLYHHAHLL---------------------------RQHGHFDSEGKNRKDP--
AtUGT86A1    155 LV---LNLYYH--MD---------------------------LLISNGHFKSLDNRKD--
AtUGT87A1    142 TI---LSLFINSDLL---------------------------ASHGHFPIEPSESKLD--
W1P188       148 TV---FSVFYHCEVL---------------------------LSKGHFPANSSANKEE--
W1P188         1 ------------------------------------------------------------
```

```
U5CYW0       158 ISVHPLMQHIPLWV--------------LKWQEL---HK-KV-VFVTV--ITDLNTC-HP
W1PAZ2       231 -----------III----SAH-----PLMQHVPL------RI-LRAKGLLKKIVFTT---
AtUGT81A1    234 --------EIAQGLMKYQPDIIISVHPLMQHVPL------RV-LRSKGLLKKIVFTT---
W1PTT2       197 --------EEMEVV----TGSGEV--VRCSEREN---GELFF-GVLGGLGQFGIITR---
U5D3V5       197 --------LQMEIV----TGKGEV--ITCSHTEE---EDLFY-GALGGLGQFGIITK---
W1NUG7       198 --------YELDVV----TGKGEL--LTCSKHQN---SE-LFHSVLGGLGQFGIITR---
W1PC48       188 --------YQLEVV----TGKGEV--VNCSAQKN---AD-LFHAVLGGLGQFGIITK---
W1PP63       178 --------LKLDVI----TGTGE--IVRCSPSKR---AELFY-GVLGGLGQFGIITS---
OsUGT701A2   192 --------LQLEVV----TGKGEV--VTCSPTEI---PELFF-AVLGGLGQFGIITR---
W1PA48       190 -------------F----PVL-----YRCLQEAH---PG-KV-CWNDV--LHWMWPL---
U5D0T8       198 AEALKVPLHIFFTM------------PWTPTSEF---PH-PLSRVKQP--AGYRLSYQ--
AtUGT80A2    317 AEALKIPIHVFFTM------------PWTPTSEF---PH-PLSRVKQP--AGYRLSYQ--
W1PJ19       124 ------------------PGFPP---SALHLYPF---EAEQIIHAYTL--LESNATM---
W1NU70       168 ------LSSPPFGF----PSSSLYV-QRYEAEQL---LW-IY-TYPEP--DISGIDR---
W1NV64       168 ----------PPDFP----PSA-----VYFHPFEA---KQ-LQ-SLSTF--QKTDVSS---
W1PKS4       166 ------LTSSPLDF----PLSA----IYFHPFEA---KQ-QV-GAHTF--PEPDIPC---
AtUGT91A1    176 ----------PKWV----PFETSVAFKLFECRFI---FKGFMAETTEG--NVPDIHR---
U5CMK2       168 ---------APEWVPF--PSI-----VALRMHEA------ARVSQLGNKVEASGMSS---
U5CWM7       158 ---------VPGWI----PFPST---IAFRPHEV---HK-IF-QDASF--DIPNASG---
U5D1X9       167 --------VVPEWITF--PST-----IAFRRHEA---QA-FL-QQVLTNLHNGSGVS---
W1PT00       171 --------ESQRIQ----PMLEQ---QTFGMRDL----------ERYKS-----------
OsUGT94A1    171 ---------EAISL----GGA---------EEDA---RYTMLTCRDDGTALLPKGER---
AtUGT79B1    172 -------AKTPLGY----PSSK----VVLRPHEA---KS-LS-FVWRK--HEAIGSF---
W1PQK6       176 --------RSPLGF----PSGCN---FVLRPFEA---RQ-FMYLYTSFAGKPSVYDR---
U5D602       170 ------------SL-------------RVLPPSSL------CV--------YEGFAAS---
U5D5X9       147 ------------------PGL-----PPPSFQNC---VS----ESFMD------------
U5D5Z2       199 ------------------PIV-----PGLPPPSF---RG-CV--------NDSFIDF---
U5D5X3       124 ------------------PVV-----PGLSPLIL---RS-FI--------NESFMDV---
ZmUGT93B1    162 ------------------PGH-----RLLSDYGLQFLPPDAC-MSREF--VDLVFR----
PoUGT95A1    191 ------------------PGLPKE--MAVTFADLSRGPQRRI-RPPGGPGKSDGRAG---
U5D0B7       186 IESQRVFGNKNQAF----PGWESN--PPPLPENM---SLFHKEMPFDRKLPPPLPES---
AtUGT89A1    170 -------STEPVCL----SDLPRS--PVFKTEHL---PS-LI--PQSP--LSQDLES---
OsUGT96A1    167 --------VVLDTL----PGS-----PAFPYEHV---PS-VV-RSYVA--GDPDWEV---
AtUGT92A1    184 ------------DF----PEA-----GEIEKTQL---NS-FM-LEADG--TDDWSVF---
U5CRD3       138 --------FEHIAL----PDFPK---TTLHRSQL---TN-EM-KLADG--TDFASVF---
W1P7W1       177 --------SNEFEL----PGLPG---LKLQRSQL---TA-AL-KAADG--TDPFSAF---
U5D7I9       168 --------ADEFPV----PGMPG---VRLHRTQL---AS-HL-RVADG--KDGWSIF---
W1PFP0       168 --------ADEFPV----PGMPG---VRLHRKQI---PS-HL-RVADG--KDGWSIF---
OsUGT99A1    173 ---------APVVV----PGLARR--VEVTRAQA---PGFFR--------DIPGWEK---
AtUGT73B1    180 ---------EPFVI----PDLPGD--ILITEEQV---------METE---EESVMGR---
OsUGT98B1    184 ---------EIVKV----SGF-------PTPLEL---PK-AR-CPGTL--CVPGLKQ---
W1PF90       192 --------HEPFEL----PGLS------IRLTKANL---PP-LM-RNPSA--EPPGVIE---
W1NIK4       174 --------TEPFVL----PGLSDE--ITVTKAQL---PE-HSKENIAW--IVSGLGK---
W1PQX3       178 --------AEPFLL----PGLPGE--LTFTRTQL---PE-FWKVNVEL--MASGFDR---
W1PPW8       177 --------TEPFML----PGLPHD--ISLTRAQL---SE-FSKGNSEL--MAAGFSR---
W1PS80       177 --------TEPFML----PGLPHD--IRLTRAQL---AE-FSKGNSEL--MAAGFSR---
AtUGT90A1    175 ---------EPVTV----PDF-----PWIKVKKC----D-FDHGTTEPEESGAALEL---
OsUGT97A1    190 --------GAEFHV----PGFPDD--VRITADEV---PDAVI-QGGNP--DDPVTQF---
W1PH20       172 --------TEPFRV----PGMPSP--VVITRAEL---SS-PV-KDLDP--NDPSFQLL--
W1PBB6       189 --------SEPVLL----PGLPHE--VRLTHVEV---PP-TF-RTLDP--KDPSWAFV--
W1PHN8       189 --------SEPVLL----PGLPHE--VRLAHVEV---PP-TF-RSLDP--KDPSWTFV--
W1PRF4       159 --------NHPVDI----PGL-----PPFPPASI---PP-AL---KDP--NHAFTTL---
AtUGT71B1    162 --------EMKFDV----PTLT----QPFPAKCL---PS-VM-----L--NKKWFPY---
W1P1M0       175 --------DFPVQV----PGC-----FPIPGKDL---PE-QL-QDRED--EDPRAS----
AtUGT72B1    166 --------TEPLML----PGC-----VPVAGKDF---LD-PA-QDRKD--DAYKW-----
W1PMA9       170 ---------PELRL----PGCS----SPFPGKDL---LA-PL-QDRKD--AAYSW-----
W1PP20       168 ---------RELRL----PGCS----SPFPGKDL---VS-PL-QDRKD--AAYAW-----
AtUGT88A1    168 --------IPTVHI----PGV-----PPMKGSDM---PK----AVLER--DDEVYDV---
U5DB27       134 --------KEPVAI----PGM-----TPLPPLDM---PTTLM-NKTND------------
U5DBY1       164 --------KEPVAI----PGM-----TPLPPLDM---PM-PV-MDKTN--DAYTW-----
U5DBY4       173 --------KEPIIV----PGL-----PPVPPIDL---PL-VL-SDKTN--DAYKW-----
U5D276       172 --------KNPIPV----PGL-----PPVPAIDL---PL-VL-QDRTN--DAYKW-----
U5DB32       172 --------KNPIPV----PGL-----PPVPPIDM---PV-AF-QDKTN--DAYKW-----
W1NHX5       165 --------KELLIV----PGL-----PLVPPTSM---PD-PL-LDRTN--KAYDW-----
W1NVU7       165 --------------------------IRFRDQ---KE-LLQVPEPP-----------
W1PAP0       164 --------DRDLFI----PGL-----PPMPLSVF---PD-SM-QDRSQ--KLYQW-----
W1PD80       160 --------ACDLVL----PGL-----PPLPPSLI---PE-VM-QDRNK--KVYEW-----
W1PZW0       163 --------DRDLVV----PGI-----PPFPPRIM---PT-VM-QDKSQ--DVYDW-----
AtUGT82A1    161 --------LEKTIV---QPEQ-----PLLSAEDL---PW-LI-GTPKA--QKKRFKF---
W1NUV1       303 ----------IDYI----PGI-----PSIDPKDI---PS-YL-QVSDT--STVVHQI---
AtUGT86A1    181 ---------VIDYV----PGV-----KAIEPKDL---MSYLQVSDKDVDTNTVVYRI---
AtUGT87A1    170 --------EIVDYI----PGL-----SPTRLSDL------QILHGY----SHQVFNI---
W1P188       176 ---------YVDYI----PGV-----SPTRILEL---PN-LFKGGESR--EIAESLK---
W1P188         1 ------------------------------------------------------------
```

```
W1P302      180 ---------HVDYL----PGV-----SLTRILDL---PH----VGGRE--EAQQRAR---
AtUGT78D1   171 ----------LGFI----PGM-----ENYRVKDI---PE-EV-VFEDL--DSVFPKA---
W1PTN8      169 ---------NVAGI----PAL-----SFIKLKEL---PE-GV-IRGDL--SSPFPLM---
ZmUGT77A1   182 ---------LLISH----PGL-----ASYRVRDL---PD-GV-VSGDF--NYVINLL---
AtUGT83A1   180 --------NKTIQL---SPGM-----PKMETDKF---VW-VCLKNKES--QKNIFQL---
W1NWE7       61 ------------------PNT-----PLIDPSHF---PW-LCVGDTMV--QKFIYRY---
W1NXD5      169 --------LLDTHMIQFSPKM-----PLMNPRHF---AW-LCVGDTAE--NKVIYRS---
AtUGT75B1   156 ------------EL----PNL-----SSLEIRDL---PS-FL-TPSNT--NKGAYDA---
AtUGT84A1   177 ----------DVKL----PCV-----PVLKNDEI---PS-FL-HPSSR--FTGFRQA---
W1PLB6      168 ---------IDISL----PGL-----PVLQASEL---PS-FL-LPSAA--HRCLADV---
U5CXN6      167 ---------IDVSL----PGL-----PVLKRSEI---PS-FL-VPFEPTLYQCLGDV---
U5D2S8      159 ---------IDISL----RGL-----PVLKTNEL---PS-FL-VPFAP--YQCLADA---
U5D817      166 ---------IDVSL----PGL-----PVLKTNEL---PS-FL-VPFAP--YQCLADA---
W1NFL7      166 ---------IDVSL----RGL-----PVLKTNEL---PS-FL-VPFGP--YQCLADA---
W1NFN9      166 ---------IDVSL----RGL-----PVLKTNEL---PS-FL-VPFAP--YQCLADA---
U5DBW3      162 --------EVPLTF----PGL-----PHLKPEDF---PT-LA-SPSST--YNRFPLL---
W1PE57      161 --------EVPVNL----PGL-----PELKLEDL---PS-LS-LPSNP--YKSFPRI---
U5D3G1      153 --------TTNVRV----TGM-----PLLNIEDL---PS-FL-QPSNT--NNIAVES---
AtUGT74B1   160 --------SAPFRI----RGL-----PSLSYDEL---PS-FVGRHWLT--HPEHGRV---
U5DFQ6      170 ------------EV----PGL-----PMLGLQDI---PS-FL-APSNP--YPAHLDL---
U5DI88      194 --------ELPLSL----PGM-----PLLDSLDL---PS-LI-AQPEA--YPAYLKL---
U5DCN3      162 --------TTPISV----PGM-----PILESSDL---PS-YM-SEPEL--YPAYLKMV--
U5D531      152 ------------------RGL-----QIIATCAF------FF------------------
U5CUG8      159 --------DMCVFL----PRM-----PPLGLRDL---PS-FI-AQPES--NPLILEM---
W1PLP3      159 --------DMCVFL----PRM-----PPLGLRDL---PS-FI-AQPES--NPLILEM---
U5DI92      162 --------DGCVAI----PGL-----PLLGLRDL---PS-FI-TQTEG--DPYHLEL---
U5D3V6      160 --------NGRVAI----PGM-----PLLGLRDL---PS-FI-AQPEG--YGFVLEM---
U5DDJ3      162 --------NGHVTI----PGM-----PLLGLRDI---PS-FI-AQPEG--NRFILEW---
U5DFQ3      162 --------NGYVAI----PGM-----PLLGLRDI---PS-FI-AQPES--YGFFLEM---
U5DI97      162 --------GARYSL----PGL-----PLLSLQDL---PS-FI-VQSEG--NGFFLDL---
U5DCP2      163 --------DACFSL----PGM-----PPLSLQDL---PK-FI-AQPKA--DRFLLDL---
U5DDK2      163 --------DACFSL----PGM-----PPLGLQDL---PK-FI-AQPEA--DRFFLEL---
OsUGT709A3  180 -------DEPVRGV----PGME----GFLRRRDL---PS-PCRHHGAN--NNDDAAA---
SgUGT720A1  173 --------NINQKL----YGVAPEAEGLLRCKDL---PG-HWAFADEL--KDDQLNF---
U5CX27       64 --------AIVDWI----PGM-----PDIRLRDL---PS-FI-RTTNL--NDPMLQV---
U5D008       84 --------AVVDWI----PGM-----PNIRLRDS---PS-FI-RTTNL--NDPM------
W1PMY7       83 --AEGYLDTTLDWI----PGM-----PNILLKDM---PS-FI-RTTDP--NFFLLNY---
AtUGT85A1   177 LTKEYLEDTVIDFI----PTM-----KNVKLKDI---PS-FI-RTTNP--DDVMISF---
U5CVP6      175 --SNGYLNTPLDWI----PGM-----PGAQLKDI---PS-FI-RTTNP--EDIMFNF---
U5CNJ5      174 --NNGYLETPLDWI----DGM-----LGIRLRDM---PS-LI-RTTDP--NDIMLQF---
U5CNG3      175 --TNGHLETPLNWI----QSM-----PSIRLRDM---PS-FL-RTTDP--DDIMLNFV--
U5CXJ9      175 --TNGYLETPLNWI----QSM-----PSIRLRDM---PS-FL-RTTDT--DDIMLNFV--
AtUGT76B1   161 -----------SPV----PEL-----PYLRMKDL---PW-FQTEDPRS--GDKLQIG---
W1PV81      162 --------ESEERI----SEL-----PPLRIKDL---PA-FS-SDNSV--LESFWAG---
W1PVY0      162 --------PSDEPI----PDF-----SPFRFKDL---P--FV-NTKDP--DSLCL-----
W1PX56      195 --------TSDELV----PDL-----SPIRFKDL---P--FV-KTK----YPDYFSL---
AtUGT90A1   175 ---------EPVTV----PDF-----PWIKVKKC----D-FDHGTTEPEESGAALEL---
OsUGT97A1   190 --------GAEFHV----PGFPDD--VRITADEV---PDAVI-QGGNP--DDPVTQF---
W1PH20      172 --------TEPFRV----PGMPSP--VVITRAEL---SS-PV-KDLDP--NDPSFQLL--
W1PBB6      189 --------SEPVLL----PGLPHE--VRLTHVEV---PP-TF-RTLDP--KDPSWAFV--
W1PHN8      189 --------SEPVLL----PGLPHE--VRLAHVEV---PP-TF-RSLDP--KDPSWTFV--
W1PRF4      159 --------NHPVDI----PGL-----PPFPPASI---PP-AL---KDP--NHAFTTL---
AtUGT71B1   162 --------EMKFDV----PTLT----QPFPAKCL---PS-VM-----L--NKKWFPY---
W1P1M0      175 --------DFPVQV----PGC-----FPIPGKDL---PE-QL-QDRED--EDPRAS----
AtUGT72B1   166 --------TEPLML----PGC-----VPVAGKDF---LD-PA-QDRKD--DAYKW-----
W1PMA9      170 ---------PELRL----PGCS----SPFPGKDL---LA-PL-QDRKD--AAYSW-----
W1PP20      168 ---------RELRL----PGCS----SPFPGKDL---VS-PL-QDRKD--AAYAW-----
AtUGT88A1   168 --------IPTVHI----PGV-----PPMKGSDM---PK----AVLER--DDEVYDV---
U5DB27      134 --------KEPVAI----PGM-----TPLPPLDM---PTTLM-NKTND------------
U5DBY1      164 --------KEPVAI----PGM-----TPLPPLDM---PM-PV-MDKTN--DAYTW-----
U5DBY4      173 --------KEPIIV----PGL-----PPVPPIDL---PL-VL-SDKTN--DAYKW-----
U5D276      172 --------KNPIPV----PGL-----PPVPAIDL---PL-VL-QDRTN--DAYKW-----
U5DB32      172 --------KNPIPV----PGL-----PPVPPIDM---PV-AF-QDKTN--DAYKW-----
W1NHX5      165 --------KELLIV----PGL-----PLVPPTSM---PD-PL-LDRTN--KAYDW-----
W1NVU7      165 -----------------------------IRFDQ---KE-LLQVPEPP------------
W1PAP0      164 --------DRDLFI----PGL-----PPMPLSVF---PD-SM-QDRSQ--KLYQW-----
W1PD80      160 --------ACDLVL----PGL-----PPLPPSLI---PE-VM-QDRNK--KVYEW-----
W1PZW0      163 --------DRDLVV----PGI-----PPFPPRIM---PT-VM-QDKSQ--DVYDW-----
AtUGT82A1   161 --------LEKTIV---QPEQ-----PLLSAEDL---PW-LI-GTPKA--QKKRFKF---
W1NUV1      303 ----------IDYI----PGI-----PSIDPKDI---PS-YL-QVSDT--STVVHQI---
AtUGT86A1   181 ---------VIDYV----PGV-----KAIEPKDL---MSYLQVSDKDVDTNTVVYRI---
AtUGT87A1   170 --------EIVDYI----PGL-----SPTRLSDL------QILHGY----SHQVFNI---
W1P188      176 ---------YVDYI----PGV-----SPTRILEL---PN-LFKGGESR--EIAESLK---
W1P188        1 ------------------------------------------------------------
```

```
U5CYW0      196 TWFNTSVTRC-----------YC--PSEE---------VAKRALLK-GLES--D-QVRVF
W1PAZ2      261 --VITDLSTC---------------HPTW-------FHKLVTRCYCPTAEVAQRASKAGL
AtUGT81A1   276 --VITDLSTC---------------HPTW-------FHKLVTRCYCPSTEVAKRAQKAGL
W1PTT2      236 --ARILLQKA-----------PD--MVRW-------IRVVYADFDD--FSRDQELLVTRA
U5D3V5      236 --ARIAIQPA-----------PQ--RTRW-------IRAIYEEFEE--FKGDQE-MLISM
W1NUG7      237 --ARIALEPA-----------PQ--RVRW-------IRVLYSDFNT--FTRDQE-HLISL
W1PC48      227 --ARIALEPA-----------PE--MVKW-------IRVLYSDFSL--FTKDQE-LLISL
W1PP63      217 --ARIVLEDA-----------PH--KVKW-------VRAFYDDFGA--FTEDQE-MLVSM
OsUGT701A2  231 --ARIPLQLA-----------PP--KVRW-------VRAFYDSFET--FTGDQE-LLVSM
W1PA48      218 --FMEDWGSW---------------RSNF---------LKLSSCPL--MDP--VTGLPLL
U5D0T8      238 --IVDSLIWL---------------GIRD----------MINDFRKKKLKLRPVTYLSGA
AtUGT80A2   357 --IVDSLIWL---------------GIRD----------MVNDLRKKKLKLRPVTYLSGT
W1PJ19      155 --AHRFLQNI------------S--DSDM---------VIFRSCNE--MEG--K-FLEFL
W1NU70      207 --LLRTISNS-----------NM--------------VIVRSCYE--LEG--K-YFEYL
W1NV64      200 --FERFTRTI------------C--NSHM---------VIIRSCYE--LEK--K-YIDYL
W1PKS4      202 --AERLFRSI------------S--NSHM---------VIIRSCYE--LEK--K-YIDYI
AtUGT91A1   214 --VGGVIDGC---------------DV-----------IFVRSCYE--YEA--E-WLGLT
U5CMK2      203 --HQRFAMTI------------E--GCDF---------VAVRSCYE--FEG--K-YLELL
U5CWM7      192 --VSDGYRFG-----------STIEGCRV---------VAVRTCNE--FEE--G-YLDLL
U5D1X9      204 --DWYRVQSI-----------LE--GCRV---------VAFRTGKE--FDG--D-YIDLF
W1PT00      195 --CVEQSTDF----------------------------LTCRSMHD--IEG--K-FINYL
OsUGT94A1   203 --LPLSFAR----------------SSEF---------VAVKTCVE--IES--K-YMDYL
AtUGT79B1   207 --FDGKVTAM------------R--NCDA---------IAIRTCRE--TEG--K-FCDYI
W1PQK6      214 --VTRSIR-----------------EADA---------LLFKTCSE--MEG--P-FCDYI
U5D602      189 --IARRQQPL------------K--FDQG---------ILYNTFEA--IEG--Q-FLQQL
U5D5X9      165 --FLQRTGRI-----------QH--LDRG---------VLHNTFHA--LEG--Q-FLSKI
U5D5Z2      221 --LPRAVKFR------------H--LDRG---------VFFNSFHA--IEG--Q-FLSRI
U5D5X3      146 --LARAVRFQ------------H--LDRG---------LLFNTFHS--LEG--Q-FLSKI
ZmUGT93B1   192 --MEEEEQGA---------------PVAG---------LVMNTCRA--LEG--E-FLDVV
PoUGT95A1   227 --PPNRMRSGSRHGPGGGPSPGPGQKPRWVDEVDGSIALLINTCDD--LEH--V-FINYM
U5D0B7      234 --MMSSAGKMFSGQGPLPPPPQPGGPPRWMEETQGSVVLMINTCSD--LER--P-FLDYM
AtUGT89A1   206 --VKDSTMNF---------------SSYG---------CIFNTCEC--LEE--D-YMEYV
OsUGT96A1   200 --ALEGFRLN------------A--RAWG---------AVVNSFDE--MER--E-FLEWL
AtUGT92A1   213 --MKKIIPGW------------S--DFDG---------FLFNTVAE--IDQ--M-GLSYF
U5CRD3      173 --LRRHIAYC------------M--RTGG---------TLCNTLEG--LEG--I-GIEQL
W1P7W1      212 --VRKNVLTS------------L--RSKI---------MICDTVEE--LEP--K-GIQQL
U5D7I9      203 --LQKHISLS-----------MH--RSRG--------DMLCNTVAE--LEP--D-GIRLL
W1PFP0      203 --LQKHISLS-----------MH--RSRG--------DMLCNTVAE--LEP--D-VLRLL
OsUGT99A1   204 --FADDLERA-----------RA--ESDG---------VVINTVLE--MEP--E-YVAGY
AtUGT73B1   210 --FMKAIRDS-----------ER--DSFG---------VLVNSFYE--LEQ--A-YSDYF
OsUGT98B1   214 --ISDKIYEA-----------ET--RSDG---------RIMNSFQE--MES--L-YIESF
W1PF90      226 --FFNEIRRA-----------DS--ESAG---------IIFNSFYE--LEP--D-YFEHC
W1NIK4      211 --VLEEMQKA-----------DA--QSYG---------TVVNSFHE--LEP--R-YVDYY
W1PQX3      215 --VFEVMRRA-----------DV--QSYG---------TLVNSFNE--LEP--E-YVDHY
W1PPW8      214 --VLEEMRRA-----------NS--HSYG---------SIVNSFEE--LES--E-YVNHY
W1PS80      214 --VLEEMRRA-----------NA--HSYG---------SIVNSFEE--LES--E-YVNHY
AtUGT90A1   209 --SMDQIKST------------T--TSHG---------FLVNSFYE--LES--A-FVDYN
OsUGT97A1   227 --LHDEVRDW-----------DH--RSWG---------VLVNSFAA--LDGDYAAILESF
W1PH20      209 --CEVGLADI---------------ESDG---------VLVNTFYE--LEP--A-WVDCF
W1PBB6      226 --MEAGVATF---------------RSYG---------EILNSFYE--LEP--E-FVEAL
W1PHN8      226 --LETGAAKL---------------RSYG---------EILNSFYE--LEP--E-FVKTL
W1PRF4      190 --FQANSNAL------------P--KATG---------IILNTFRA--FET--D-ALSAL
AtUGT71B1   192 --VLGRARSF------------R--ATKG---------ILVNSVAD--MEP--Q-ALSFF
W1P1M0      207 -------------------------SNKN-----------------------LER--QEAWGAI
AtUGT72B1   197 --LLHNTKRY------------K--EAEG---------ILVNTFFE--LEP--N-AIKAL
W1PMA9      201 --LLHQAKRY------------R--QADG---------IFVNTFEG--LES--Q-ALQFL
W1PP20      199 --FLHQAKRY------------P--RADG---------IFVNTFEA--LES--Q-ALQFL
AtUGT88A1   199 --FIMFGKQL------------S--KSSG---------IIINTFDA--LEN--R-AIKAI
U5DB27      161 -------------------------AYTW---------FLYTPAPS---------CLPGH
U5DBY1      195 --FLYHSRRL------------R--EVEG---------ILVNTFEE--FEP--Q-PLKAI
U5DBY4      204 --FLYNSRRF------------H--QAKG---------ILINTFLD--LET--R-SLDAL
U5D276      203 --FLYHAGRL------------R--ESKG---------IIINTFAE--LEP--L-TLKAL
U5DB32      203 --FLYHCRRL------------R--ESKG---------IIVNTFAE--LEP--L-TLKAL
W1NHX5      196 --FVKACNRI------------A--LTQG---------ILVNSFDE--LEP--PRALRAL
W1NVU7      181 --------------------------------------RVLEALAS------GECTPDV
W1PAP0      195 --CLHHASRL------------P--RGRG---------IIINSFHD--LEP--L-GMRAL
W1PD80      191 --FLYHSAKV------------P--QGKG---------ILLNSFEE--LEPLGLKALQAL
W1PZW0      194 --CMYYASTM------------P--KADG---------ILVNTFEQ--LEP--L-ALKAL
AtUGT82A1   195 --WQRTLERT------------K--SLRW---------ILTSSFKD-EYEDVDN-HKASY
W1NUV1      334 --ILAAFNGA------------K--RADW---------VLSNTVEE--LEL--H-TIEAL
AtUGT86A1   217 --LFKAFKDV------------K--RADF---------VVCNTVQE--LEP--D-SLSAL
AtUGT87A1   200 --FKKSFGEL------------Y--KAKY---------LLFPSAYE--LEP--K-AIDFF
W1P188      209 --FFHQIQNA---------------KS-----------ILFNSFYE--LEP--S-AIDAL
W1P188        1 ------------------------------------------------------------
```

```
W1P302      210 --CLQFFQQF------------Q--QVNF---------ILIYSFYE--LES--P-TIDAL
AtUGT78D1   202 --LYQMSLAL------------P--RASA---------VFISSFEE--LEP----TLNYN
W1PTN8      201 --LLKMGEQL------------P--QATA---------VILNCFED--LDP--S-TLSDL
ZmUGT77A1   214 --VHRMGQCL-----------PR--SAAA---------VALNTFPG--LDP--PDVTAAL
AtUGT83A1   215 --MLQNNNSI------------E--STDW---------LLCNSVHE--LET----AAFGL
W1NWE7       89 --IRQFMKNL------------E--GVEW---------VLCNSFYE--LEA--A-TFDSF
W1NXD5      207 --ADVCIKGL------------E--GVEW---------LICNSFYE--IES--T-TYDTF
AtUGT75B1   185 --FQEMMEFL-----------IK--ETKP--------KILINTFDS--LEP--E-ALTAF
AtUGT84A1   208 --ILGQFKNL------------S--KSFC---------VLIDSFDS--LEQ--E-VIDYM
W1PLB6      200 --ILSHIKKI------------K--KVKW---------VLGNSFYA--LET--E-AIQLM
U5CXN6      201 --IFDHIRKL------------K--KVRW---------VLGNSFHA--LET--E-AIQSM
U5D2S8      191 --ILCHIRKI------------N--KVKW---------VLANSFHA--LET--E-AIRSM
U5D817      198 --ILGHIKKI------------K--KVKW---------VLANSFHA--LET--E-AIQSV
W1NFL7      198 --ILGHIKKI------------K--KVKW---------VLANSFHA--LET--E-AIQSV
W1NFN9      198 --ILGHIKKI------------K--KVKW---------VLANSFHA--LET--E-AIQSV
U5DBW3      195 --LTQLYEKL------------G--DVKM---------VLGNSFNE--LEPEEIWFAEGL
W1PE57      194 --LTQLYARL------------G--DVRM---------VLGNSFDE--LEPEEIRFVEGL
U5D3G1      186 --LVELFEML------------DSRRIDW---------VIASTFDA--LEK--D-LISAM
AtUGT74B1   194 --LLNQFPNH------------E--NADW---------LFVNGFEG--LEETQD-CENGE
U5DFQ6      199 --LLSQFSNL------------D--KAQY---------LFVNSFYE--LEE--EAIVSLM
U5DI88      227 --VMEQFRNM------------D--KADF---------VLENSFES--LEG--E-FSREL
U5DCN3      196 --VLDQLSNM------------D--QADF---------VLANSFDS--LEG--E-LSREL
U5D531      165 ----------------------P--ECRH---------WAYETSPR--SSP----NVRAI
U5CUG8      192 --MLGQFKNM------------G--KADF---------VLVNSFDK--LEE--E-ARKEM
W1PLP3      192 --MLGQFKNM------------G--KADF---------VLVNSFDK--LEE--E-ARKEM
U5DI92      195 --LSGQFKNI------------D--QADY---------VLVNSFDS--LEA--E-VRREM
U5D3V6      193 --LPGQFKNI------------D--RADY---------VLVNSFDS--LEA--E-LRKEM
U5DDJ3      195 --LSGQFENI------------D--QADY---------VLVNSFDS--LEA--E-VRKEI
U5DFQ3      195 --LSGQFKNI------------D--QADY---------VLVNSFDS--LEA--E-VRKEI
U5DI97      195 --LLGQFKNI------------E--KADY---------VLMNSFDS--LES--K-VSEET
U5DCP2      196 --LLGQFKNI------------D--KADY---------VLLNSFDC--LET--G-VIEGM
U5DDK2      196 --LLGQFKNI------------D--KADY---------VLLNSFDC--LEA--G-VIEGM
OsUGT709A3  216 --LLGRLADA----------AVHCSKARA---------LILNTAAS--LEA--P-ALAHI
SgUGT720A1  212 --VDQTTASS---------------RSSG---------LILNTFDD--LEA--P-FLGRL
U5CX27       97 --CTIESPNT---------------LKSW--------AIILNTFED--LDK--P-VLDAM
U5D008      114 --LQNCTVGC-----------PEALKSSA---------LIINTFEE--LEK--S-VLDAI
W1PMY7      122 --IKDEVQHA------------F--NASA---------LILNTFDD--LEK--D-VLNAI
AtUGT85A1   218 --ALRETERA------------K--RASA---------IILNTFDD--LEH--D-VVHAM
U5CVP6      214 --LKDEALAS------------L--KGSA---------LILNTFDA--FEQ--T-ALNLV
U5CNJ5      213 --CTVESQLA------------T--TASA---------MIINTFDH--LEA--T-ILKIL
U5CNG3      215 --LKEPVIAI---------------KSTA---------VILNTFDS--LEQ--P-VLDAM
U5CXJ9      215 --NEEPLIAI---------------KSTA---------VILNTFDS--LEQ--P-VLDTM
AtUGT76B1   192 --VMKSLK-----------------SSSG---------IIFNAIED--LET--D-QLDEA
W1PV81      195 --ISQLTKQT------------K--KASA---------LILNSLSA--LEN--E-ALKMI
W1PVY0      192 --FISSFTGK-----------SF--RASA---------LIFNTSDC--LES--P-ALEKI
W1PX56      225 --FISSFTSK-----------SF--RASA---------LIFNTSDS--LES--P-ALEKI
AtUGT90A1   209 --SMDQIKST------------T--TSHG---------FLVNSFYE--LES--A-FVDYN
OsUGT97A1   227 --LHDEVRDW-----------DH--RSWG---------VLVNSFAA--LDGDYAAILESF
W1PH20      209 --CEVGLADI---------------ESDG---------VLVNTFYE--LEP--A-WVDCF
W1PBB6      226 --MEAGVATF---------------RSYG---------EILNSFYE--LEP--E-FVEAL
W1PHN8      226 --LETGAAKL---------------RSYG---------EILNSFYE--LEP--E-FVKTL
W1PRF4      190 --FQANSNAL------------P--KATG---------IILNTFRA--FET--D-ALSAL
AtUGT71B1   192 --VLGRARSF------------R--ATKG---------ILVNSVAD--MEP--Q-ALSFF
W1P1M0      207 -------------------------SNKN-------------------LER--QEAWGAI
AtUGT72B1   197 --LLHNTKRY------------K--EAEG---------ILVNTFFE--LEP--N-AIKAL
W1PMA9      201 --LLHQAKRY------------R--QADG---------IFVNTFEG--LES--Q-ALQFL
W1PP20      199 --FLHQAKRY------------P--RADG---------IFVNTFEA--LES--Q-ALQFL
AtUGT88A1   199 --FIMFGKQL------------S--KSSG---------IIINTFDA--LEN--R-AIKAI
U5DB27      161 -------------------------AYTW---------FLYTPAPS---------CLPGH
U5DBY1      195 --FLYHSRRL------------R--EVEG---------ILVNTFEE--FEP--Q-PLKAI
U5DBY4      204 --FLYNSRRF------------H--QAKG---------ILINTFLD--LET--R-SLDAL
U5D276      203 --FLYHAGRL------------R--ESKG---------IIINTFAE--LEP--L-TLKAL
U5DB32      203 --FLYHCRRL------------R--ESKG---------IIVNTFAE--LEP--L-TLKAL
W1NHX5      196 --FVKACNRI------------A--LTQG---------ILVNSFDE--LEP--PRALRAL
W1NVU7      181 --------------------------------------RVLEALAS-------GECTPDV
W1PAP0      195 --CLHHASRL------------P--RGRG---------IIINSFHD--LEP--L-GMRAL
W1PD80      191 --FLYHSAKV------------P--QGKG---------ILLNSFEE--LEPLGLKALQAL
W1PZW0      194 --CMYYASTM------------P--KADG---------ILVNTFEQ--LEP--L-ALKAL
AtUGT82A1   195 --WQRTLERT------------K--SLRW---------ILTSSFKD-EYEDVDN-HKASY
W1NUV1      334 --ILAAFNGA------------K--RADW---------VLSNTVEE--LEL--H-TIEAL
AtUGT86A1   217 --LFKAFKDV------------K--RADF---------VVCNTVQE--LEP--D-SLSAL
AtUGT87A1   200 --FKKSFGEL------------Y--KAKY---------LLFPSAYE--LEP--K-AIDFF
W1P188      209 --FFHQIQNA------------KS--------------ILFNSFYE--LEP--S-AIDAL
W1P188        1 ------------------------------------------------------------
```

```
U5CYW0      230 G---------------------LPIRP---SFCRPLLSKEELREELEMDMA-----LPAV
W1PAZ2      297 K-------------PSQIRVYGLPVRP---SFVNPVRPKEELRREFGMDEE-----LPAV
AtUGT81A1   312 E-------------TSQIKVYGLPVRP---SFVKPVRPKVELRRELGMDEN-----LPAV
W1PTT2      272 S-------------------SESFDYI-----EGFVFTNSDHPVNGCH--------SVPL
U5D3V5      271 P------------------SSSHHVFDYIEGFVACIGDDPIDGWDTI---------PPNT
W1NUG7      272 H------------------GRTKFDYV-----EGFVIVDEGLINNW----------RSSF
W1PC48      262 E--------------------KGFDYV-----EGFVIINRNG--------------ILNN
W1PP63      252 A--------------------DKVDYV-----EGFMVLNEQ---------------SLEG
OsUGT701A2  266 P--------------------EQVDYV-----EGFMVLNEQSLHSS----------SVAF
W1PA48      248 H--DWPASPLVLYGFSKEVVECPDYWPQSTRICGFWFPPMEWELPYKKCGDFSFTSLENL
U5D0T8      271 Q-------------GCTSDIPTGYIWS-----PHLVPKPKDWGPKVDCVGFCFLDLATNY
AtUGT80A2   390 Q-------------GSGSNIPHGYMWS-----PHLVPKPKDWGPQIDVVGFCYLDLASNY
W1PJ19      185 E--KQLGNGYKENRREHEYNKYKNIFT-----VGIQAPRPGDEPPLVSGGEGG---AMKP
W1NU70      234 EKEFQSHANRESSDRFYKNIGNLNFLP-----VGFLSPRLGDEAPRDNSTGID---GGSS
W1NV64      230 VKEYLNYFKECGG------SGVKKFLP-----IGFLPPRPGDEAHRD---------NGTM
W1PKS4      232 Q----ENYQSYFKECRGGGDGDKKFLP-----IGFLPPRLG---------------GEAQ
AtUGT91A1   241 Q-----------------ELHRKPVIP-----VGVLPPKPDEKFE-----------DTDT
U5CMK2      233 R-----------------ELYGKPVLP-----FGLVPSPPVEK-------------RGAG
U5CWM7      225 E-----------------KLYEKSVIP-----IGFRPPARKEREG-----------SGNS
U5D1X9      235 E-----------------KLYEKSVFP-----LGFLPPSQAEREG-----------NDNS
W1PT00      220 S-----------------ALAGKRVIP-----VGPLVSS-----------------RTPA
OsUGT94A1   231 S-----------------KLVGKEIIP-----CGPLLVDS----------------GDVS
AtUGT79B1   237 S-----------------RQYSKPVYL-----TGPVLPGSQPNQPS----------LDPQ
W1PQK6      241 A-----------------AQHKKPILL-----AGPAIPGPNQQRIGIAA-------LDNE
U5D602      219 P---------------------HKAWA-----VGPILPLS----------------PRPQ
U5D5X9      196 S----------------QRSLGLHVWA-----VGRTLLLR----------------EEPQ
U5D5Z2      251 A--------------------SSHVWA-----IGPTVLLQK---------------KPQE
U5D5X3      176 A----------------QRSPGLHVWA---------------------------------
ZmUGT93B1   221 A--------------AQPPFQGQRFFA-----VGPLNPLLLDADA-----------PTTP
PoUGT95A1   280 A-----------------EQTKLPVWG-----VGPLLPEQFWKSAGELLHDHEMRSNHKS
U5D0B7      287 A-----------------DVAGKPVWG-----VGPLLPSSFWDWTHGEFHDSAVRATRDS
AtUGT89A1   235 K----------------QKVSENRVFG-----VGPLSSVGLSK-------------EDSV
OsUGT96A1   230 K----------------RFFGHGRVWA-----VGPVADSGC---------------RGEE
AtUGT92A1   243 R-----------------RITGVPVWP-----VGPVLKSPDKK-------------VGSR
U5CRD3      203 R-----------------KTTKTSVWA-----VGPLLPRSLINPNFMEKGT-----DEHV
W1P7W1      242 R----------------DASVGSVVFA-----MGPIMRRPSLAH------------WGRD
U5D7I9      235 Q-----------------TMTGSRVLP-----IGPLLPLSLFSENWARER------GGKP
W1PFP0      235 Q-----------------AMTGRRVLP-----IGPLLPLSLFSENWARER------GGKP
OsUGT99A1   235 A-----------------EARGMKLWT-----VGPVALYHRSTATLAA--------RGNT
AtUGT73B1   241 K-----------------SFVAKRAWH-----IGPLSLGNRKFEEKAE--------RGKK
OsUGT98B1   245 E-----------------RTIGKKIWT-----IGPMCLCHRDSNAMAA--------RGNK
W1PF90      257 R-------------------KTTRAWH-----VGPVSMVTPD--------------TGSR
W1NIK4      242 I-----------------NVMGRKAWP-----IGPVSLYNRDQADKAE--------RGMK
W1PQX3      246 K-----------------NMEGTKAWP-----IGPVSLCNRDQIDKAA--------RGKK
W1PPW8      245 R-----------------NVVGMKAWP-----IGPVSLCNRDQIDKAE--------RGKK
W1PS80      245 R-----------------NVVGMKAWP-----IGPVSLCNRDQIDKAE--------RGKK
AtUGT90A1   239 N----------------NSGDKPKSWC-----VGPLCLTDPPKQGS----------AKPA
OsUGT97A1   261 Y------------------HPGSRAWL-----VGPLFLAAGESPET----------KQEE
W1PH20      238 E---------------GFYKKGSRAWC-----VGPLCLYS----------------RSPQ
W1PBB6      255 N------------------TQGRRYWC-----VGPMCLWDEPK-------------LVER
W1PHN8      255 N------------------TQGQRYWC-----VGPMCLWDEPK-------------PVEI
W1PRF4      220 A----------------MQAHVPPIFP-----IGPLPPDPLGPSA---------------
AtUGT71B1   222 S-------------GGNGNTNIPPVYA-----VGPIMDL-----------------ESSG
W1P1M0      221 S--------------------VPKIVP-----VGPL--------------------TLDL
AtUGT72B1   227 Q---------------EPGLDKPPVYP-----VGPLVNIG----------------KQEA
W1PMA9      231 K---------------ENRASFPEVFP-----VGPL--------------------TLEL
W1PP20      229 K---------------ENRASFPEVFP-----VGPLTLE---------------------
AtUGT88A1   229 T----------------EELCFRNIYP-----IGPLIV------------------NGRI
U5DB27      178 P--------------------TPPVYA-----VGPLLNLD----------------EQQP
U5DBY1      225 R-----------DGQCLPDHPTPPVYA-----VGPLLNLD----------------EQQP
U5DBY4      234 H-----------KGICLSDHPSPPVYP-----VGPLLNLD----------------EYQT
U5D276      233 R-----------EGLCLPNHPTPPIYT-----VGPLLNL-----------------DDQP
U5DB32      233 R-----------EGLCLPEHPMPTIYT-----VGPLLNLHDLP-----------------
W1NHX5      227 A-----------FGECTPDVPTPPVYC-----IGPLIA------------------ASTS
W1NVU7      196 P--------------------TPPVYC-----LGPLLLTPA---------------GGNR
W1PAP0      225 A-----------FEECAPGVPMPLTYC-----VGPLIAA-----------------GDSR
W1PD80      224 M------------DHTHGGVPRPPVYW-----IGPLV-------------------GGED
W1PZW0      224 A-----------ANEFRPGVPMPKIYC-----VGPLITV-----------------GDAD
AtUGT82A1   228 K--------KSNDLNKENNGQNPQILH-----LGPLHNQEATNNITIT--------KTSF
W1NUV1      364 N-------------------NYFPFYA-----VGPILPPKFTENSI----------STSL
AtUGT86A1   247 Q-------------------AKQPVYA-----IGPVFSTDSVV-------------PTSL
AtUGT87A1   230 T-----------------SKFDFPVYS-----TGPLIPLEE---------------LSVG
W1P188      236 S-----------------SLVPRPIFP-----VGPLIPFPAP--------------KDHE
W1P188        1 ------------------------------------------------------------
```

```
W1P302      240 H----------------ALLPRGHIFP-----VGPLIPLP----------------QENE
AtUGT78D1   231 L-----------------RSKLKRFLN-----IAPLTLLSS---------------TSEK
W1PTN8      231 R------------------ANMAPCFT-----IGPLPPIP----------------SPNA
ZmUGT77A1   246 A------------------EILPNCVP-----FGPYHLLLAEDD------------ADTA
AtUGT83A1   244 G---------------------PNIVP-----IGPIGWAHSLEEGST---------SLGS
W1NWE7      119 P----------------------NLLP-----IGPLQSNHHGQ-------------PTSL
W1NXD5      237 P----------------------NIRP-----IGPLFIGNQPTQAK----------PTSF
AtUGT75B1   217 P--------------------NIDMVA-----VGPLLPTEIFSGS-----------TNKS
AtUGT84A1   238 S-------------------SLCPVKT-----VGPLFKVARTVTSDV---------SGDI
W1PLB6      230 E-------------------ELAPIRP-----IGPLVPSELLGQSTDTEV------RGDL
U5CXN6      231 S-------------------DLTPIIP-----VGPLVPMELLGKSTAVEA------GGDL
U5D2S8      221 S-------------------ELTPIMP-----VGPLVPSKLLGKSTASEM------QGDL
U5D817      228 S-------------------ELTPIMT-----IGPLVPLGLLERSTASEV------RGDL
W1NFL7      228 S-------------------ELTPIMT-----IGPLVPLGLLGRSTASEV------RGDL
W1NFN9      228 S-------------------ELTPIMT-----IGPLVPLGLLRRSTASEM------RGDL
U5DBW3      228 H----------------------LMKL-----VGPLIPQALLGCPTAPRL------RKDM
W1PE57      227 H----------------------PIRL-----VGPLVPQALLERSQPAAISDL---RRDM
U5D3G1      218 E------------------KENMTLLS-----IGPLVPSAFIDGKNPEDTTF----GGDM
AtUGT74B1   226 S-------------------DAMKATL-----IGPMIPSAYLDDRMEDDKDY----GASL
U5DFQ6      230 G--------------------RWPIKT-----IGPMVPSAFTDTMDPSDRSF----GLSL
U5DI88      257 A-------------------KGLPIKM-----IGPAVPSAYLDNRIPDDK------GPTA
U5DCN3      226 A-------------------KAFPLKM-----IGPAVPSVYLDNMIADDK------GSDA
U5D531      186 H--------------------QSLKWT-----------------------------RDEQ
U5CUG8      222 V----------------------EPWS-----------------------------LKMI
W1PLP3      222 V-----------------------------------------------------------
U5DI92      225 A----------------------EPWS--PKLIGPALPSVYIGSRNP---------RASK
U5D3V6      223 A----------------------EPWS--LKMIGPSLPSVYLGSRTP---------RVTK
U5DDJ3      225 A----------------------EAWS--LMMIGPALPAVYLGSKNP---------RATK
U5DFQ3      225 A----------------------ESWS--LMMIGPALPLVYLGSKNP---------RATK
U5DI97      225 A----------------------EPWS--PKMIGPALPLAYLVGRNA---------RSTS
U5DCP2      226 A----------------------EPWS--PKMIGPALPSLYLDSRNT---------RATK
U5DDK2      226 A----------------------EPWS--PKMIGPALPSLYLDSRNT---------RTTK
OsUGT709A3  250 A------------------PRMRDVFA-----VGPLHAMSPAPAA-----------ATSL
SgUGT720A1  241 S------------------TIFKKIYA-----VGPIHSLLNSH-------------HCGL
U5CX27      127 R-----------------ERIGRPIYT-----IGPVVSFSQFESNDRLKSV-----SMSL
U5D008      147 R-----------------ERTHCPIYT-----IGPIASFSQFESKDCLKS------VSMS
W1PMY7      152 K-----------------ERIPCPLYT-----LGPLTFSHDVTKEEVKPI------STSL
AtUGT85A1   248 Q------------------SILPPVYS-----VGPLHLLANREIEEGSEIGMM---SSNL
U5CVP6      244 R------------------PIAPPMYT-----IGPLPTLCHTLVDPKLLSI-----GSSL
U5CNJ5      243 C------------------SRVPSIYT-----IGPLHTLSRKISDPKLHSI-----ESNL
U5CNG3      244 R------------------AKVPALYT-----VGQLHMLCKRAITEPKLGSI----GCSL
U5CXJ9      244 R------------------AKVPTLYT-----VGQLSMLCKRAITDSKLSSI----DSSL
AtUGT76B1   219 R-----------------IEFPVPLFC-----IGPFHRYVSAS-------------SSSL
W1PV81      225 Q-----------------HDFHIPIYA-----IGPLNCQ-----------------ISLN
W1PVY0      223 R-----------------PQCRGPVFA-----VGPLNFP-----------------SSSS
W1PX56      256 R-----------------PQCRGPVYA-----IGPLNLPS----------------SSSI
AtUGT90A1   239 N----------------NSGDKPKSWC-----VGPLCLTDPPKQGS----------AKPA
OsUGT97A1   261 Y------------------HPGSRAWL-----VGPLFLAAGESPET----------KQEE
W1PH20      238 E---------------GFYKKGSRAWC-----VGPLCLYS----------------RSPQ
W1PBB6      255 N------------------TQGRRYWC-----VGPMCLWDEPK-------------LVER
W1PHN8      255 N------------------TQGQRYWC-----VGPMCLWDEPK-------------PVEI
W1PRF4      220 A----------------MQAHVPPIFP-----IGPLPPDPLGPSA---------------
AtUGT71B1   222 S-------------GGNGNTNIPPVYA-----VGPIMDL-----------------ESSG
W1P1M0      221 S--------------------VPKIVP-----VGPL--------------------TLDL
AtUGT72B1   227 Q---------------EPGLDKPPVYP-----VGPLVNIG----------------KQEA
W1PMA9      231 K---------------ENRASFPEVFP-----VGPL--------------------TLEL
W1PP20      229 K---------------ENRASFPEVFP-----VGPLTLE---------------------
AtUGT88A1   229 T----------------EELCFRNIYP-----IGPLIV------------------NGRI
U5DB27      178 P--------------------TPPVYA-----VGPLLNLD----------------EQQP
U5DBY1      225 R-----------DGQCLPDHPTPPVYA-----VGPLLNLD----------------EQQP
U5DBY4      234 H-----------KGICLSDHPSPPVYP-----VGPLLNLD----------------EYQT
U5D276      233 R-----------EGLCLPNHPTPPIYT-----VGPLLNL-----------------DDQP
U5DB32      233 R-----------EGLCLPEHPMPTIYT-----VGPLLNLHDLP-----------------
W1NHX5      227 A-----------FGECTPDVPTPPVYC-----IGPLIA------------------ASTS
W1NVU7      196 P--------------------TPPVYC-----LGPLLLTPA---------------GGNR
W1PAP0      225 A-----------FEECAPGVPMPLTYC-----VGPLIAA-----------------GDSR
W1PD80      224 M------------DHTHGGVPRPPVYW-----IGPLV-------------------GGED
W1PZW0      224 A-----------ANEFRPGVPMPKIYC-----VGPLITV-----------------GDAD
AtUGT82A1   228 K--------KSNDLNKENNGQNPQILH-----LGPLHNQEATNNITIT--------KTSF
W1NUV1      364 N-------------------NYFPFYA-----VGPILPPKFTENSI----------STSL
AtUGT86A1   247 Q-------------------AKQPVYA-----IGPVFSTDSVV-------------PTSL
AtUGT87A1   230 T-----------------SKFDFPVYS-----TGPLIPLEE---------------LSVG
W1P188      236 S-----------------SLVPRPIFP-----VGPLIPFPAP--------------KHDE
W1P188        1 ------------------------------------------------------------
```

U5CYW0	261	LLMGGGE---GM-----GPVKKTAKALGDALFDQSLSRPIGQI-------VIICGRNQ-M
W1PAZ2	336	LLMGGGE---GM-----GPIEATARALGDLLYDENMGGPIGQV-------LIICGRNK-K
AtUGT81A1	351	LLMGGGE---GM-----GPIEATARALADALYDKNLGEAVGQV-------LIICGRNK-K
W1PTT2	300	LPHQTFD------------LNLIPANAGPVLYCLEIALHFANR-------DDAKTIEK-R
U5D3V5	304	MITHHTYDHHHA-----AWNSSSS---STVRYCLELAINYNTADSTHDIDKKMKAMVG-S
W1NUG7	299	FSPRNPI---KL-----QSIVSGSK--GTVLY--CLEV-TKNY----DE-ATADTVDQ-E
W1PC48	283	WRSSFFTPENPI-----QASRFNSN--GRTLYCLEIAK-NFDF-------NNANATDQ-E
W1PP63	272	ASVAFPS---HF-----HFIASNTNNTSQVYYCVEIAFYYYSQ----KA-SHVEKVAK--
OsUGT701A2	291	PAQLNFS----------PDFGSKGR--KKVYYCIEFA--VHDFQQDSSRADHVVKLVS-A
W1PA48	306	CIKVGSW---MVPTALCNFLKGTPNNFSAPIF-IGLNS-IGRMGFMKNP-QAFLELLKAV
U5D0T8	313	KPPESLE----------KFIGDPGD--KKPIY-IGFGS-LPVQ----EP-EKMTQIIVEA
AtUGT80A2	432	EPPAELV----------EWLEAG----DKPIY-IGFGS-LPVQ----EP-EKMTEIIVEA
W1PJ19	235	WVE-------AL-----KWLDGQPD--RSVTY-VAFGS-DFVL----TA-AQIEAVAL-G
W1NU70	286	VEKSWTE---CL-----DWLDKQPF--KSVVY-VAFGT-EWTL----TP-SQTRAVAL-G
W1NV64	270	DSGSCQK---FVKKPWAKWLDKQPV--SSVTY-VAFGT-ECLL----NQ-SQTNAVAI-G
W1PKS4	268	WSNDSGSCQKCM-----DWLDKQPI--RSVTY-VAFGT-ECRL----NQ-AQTNAVAI-G
AtUGT91A1	268	WLSVK------------KWLDSRKS--KSIVY-VAFGS-EAKP----SQ-TELNEIAL-G
U5CMK2	258	EGGNGNEYSRIF-----SWLDEKAK--GSIVF-VGFGT-ETRL----SM-DEVHEMAF-G
U5CWM7	252	WNDENKWDI-AF-----HWLDAQPL--RSVVF-VGFGS---------GA-LEVQDGSG--
U5D1X9	262	WIDENKWST-AF-----EWLDAQPL--RSVVF-VGFGS-ECRL----SI-EQVHEIAY-G
W1PT00	241	TTDEFFR----------QWLDARPE--KSVVF-TSFGS-EYFM----SE-DEIKEIAL-G
OsUGT94A1	253	AGSEADG---VM-----RWLDGQEP--GSVVL-VSFGS-EYFM----TE-KQLAEMAR-G
AtUGT79B1	265	WA---------------EWLAKFNH--GSVVF-CAFGS-QPVV----NKIDQFQELCL-G
W1PQK6	272	WS---------------TWLSSHPA--GSVVF-CAFGS-ETFL----TE-KEIQELLL-G
U5D602	237	EQERQHG---CI-----QWLDKQAE--ESVLY-VSFGT-ISSL----SA-PQVRALAE-G
U5D5X9	219	EPHKFHE---CL-----QWLSKQAP--QSVLY-ISFGT-CVSM----CA-AEVRKLAP-R
U5D5Z2	271	PPHKLHE---CM-----QWLDKQAP--RSVLY-VSFGT-VVSM----SA-AEVRELAQ-G
U5D5X3	187	-------------------LDEQAP--QSVVY-VAFGT---------TVSVEVRELAQ-G
ZmUGT93B1	251	PGQARHE---CL-----EWLDRQPP--ESVLY-VSFGT-TSCL----HA-DQVAELAA-A
PoUGT95A1	318	NYTEDE----VV-----QWLESKPR--ESVIY-ISFGS-EVGP----TI-EEYKELAK-A
U5D0B7	325	SVSEEE----VR-----HWLDNMPH--GSVLY-VAFGT-EVGP----TR-TELAELAV-A
AtUGT89A1	261	SNVDAKA---LL-----SWLDGCPD--DSVLY-ICFGS-QKVL----TK-EQCDDLAL-G
OsUGT96A1	254	RLPEAEQ---LF-----SWLDTCPA--RSVVY-VCFGS-MYKP----PP-AQAAALGA-A
AtUGT92A1	268	STEEAVK----------SWLDSKPD--HSVVY-VCFGS-MNSI----LQ-THMLELAM-A
U5CRD3	236	WREQGLETVVCV-----ERMDQYPP--ASILY-VSFGS-QFNI----SE-KQMKDLAL-G
W1P7W1	269	FGITPER---CS-----EWLDLHEP--RAVLF-VCFSA-HNKV----SA-WQMKELAL-G
U5D7I9	267	LGISAES---CI-----AWLDTQPL--RSVLY-VSFGS-QNTV----SR-WQLKELAL-G
W1PFP0	267	LGISAES---CI-----AWLDTQPL--RSVLY-VSFGS-QNTV----SG-WQLKELGL-G
OsUGT99A1	265	AAIGADE---CL-----RWLDGKEP--GSVVY-VSFGS-IVHP----EE-KQAVELGL-G
AtUGT73B1	271	ASIDEHE---CL-----KWLDSKKC--DSVIY-MAFGT-MSSF----KN-EQLIEIAA-G
OsUGT98B1	275	ASMDDAK---CL-----QWLDSKKP--GSVIF-VSFGS-LSST----DP-QQLVELGL-G
W1PF90	279	GKAGSVE---CL-----AWLDMFER--GTVVY-ACFGS-MCRF----ES-DQLAEIGL-G
W1NIK4	272	ASIDERV---CL-----SWLDSQKQ--GSVLY-VCFGS-ISHF----NP-AQLVEIAL-G
W1PQX3	276	ASVDKSA---CL-----RWLDTQKP--ESVVY-VCFGS-THKL----AP-TQLTEIGL-G
W1PPW8	275	APVDESA---CL-----RWLDSQKP--ESVVY-VCFGS-LCRL----AP-QQLTDIGL-G
W1PS80	275	ASVDESA---CL-----RWLDSQKP--ESVVY-VCFGS-LCRL----AP-QQLTDIGL-G
AtUGT90A1	268	WI---------------HWLDQKREEGRPVLY-VAFGT-QAEI----SN-KQLMELAF-G
OsUGT97A1	288	DDDDDDDPEGCV-----AWLDERAARPGSVVY-VSFGT-QAHL----PD-AQLDELAH-G
W1PH20	262	DALDSNP---CI-----DWLGRQEV--GSVVY-VSFGT-QVAV----SR-GQMREIAL-G
W1PBB6	279	PKTEQAE---CL-----DWLNTKEE--GSVLY-VAFGS-QARL----EE-AQLREIGT-G
W1PHN8	279	PKTERAE---CL-----DWLDMKDE--GSVLY-VAFGS-QARL----EE-AQMREIGA-G
W1PRF4	244	----------PM-----DWLDGQPN--GSVIY-VSFGS-RTTI----SK-NQVDELGA-G
AtUGT71B1	247	DEEKRKE---IL-----HWLKEQPT--KSVVF-LCFGS-MGGF----SE-EQAREIAV-A
W1P1M0	236	PDNPGEE---CL-----KWLDEQPL--NSVLF-VCFGS-GGTF----TH-EQMNEMAM-G
AtUGT72B1	251	KQTEESE---CL-----KWLDNQPL--GSVLY-VSFGS-GGTL----TC-EQLNELAL-G
W1PMA9	251	PENDSE----CL-----RWLDEQPP--GSVLF-VSFGS-GGTL----SC-GQYQNLAH-G
W1PP20	248	QPENDSD---CL-----RWLDEQPP--GSVVF-VSFGS-GGTL----SY-EQYQNLAH-G
AtUGT88A1	250	EDRNDNKAVSCL-----NWLDSQPE--KSVVF-LCFGS-LGLF----SK-EQVIEIAV-G
U5DB27	197	PASEPHE---CL-----KWLGTQPD--GSVLF-LCFGS-WGAF----EP-AQAKEIAL-G
U5DBY1	253	PASEPHE---CL-----KWLGTQPD--GSVLF-LCFGS-WGAF----EP-AQAKEIAL-G
U5DBY4	262	SAGEHHK---CL-----QWLDKQPQ--DSVVF-LCFGS-LGTL----SN-RQTKQVAL-G
U5D276	260	AGGEGHE---CL-----RWLDKQPH--GSVVF-LCFGS-LGAF----PA-HQTNEIAL-G
U5DB32	260	AGGERHQ---CL-----QWLDKQPH--GSVVF-LCFGS-LGAF----SA-HQTNEMAL-G
W1NHX5	253	GGDDDCQ---CL-----EWLRVQPA--ASVVY-LCFGS-QGTF----SP-PQIKEIAI-G
W1NVU7	216	YSSC------CM-----ESLDAQPA--GSVVY-LCFGS-QGTF----ST-AQIKEIAT-G
W1PAP0	252	NPSE------SL-----SWLDTQPA--GTVVF-LCFGS-MGRL----SA-AQLNEIAV-A
W1PD80	248	RSGGDND---TL-----QWLDSQPA--GSVVF-LSFGS-VGRF----SE-AQIREIAV-G
W1PZW0	251	RSSE------VL-----AWLDTQPE--GSVVF-LCFGS-VGRL----SA-TQIREIAI-G
AtUGT82A1	267	WEEDMS----CL-----GWLQEQNP--NSVIY-ISFGSWVSPI----GE-SNIQTLAL-A
W1NUV1	390	WPESD-----CS-----SWLDTHPD--RSVIY-ISFGS-YAHI----SK-EEIQEIAE-G
AtUGT86A1	270	WAESD-----CT-----EWLKGRPT--GSVLY-VSFGS-YAHV----GK-KEIVEIAH-G
AtUGT87A1	253	NENRELD---YF-----KWLDEQPE--SSVLY-ISQGS-FLSV----SE-AQMEEIVV-G
W1P188	260	LKTNARPELDCS-----SWLDSQPH--GSTLY-VSLGS-VMLV----PA-KQMEELAH-G
W1P188	1	--

```
W1P302      263 FKTNARPEVDCS-----SWLDSQPH--GSTLY-VSLGS-LVTV----PD-RQMMELAF-G
AtUGT78D1   254 EMRDPHG---CF-----AWMGKRSA--ASVAY-ISFGT-VMEP----PP-EELVAIAQ-G
W1PTN8      252 NPGARDE---CL-----AWLDDQSK--ASVLY-VSFGT-VKAL----EA-PEVVALAE-G
ZmUGT77A1   271 APADPHG---CL-----AWLGRQPA--RGVAY-VSFGT-VACP----RP-DELRELAA-G
AtUGT83A1   269 FLPHDRD---CL-----DWLDRQIP--GSVIY-VAFGS-FGVM----GN-PQLEELAI-G
W1NWE7      140 WTEDET----CL-----EWLNKQPE--CSVIY-VSFGS-LTLF----DK-CQLHELAL-A
W1NXD5      260 WTEDNT----CL-----EWLDQQPS--NSVIY-ISFGS-FTIF----DE-HQFLELTL-A
AtUGT75B1   241 VKDQSSS---YT-----LWLDSKTE--SSVIY-VSFGT-MVEL----SK-KQIEELAR-A
AtUGT84A1   265 CKSTDK----CL-----EWLDSRPK--SSVVY-ISFGT-VAYL----KQ-EQIEEIAH-G
W1PLB6      260 WKPAHD----CL-----DWLSTQPQ--GSVIY-VAFGS-VVTL----ST-KQIEELAW-G
U5CXN6      261 WKPEDD----CL-----DWLSHQPH--KSVIY-VAFGS-VVIL----SP-KQIEELAW-G
U5D2S8      251 WKPEDD----CL-----NWLSTQPR--EFVIY-VAFGS-VVSL----PP-KQIEELAW-G
U5D817      258 WKSEDD----CL-----DWLNTQPK--ESVIY-VSFGS-IVSL----SP-KQIEELAW-G
W1NFL7      258 WKSEDD----CL-----DWLNTQPK--ESVIY-VSFGS-IVSL----SP-KQIEELAW-G
W1NFN9      258 WKSEDD----CL-----DWLNTQPK--ESVIY-VSFGS-IVSL----SP-TQIEELAW-G
U5DBW3      255 WVAPSN----CL-----SWLDSKPE--GSVVY-VSLGS-VSVL----PP-KQTEEMAW-G
W1PE57      257 WDASPQ----CL-----TWLDSKKA--TSVVY-VSMGS-LLVM----PP-KQMEEMAW-G
U5D3G1      251 WEKSKD----YM-----EWLGSKPV--GSVVY-VSFGS-LRVL----SP-MQARNIAR-A
AtUGT74B1   258 LKPISKE---CM-----EWLETKQA--QSVAF-VSFGS-FGIL----FE-KQLAEVAI-A
U5DFQ6      261 WEEEE-----CL-----VWLDTKPI--GSVVY-VSFGS-LVGL----DE-TTMSEIAW-G
U5DI88      287 HLCKAVD---CL-----NWLDERPF--RSTVY-AAFGS-LVSM----AV-EQMEEIAW-G
U5DCN3      256 DLYKAVN---CL-----GWLDERPV--RSTVY-VSFGS-LVGM----PK-EQMEEIAW-G
U5D531      197 GDPAD-----CL-----NWLDKRPV--SSVVY-VPFGS-LAAL----PA-EQMEEVAE-A
U5CUG8      231 WPEHESHPADCL-----RWLDERPD--SSVVY-VSFGS-LATL----PA-EQMEEIAN-A
W1PLP3      223 ---------------------------------------------------EP-------W--
U5DI92      252 TSDEQGKPTDCL-----HWLDDRSA--GSVVY-VSFGS-LAAL----SP-EQMEEVAG-A
U5D3V6      250 TSDEQGKLIDCL-----HWLDKRPV--GSVVY-VSFGS-VAVL----SA-EKMEEIAG-T
U5DDJ3      252 MSGEQGMLTDCL-----QWLDGRPV--GSVVY-VSFGS-MAVL----SA-EQMEEVSG-A
U5DFQ3      252 ISGEQGMLTDCL-----QWLDGKPV--GSVVY-VSFGS-MAVL----SA-EQMEEVSG-A
U5DI97      252 TGDDQGRLVNCL-----SWLDERPV--GSVVY-VSFGS-IAAL----SV-EQMEEIAS-A
U5DCP2      253 TGDEQGKLVNCL-----GWVDERPD--GSVVY-VSFGS-LAAL----TV-EQMEEIGG-A
U5DDK2      253 TGDEQEKLVNCL-----GWVNERPD--GSVVY-VSFGS-LASL----IT-EQMEEIAS-A
OsUGT709A3  276 WRADDG----CM-----AWLDCQAD--RSVVY-VSLGS-LTVI----SP-EQFTEFLS-G
SgUGT720A1  265 WKEDHS----CL-----AWLDSRAA--KSVVF-VSFGS-LVKI----TS-RQLMEFWH-G
U5CX27      160 WNEEDT----CV-----GWLNDKEP--KSVVY-VNFGS-ATVA----TP-IQLNEFAW-G
U5D008      179 LGKEEDA---CV-----EWLNNKET--SSAFL-------------TP-YQLNEFAW-G
W1PMY7      185 WKEESE----CL-----DWLDSHQP--RSVIY-VNFGS-ITVL----TP-HQLNEFAW-G
AtUGT85A1   282 WKEEME----CL-----DWLDTKTQ--NSVIY-INFGS-ITVL----SV-KQLVEFAW-G
U5CVP6      276 WKEDTR----CL-----DWLGNQET--ASVVY-VNYGS-ITTM----TP-SQLVEFAW-G
U5CNJ5      275 WIEDTS----CL-----EWLDNKQP--QSVLY-VNFGS-ITVV----TS-KQLEEFAW-G
U5CNG3      277 WTPDTS----VL-----DWLDEKPA--GSVVY-VNFGS-ITIV----TP-QQLEEFAW-G
U5CXJ9      277 WTPDAS----VL-----DWLDNKPA--GSVVY-VNFGS-ITIV----TP-QQLEEFAW-G
AtUGT76B1   244 LAHDMT----CL-----SWLDKQAT--NSVIY-ASLGS-IASI----DE-SEFLEIAW-G
W1PV81      246 LLPEDSK---CL-----SWLDAQPP--KSVIY-TSFGS-LAGM----DS-SQVAEIAW-G
W1PVY0      244 ILPEDDK---CI-----SWLNTQPP--RSVVY-TSFGS-LASM----ES-SQVMEIAW-G
W1PX56      278 LLEDDK----CI-----SWLDTQPP--RSVVY-TSFGS-IAGL----DS-SQVMEIAW-G
AtUGT90A1   268 WI---------------HWLDQKREEGRPVLY-VAFGT-QAEI----SN-KQLMELAF-G
OsUGT97A1   288 DDDDDDDPEGCV-----AWLDERAARPGSVVY-VSFGT-QAHL----PD-AQLDELAH-G
W1PH20      262 DALDSNP---CI-----DWLGRQEV--GSVVY-VSFGT-QVAV----SR-GQMREIAL-G
W1PBB6      279 PKTEQAE---CL-----DWLNTKEE--GSVLY-VAFGS-QARL----EE-AQLREIGT-G
W1PHN8      279 PKTERAE---CL-----DWLDMKDE--GSVLY-VAFGS-QARL----EE-AQMREIGA-G
W1PRF4      244 ----------PM-----DWLDGQPN--GSVIY-VSFGS-RTTI----SK-NQVDELGA-G
AtUGT71B1   247 DEEKRKE---IL-----HWLKEQPT--KSVVF-LCFGS-MGGF----SE-EQAREIAV-A
W1P1M0      236 PDNPGEE---CL-----KWLDEQPL--NSVLF-VCFGS-GGTF----TH-EQMNEMAM-G
AtUGT72B1   251 KQTEESE---CL-----KWLDNQPL--GSVLY-VSFGS-GGTL----TC-EQLNELAL-G
W1PMA9      251 PENDSE----CL-----RWLDEQPP--GSVLF-VSFGS-GGTL----SC-GQYQNLAH-G
W1PP20      248 QPENDSD---CL-----RWLDEQPP--GSVVF-VSFGS-GGTL----SY-EQYQNLAH-G
AtUGT88A1   250 EDRNDNKAVSCL-----NWLDSQPE--KSVVF-LCFGS-LGLF----SK-EQVIEIAV-G
U5DB27      197 PASEPHE---CL-----KWLGTQPD--GSVLF-LCFGS-WGAF----EP-AQAKEIAL-G
U5DBY1      253 PASEPHE---CL-----KWLGTQPD--GSVLF-LCFGS-WGAF----EP-AQAKEIAL-G
U5DBY4      262 SAGEHHK---CL-----QWLDKQPQ--DSVVF-LCFGS-LGTL----SN-RQTKQVAL-G
U5D276      260 AGGEGHE---CL-----RWLDKQPH--GSVVF-LCFGS-LGAF----PA-HQTNEIAL-G
U5DB32      260 AGGERHQ---CL-----QWLDKQPH--GSVVF-LCFGS-LGAF----SA-HQTNEMAL-G
W1NHX5      253 GGDDDCQ---CL-----EWLRVQPA--ASVVY-LCFGS-QGTF----SP-PQIKEIAI-G
W1NVU7      216 YSSC------CM-----ESLDAQPA--GSVVY-LCFGS-QGTF----ST-AQIKEIAT-G
W1PAP0      252 NPSE------SL-----SWLDTQPA--GTVVF-LCFGS-MGRL----SA-AQLNEIAV-A
W1PD80      248 RSGGDND---TL-----QWLDSQPA--GSVVF-LSFGS-VGRF----SE-AQIREIAV-G
W1PZW0      251 RSSE------VL-----AWLDTQPE--GSVVF-LCFGS-VGRL----SA-TQIREIAI-G
AtUGT82A1   267 WEEDMS----CL-----GWLQEQNP--NSVIY-ISFGSWVSPI----GE-SNIQTLAL-A
W1NUV1      390 WPESD-----CS-----SWLDTHPD--RSVIY-ISFGS-YAHI----SK-EEIQEIAE-G
AtUGT86A1   270 WAESD-----CT-----EWLKGRPT--GSVLY-VSFGS-YAHV----GK-KEIVEIAH-G
AtUGT87A1   253 NENRELD---YF-----KWLDEQPE--SSVLY-ISQGS-FLSV----SE-AQMEEIVV-G
W1P188      260 LKTNARPELDCS-----SWLDSQPH--GSTLY-VSLGS-VMLV----PA-KQMEELAH-G
W1P188        1 -----------------------------------------------------------
```

```
U5CYW0      305 LASALQAFEW-KV--------------------------------------------PVK
W1PAZ2      380 LASKLQSINW-RI----------------------PVQIEG-------------------
AtUGT81A1   395 LQSKLSSLDW-KI----------------------PVQVKG-------------------
W1PTT2      340 VSQMLEPLKF---RRG-------------------LKFSCD--------------LTYYD
U5D3V5      355 LRFSG-GVVY-SVDVS-----------------------------------------YLD
W1NUG7      340 VDSLLKTLSF-IQGSV-------------------FTTDLP----------------YID
W1PC48      327 VESLLAQLSF-IPSTL-------------------FSSELP----------------YLD
W1PP63      317 ---ETLRKLD-CI-PS-------------------LVYETE--VS------------YFD
OsUGT701A2  336 KLSYLRPHVY-SVEVS-----------------------------------------YFD
W1PA48      360 LEASDYRAI--LLSAD-------------------YEPLND--AIMNMLGSDNNGCDRLS
U5D0T8      354 LEITEQRGII-------------------------NKGWGG-------------------
AtUGT80A2   471 LQRTKQRGII-------------------------NKGWGG-------------------
W1PJ19      273 LEASGAPFLW-IKRTP-------------------RHVELG-------------AGDGLP
W1NU70      328 LESSGARFLW-IKRTP-------------------RYAPSE-------------ASDGLP
W1NV64      317 LEQSGFRFLW-VGRAP-------------------VSGKLP-----------------LP
W1PKS4      313 LEESGFRFLW-VRRVP-------------------IDGLPE----------------KLP
AtUGT91A1   306 LELSGLPFFW-VLKTR-------------------RGPWDT-------------EPVELP
U5CMK2      303 LELSGLPFVW-LLKKP-------------------QEGCEG--------------MDLLP
U5CWM7      291 -----------ILSEG--------------------------------------------
U5D1X9      306 LELSKLPFVW-ALRKP-------------------FETHDG--LE-------------ILP
W1PT00      281 LELSNLPFIW-VIRVS-------------------PNEVIK------------EINEVLP
OsUGT94A1   295 LELSGAAFVW-VVRFP-------------------QQSPDG-----DEDDHGAAAARAMP
AtUGT79B1   301 LESTGFPFLV-AIKPP----------------------SGV--ST---------VEEALP
W1PQK6      307 IEMTGLPFAV-VLNFP-------------------SPHPIQ---------------SPMP
U5D602      279 LEASGQPFLW-VLRHA-------------------DKGDIL-------AMDDSEEPRSLP
U5D5X9      261 IGSQREALHL-GAKTR-------------------RQREHI-------------RRRRCG
U5D5Z2      313 LEASGQPFLW-VLRYA-------------------DKGDIF-------AGREADSPRGLP
U5D5X3      215 LEPSGQPFLW-VLRDA-------------------DKGDVF-------ARREAEGPQGLP
ZmUGT93B1   293 LKGSKQRFVW-VLRDA-------------------DRADIY-------AESGESRHAMFL
PoUGT95A1   359 LEESDQPFIW-VIQPG---------------SGKSGIPRSFLGPAAAHTDDSEEEEGYYP
U5D0B7      366 LEETHRPFIW-AIQRNAGRRMPPPPPGIAADVEELRPPPPG---IAAEGVEGVDNMSILP
AtUGT89A1   303 LEKSMTRFVW-VVKKD-----------------------------------------PIP
OsUGT96A1   296 LEASGARFVW-AVGAD---------------------------------------AAVLP
AtUGT92A1   308 LESSEKNFIW-VVRPP-------------------IGVEVK---------SEFDVKGYLP
U5CRD3      281 LEASGRPFIW-SIRPP--------------------------------------------
W1P7W1      311 LEASGHVFIW-AVTPS-------------------RENAGA----------VEFSSESLP
U5D7I9      309 LEASGAGFIW-VVRPP-------------------LEFPMT----------AEFRSEWLP
W1PFP0      309 LEASGAGFIW-VVRPP-------------------LEFPMT----------AEFRSEWLP
OsUGT99A1   307 LEASGHPFIW-VVRSP-------------------DRHGEA-------------ALAFLR
AtUGT73B1   313 LDMSGHDFVWVVNRKG-------------------SQVEKE---------------DWLP
OsUGT98B1   317 LEASKKPFIW-VIKAG-------------------KKFPEV------------EE--WLA
W1PF90      321 LEATGRPFIW-VVRET-------------------TEPLGS--------------ISGLD
W1NIK4      314 LEASRRPFIW-VVKGD-------------------AQQVDQ----------------WLP
W1PQX3      318 LEASGLPFIW-VVRGD-------------------TEQVDQ----------------WLP
W1PPW8      317 LEASGLPFIW-VLRGD-------------------REQVDQ----------------WLP
W1PS80      317 LEASGLPFIW-VLRGD-------------------REQVDQ----------------WLP
AtUGT90A1   305 LEDSKVNFLW-VTRKD-------------------VEEIIG-------------------
OsUGT97A1   335 LVDSGHAFLW-AIGRS----------------------GGE----------------WSP
W1PH20      304 LERSGAKYLW-VVRGE---------------------EGEE-------------------
W1PBB6      321 LEGSGVNFLW-VVKGE-------------------AKLDDG-------------------
W1PHN8      321 LEGSGLNFLW-VVRGE-------------------PKLDDG-------------------
W1PRF4      279 LEASGVRFLA-VV-KS-------------------KVVDEG--AQ---------------
AtUGT71B1   289 LERSGHRFLW-SLRRA--------------------SPVGN--KSNPPPGEFTNLEEILP
W1P1M0      278 LEASEQRFLW-VIRKP-------------------HQTSSS-ASFFSSTSNGEDPLAFLP
AtUGT72B1   293 LADSEQRFLW-VIRSP----------------------SGIANSSYFDSHSQTDPLTFLP
W1PMA9      292 LEKSGQRFLW-VIRTP-------------------EVTEGP--AAFFTVESKGDPKAYLP
W1PP20      290 LEKSGQRFLW-VIRTP-------------------ELTEGP--AAFFTVESKGDPKAYLP
AtUGT88A1   295 LEKSGQRFLW-VVRNP-------------------PELEKT----------ELDLKSLLP
U5DB27      239 LEQSGHRFLW-SLRGP-------------------PKEKFS-------GPTNADLEVLLP
U5DBY1      295 IEQSGHRFLW-SLRGR-------------------PKEKFS-------GPTDTDLDVLLP
U5DBY4      304 LEQSGHRFLW-SLRGP-------------------TSMDQL------FDTKDAELRDLLP
U5D276      302 LEQSGQRFLW-SLRTT-------------------SHDSEP-----------------LP
U5DB32      302 LKQSGQRFLW-SLRTT-------------------SHDSEP-----------------LS
W1NHX5      295 LENSRQRFLW-VIHST-------------------RLMHEP------------DLDALLP
W1NVU7      255 LENSRQRFLW-VIHST-------------------QHMLEP------------DINGLLP
W1PAP0      291 LERSEHRFLW-VVRSP-------------------PAEGEE----MGKRPPEPDLEALLP
W1PD80      290 LENSGQRFLW-VVRSP-------------------PREDND--RTKRLRPSEPDLEELLP
W1PZW0      290 LESQRQRFLW-VVRSP-------------------PTEDPS---RKFLPPADPDLDALLP
AtUGT82A1   309 LEASGRPFLW-ALNRV--------------------------------------WQEGLP
W1NUV1      430 LMLSGRSFVW-VLRPD-------------------IVSSDE--------------PEPLP
AtUGT86A1   310 LLLSGISFIW-VLRPD-------------------IVGSNV--------------PDFLP
AtUGT87A1   295 VREAGVKFFW-VARGG-----------------------------------------ELK
W1P188      305 LIESVTRFIW-VAREP-----------------------------------------SIN
W1P188        1 ------------------------------------------------------------
```

```
W1P302      308 LIESEIRFIW-VARDP----------------------TRN-------------------
AtUGT78D1   296 LESSKVPFVW-SLKEK-------------------NMV-------------------HLP
W1PTN8      294 LENSARPFLW-SMRLE----------------------QHE----------------LLP
ZmUGT77A1   313 LEDSGAPFLW-SL-RE-------------------DSWP------------------HLP
AtUGT83A1   311 LELTKRPVLW-VTGDQ------------------------------------------QP
W1NWE7      181 LENLGRPFLW-VARPD-------------------LIDKAG--------------VAYLE
W1NXD5      301 LENLGRPFLW-VARPD-------------------LVDKSG---------------AGYP
AtUGT75B1   283 LIEGKRPFLW-VITDK----------------SNRETKTEG--EE----------ETEIE
AtUGT84A1   306 VLKSGLSFLW-VIRPP-------------------PHDLKV-------------ETHVLP
W1PLB6      301 LSSSGRPFLW-AAKPV-------------------DSTSEF----------------QLP
U5CXN6      302 LAGGGRPFLW-VAKPF-------------------DRTSEF----------------RLP
U5D2S8      292 LASSGRPFLW-VGKPV-------------------ASTSKF----------------LLP
U5D817      299 LASSGRPFLW-VAKPV-------------------DSTSEF----------------LLP
W1NFL7      299 LASSGRPFLW-VAKPV-------------------DSTSEF----------------LLP
W1NFN9      299 LASSGRPFLW-VAKPV-------------------DSTSEF----------------LLP
U5DBW3      296 LKISRRAFIW-VVKPP-------------------ENSSET----------------GLP
W1PE57      298 LKASALPFLW-VAKPS-------------------ENSSNT---------------TGMP
U5D3G1      292 LSESRHSYLW-VVGSS-------------------EKALQN-------------------
AtUGT74B1   300 LQESDLNFLW-VIKEA-------------------HIA-------------------KLP
U5DFQ6      301 LLQNQKPFLW-VVRPP-------------------GVPQGY-------------MGNELP
U5DI88      329 LHASKRPFVW-VVKPP-------------------PIYLEG--------------GNLLP
U5DCN3      298 LHASKRFFVW-VVRTL-------------------PRHLEG--RN------------LLP
U5D531      237 LHSSKRPFLW-VVKLP-------------------YAYQKG--------------GNSLP
U5CUG8      276 LLSSNRPFLW-VVKAP-------------------YAHQKG--------------GNSLP
W1PLP3      226 ----SLKMIWPVVKAP-------------------YAHQKG--------------GNSLP
U5DI92      297 LRWCKWPFLW-VVRLP-------------------FANQKG--------------GNALP
U5D3V6      295 LHSIKWPFLW-VVRPP-------------------SAHQKG--------------GNSLP
U5DDJ3      297 LRRSKWAFLW-VVRPP-------------------SANEKA--------------GNSLP
U5DFQ3      297 LRWCKWPFLW-VVRPP-------------------SANEKG--GN------------SLP
U5DI97      297 LRSSKWPFLW-VVRIP-------------------FAHQQG--GN------------QLP
U5DCP2      298 LRSIKWPFLW-VVRPP-------------------YAYEQG--------------RNSLP
U5DDK2      298 LRSSKRPFLW-VVKPP-------------------RAHEQG--RN------------SLP
OsUGT709A3  317 LVAAGNPFLW-VLRPD-------------------MVTARR---------------RHAD
SgUGT720A1  306 LLNSGKSFLF-VL-RS-------------------DVVEGD------------DEKQVVK
U5CX27      201 LANSQRPFIW-VIRED-------------------LVFGKS---------------ATLE
U5D008      212 LANSQRPFIW-DIRKD-------------------LVVGES---------------AMLE
W1PMY7      226 LANSEKPFIW-IIRQG-------------------LVQGES---------------TTLS
AtUGT85A1   323 LAGSGKEFLW-VIRPD-------------------LVAGEE---------------AMVP
U5CVP6      317 LANSEAPFVW-IIRPD-------------------LVQGEA---------------ALLP
U5CNJ5      316 LANSKMPFLW-VIRPD-------------------LVREEG---------------ARLP
U5CNG3      318 LANSGLPFVW-IIRPD-------------------LVRGEG---------------AMIP
U5CXJ9      318 LANSGLPFVW-IIRPD-------------------LVRGEG---------------AMIP
AtUGT76B1   285 LRNSNQPFLW-VVRPG-------------------LIHGKE-------------WIEILP
W1PV81      288 LANSGCRFLW-VVRPG-------------------SVNGYD--------------AAELP
W1PVY0      286 LANSGRPFLW-VVRPG-------------------LINVLN--------------AACLP
W1PX56      319 LANSGRPFLW-VVRSD-------------------LVNGLN--------------TAGLP
AtUGT90A1   305 LEDSKVNFLW-VTRKD-------------------VEEIIG-------------------
OsUGT97A1   335 LVDSGHAFLW-AIGRS----------------------GGE----------------WSP
W1PH20      304 LERSGAKYLW-VVRGE---------------------EGEE-------------------
W1PBB6      321 LEGSGVNFLW-VVKGE-------------------AKLDDG-------------------
W1PHN8      321 LEGSGLNFLW-VVRGE-------------------PKLDDG-------------------
W1PRF4      279 LEASGVRFLA-VV-KS-------------------KVVDEG--AQ---------------
AtUGT71B1   289 LERSGHRFLW-SLRRA--------------------SPVGN--KSNPPPGEFTNLEEILP
W1P1M0      278 LEASEQRFLW-VIRKP-------------------HQTSSS-ASFFSSTSNGEDPLAFLP
AtUGT72B1   293 LADSEQRFLW-VIRSP----------------------SGIANSSYFDSHSQTDPLTFLP
W1PMA9      292 LEKSGQRFLW-VIRTP-------------------EVTEGP--AAFFTVESKGDPKAYLP
W1PP20      290 LEKSGQRFLW-VIRTP-------------------ELTEGP--AAFFTVESKGDPKAYLP
AtUGT88A1   295 LEKSGQRFLW-VVRNP-------------------PELEKT----------ELDLKSLLP
U5DB27      239 LEQSGHRFLW-SLRGP-------------------PKEKFS-------GPTNADLEVLLP
U5DBY1      295 IEQSGHRFLW-SLRGR-------------------PKEKFS-------GPTDTDLDVLLP
U5DBY4      304 LEQSGHRFLW-SLRGP-------------------TSMDQL------FDTKDAELRDLLP
U5D276      302 LEQSGQRFLW-SLRTT-------------------SHDSEP-----------------LP
U5DB32      302 LKQSGQRFLW-SLRTT-------------------SHDSEP-----------------LS
W1NHX5      295 LENSRQRFLW-VIHST-------------------RLMHEP------------DLDALLP
W1NVU7      255 LENSRQRFLW-VIHST-------------------QHMLEP------------DINGLLP
W1PAP0      291 LERSEHRFLW-VVRSP-------------------PAEGEE----MGKRPPEPDLEALLP
W1PD80      290 LENSGQRFLW-VVRSP-------------------PREDND--RTKRLRPSEPDLEELLP
W1PZW0      290 LESQRQRFLW-VVRSP-------------------PTEDPS---RKFLPPADPDLDALLP
AtUGT82A1   309 LEASGRPFLW-ALNRV--------------------------------------WQEGLP
W1NUV1      430 LMLSGRSFVW-VLRPD-------------------IVSSDE--------------PEPLP
AtUGT86A1   310 LLLSGISFIW-VLRPD-------------------IVGSNV--------------PDFLP
AtUGT87A1   295 VREAGVKFFW-VARGG-----------------------------------------ELK
W1P188      305 LIESVTRFIW-VAREP-----------------------------------------SIN
W1P188        1 ------------------------------------------------------------
```

```
U5CYW0       320 -IR-G---FETQM------------EKWM--------GACDCIITKAGPGTIAEALVRGL
W1PAZ2       398 --------FVTKM---EDCMGA----------------CDCIITKAGPGTIAEAMIRGL
AtUGT81A1    413 --------FITKM---EECMGA----------------CDCIITKAGPGTIAEAMIRGL
W1PTT2       364 --------FLQRVKSNEE-AAK-ANGTWDA-----PHPWLNLFISSKNIQEFDSKVFNKI
U5D3V5       372 --------FLSRVSYSEA-RAR-AIGTWDA-----PHPWLNILVPVSRIADFDRTIFKET
W1NUG7       364 --------FLDRV------HKA-ELKLRAKGLWDVPHPWLNLFIPKSKIADFDAGVFKGI
W1PC48       351 --------FLDRV------HAS-EVKLRAQGLWEVPHPWLNLLIPQSTIHDFAEEIFGKL
W1PP63       339 --------FLNRVSA-EE-ANLRARGLWE-----VAHPWLNMFVPRSQIKAFKDLLLQTI
OsUGT701A2   354 --------FLNRVRM-EE-ESLRSRGLWD-----VPHPWLNVFVPKHGITQFKGLLMDTV
W1PA48       397 -ED-GVLLFYDRL---FCFSGT------IPYSWLFSRCSVS--IHHGGSGSTAAALRAGI
U5D0T8       370 --------LGNSA---EK-KEFVYLLDNCPHDWLFLH--CAAVVHHGGAGTTAAGLKAAC
AtUGT80A2    487 ---------LGNL---KEPKDFVYLLDNVPHDWLF--PRCKAVVHHGGAGTTAAGLKASC
W1PJ19       300 -DR-----FEDRI---EG-RGL-VVTEWIPQMKVLEHPAVGVFFSHGGISSSIEGLASGQ
W1NU70       355 --E-G---FVDRV---HR-QGL-VFKEWVPQSMILAHPSVGVFFSHSGTSSVIEGLATGK
W1NV64       340 --D-G---FLERV---QG-RGL-IVEEWVPQAQILSHPSIGVFFSHSGTSSVIEGLTTGK
W1PKS4       337 -DD-----FLERV---QG-RGL-IVEEWVPQPNILSHPSIGVFFSHSGTSSVIEGLTAGK
AtUGT91A1    333 -EG-----FEERT---AD-RGM-VWRGWVEQLRTLSHDSIGLVLTHPGWGTIIEAIRFAK
U5CMK2       329 -QG-----FETRI---SN-RGV-VHVGWAPQAQLLAHPAIGGCLFHSGWGSVIEALQWGH
U5CWM7       296 --------FESRT---EG-RGI-VCIGWAPQQSIVAHPSIGGCLFHSGWGSIIEALTHGL
U5D1X9       332 --K-G---FEGRT---ER-QGI-ICIGWAPQQRIFAHPSIGGCLFHSGWGSIIEALSHGL
W1PT00       309 -LG-----FIERV---KE-RGL--VVGWAPQGEILRHVAIGGFLTHCGWGSCMESLGLES
OsUGT94A1    330 -PG-----FAPA--------RGL-VVEGWAPQRRVLSHRSCGAFLTHCGWSSVMESMSAGV
AtUGT79B1    327 --E-G---FKERV---QG-RGV-VFGGWIQQPLVLNHPSVGCFVSHCGFGSMWESLMSDC
W1PQK6       332 -EG-----FEERV---RG-RGV-VKTGWVQQRLILGHESVGGFLCHSGFGSLIEGVVSGC
U5D602       312 -DC-----FEERV---KG-RGF-IVRDWAPQLQILGHSSTAGFLSHCGWNSCMESIFMGV
U5D5X9       288 -RSSG---ATRRV---RG-EGA--REGVDGEGLGIPIGDTASPL--YRWNSCMESISMGV
U5D5Z2       346 --D-G---YEERV---QG-RGL-IVRDWAPQLEILHHPSTGGFLTHCGWNSCMESIAMGV
U5D5X3       248 -EG-----Y-------EG-RGL-IVRDWAPQLEILRHPSTGGFFTHCRWNSCVESFAMGI
ZmUGT93B1    326 -SE-----FTRET---EG-TGL-VITGWAPQLEILAHGATAAFMSHCGWNSTIESLSHGK
PoUGT95A1    403 -DG-----LDVTV---GN-RGL-IITGWAPQLLILSHPSTGGFLSHCGWNSTAEAIGRGV
U5D0B7       422 --E-G---FEQRV---GS-RGL-VIHGWAPQLMILNHTATGGFLSHCGWNSMVEAVCAGV
AtUGT89A1    321 --D-G---FEDRV---AG-RGM-VVRGWAPQVAMLSHVAVGGFLIHCGWNSVLEAMASGT
OsUGT96A1    316 -EG-----LEERT---AA-RGR-VVRGWAPQVEILRHAAVGAFLTHCGWNSTLEGVAAGV
AtUGT92A1    339 -EG-----FEERIT--RSERGL-VKKWAPQVDILSHKATCVFLSHCGWNSILESLSHGV
U5CRD3       296 -----------------LGL-------PKIAEFSSESTGCFLRHCGWNSVMESLSKGV
W1P7W1       341 --E-G---FEARVSG-RK-QGL-VVRGWVPQHTILSHPATGAFLNHCGWHSVLESLRQGV
U5D7I9       339 --E-G---FEERV---KD-RGL-IVKGWAPQLAILSHKGTGAFLSHCGWNSVLESLSQGV
W1PFP0       339 --E-G---FEERV---KD-RGL-IVKGWAPQLAILSHKGTGAFLSHCGWNSVLESLSQGV
OsUGT99A1    334 --E-----LEARVAP-AG-RGL-LIWGWAPQALILSHRAAGAFVTHCGWNSTLEAATAGL
AtUGT73B1    339 -EG-----FEEKT---KG-KGL-IIRGWAPQVLILEHKAIGGFLTHCGWNSLLEGVAAGL
OsUGT98B1    343 --D-G---FEERV---KD-RGM-IIRGWAPQMMILWHQAIGGFMTHCGWNSTLEGISAGV
W1PF90       347 ---G---FEERV---KD-RGL-VIRGWAAQVLILSHESVGAFITHGGWNSVLEGVSSGL
W1NIK4       338 --E-G---FEERV---QG-RGM-IIRGWAPQLLILSHAATGGFLTHCGWNSVLEGLSVGL
W1PQX3       342 --K-G---FEERV---KG-RGM-VIKGWAPQILILSHPATGGFVTHCGWNSTLEGLTAGL
W1PPW8       341 --E-G---FEERV---KG-RGM-VIKGWAPQILILSHPATGGFVTHCGWNSTLEGLTAGL
W1PS80       341 --E-G---FEERV---KG-RGM-VIKGWAPQILILSHPATGGFVTHCGWNSTLEGLTAGL
AtUGT90A1    326 --E-G---FNDRI---RE-SGM-IVRDWVDQWEILSHESVKGFLSHCGWNSAQESICVGV
OsUGT97A1    356 --------PVDA----GG-DGK-IVRGWVPQRRVLSHPAVGAFVTHAGWNSVLESLAAGL
W1PH20       323 --------FEERE------NGL-IARVWVPQVAVLCHVAIGGFMSHCGWNSVLESLVHGV
W1PBB6       342 --------FRDRV---KG-RGL-VIQGWVSQVEVLSHNAVGGFMSHCGWNSVLESLVFGV
W1PHN8       342 --------FGDRV---KG-RGL-VIRGWVPQVEVLSHNAVGGFMSHCGWNSLLESLVYGV
W1PRF4       301 ----G----LSVV---KG-RGI-VVDGWVDQGSVLGHQSVGGFVSHCGWNSVAEAALNGV
AtUGT71B1    326 --K-G---FLDRT---VE-IGK--IISWAPQVDVLNSPAIGAFVTHCGWNSILESLWFGV
W1P1M0       317 -LG-----FVSRI---QG-RGL-VIPDWAPQGRILAHSSIGGFMSHCGWNSTLESVKNGV
AtUGT72B1    330 --P-G---FLERT---KK-RGF-VIPFWAPQAQVLAHPSTGGFLTHCGWNSTLESVVSGI
W1PMA9       330 --Q-G---FLERT---KG-VGM-VVPSWAPQTRVLAHAATGGFLTHCGWNSTLESFQNGI
W1PP20       328 --E-G---FLERT---KG-VGM-VVPSWAPQTRVLAHAATGGFLTHCGWNSTLESIQNGI
AtUGT88A1    325 -EG-----FLSRT---ED-KGM-VVKSWAPQVPVLNHKAVGGFVTHCGWNSILEAVCAGV
U5DB27       272 -EG-----FIDRT---RE-RGM-VWPTWVPQVAVLAHPAVGGFVSHCGWNSILESVWHGV
U5DBY1       328 EG------FIDRT---RE-RGM-VWPTWVPQVAVLAHPAVGGFVSHCGWNSILESVWHGV
U5DBY4       338 -EG-----FLERT---HD-LGM-VWPKWAPQAAVLAHPAVGAFVSHCGWNSTLESVWFGV
U5D276       325 -EG-----FLERT---QG-YGM-VWPKWVPQVAVLTHPAVGGFVSHCGWNSTLESLCHGV
U5DB32       325 -EG-----FAERT---QE-YGM-VWPKWVPQVAVLAHPAVGGFVSHCGWNSTLESLWHGV
W1NHX5       323 -EG-----FLGRT---AE-FGM-VMTSWAPQVAVLNHPAVGGFVTHCGWNSALESVCAGV
W1NVU7       283 -EG-----FSGRT---SW-LGM-VVKSWAPQVAVLNHGAVGRFVTHRRWNSMLESVCAGG
W1PAP0       327 -EG-----FLKRT---AG-KGL-VVKTWAPQVAVLNHMAVGGFVTHCGWNSLLESVCAGV
W1PD80       328 -QG-----FLQRT---AD-KGL-VMKSWASQAAVLSCAAVGGFVSHCGWNSVLESVCAGV
W1PZW0       327 -EG-----FLERT---SG-RGM-VVKNWAPQVAVLRHSAVGGFVTHCGWNSILESICAGV
AtUGT82A1    330 --P-G---FVHRVTITKN-QGR--IVSWAPQLEVLRNDSVGCYVTHCGWNSTMEAVASSR
W1NUV1       456 -KD-----FQERT---RG-QGL--VVHWCRQREVLSHRAIGGFLTHCGWNSIMESVWCEV
AtUGT86A1    336 -AG-----FVDQA---QD-RGL--VVQWCCQMEVISNPAVGGFFTHCGWNSILESVWCGL
AtUGT87A1    313 --------LKEAL---EGSLGV--VVSWCDQLRVLCHAAIGGFWTHCGYNSTLEGICSGV
W1P188       323 --------LRDMC---TD-RGL--VVPWCSQSKVLRHPSVGGFLTHCGWNSTVEGVLTGV
W1P188         1 ------------------------------------------------------------
```

```
W1P302      326 --------LRDMC---KD-NGL--VVTWCSQSKVLRHPSIGGFLTHCGWNSTMEAVLAGV
AtUGT78D1   317 --K-G---FLDRT---RE-QGI--VVPWAPQVELLKHEAMGVNVTHCGWNSVLESVSAGV
W1PTN8      315 --D-G---FDARV---GQ-RGR--VVAWAPQAQVLGHDAVGAFLTHCGWNSVLESIIGGV
ZmUGT77A1   334 --P-G---FLDRA---AG-TGSGLVVPWAPQVAVLRHPSVGAFVTHAGWASVLEGLSSGV
AtUGT83A1   328 -IKLG-----------SD-RVK--VVRWAPQREVLSSGAIGCFVSHCGWNSTLEGAQNGI
W1NWE7      207 ----G---FLDRM---EV-HGK--VVGWCPQREVLTHPSIACFVTHCGWNSTIEGVSNGV
W1NXD5      326 --E-G---FLGRV---AA-YGK--VVGWCPQKKVLAHPSIACFVTHCGWNSTLEGLSNGV
AtUGT75B1   314 -KIAG---FRHEL---EE-VGM--IVSWCSQIEVLSHRAVGCFVTHCGWSSTLESLVLGV
AtUGT84A1   333 -QE-----LKESS---AKGKGM--IVDWCPQEQVLSHPSVACFVTHCGWNSTMESLSSGV
W1PLB6      325 --D-G---FLKVI---VG-RGK--LVKWCPQVEVLTHPSVACFVTHCGWNSTLETIAAGT
U5CXN6      326 -DE-----FLDAI---AG-RGK--VVKWCPQVEVLTHPSITCFLTHCGWNSTLETIAAGT
U5D2S8      316 --D-G---FLDAT---AG-RGK--VVKWCPQVEVLTHPAVACFVTHCGWNSTLETIAAGT
U5D817      323 --T-G---FLDAA---AG-RGK--IVKWCPQVEVLTHPSVACFVTHCGWNSTLETIAAGT
W1NFL7      323 --T-G---FLDAT---AG-RGK--IVKWCPQVEVLTHPSVSCFVTHCGWNSTLETIAAGT
W1NFN9      323 --T-G---FLDAT---AG-RGK--IVKWCPQVEVLTHPSVSCFVTHCGWNSTLETIAAGT
U5DBW3      320 --E-G---FLEGV---EG-QGI--VVPWCPQVEVLSHPSLACFLTHCGWNSTLETVAAGV
W1PE57      323 --E-G---FLEEV---QG-QGI--VVPWCPQAEVLSHPSIACFVTHCGWNSTLETITAGV
U5D3G1      313 --------IEIED---SE-RGL--VVGWCNQVELLAHPSIGCFVMHGGWNSTIESLVAGV
AtUGT74B1   321 --E-G---FVEST---KD-RAL--LVSWCNQLEVLAHESIGCFLTHCGWNSTLEGLSLGV
U5DFQ6      328 -VG-----FVDEV---KG-RGL--VVEWCPQLEVLSHQSVGCFVTHCGWNSTLEGLSLGV
U5DI88      355 --K-G---FSEAT---ID-QGI--VVPWCPQLEVLSHTATACFLTHCGWNSTLEGLSLGV
U5DCN3      324 --K-G---FSEAT---KD-RGL--VVSWCPQLEVLSHRAVACFWTHCGWNSTLEGLSLGV
U5D531      263 --E-G---FVEAT---SE-QGL--VVPWCPQLDVLAHRAVGCFVTHCGWNSTLEALSQGV
U5CUG8      302 --E-G---FVEAT---SE-QGL--VVPWCPQLHVLAHRGVGCFVTHCGWNSTLEGLSQGV
W1PLP3      249 --E-G---FVEAT---SE-QGL--VVPWCPQLDVLAHRGVGCFVTHCGWNSTLEGLSQGV
U5DI92      323 -QG-----FIEAT---SE-QGL--VVPWCPQLDVLVHRAVGCFVTHCGWNSTLEGLTQGV
U5D3V6      321 --E-G---FIDAT---SE-QGL--VVPWCPQLDVLAHRAVGCFVTHCGWNSTLEGLTQGV
U5DDJ3      323 --E-G---FMEAT---SE-QGL--VVPWCSQLDVLSHRAVGCFVTHCGWNSTLEGLTQGV
U5DFQ3      323 --E-G---FIEAT---SE-QGL--VVPWCPQLDVLAHRAVGCFVTHCGWNSTLEGLTQGV
U5DI97      323 --E-G---FLEAT---FQ-QGL--VVPWCPQLDVLAHRAVGCFVTHCGWNSTLEGLSQGV
U5DCP2      324 --D-G---FLDAT---SE-QGL--VVPWCPQLEVLAHRAIGCFVSHCGWNSTLEAISQGV
U5DDK2      324 --D-G---FLDAT---TE-QGL--VVPWCPQLDVLAHRAIGCFVTHCGWNSTLEAISQGV
OsUGT709A3  342 --------LQESVTAAAG-DSKARVVGWAPQRDVLRHRAVGCFLTHAGWNSTLEAAVEGV
SgUGT720A1  333 -EI-----YETKA---EG-KWL--VVGWAPQEKVLAHEAVGGFLTHSGWNSILESIAAGV
U5CX27      226 -KT-----FVEET---KE-RRL--ILSWCNQIKVLSHPAVGCFLTHSGWNSTIESISNGV
U5D008      237 -KE-----FVEAT---KE-CGL--ILSWCNQMKVLPHHAIGGFLTHNGWKSTLDSILNGV
W1PMY7      251 -HD-----FYDKT---KG-RGL--IASWCNQKKVLSHSSIGGFLTHSGWNSILESICSGV
AtUGT85A1   348 -PD-----FLMET---KD-RSM--LASWCPQEKVLSHPAIGGFLTHCGWNSILESLSCGV
U5CVP6      342 -KE-----FVEVT---KE-RGF--LASWCNQEKVLLHPAVGVFLTHCGWNSIMESVCGGI
U5CNJ5      341 -QG-----FAEET---KE-RGI--VATWCNQEQVLGHRAIGGFLTHSGWNSTMESICNGV
U5CNG3      343 -KA-----FVEDT---KE-RSL--LASWCDQERVLQHPAVGGFLTHSGWNSTIESLSGGV
U5CXJ9      343 -EA-----FVEDT---KE-RSL--LASWCDQERVLQHPAVGGFLTHSGWNSTIESLSGGV
AtUGT76B1   312 -KG-----FIENL---EG-RGK--IVKWAPQPEVLAHRATGGFLTHCGWNSTLEGICEAI
W1PV81      314 --S-G---FMDQV---GS-RGL--VVGWAPQKDVLAHQAVGAFWTHCGWNSTLESLCAAV
W1PVY0      312 --S-G---FTEQV----GLRGL--VVEWAPQKKVLAHGAVGAFWSHCGWNSTLESICAGV
W1PX56      345 --S-G---FMDQV---GS-CGL--VVEWAPQKKVLAHRAVGAFWTHCGWNSTLESICAGV
AtUGT90A1   326 --E-G---FNDRI---RE-SGM-IVRDWVDQWEILSHESVKGFLSHCGWNSAQESICVGV
OsUGT97A1   356 --------PVDA----GG-DGK-IVRGWVPQRRVLSHPAVGAFVTHAGWNSVLESLAAGL
W1PH20      323 --------FEERE------NGL-IARVWVPQVAVLCHVAIGGFMSHCGWNSVLESLVHGV
W1PBB6      342 --------FRDRV---KG-RGL-VIQGWVSQVEVLSHNAVGGFMSHCGWNSVLESLVFGV
W1PHN8      342 --------FGDRV---KG-RGL-VIRGWVPQVEVLSHNAVGGFMSHCGWNSLLESLVYGV
W1PRF4      301 ----G----LSVV---KG-RGI-VVDGWVDQGSVLGHQSVGGFVSHCGWNSVAEAALNGV
AtUGT71B1   326 --K-G---FLDRT---VE-IGK--IISWAPQVDVLNSPAIGAFVTHCGWNSILESLWFGV
W1P1M0      317 -LG-----FVSRI---QG-RGL-VIPDWAPQGRILAHSSIGGFMSHCGWNSTLESVKNGV
AtUGT72B1   330 --P-G---FLERT---KK-RGF-VIPFWAPQAQVLAHPSTGGFLTHCGWNSTLESVVSGI
W1PMA9      330 --Q-G---FLERT---KG-VGM-VVPSWAPQTRVLAHAATGGFLTHCGWNSTLESFQNGI
W1PP20      328 --E-G---FLERT---KG-VGM-VVPSWAPQTRVLAHAATGGFLTHCGWNSTLESIQNGI
AtUGT88A1   325 -EG-----FLSRT---ED-KGM-VVKSWAPQVPVLNHKAVGGFVTHCGWNSILEAVCAGV
U5DB27      272 -EG-----FIDRT---RE-RGM-VWPTWVPQVAVLAHPAVGGFVSHCGWNSILESVWHGV
U5DBY1      328 EG------FIDRT---RE-RGM-VWPTWVPQVAVLAHPAVGGFVSHCGWNSILESVWHGV
U5DBY4      338 -EG-----FLERT---HD-LGM-VWPKWAPQAAVLAHPAVGAFVSHCGWNSTLESVWFGV
U5D276      325 -EG-----FLERT---QG-YGM-VWPKWVPQVAVLTHPAVGGFVSHCGWNSTLESLCHGV
U5DB32      325 -EG-----FAERT---QE-YGM-VWPKWVPQVAVLAHPAVGGFVSHCGWNSTLESLWHGV
W1NHX5      323 -EG-----FLGRT---AE-FGM-VMTSWAPQVAVLNHPAVGGFVTHCGWNSALESVCAGV
W1NVU7      283 -EG-----FSGRT---SW-LGM-VVKSWAPQVAVLNHGAVGRFVTHRRWNSMLESVCAGG
W1PAP0      327 -EG-----FLKRT---AG-KGL-VVKTWAPQVAVLNHMAVGGFVTHCGWNSLLESVCAGV
W1PD80      328 -QG-----FLQRT---AD-KGL-VMKSWASQAAVLSCAAVGGFVSHCGWNSVLESVCAGV
W1PZW0      327 -EG-----FLERT---SG-RGM-VVKNWAPQVAVLRHSAVGGFVTHCGWNSILESICAGV
AtUGT82A1   330 --P-G---FVHRVTITKN-QGR--IVSWAPQLEVLRNDSVGCYVTHCGWNSTMEAVASSR
W1NUV1      456 -KD-----FQERT---RG-QGL--VVHWCRQREVLSHRAIGGFLTHCGWNSIMESVWCEV
AtUGT86A1   336 -AG-----FVDQA---QD-RGL--VVQWCCQMEVISNPAVGGFFTHCGWNSILESVWCGL
AtUGT87A1   313 --------LKEAL---EGSLGV--VVSWCDQLRVLCHAAIGGFWTHCGYNSTLEGICSGV
W1P188      323 --------LRDMC---TD-RGL--VVPWCSQSKVLRHPSVGGFLTHCGWNSTVEGVLTGV
W1P188        1 ------------------------------------------------------------
```

```
U5CYW0      355 -------PIILNDFIPGQEV----------GNVPY-VVENGAG-----------------
W1PAZ2      430 -------PIILNDYIAGQEA----------GNVPY-VVENGAG-----------------
AtUGT81A1   445 -------PIILNGYIAGQEA----------GNVPY-VVENGCG-----------------
W1PTT2      409 LKNGIGGSILVYPLIKSKWD-PRMSVVVPDNEIFYLVALLRFN--------------K--
U5D3V5      417 VRLGVGGPILVYPLNSNK------------------------------------------
W1NUG7      409 LGKKTSGPILIYPMNKNRWDEKTSAVTPDEDVFYL-VAFLRSA--------------LDN
W1PC48      396 LTDSSNGPILIYPLNKEKWD-NRTSAVTPNEDIFYLVAFLSSS-----------------
W1PP63      384 SPDHFEGPILIYPIRRDKWN---------GNTSAV-LPEDKEN-------------ILYI
OsUGT701A2  399 SADDFEGPILVYPLLTDKWD---------GNTSAV-VPAAPDG--------------V--
W1PA48      444 -------PQITCPFMLDQFY-------WAERMSWLGVAPEPLC-----------------
U5D0T8      416 -------PTTVVPFFGDQPF-------------------WGER--------------VHA
AtUGT80A2   533 -------PTTIVPFFGDQPF-------------------WGER--------------VHA
W1PJ19      349 -------RLVALPIQLDQGI----------NTRLV-AEELKVV--------------V--
W1NU70      404 -------RLVLLPLVFDQGL----------IARLV-ANEWKAA--------------I--
W1NV64      389 -------RLVLLPMILDQGL----------IARLV-ADEWRAA--------------L--
W1PKS4      386 -------RLVLLPMIIDQGL----------IARLV-ADEWRAA--------------L--
AtUGT91A1   382 -------PMAMLVFVYDQGL----------NARVI--EEKKIG-----------------
U5CMK2      378 -------TLIVLPFVFDQGL----------NAKLL--VEKGIA--------------L--
U5CWM7      343 -------ALIILPFIADQGL----------NARLL--EEKKLA--------------F--
U5D1X9      381 -------ALILLPFIIDQGI----------NARLL--GEKKLG--------------F--
W1PT00      357 -------PLITLPLQLDQPF----------NSRVM-TREVRVG--------------V--
OsUGT94A1   376 -------PMVALPLHIDQPV----------GANL--AAELGVA--------------A--
AtUGT79B1   376 -------QIVLVPQHGEQIL----------NARLM-TEEMEVA--------------V--
W1PQK6      381 -------QLVLLPLKGDQFL----------NARLM-GRELRVG--------------V--
U5D602      361 -------PIATWPMHSDQPA----------NSHLV-TKILGMG--------------V--
U5D5X9      336 -------PIVEWPMHSDQPA----------NAQLV-AGVLGVG--------------V--
U5D5Z2      395 -------PMVAWPMHSDQPV----------NAQLV-TGVLGVG--------------V--
U5D5X3      293 -------PIVTWPLHSDQPV----------NAQLV-TGVLGVG--------------V--
ZmUGT93B1   375 -------PVLAWPMHSDQPW----------DSELL-CKYFKAG--------------L--
PoUGT95A1   452 -------PILGWPIRGDQFD----------NAKLV-AYHLKIG--------------HVM
U5D0B7      471 -------PLLAWPIRGDQHY----------NAKMV-ASWLKIG-----------------
AtUGT89A1   370 -------MILAWPMEADQFV----------DARLV-VEHMGVA--------------V--
OsUGT96A1   365 -------PLLAWPMKADQFI----------DARLV-VDLRGAA--------------V--
AtUGT92A1   390 -------PLLGWPMAAEQFF----------NSILM-EKHIGVS--------------V--
U5CRD3      330 -------PIIGWPMGAEQFF----------NVKLL-VEEVGVC--------------I--
W1P7W1      392 -------PIIGWPMGADQFY----------ISKEL-EEELGVG--------------L--
U5D7I9      388 -------PLIGWPMAGEQFY----------NTIMLGDEKVGVC--------------V--
W1PFP0      388 -------PLIGWPLAGEQFY----------NTIMLGDEKVGVC--------------V--
OsUGT99A1   384 -------PVVAWPHFTDQFL----------NAKMA-VEVLGIG--------------V--
AtUGT73B1   388 -------PMVTWPVGAEQFY----------NEKLV-TQVLKTG--------------V--
OsUGT98B1   392 -------PMITWPHCSEQFV----------NEKLV-VDHLKIG--------------V--
W1PF90      395 -------AMVTWPLSAEQFI----------NAKMV-SEVMGIG--------------FGV
W1NIK4      387 -------PLAAWPMFAEQPL----------NARLV-VDVLKTG--------------V--
W1PQX3      391 -------PMVSWPLYAEQPL----------NARLV-VDVLKVA--------------V--
W1PPW8      390 -------PMVAWPLNAEQPM----------NAKLV-VDVLKVA--------------V--
W1PS80      390 -------PMVAWPLNAEQPM----------NAKLV-VDVLKVA--------------V--
AtUGT90A1   375 -------PLLAWPMMAEQPL----------NAKMV-VEEIKVG--------------V--
OsUGT97A1   402 -------PMLAWPVMAEQAA----------NAKLV-ADIIGAG--------------V--
W1PH20      368 -------PILGWGMIAEQQL----------NAKVV-EEVLGVG--------------I--
W1PBB6      389 -------PILGWPQMAEQSL----------NAKMV-EEELGVG--------------S--
W1PHN8      389 -------PILGWPMMAEQSL----------NGAWA-GPRLVFA--------------F--
W1PRF4      348 -------PILAWPQFGDQMI----------NAGVV--EKSGLG-----------------
AtUGT71B1   374 -------PMAAWPIYAEQQF----------NAFHM-VDELGLA--------------A--
W1P1M0      366 -------PMIAWPLYAEQRM----------NALML-ANEIKVA--------------L--
AtUGT72B1   379 -------PLIAWPLYAEQKM----------NAVLL-SEDIRAA--------------L--
W1PMA9      379 -------PLIAWPLYAEQKM----------NAILL-VDEIKVA--------------I--
W1PP20      377 -------PLIAWPLYAEQKM----------NAILL-VDEIKVA--------------I--
AtUGT88A1   374 -------PMVAWPLYAEQRF----------NRVMI-VDEIKIA--------------I--
U5DB27      321 -------PILAFPLYAEQRL----------NAWEL-VKDMGVGVGVGVGVGLGVGVDV--
U5DBY1      377 -------PIVAFPLYAEQRL----------NAWEL-VKDVGVGVGVGVGVGVGVGVDV--
U5DBY4      387 -------PMVAWPLYAEQRL----------NALEL-VREVGVA--------------L--
U5D276      374 -------PMVAWPLYAEQRL----------NALVL-VRELGVA--------------L--
U5DB32      374 -------PMVAWPLYAEQRL----------NALVL-VRELGVA--------------L--
W1NHX5      372 -------PMLAWPLYAEQHL----------NALFM-VEEMKIA--------------M--
W1NVU7      332 ----------MAPLCRATFE----------RAVF--GGRDGTG-----------------
W1PAP0      376 -------PMAAWPLYAEQHV----------NAWLM-VGEREMG--------------VAV
W1PD80      377 -------PMAAWPIYAEQAM----------NAFLM-VGEMGLA--------------V--
W1PZW0      376 -------PMAAWPLYAEQES----------NAFMM-VGALQLS--------------V--
AtUGT82A1   381 -------RLLCYPVAGDQFV----------NCKYI-VDVWKIG--------------V--
W1NUV1      504 -------PMLGLPLLTDQFT----------NRKLI-VDDWGVG--------------L--
AtUGT86A1   384 -------PLLCYPLLTDQFT----------NRKLV-VDDWCIG--------------I--
AtUGT87A1   360 -------PLLTFPVFWDQFL----------NAKMI-VEEWRVG--------------M--
W1P188      369 -------PMLTFPIYFDQQT----------NSKLI-VDDWKVG--------------L--
W1P188        1 ------------------------------------------------------------
```

```
W1P302      372  -------PMLTFPINFDQPM----------NSKLV-TDDWKVG--------------L--
AtUGT78D1   365  -------PMIGRPILADNRL----------NGRAV-EVVWKVG--------------V--
W1PTN8      363  -------PMICHPFFGDQRI----------NARTV-TDLWRIG--------------V--
ZmUGT77A1   384  -------PMACRPFFGDQRM----------NARSV-AHVWGFG--------------A--
AtUGT83A1   373  -------PFLCIPYFADQFI----------NKAYI-CDVWKIG--------------L--
W1NWE7      254  -------PLICWPKFVDQFL----------NKSYI-VDIWKVG--------------L--
W1NXD5      374  -------PFICWPYFADQFL----------NQTYI-VDIWKVG--------------V--
AtUGT75B1   364  -------PVVAFPMWSDQPT----------NAKLL-EESWKTG--------------V--
AtUGT84A1   382  -------PVVCCPQWGDQVT----------DAVYL-IDVFKTG--------------V--
W1PLB6      373  -------PVIALPHWGDQVT----------NAKFL-VDIYGVG--------------V--
U5CXN6      374  -------PVLALAHWGDQVT----------NEKFL-VDVYSVG--------------I--
U5D2S8      364  -------PILAFAHWGDQVT----------NAKFL-VDVFGVG--------------V--
U5D817      371  -------PILAFSHWGDQVT----------NAKFL-VDVYSVG--------------V--
W1NFL7      371  -------PILAFAHWGDQVT----------NAKFL-VDVYSVG--------------V--
W1NFN9      371  -------PVLAFAHWGDQVT----------NAKFL-VDVYSVG--------------V--
U5DBW3      368  -------PVIAFPQWTDQPT----------NAMFL-VDVLRVG--------------V--
W1PE57      371  -------PVIAIPEWTDQPT----------NAMLL-VEVLGVG--------------L--
U5D3G1      359  -------PMVVLPEWSDQTT----------NAWLV-ERVWKCG--------------V--
AtUGT74B1   369  -------PMVGVPQWSDQMN----------DAKFV-EEVWKVG--------------Y--
U5DFQ6      376  -------PLVAIPQWTDQLT----------NAKYV-EDVWRVG--------------L--
U5DI88      403  -------PMVAVPQWTDQPT----------DAKFV-SDVWQVG--------------I--
U5DCN3      372  -------PMVAMPQWTDQLT----------NAKFV-SDVWRVG--------------M--
U5D531      311  -------PMVSVPQWGDQQT----------NSKFV-SDVWKTR--------------T--
U5CUG8      350  -------PMVTVPQRRYHPT----------NAKFV-SDVWITG--------------A--
W1PLP3      297  -------PMVTVPQRSYQPT----------NAKFV-SDVWITG--------------A--
U5DI92      371  -------PMIAIPQWSDQPT----------NSKLV-SDVWKLG--------------V--
U5D3V6      369  -------PMVAIPLRSDQPT----------NSKFI-SDVWKMG--------------V--
U5DDJ3      371  -------PMVAIPQWSDQPT----------NSKLV-SDVWKMG--------------V--
U5DFQ3      371  -------PMVAIPQWSDQPT----------NSKLV-SDVWKMG--------------V--
U5DI97      371  -------PMVAAPLRSDQPT----------NSKFI-FDVWKVG--------------V--
U5DCP2      372  -------PMVAVPQWSDQPT----------NAKFI-SDVWKMG--------------I--
U5DDK2      372  -------PMVAVPQWSDQPT----------NAKFI-SDVWKMG--------------I--
OsUGT709A3  393  -------PTVCWPFFTDQQI----------NSRFV-GGVWRTG--------------L--
SgUGT720A1  381  -------PMISCPKIGDQSS----------NCTWI-SKVWKIG--------------L--
U5CX27      274  -------PMLCWPFFADHPT----------ICRYV-CKEWGIG--------------M--
U5D008      285  -------PMICWPFFADPQT----------NCWYV-CKELGIG--------------M--
W1PMY7      299  -------PVLCWPFFAEQQT----------NCRFA-CVEWGIG--------------M--
AtUGT85A1   396  -------PMVCWPFFADQQM----------NCKFC-CDEWDVG--------------I--
U5CVP6      390  -------PVICWPFFAEQQT----------NCWFA-CNEWGMG--------------M--
U5CNJ5      389  -------PMICWPFFADQQT----------NCYLA-CAVWGIG--------------I--
U5CNG3      391  -------PMICWPFFAEQQT----------NCFFA-CTEWEVG--------------M--
U5CXJ9      391  -------PMICWPFFAEQQT----------NCFFA-CTEWGVG--------------M--
AtUGT76B1   360  -------PMICRPSFGDQRV----------NARYI-NDVWKIG--------------L--
W1PV81      362  -------PMACWARFGDQKV-------------------------------------L--
W1PVY0      360  -------PVACWPQFSDQRV----------NARLI-CHVWRVG--------------A--
W1PX56      393  -------PMACWPQFSDQRV----------NSRLI-CHVWGVG--------------A--
AtUGT90A1   375  -------PLLAWPMMAEQPL----------NAKMV-VEEIKVG--------------V--
OsUGT97A1   402  -------PMLAWPVMAEQAA----------NAKLV-ADIIGAG--------------V--
W1PH20      368  -------PILGWGMIAEQQL----------NAKVV-EEVLGVG--------------I--
W1PBB6      389  -------PILGWPQMAEQSL----------NAKMV-EEELGVG--------------S--
W1PHN8      389  -------PILGWPMMAEQSL----------NGAWA-GPRLVFA--------------F--
W1PRF4      348  -------PILAWPQFGDQMI----------NAGVV--EKSGLG-----------------
AtUGT71B1   374  -------PMAAWPIYAEQQF----------NAFHM-VDELGLA--------------A--
W1P1M0      366  -------PMIAWPLYAEQRM----------NALML-ANEIKVA--------------L--
AtUGT72B1   379  -------PLIAWPLYAEQKM----------NAVLL-SEDIRAA--------------L--
W1PMA9      379  -------PLIAWPLYAEQKM----------NAILL-VDEIKVA--------------I--
W1PP20      377  -------PLIAWPLYAEQKM----------NAILL-VDEIKVA--------------I--
AtUGT88A1   374  -------PMVAWPLYAEQRF----------NRVMI-VDEIKIA--------------I--
U5DB27      321  -------PILAFPLYAEQRL----------NAWEL-VKDMGVGVGVGVGVGLGVGVDV--
U5DBY1      377  -------PIVAFPLYAEQRL----------NAWEL-VKDVGVGVGVGVGVGVGVGVDV--
U5DBY4      387  -------PMVAWPLYAEQRL----------NALEL-VREVGVA--------------L--
U5D276      374  -------PMVAWPLYAEQRL----------NALVL-VRELGVA--------------L--
U5DB32      374  -------PMVAWPLYAEQRL----------NALVL-VRELGVA--------------L--
W1NHX5      372  -------PMLAWPLYAEQHL----------NALFM-VEEMKIA--------------M--
W1NVU7      332  ----------MAPLCRATFE----------RAVF--GGRDGTG-----------------
W1PAP0      376  -------PMAAWPLYAEQHV----------NAWLM-VGEREMG--------------VAV
W1PD80      377  -------PMAAWPIYAEQAM----------NAFLM-VGEMGLA--------------V--
W1PZW0      376  -------PMAAWPLYAEQES----------NAFMM-VGALQLS--------------V--
AtUGT82A1   381  -------RLLCYPVAGDQFV----------NCKYI-VDVWKIG--------------V--
W1NUV1      504  -------PMLGLPLLTDQFT----------NRKLI-VDDWGVG--------------L--
AtUGT86A1   384  -------PLLCYPLLTDQFT----------NRKLV-VDDWCIG--------------I--
AtUGT87A1   360  -------PLLTFPVFWDQFL----------NAKMI-VEEWRVG--------------M--
W1P188      369  -------PMLTFPIYFDQQT----------NSKLI-VDDWKVG--------------L--
W1P188        1  ------------------------------------------------------------
```

```
U5CYW0       380 -VFS---------KSPKDTANTVG-EWFGPKVE---------------------------
W1PAZ2       455 -KFS---------KSP---------KEIANIVA---------------------------
AtUGT81A1    470 -KFS---------KSP---------KEISKIVA---------------------------
W1PTT2       452 -PYP---------EGPSLQSLLAQNQEIIDCCTSNNFDFK--------------------
U5D3V5           ------------------------------------------------------------
W1NUG7       454 GEET----QSLRYLQDQNERILRFCEEANIEMK---------------------------
W1PC48       438 -TVD-----SLEHSLSQNRRILEFCEKQGIGMK---------------------------
W1PP63       421 VGMLRSASPQCVSESPCLQSYLQQNNKITEGATSISLHSNSKIQIADPNGQDHELETISG
OsUGT701A2   433 -MYI--------------FGVLRS-TDPARCGR---------------------------
W1PA48       473 -RDQ---------LLPNNEGEITI-LQASKLVL---------------------------
U5D0T8       436 RGVG---------PPPIPVDQFSC-PKLVDAIR---------------------------
AtUGT80A2    553 RGVG---------PSPIPVDEFSL-HKLEDAIN---------------------------
W1PJ19       375 -EVR---------RTNLEDGSVSA-ENVCEAIR---------------------------
W1NU70       430 -EIE---------GRSLEDGNFTS-ELINRAIV---------------------------
W1NV64       415 -EIE---------RANLEDGSFTA-DSVCRAVQ---------------------------
W1PKS4       412 -EIE---------RANLEDGSFTA-ESVCRAVQ---------------------------
AtUGT91A1    406 -YMI---------PRDETEGFFTK-ESVANSLR---------------------------
U5CMK2       403 -EIE---------RNK--DGSFTK-EAVAKGLR---------------------------
U5CWM7       368 -EVE----------RNENDGSFTK-ISIAKALR---------------------------
U5D1X9       406 -EVE----------RNEDDGSFTK-VSIAKALR---------------------------
W1PT00       383 -EVE---------RES--DGGFKG-EKVAKALK---------------------------
OsUGT94A1    401 -RVR---------QER--FGEFEA-EEVARAVR---------------------------
AtUGT79B1    402 -EVE---------REK--KGWFSR-QSLENAVK---------------------------
W1PQK6       407 -EVE----------RRAEDGFFRR-EDVCKAVR---------------------------
U5D602       387 -AVR---------ELNEGDGFVIA-EAVERAVR---------------------------
U5D5X9       362 -GVK---------EWHEREDIVRA-ERVEKAVR---------------------------
U5D5Z2       421 -AVG---------EWHERDDIVRA-ESVERAVR---------------------------
U5D5X3       319 -ALK---------EWHERDDIVRT-ERVERAVR---------------------------
ZmUGT93B1    401 -LVR---------PWEKHAEIVPA-QAIQKVIE---------------------------
PoUGT95A1    480 SRGA---------NGEVGPGKFTK-DDITSGIE---------------------------
U5D0B7       496 -SII---------MDTEMDGEISK-DVILAGIE---------------------------
AtUGT89A1    396 -SVC---------EGG---KTVPDPYEMGRIIA---------------------------
OsUGT96A1    391 -RVA---------EGA--AAVPDA-ATLARALA---------------------------
AtUGT92A1    416 -EVA---------RGK--RCEIKC-DDIVSKIK---------------------------
U5CRD3       356 -ELG---------RGQ--EEVVGW-EKVERAVR---------------------------
W1P7W1       418 -ELT---------RGQ--EAGVGR-EVVARVVG---------------------------
U5D7I9       415 -EVG---------RGK--EVGVDR-EKVARVVA---------------------------
W1PFP0       415 -EVG---------RGK--EVGVDR-EKVARVVA---------------------------
OsUGT99A1    410 -GVGVEEPLVYQRVRK--EIVVGR-GTVEAAVR---------------------------
AtUGT73B1    414 -SVG------VKKMMQVVGDFISR-EKVEGAVR---------------------------
OsUGT98B1    418 -EVGVKGVTQWGTEQK--EVKVTR-TAVETAVS---------------------------
W1PF90       423 ERVG--------------KEVVRR-EKVKEAVE---------------------------
W1NIK4       413 -EIS----------KDDPNGLVRK-EEIHRALC---------------------------
W1PQX3       417 -AIV----------DKETQGVVPK-EAVERAVS---------------------------
W1PPW8       416 -PIV---------FDT--QGVVPK-EAVEQAVS---------------------------
W1PS80       416 -PIV---------FDT--QGVVPK-EAVEQAVS---------------------------
AtUGT90A1    401 -RVE--------TEDGSVKGFVTR-EELSGKIK---------------------------
OsUGT97A1    428 -RAV---------RGG--GVVVGR-AEVAGKVG---------------------------
W1PH20       394 -RVL--------EEGEGEGGNLVEGEVISGKVA---------------------------
W1PBB6       415 -RVV------WGVGEK--GEIVER-EVVAREVV---------------------------
W1PHN8       415 -PVA---------PFALSSALCPLPYPVPFALS---------------------------
W1PRF4       372 -VWP---------QGWCGERVVNR-EDVCKQIK---------------------------
AtUGT71B1    400 -EVKKEYRRDFLVEEP---EIVTA-DEIERGIK---------------------------
W1P1M0       392 -RPK---------MED--DGIVYK-HEIERAVR---------------------------
AtUGT72B1    405 -RPR---------AGD--DGLVRR-EEVARVVK---------------------------
W1PMA9       405 -RPK---------MQE--DGIIKV-EEIDRVVR---------------------------
W1PP20       403 -RPK---------IQE--DGIIKE-EEIERVVR---------------------------
AtUGT88A1    400 -SMN---------ESE--TGFVSS-TEVEKRVQ---------------------------
U5DB27       361 -EVD---------SGS--CGLVKA-EKLEKAVR---------------------------
U5DBY1       417 -GVG---------VGS---GLVKA-EELEKAVQ---------------------------
U5DBY4       413 -ELK---------MEE--DGWVKA-EELERVAR---------------------------
U5D276       400 -EME---------MAE--DGVVRA-EALGSRVR---------------------------
U5DB32       400 -EVE---------MGE--DGVVRA-EELGSRVR---------------------------
W1NHX5       398 -AME---------IGD--SGFVDG-GAVARCVK---------------------------
W1NVU7       353 ------------------NGDGGRGRWLCRCRC---------------------------
W1PAP0       404 GAVE---------RGE--DGVVGA-EAVARLVR---------------------------
W1PD80       403 -AVE---------RGD--DGFVKA-EEVARCVR---------------------------
W1PZW0       402 -RLE---------RDD--NGFVKA-EALARCLR---------------------------
AtUGT82A1    407 -RLS----------------GFGE-KEVEDGLR---------------------------
W1NUV1       530 -DLM---------------RKLRR-EEVKENME---------------------------
AtUGT86A1    410 -NLC-------------EKKTITR-DQVSANVK---------------------------
AtUGT87A1    386 -GIE---------RKKQMELLIVS-DEIKELVK---------------------------
W1P188       395 -RVR---------SGPMDEMEVVDRREIANVVR---------------------------
W1P188         1 ------------------------------------------------------------
```

```
W1P302       398 -RVR---------SGPMGENELVGRGEIASLVR---------------------------
AtUGT78D1    391 -MMD--------------NGVFTK-EGFEKCLN---------------------------
W1PTN8       389 -TIE--------------GGVLTR-EGVEGAVH---------------------------
ZmUGT77A1    410 -AFE---------------GAMTS-AGVATAVE---------------------------
AtUGT83A1    399 -GLE---------RDA--RGVVPR-LEVKKKID---------------------------
W1NWE7       280 -ELE---------GNK--DGFFSK-EEIRGKVE---------------------------
W1NXD5       400 -GLK---------ANE--DGVFSK-EEIKSKVE---------------------------
AtUGT75B1    390 -RVR---------ENK--DGLVER-GEIRRCLE---------------------------
AtUGT84A1    408 -RLG---------RGATEERVVPR-EEVAEKLL---------------------------
W1PLB6       399 -RLK---------RGS--EGFVRR-EEVCRCMD---------------------------
U5CXN6       400 -RLE---------RYS--EGVVRR-EEVSRCIE---------------------------
U5D2S8       390 -RLE---------RDA--EGVVRR-DEVCRCIE---------------------------
U5D817       397 -RLE---------RDA--EGVVRR-EEVCRCIK---------------------------
W1NFL7       397 -RLE---------RDA--EGVVRR-EEVCRCIE---------------------------
W1NFN9       397 -RLE---------RDA--EGVVRR-EEVCRCIE---------------------------
U5DBW3       394 -RLK--------------VDLVTR-DEVERCIA---------------------------
W1PE57       397 -RLR---------AGP--DGVVSR-EEVAKCIT---------------------------
U5D3G1       385 -RAK---------VRE--DGVLEE-AELKRCIE---------------------------
AtUGT74B1    395 -RAK----------EEAGEVIVKS-EELVRCLK---------------------------
U5DFQ6       402 -RAR---------VND--MGFVSR-GELERCIR---------------------------
U5DI88       429 -RAK---------ADD--RGIVCR-DEVAKCLR---------------------------
U5DCN3       398 -RPK---------ADN--RGIVGR-EEVERCLR---------------------------
U5D531       337 -RVE---------IGD--KGFVLR-GELERCIR---------------------------
U5CUG8       376 -RLE---------VGN--KGFVLK-GELERCIR---------------------------
W1PLP3       323 -RLE---------VGN--KGFVLK-RELERCLR---------------------------
U5DI92       397 -RAE---------ADE--TEVVRR-GELERCIR---------------------------
U5D3V6       395 -RAE---------ADE--QEVVRR-GELKRCIR---------------------------
U5DDJ3       397 -RVE---------VDE--KEVVRS-GEVERCIR---------------------------
U5DFQ3       397 -RVE---------VDE--KEVVRR-GEVERCIR---------------------------
U5DI97       397 -CAE---------VDG--NEVIRK-GELERCIR---------------------------
U5DCP2       398 -RVE---------VDE--KEVVRS-GELERCIR---------------------------
U5DDK2       398 -RVE---------VDD--KKMVQR-GELERCIR---------------------------
OsUGT709A3   419 -DMK---------------DVCDA-AVVARMVR---------------------------
SgUGT720A1   407 -EME---------------DRYDR-VSVETMVR---------------------------
U5CX27       300 -EIN---------------NDVKR-EEVEALVR---------------------------
U5D008       311 -EIN---------------NDVKR-EDVEELVR---------------------------
W1PMY7       325 -EIG---------------DDVKR-QKVETQVK---------------------------
AtUGT85A1    422 -EIG---------------GDVKR-EEVEAVVR---------------------------
U5CVP6       416 -EIE---------------NDVKR-EEVEALVR---------------------------
U5CNJ5       415 -EID---------------NNVKR-EEVEAHVK---------------------------
U5CNG3       417 -EID---------------NNVKR-EEVEAQVR---------------------------
U5CXJ9       417 -EID---------------NNVKR-EEVEAQVR---------------------------
AtUGT76B1    386 -HLE---------------NKVER-LVIENAVR---------------------------
W1PV81       376 ------------------ESSLEK------------------------------------
W1PVY0       386 -EMG---------------REVER-GEVEMVVR---------------------------
W1PX56       419 -EMR---------------REVER-GEVEMVVR---------------------------
AtUGT90A1    401 -RVE--------TEDGSVKGFVTR-EELSGKIK---------------------------
OsUGT97A1    428 -RAV---------RGG--GVVVGR-AEVAGKVG---------------------------
W1PH20       394 -RVL--------EEGEGEGGNLVEGEVISGKVA---------------------------
W1PBB6       415 -RVV------WGVGEK--GEIVER-EVVAREVV---------------------------
W1PHN8       415 -PVA---------PFALSSALCPLPYPVPFALS---------------------------
W1PRF4       372 -VWP---------QGWCGERVVNR-EDVCKQIK---------------------------
AtUGT71B1    400 -EVKKEYRRDFLVEEP---EIVTA-DEIERGIK---------------------------
W1P1M0       392 -RPK---------MED--DGIVYK-HEIERAVR---------------------------
AtUGT72B1    405 -RPR---------AGD--DGLVRR-EEVARVVK---------------------------
W1PMA9       405 -RPK---------MQE--DGIIKV-EEIDRVVR---------------------------
W1PP20       403 -RPK---------IQE--DGIIKE-EEIERVVR---------------------------
AtUGT88A1    400 -SMN---------ESE--TGFVSS-TEVEKRVQ---------------------------
U5DB27       361 -EVD---------SGS--CGLVKA-EKLEKAVR---------------------------
U5DBY1       417 -GVG---------VGS---GLVKA-EELEKAVQ---------------------------
U5DBY4       413 -ELK---------MEE--DGWVKA-EELERVAR---------------------------
U5D276       400 -EME---------MAE--DGVVRA-EALGSRVR---------------------------
U5DB32       400 -EVE---------MGE--DGVVRA-EELGSRVR---------------------------
W1NHX5       398 -AME---------IGD--SGFVDG-GAVARCVK---------------------------
W1NVU7       353 ------------------NGDGGRGRWLCRCRC---------------------------
W1PAP0       404 GAVE---------RGE--DGVVGA-EAVARLVR---------------------------
W1PD80       403 -AVE---------RGD--DGFVKA-EEVARCVR---------------------------
W1PZW0       402 -RLE---------RDD--NGFVKA-EALARCLR---------------------------
AtUGT82A1    407 -RLS----------------GFGE-KEVEDGLR---------------------------
W1NUV1       530 -DLM---------------RKLRR-EEVKENME---------------------------
AtUGT86A1    410 -NLC-------------EKKTITR-DQVSANVK---------------------------
AtUGT87A1    386 -GIE---------RKKQMELLIVS-DEIKELVK---------------------------
W1P188       395 -RVR---------SGPMDEMEVVDRREIANVVR---------------------------
W1P188         1 ------------------------------------------------------------
```

```
U5CYW0       402 -----------ELM--------------QMSECARRLAQPDAVFNIVRDIDDLARKRGPM
W1PAZ2       469 -----------QWF----------GPKADELKSMSQNALKLARPDAVFKIVH--------
AtUGT81A1    484 -----------DWF----------GPASKELEIMSQNALRLAKPEAVFKIVH--------
W1PTT2       482 -----------VYI----------PHCNSQDQWKAHFGNQWSRFVERKAAFDPMAVL-AP
U5D3V5           ------------------------------------------------------------
W1NUG7       483 -----------QYL----------PHHTKRAQWVQHYGPKWAEFEERKATFDPKNIL-AP
W1PC48       465 -----------QYL----------PHYTTKSEWAAHFSHRWQE-FVRR----------KA
W1PP63       481 SDSLKRSDLGGKLY---------LSYYSEEEHWKKHFGGKWERFLARKKMFDPLQIL-AP
OsUGT701A2   450 -----------ACVDSIMARHRRVADEACRDGGGGGRGIGAKQYLARQPSPARWRDHFGA
W1PA48       495 -----------NAI--------------EVALSTKVKTCASQI-ARRISFED----GIGE
U5D0T8       459 -----------FML----------------DPKVKERAEELARVMASED-------GVAG
AtUGT80A2    576 ------------FM---------LDDKVKSSAETLAKAMKDED-GVAGAVKAFFKHL-PS
W1PJ19       397 -----------RAV----------SE--GDDTETWRNAKDLQERVVHKLELE--------
W1NU70       452 -----------RVV----------ME--AEDTETWINAKDLQARVFGNMELQ--------
W1NV64       437 -----------RAV----------SE--EDDSETWRNAKDLQARIFCDTELE--------
W1PKS4       434 -----------RAV----------SE--EDDAKTWSNAKDLQARVFGNTELE--------
AtUGT91A1    428 -----------LVM----------VE--EEGKVYRENVKEMKGVFGDMDRQD--------
U5CMK2       423 -----------QAM----------VE--EEGEPLRVKAREAEAIFADR----------GL
U5CWM7       389 -----------LAM----------VE--EEGEPLRIKAREMNPIFGNRELSN--------
U5D1X9       427 -----------FVM---------VE---EEGEPLRVKAREMKPIFGNKELSD--------
W1PT00       403 -----------MVV----------LE--REGELVRERARRLAE-KIK-----------ED
OsUGT94A1    421 -----------AVM--------------RGGEALRRRATELREVVARRDAEC--DEQIGA
AtUGT79B1    422 -----------SVM----------EEGSEIGEKVRKNHDKWRCVLTDS----------GF
W1PQK6       428 -----------CVM---------VEADKEPGKRVRENHERLKQFLLDDKVQC--------
U5D602       409 -----------SLM----------AS--EEGSEVREKALRLRD-SARQAE------G-GT
U5D5X9       384 -----------KLM----------GS--GEGAEMKRKAAELRD-AARRAVGK----G-GT
U5D5Z2       443 -----------KLM----------AS--EEGAEMGRKAAELRD-AAQQAVGE----G-GT
U5D5X3       341 -----------KLMVFEEGGGDEEEGGGAEGRRPKGGGRGLAPPLIPFSNSSLVLKTILA
ZmUGT93B1    423 -----------EAM----------LS--DSGMAVRQRAKELGE-AVRASVAD----G-GN
PoUGT95A1    503 -----------KLM---------------KDEKVHKQAKELSK-EFEGGFPV-------S
U5D0B7       518 -----------RVM---------------SAVEARRQVESIRK-IFSGGFPA----SSRS
AtUGT89A1    416 -----------DTM-----------G--ESGGEARARAKEM---------------G---
OsUGT96A1    411 -----------DAV----------DG--AKCGDVRAKAAALAA-AAAAAVEE----G-GS
AtUGT92A1    436 -----------LVM---------EET--EVGKEIRKKAREVKE-LVRRAMVD--GVK-GS
U5CRD3       376 -----------VVM--------------------------------------------GD
W1P7W1       438 -----------EVM----------GG--KKGEELRKRAATASE-ALRTAAED----G-GS
U5D7I9       435 -----------EVV----------KG--EKGAEMRRKAAEMRE-DLRKGMAEEESGG-GS
W1PFP0       435 -----------EVV----------KG--EKGAEMRRKAGEMRE-ALRKGMAEEESGG-GS
OsUGT99A1    439 -----------SAM---------DGG--EEGEARRWRARALAA-KARAAARE----G-GS
AtUGT73B1    439 -----------EVM---------------VGEERRKRAKELAE-MAKNAVKE----G-GS
OsUGT98B1    447 -----------MLM---------DEG--EVAQEIRMRAKDFGM-KARRALEE----G-GS
W1PF90       441 -----------EVM-----------G--EGGERKRMRAKELKE-MGRRAVEE----G-GS
W1NIK4       434 -----------EVM---------------SNSEMRDRAMKVGE-MAGKAVEE----G-GS
W1PQX3       438 -----------EMM---------------GNGEMRGRARELGE-MARRAVED----G-GS
W1PPW8       436 -----------EMM---------------GNGEMRGRARELGE-MARRAVEE----G-GS
W1PS80       436 -----------EMM---------GNG------EMRGRARELGE-MARRAVEE----G-GS
AtUGT90A1    424 -----------ELM----------EG--ETGKTARKNVKEYSK-MAKAALVE----GTGS
OsUGT97A1    448 -----------RLM---------DGG--EEGRAIRARAEEVRE-AARAAVGE----G-GA
W1PH20       418 -----------ELM----------RG--ERGRKARRRAAEVAE-AAREAVAP----G-GS
W1PBB6       438 -----------ELM----------SG--EKGRKARERAREIKE-KARKAVGE----G-GS
W1PHN8       438 ----------------------------------------------------------SA
W1PRF4       394 -----------GFM---------------GDVCAREGAARVRE-EARKAVAD----G-GD
AtUGT71B1    428 -----------CAM--------------EQDSKMRKRVMEMKD-KLHVALVD----G-GS
W1P1M0       412 -----------CLM----------EE--EEGKLLREKMGELKT-ATDSALAK----G-GT
AtUGT72B1    425 -----------GLM----------EG--EEGKGVRNKMKELKE-AACRVLKD----D-GT
W1PMA9       425 -----------GLM----------EG--EEGKALRAKMRDLRD-AASTAVAE----G-GS
W1PP20       423 -----------GLM----------EG--EEGKALRAKMRDLRDAAAATALAE----G-GS
AtUGT88A1    420 -----------EII---------------GECPVRERTMAMKN-AAELALTE----T-GS
U5DB27       381 -----------RLM----------EA--EEGQRVRRRGKELKE-AARKAVEE----G-GP
U5DBY1       436 -----------RLM----------EA--EEGQRLTRRVKELRE-VARKVVEE----G-GP
U5DBY4       433 -----------RLM----------EG--DEGKIVRKRVKELKE-ASTKVLEE----G-GS
U5D276       420 -----------ALM----------EE--EEGRRVRKRAKELKA-AAMKVVEE----G-GS
U5DB32       420 -----------ALM---------EEE--EEGRRVRKRAKELKE-AAMKALEE----G-GS
W1NHX5       418 -----------GLM----------ES--EKGNMLRDRSKALKE-KAATALSE----G-GT
W1NVU7       368 ------------------------SGSKREGGDGIRRGEDVK----------------GT
W1PAP0       425 -----------ELM----------DS--SRSMRVRERVAKLKE-RAVAALAE----G-GT
W1PD80       423 -----------ELM----------ES--EVGKRIRERMAAVKD-SAARALRV----G-GS
W1PZW0       422 -----------ELM----------ET--ESGKRLRERTTVLKD-GAAKALSE----G-GT
AtUGT82A1    422 -----------KVM---------------EDQDMGERLRKLRD-RAMGNEAR------LS
W1NUV1       546 -----------RVF---------------GEEGFRERVREVRGVLARAIGEE------GS
AtUGT86A1    428 -----------RLM----------NG--ETSSELRNNVEKVKR-HLKDAVTT----V-GS
AtUGT87A1    408 -----------RFM----------DGESEEGKEMRRRTCDLSE-ICRGAVAK----G-GS
W1P188       418 -----------RLM----------EG--EESKEMRRRIEELRE-AGEKALEK----G-GS
W1P188         1 ------------------------------------------------------------
```

```
W1P302       421 -----------RLF----------DG--EESKEMRRRIEKLKE-AGLKTFER----G-GS
AtUGT78D1    408 -----------DVF----------VH--DDGKTMKANAKKLKE-KLQEDFSM----K-GS
W1PTN8       406 -----------RLM----------AI--EEGQVLRGNVRTLMD-LATQATSN----E-GS
ZmUGT77A1    426 -----------ELL----------RG--EEGARMRARAKELQA-LVAEAFGP----G-GE
AtUGT83A1    419 -----------EIM--------------RDGGEYEERAMKVKE-IVMKSVAK----D-GI
W1NWE7       300 -----------ELL---------------GDQAIRERALKLKE-QAIYSVTE------GT
W1NXD5       420 -----------DLM---------------GDEGMRERVLKLKE-EAISSVTE------GS
AtUGT75B1    410 -----------AVM-------------EEKSVELRENAKKWKR-LAMEAGRE----G-GS
AtUGT84A1    430 -----------EAT----------VG--EKAEELRKNALKWKA-EAEAAVAP----G-GS
W1PLB6       419 -----------EIM----------EG--PSALRIRDNAIKLKE-AARDAWET----G-GS
U5CXN6       420 -----------EIT----------KG--PRASTIREKAMKLKD-AAREALEV----E-GS
U5D2S8       410 -----------EIR----------EG--PRASKMRENAMKLKE-AAREALEE----G-GS
U5D817       417 -----------EIR----------EG--QRASKIRENAMKLKE-AAREALEE----G-GS
W1NFL7       417 -----------EIR----------EG--PRAAKIRENAMKLKE-AAREALEE----G-GS
W1NFN9       417 -----------EIR----------EG--SRAAKIRENAMKLKE-AAREALEE----G-GS
U5DBW3       411 -----------EVT----------DG--EKATEMKKAAARCKE-MARASVAK----C-GS
W1PE57       417 -----------ATT----------QG--AMAEEMKTKAWTRKE-AARAALAH----G-GS
U5D3G1       405 -----------LVM---------GGG--EEGAEMKKKARELSW-LAKDAWKE----G-GS
AtUGT74B1    416 -----------GVM----------EG--ESSVKIRESSKKWKD-LAVKAMSE----G-GS
U5DFQ6       422 -----------EIM----------DG--DRGVEIKKNAAKWKI-LAMMAREI----S-GT
U5DI88       449 -----------DVL----------DD--DRAAQIRRNAERWRE-LAKEALDE----G-GS
U5DCN3       418 -----------EAM----------EG--ETAAEMRRNAERWSE-LAKEALDE----G-GS
U5D531       357 -----------EVM----------EG--ETGAHIRENAQRWRD-LTREAVAE----G-GS
U5CUG8       396 -----------EVM----------EG--ERGAHIRANAQRWRD-QAREAVAE----G-GP
W1PLP3       343 -----------EVI----------EG--ERGAHIRATAQRWRS-------------G---
U5DI92       417 -----------EFR----------------------------------------------
U5D3V6       415 -----------EVM----------EG--ERGAELRRNAQRWRD-LALEAVAE----G-GS
U5DDJ3       417 -----------EVM----------EG--ERGAELRRNAQRWKD-LAWEAVAE----G-GS
U5DFQ3       417 -----------EVM----------EG--ERGAELRRNARRWRD-FAWEAVAE----G-GS
U5DI97       417 -----------EVM----------EG--ERGAELRRNAHRWRG-LTTEAVAE----G-GT
U5DCP2       418 -----------EVM----------DG--ETGAELRENGQRWKK-LARAAVAD----G-GS
U5DDK2       418 -----------EIM----------DG--EKGAEFRENAQRWKG-LARAAVAE----R-GT
OsUGT709A3   435 -----------EAM---------------ESGEIRASAQSVAR-QLRRDVAE----G-GS
SgUGT720A1   423 -----------SIM-----------E--QEGEKMQKTIAELAK-QAKYKVSK----D-GT
U5CX27       316 -----------ELM----------EG--EKGKEMQLKAKKWKE-ISKTVVQL----G-GS
U5D008       327 -----------KLM----------GG--EKGKELQLKAKKSKV-SSKIVVQF----G-GS
W1PMY7       341 -----------EMI----------EG--LKGMKMRANAMKWKE-SSERAIGS----G-GS
AtUGT85A1    438 -----------ELM----------DG--EKGKKMREKAVEWQR-LAEKATEH----KLGS
U5CVP6       432 -----------EMM----------HG--EKGKAMRAKVRRWRE-LSEKSVEA----G-GY
U5CNJ5       431 -----------ELL----------QG--DKGKEMRKMAMKWKE-LAENAIE-----G-GS
U5CNG3       433 -----------ELM----------KG--EKGKEMREKAMKWKE-ESEKAIQC----G-GS
U5CXJ9       433 -----------ELM----------KG--EKGKEMREKAMKWKE-ESEKAIQC----G-GS
AtUGT76B1    402 -----------TLM----------TS--SEGEEIRKRIMPMKE-TVEQCLKL----G-GS
W1PV81       382 --------------------------------------------------------G-GS
W1PVY0       402 -----------RLL----------VE--REGEEMREKASELKK-VVQRSLGK----G-GS
W1PX56       435 -----------RLL-----------E--REGEEMRERASELKK-VVEKSLGK----G-GS
AtUGT90A1    424 -----------ELM----------EG--ETGKTARKNVKEYSK-MAKAALVE----GTGS
OsUGT97A1    448 -----------RLM---------DGG--EEGRAIRARAEEVRE-AARAAVGE----G-GA
W1PH20       418 -----------ELM----------RG--ERGRKARRRAAEVAE-AAREAVAP----G-GS
W1PBB6       438 -----------ELM----------SG--EKGRKARERAREIKE-KARKAVGE----G-GS
W1PHN8       438 ----------------------------------------------------------SA
W1PRF4       394 -----------GFM---------------GDVCAREGAARVRE-EARKAVAD----G-GD
AtUGT71B1    428 -----------CAM--------------EQDSKMRKRVMEMKD-KLHVALVD----G-GS
W1P1M0       412 -----------CLM----------EE--EEGKLLREKMGELKT-ATDSALAK----G-GT
AtUGT72B1    425 -----------GLM----------EG--EEGKGVRNKMKELKE-AACRVLKD----D-GT
W1PMA9       425 -----------GLM----------EG--EEGKALRAKMRDLRD-AASTAVAE----G-GS
W1PP20       423 -----------GLM----------EG--EEGKALRAKMRDLRDAAAATALAE----G-GS
AtUGT88A1    420 -----------EII---------------GECPVRERTMAMKN-AAELALTE----T-GS
U5DB27       381 -----------RLM----------EA--EEGQRVRRRGKELKE-AARKAVEE----G-GP
U5DBY1       436 -----------RLM----------EA--EEGQRLTRRVKELRE-VARKVVEE----G-GP
U5DBY4       433 -----------RLM----------EG--DEGKIVRKRVKELKE-ASTKVLEE----G-GS
U5D276       420 -----------ALM----------EE--EEGRRVRKRAKELKA-AAMKVVEE----G-GS
U5DB32       420 -----------ALM---------EEE--EEGRRVRKRAKELKE-AAMKALEE----G-GS
W1NHX5       418 -----------GLM----------ES--EKGNMLRDRSKALKE-KAATALSE----G-GT
W1NVU7       368 ------------------------SGSKREGGDGIRRGEDVK----------------GT
W1PAP0       425 -----------ELM----------DS--SRSMRVRERVAKLKE-RAVAALAE----G-GT
W1PD80       423 -----------ELM----------ES--EVGKRIRERMAAVKD-SAARALRV----G-GS
W1PZW0       422 -----------ELM----------ET--ESGKRLRERTTVLKD-GAAKALSE----G-GT
AtUGT82A1    422 -----------KVM---------------EDQDMGERLRKLRD-RAMGNEAR------LS
W1NUV1       546 -----------RVF---------------GEEGFRERVREVRGVLARAIGEE------GS
AtUGT86A1    428 -----------RLM----------NG--ETSSELRNNVEKVKR-HLKDAVTT----V-GS
AtUGT87A1    408 -----------RFM----------DGESEEGKEMRRRTCDLSE-ICRGAVAK----G-GS
W1P188       418 -----------RLM----------EG--EESKEMRRRIEELRE-AGEKALEK----G-GS
W1P188         1 ------------------------------------------------------------
```

```
U5CYW0       437 SSLTYSSMTSS---FASIPI----------------------------------------
W1PAZ2       500 ---DLHELVRQ---RSMLPQYACAS-----------------------------------
AtUGT81A1    515 ---DMHELVRK---KNSLPQLSCTA-----------------------------------
W1PTT2       520 GQRIFPRVQSSQ------------------------------------------------
U5D3V5           ------------------------------------------------------------
W1NUG7       521 GQRIFSAAQPI----ILPSASSS-------------------------------------
W1PC48       493 AYDPLAILAPG---QRIFPKANAFA-----------------------------------
W1PP63       531 GQRIFKR--------KYSA-HL--------------------------------------
OsUGT701A2   499 GWGRFAARKAR----FDPLHVLGPGQGIFPRTDSAGSM----------------------
W1PA48       525 ALRILKEEKIC---IPRPED----------------------------------------
U5D0T8       485 AVKAFHKHLPRNMPQPQPQPQPPTAPPGFFDRCLGLDFPLFWFLFPPSSTLPSRTYCEGM
AtUGT80A2    613 AKQNISDPIPEPSGFLSFRKCFGCS-----------------------------------
W1PJ19       426 -DQYVQEFIDR---VAF-------------------------------------------
W1NU70       481 -DKYIKLFVDH---VDTLVCCKKE------------------------------------
W1NV64       466 -MHYLSKFVNC---ATKLVCPKS-------------------------------------
W1PKS4       463 -KHYLSKFIDC---ATKLDYSKS-------------------------------------
AtUGT91A1    457 --RYVDSFLEY---LVTNR-----------------------------------------
U5CMK2       450 HDRYMNDFIKY---LQDHRRN---------------------------------------
U5CWM7       418 --MYLDGFANF---LKDNK-----------------------------------------
U5D1X9       456 --MYLDGFANF---LKNNK-----------------------------------------
W1PT00       428 GDKGVDELVKE---IRRLSLIKGDENLA--------------------------------
OsUGT94A1    454 LLHRMARLCGK---GTGRAAQLGH------------------------------------
AtUGT79B1    451 SDGYIDKFEQN---LIELVKS---------------------------------------
W1PQK6       460 -RTFEEDFVKK---LRCLLV----------------------------------------
U5D602       438 SHTALNSFLAL---ISRP------------------------------------------
U5D5X9       415 SRAALDSFLAH---ISRPWSILPDSCP---------------------------------
U5D5Z2       474 SGAALDTFLAL---ISRP------------------------------------------
U5D5X3       390 AIYYLMHLLDY---LTLIAFSRICL-----------------------------------
ZmUGT93B1    454 SRKDLDDFIGY---ITR-------------------------------------------
PoUGT95A1    529 SVKALGAFVEF---ISQKAT----------------------------------------
U5D0B7       547 ALDDFRQLVQQ---LNQQE-----------------------------------------
AtUGT89A1        ------------------------------------------------------------
OsUGT96A1    442 SRVAFESMAKE---LETACMSPSFG-----------------------------------
AtUGT92A1    470 SVIGLEEFLDQ-A-MVKKVEN---------------------------------------
U5CRD3       381 EE----------------------------------------------------------
W1P7W1       469 SLRAVEEFAKT---LVEGMAC---------------------------------------
U5D7I9       470 SLRALDEFVAS---IVSPHVHFVA------------------------------------
W1PFP0       470 SLRALDEFVAS---IVSPHV----------------------------------------
OsUGT99A1    471 SHANLLDLVER---FRPRHVAASEAANGTTAPPPPPRQ----------------------
AtUGT73B1    467 SDLEVDRLMEE---LTLVKLQKEKV-----------------------------------
OsUGT98B1    479 SYNNIKLLIQE---MGNKQNASG-------------------------------------
W1PF90       471 SHMSLGRLVED---LLNGLKQGA-------------------------------------
W1NIK4       462 SHLSLSLLIEE---LVSYNNRKQ-------------------------------------
W1PQX3       466 SHMNLTLFIQD---MITYKKPHAM------------------------------------
W1PPW8       464 SYMNLTLFIQD---MIAYKKARTMDTAPVCDSPPTCTSSK--------------------
W1PS80       464 SYMNLTLFIQD---MIAYKKARTMDTAPVCDSPPPCTSSE--------------------
AtUGT90A1    456 SWKNLDMILKE---LCKSRDSNGASE----------------------------------
OsUGT97A1    480 SREALRRLVDE---LRSSYVVAGDGTAAASSANGGSGKC---------------------
W1PH20       449 SYTALANLVED---LAGKRR----------------------------------------
W1PBB6       469 SHNNLSGFIDE---FCKRKP----------------------------------------
W1PHN8       440 TPYIYNRIV---------------------------------------------------
W1PRF4       422 SHLGLVRAMEK---WSHH------------------------------------------
AtUGT71B1    457 SNCALKKFVQD---VVDNVP----------------------------------------
W1P1M0       443 SYESVNDVAEE---WRFGSPIWKDMKKVSFVVGNR-------------------------
AtUGT72B1    456 STKALSLVALK-WKAHKKELEQNGNH----------------------------------
W1PMA9       456 SYHSVVNATQI---WRSTV-----------------------------------------
W1PP20       455 SYGSLVNATQK---WRSNV-----------------------------------------
AtUGT88A1    448 SHTALTTLLQS---WSPK------------------------------------------
U5DB27       412 SYAALATVAGK---WVQEK-----------------------------------------
U5DBY1       467 SYSALATVAGK-W-VQEK------------------------------------------
U5DBY4       464 SYASLASAIHR---ILKG------------------------------------------
U5D276       451 SQSSLKAAIHE---ILKAGVVSV-------------------------------------
U5DB32       452 SQSSLKAAINE---MLKGGEA---------------------------------------
W1NHX5       449 SRLSLDAAVEA---WLGAVQP---------------------------------------
W1NVU7       388 R-----------------------------------------------------------
W1PAP0       456 SVTSLAELVNA---WLEGPPPPPPPR----------------------------------
W1PD80       454 SLKNLEAMVNA---GKHLPSSLETEA----------------------------------
W1PZW0       453 SLKSLELLVKA---WDQAPN----------------------------------------
AtUGT82A1    449 SEMNFTFLKNE---LN--------------------------------------------
W1NUV1       574 SQANLDKFVVD---LKRKLGGPSKGEVISFDRDVYGE-----------------------
AtUGT86A1    459 SETNFNLFVSE---VRNRIETKLCNVNGLEISPSN-------------------------
AtUGT87A1    441 SDANIDAFIKD---ITKIV-----------------------------------------
W1P188       449 SDANMNDFLKS---LRS-------------------------------------------
W1P188         1 ------------------------------------------------------------
```

```
W1P302       452 SDISLNAFLES---LDC-------------------------------------------
AtUGT78D1    439 SLENFKILLDE---IVKV------------------------------------------
W1PTN8       437 STANLKALVEL---VVSL------------------------------------------
ZmUGT77A1    457 CRKNFDRFVEI---VCRA------------------------------------------
AtUGT83A1    448 SCENLNKFVNW---IKSQVN----------------------------------------
W1NWE7       327 SRKNLTSFIEA---MKMHV-----------------------------------------
W1NXD5       447 SMENLHGFMEA---MKRKS-----------------------------------------
AtUGT75B1    440 SDKNMEAFVED---ICGESLIQNLCEAEEVKVK---------------------------
AtUGT84A1    461 SDKNFREFVEKLGAGVTKTKDNGY------------------------------------
W1PLB6       450 SDKNLKAFVEE---VISLGRQEEVIEARLPSLNLKDHALTE-------------------
U5CXN6       451 SDKNLRAFAEE---VMAMAMARGEEAIRACSPTLNEFEGPIT------------------
U5D2S8       441 SYKNLRVFVEN---LMSLALEVQAIRVRLPEDQSPD------------------------
U5D817       448 SDKNLRVFVED---LISLAREEEAIRVRLPNDQSPDD-----------------------
W1NFL7       448 SDKNLMVFVED---LISLASEEEAIRLRLPKDQSPDD-----------------------
W1NFN9       448 SDKNLRVFVED---LISLASEEEAIRLLLPKDQSPDD-----------------------
U5DBW3       442 SERNMQAFVDD---IMDNGPIHEPKPGNGVLC----------------------------
W1PE57       448 SDRNIVAFVDD---VMKSGHSQGPG-----------------------------------
U5D3G1       437 SEINLDRLLEK---IVA-------------------------------------------
AtUGT74B1    447 SDRSINEFIES---LGK-------------------------------------------
U5DFQ6       453 SHRNIEDFVGE---LLHT------------------------------------------
U5DI88       480 SDRNIDEFVAA---VLENKDSGRDSLGVGGPNQA--------------------------
U5DCN3       449 SDRNINEFVAT---VCFRQVRHSCCIGH--------------------------------
U5D531       388 SDNNINEFITK---ISIHEGDGNI------------------------------------
U5CUG8       427 SDNNVKEFVTK---VPFLLLDREK------------------------------------
W1PLP3           ------------------------------------------------------------
U5DI92           ------------------------------------------------------------
U5D3V6       446 SDNNIKEFVAK---VVVFSLE---------------------------------------
U5DDJ3       448 SDNNIKKFVAN---MSSSHLNSM-------------------------------------
U5DFQ3       448 SDKNIKQFVAK---LSSSHLNSK-------------------------------------
U5DI97       448 SDNNIKELVAT---LSNFHLNNYHK-----------------------------------
U5DCP2       449 SDNNIKEFVAM---VSRC------------------------------------------
U5DDK2       449 SDNNIEEFVAM---VSRCRLKK--------------------------------------
OsUGT709A3   463 SAMELKRLVGFIGELATPIQHAKSESEV--------------------------------
SgUGT720A1   453 SYQNLECLIQD---IKKLNQIEGFINNPNFSDLLRV------------------------
U5CX27       347 SYNNLERLIKQ---VLPEKR----------------------------------------
U5D008       358 SHNNFERLIKE---VLLKKH----------------------------------------
W1PMY7       372 SSENLDRLVVE---VLLKKHGE--------------------------------------
AtUGT85A1    470 SVMNFETVVSK---FLLGQKSQD-------------------------------------
U5CVP6       463 SSLNLDRVIKE---VMLKPCKQLGDAC---------------------------------
U5CNJ5       461 SINNLDRLVTQ---GQLKLS----------------------------------------
U5CNG3       464 SLTNFDALG---------------------------------------------------
U5CXJ9       464 SLTNLDALVND---VLFKKSRA--------------------------------------
AtUGT76B1    433 SFRNLENLIAY---ILSF------------------------------------------
W1PV81       385 SYEAFEALVNK---LKS-------------------------------------------
W1PVY0       433 SYEALDSLILQ---LMSC------------------------------------------
W1PX56       465 SYDALESLILQ---LMSC------------------------------------------
AtUGT90A1    456 SWKNLDMILKE---LCKSRDSNGASE----------------------------------
OsUGT97A1    480 SREALRRLVDE---LRSSYVVAGDGTAAASSANGGSGKC---------------------
W1PH20       449 SYTALANLVED---LAGKRR----------------------------------------
W1PBB6       469 SHNNLSGFIDE---FCKRKP----------------------------------------
W1PHN8       440 TPYIYNRIV---------------------------------------------------
W1PRF4       422 SHLGLVRAMEK---WSHH------------------------------------------
AtUGT71B1    457 SNCALKKFVQD---VVDNVP----------------------------------------
W1P1M0       443 SYESVNDVAEE---WRFGSPIWKDMKKVSFVVGNR-------------------------
AtUGT72B1    456 STKALSLVALK-WKAHKKELEQNGNH----------------------------------
W1PMA9       456 SYHSVVNATQI---WRSTV-----------------------------------------
W1PP20       455 SYGSLVNATQK---WRSNV-----------------------------------------
AtUGT88A1    448 SHTALTTLLQS---WSPK------------------------------------------
U5DB27       412 SYAALATVAGK---WVQEK-----------------------------------------
U5DBY1       467 SYSALATVAGK-W-VQEK------------------------------------------
U5DBY4       464 SYASLASAIHR---ILKG------------------------------------------
U5D276       451 SQSSLKAAIHE---ILKAGVVSV-------------------------------------
U5DB32       452 SQSSLKAAINE---MLKGGEA---------------------------------------
W1NHX5       449 SRLSLDAAVEA---WLGAVQP---------------------------------------
W1NVU7       388 R-----------------------------------------------------------
W1PAP0       456 SVTSLAELVNA---WLEGPPPPPPPR----------------------------------
W1PD80       454 SLKNLEAMVNA---GKHLPSSLETEA----------------------------------
W1PZW0       453 SLKSLELLVKA---WDQAPN----------------------------------------
AtUGT82A1    449 SEMNFTFLKNE---LN--------------------------------------------
W1NUV1       574 SQANLDKFVVD---LKRKLGGPSKGEVISFDRDVYGE-----------------------
AtUGT86A1    459 SETNFNLFVSE---VRNRIETKLCNVNGLEISPSN-------------------------
AtUGT87A1    441 SDANIDAFIKD---ITKIV-----------------------------------------
W1P188       449 SDANMNDFLKS---LRS-------------------------------------------
W1P188         1 ------------------------------------------------------------
```

```
U5CYW0           -------FLES---LDC-------------------------------------------
W1PAZ2           -------LLDE---IVKV------------------------------------------
AtUGT81A1        -------LVEL---VVSL------------------------------------------
W1PTT2           -------FVEI---VCRA------------------------------------------
U5D3V5           -------FVNW---IKSQVN----------------------------------------
W1NUG7           -------FIEA---MKMHV-----------------------------------------
W1PC48           -------FMEA---MKRKS-----------------------------------------
W1PP63           -------FVED---ICGESLIQNLCEAEEVKVK---------------------------
OsUGT701A2       -------FVEKLGAGVTKTKDNGY------------------------------------
W1PA48           -------FVEE---VISLGRQEEVIEARLPSLNLKDHALTE-------------------
U5D0T8       545 KFMCGGEFAEE---VMAMAMARGEEAIRACSPTLNEFEGPIT------------------
AtUGT80A2        -------FVEN---LMSLALEVQAIRVRLPEDQSPD------------------------
W1PJ19           -------FVED---LISLAREEEAIRVRLPNDQSPDD-----------------------
W1NU70           -------FVED---LISLASEEEAIRLRLPKDQSPDD-----------------------
W1NV64           -------FVED---LISLASEEEAIRLLLPKDQSPDD-----------------------
W1PKS4           -------FVDD---IMDNGPIHEPKPGNGVLC----------------------------
AtUGT91A1        -------FVDD---VMKSGHSQGPG-----------------------------------
U5CMK2           -------LLEK---IVA-------------------------------------------
U5CWM7           -------FIES---LGK-------------------------------------------
U5D1X9           -------FVGE---LLHT------------------------------------------
W1PT00           -------FVAA---VLENKDSGRDSLGVGGPNQA--------------------------
OsUGT94A1        -------FVAT---VCFRQVRHSCCIGH--------------------------------
AtUGT79B1        -------FITK---ISIHEGDGNI------------------------------------
W1PQK6           -------FVTK---VPFLLLDREK------------------------------------
U5D602           ------------------------------------------------------------
U5D5X9           ------------------------------------------------------------
U5D5Z2           -------FVAK---VVVFSLE---------------------------------------
U5D5X3           -------FVAN---MSSSHLNSM-------------------------------------
ZmUGT93B1        -------FVAK---LSSSHLNSK-------------------------------------
PoUGT95A1        -------LVAT---LSNFHLNNYHK-----------------------------------
U5D0B7           -------FVAM---VSRC------------------------------------------
AtUGT89A1        -------FVAM---VSRCRLKK--------------------------------------
OsUGT96A1        -------LVGFIGELATPIQHAKSESEV--------------------------------
AtUGT92A1        -------LIQD---IKKLNQIEGFINNPNFSDLLRV------------------------
U5CRD3           -------LIKQ---VLPEKR----------------------------------------
W1P7W1           -------LIKE---VLLKKH----------------------------------------
U5D7I9           -------LVVE---VLLKKHGE--------------------------------------
W1PFP0           -------VVSK---FLLGQKSQD-------------------------------------
OsUGT99A1        -------VIKE---VMLKPCKQLGDAC---------------------------------
AtUGT73B1        -------LVTQ---GQLKLS----------------------------------------
OsUGT98B1        -------LG---------------------------------------------------
W1PF90           -------LVND---VLFKKSRA--------------------------------------
W1NIK4           -------LIAY---ILSF------------------------------------------
W1PQX3           -------LVNK---LKS-------------------------------------------
W1PPW8           -------LILQ---LMSC------------------------------------------
W1PS80           -------LILQ---LMSC------------------------------------------
AtUGT90A1        -------ILKE---LCKSRDSNGASE----------------------------------
OsUGT97A1        -------LVDE---LRSSYVVAGDGTAAASSANGGSGKC---------------------
W1PH20           -------LVED---LAGKRR----------------------------------------
W1PBB6           -------FIDE---FCKRKP----------------------------------------
W1PHN8           -------IV---------------------------------------------------
W1PRF4           -------AMEK---WSHH------------------------------------------
AtUGT71B1        -------FVQD---VVDNVP----------------------------------------
W1P1M0           -------VAEE---WRFGSPIWKDMKKVSFVVGNR-------------------------
AtUGT72B1        -------VALK-WKAHKKELEQNGNH----------------------------------
W1PMA9           -------ATQI---WRSTV-----------------------------------------
W1PP20           -------ATQK---WRSNV-----------------------------------------
AtUGT88A1        -------LLQS---WSPK------------------------------------------
U5DB27           -------VAGK---WVQEK-----------------------------------------
U5DBY1           -------VAGK-W-VQEK------------------------------------------
U5DBY4           -------AIHR---ILKG------------------------------------------
U5D276           -------AIHE---ILKAGVVSV-------------------------------------
U5DB32           -------AINE---MLKGGEA---------------------------------------
W1NHX5           -------AVEA---WLGAVQP---------------------------------------
W1NVU7           ------------------------------------------------------------
W1PAP0           -------LVNA---WLEGPPPPPPPR----------------------------------
W1PD80           -------MVNA---GKHLPSSLETEA----------------------------------
W1PZW0           -------LVKA---WDQAPN----------------------------------------
AtUGT82A1        -------LKNE---LN--------------------------------------------
W1NUV1           -------FVVD---LKRKLGGPSKGEVISFDRDVYGE-----------------------
AtUGT86A1        -------FVSE---VRNRIETKLCNVNGLEISPSN-------------------------
AtUGT87A1        -------FIKD---ITKIV-----------------------------------------
W1P188           -------FLKS---LRS-------------------------------------------
W1P188         1 ------------------------------------------------------------
```

```
W1P302          -------FLES---LDC-------------------------------------------
AtUGT78D1       -------LLDE---IVKV------------------------------------------
W1PTN8          -------LVEL---VVSL------------------------------------------
ZmUGT77A1       -------FVEI---VCRA------------------------------------------
AtUGT83A1       -------FVNW---IKSQVN----------------------------------------
W1NWE7          -------FIEA---MKMHV-----------------------------------------
W1NXD5          -------FMEA---MKRKS-----------------------------------------
AtUGT75B1       -------FVED---ICGESLIQNLCEAEEVKVK---------------------------
AtUGT84A1       -------FVEKLGAGVTKTKDNGY------------------------------------
W1PLB6          -------FVEE---VISLGRQEEVIEARLPSLNLKDHALTE-------------------
U5CXN6          -------FAEE---VMAMAMARGEEAIRACSPTLNEFEGPIT------------------
U5D2S8          -------FVEN---LMSLALEVQAIRVRLPEDQSPD------------------------
U5D817          -------FVED---LISLAREEEAIRVRLPNDQSPDD-----------------------
W1NFL7          -------FVED---LISLASEEEAIRLRLPKDQSPDD-----------------------
W1NFN9          -------FVED---LISLASEEEAIRLLLPKDQSPDD-----------------------
U5DBW3          -------FVDD---IMDNGPIHEPKPGNGVLC----------------------------
W1PE57          -------FVDD---VMKSGHSQGPG-----------------------------------
U5D3G1          -------LLEK---IVA-------------------------------------------
AtUGT74B1       -------FIES---LGK-------------------------------------------
U5DFQ6          -------FVGE---LLHT------------------------------------------
U5DI88          -------FVAA---VLENKDSGRDSLGVGGPNQA--------------------------
U5DCN3          -------FVAT---VCFRQVRHSCCIGH--------------------------------
U5D531          -------FITK---ISIHEGDGNI------------------------------------
U5CUG8          -------FVTK---VPFLLLDREK------------------------------------
W1PLP3          ------------------------------------------------------------
U5DI92          ------------------------------------------------------------
U5D3V6          -------FVAK---VVVFSLE---------------------------------------
U5DDJ3          -------FVAN---MSSSHLNSM-------------------------------------
U5DFQ3          -------FVAK---LSSSHLNSK-------------------------------------
U5DI97          -------LVAT---LSNFHLNNYHK-----------------------------------
U5DCP2          -------FVAM---VSRC------------------------------------------
U5DDK2          -------FVAM---VSRCRLKK--------------------------------------
OsUGT709A3      -------LVGFIGELATPIQHAKSESEV--------------------------------
SgUGT720A1      -------LIQD---IKKLNQIEGFINNPNFSDLLRV------------------------
U5CX27          -------LIKQ---VLPEKR----------------------------------------
U5D008          -------LIKE---VLLKKH----------------------------------------
W1PMY7          -------LVVE---VLLKKHGE--------------------------------------
AtUGT85A1       -------VVSK---FLLGQKSQD-------------------------------------
U5CVP6          -------VIKE---VMLKPCKQLGDAC---------------------------------
U5CNJ5          -------LVTQ---GQLKLS----------------------------------------
U5CNG3          -------LG---------------------------------------------------
U5CXJ9          -------LVND---VLFKKSRA--------------------------------------
AtUGT76B1       -------LIAY---ILSF------------------------------------------
W1PV81          -------LVNK---LKS-------------------------------------------
W1PVY0          -------LILQ---LMSC------------------------------------------
W1PX56          -------LILQ---LMSC------------------------------------------
AtUGT90A1       -------ILKE---LCKSRDSNGASE----------------------------------
OsUGT97A1       -------LVDE---LRSSYVVAGDGTAAASSANGGSGKC---------------------
W1PH20          -------LVED---LAGKRR----------------------------------------
W1PBB6          -------FIDE---FCKRKP----------------------------------------
W1PHN8          -------IV---------------------------------------------------
W1PRF4          -------AMEK---WSHH------------------------------------------
AtUGT71B1       -------FVQD---VVDNVP----------------------------------------
W1P1M0          -------VAEE---WRFGSPIWKDMKKVSFVVGNR-------------------------
AtUGT72B1       -------VALK-WKAHKKELEQNGNH----------------------------------
W1PMA9          -------ATQI---WRSTV-----------------------------------------
W1PP20          -------ATQK---WRSNV-----------------------------------------
AtUGT88A1       -------LLQS---WSPK------------------------------------------
U5DB27          -------VAGK---WVQEK-----------------------------------------
U5DBY1          -------VAGK-W-VQEK------------------------------------------
U5DBY4          -------AIHR---ILKG------------------------------------------
U5D276          -------AIHE---ILKAGVVSV-------------------------------------
U5DB32          -------AINE---MLKGGEA---------------------------------------
W1NHX5          -------AVEA---WLGAVQP---------------------------------------
W1NVU7          ------------------------------------------------------------
W1PAP0          -------LVNA---WLEGPPPPPPPPR---------------------------------
W1PD80          -------MVNA---GKHLPSSLETEA----------------------------------
W1PZW0          -------LVKA---WDQAPN----------------------------------------
AtUGT82A1       -------LKNE---LN--------------------------------------------
W1NUV1          -------FVVD---LKRKLGGPSKGEVISFDRDVYGE-----------------------
AtUGT86A1       -------FVSE---VRNRIETKLCNVNGLEISPSN-------------------------
AtUGT87A1       -------FIKD---ITKIV-----------------------------------------
W1P188          -------FLKS---LRS-------------------------------------------
W1P188        1 ------------------------------------------------------------
```

```
W1P302          -------FLES---LDC-------------------------------------------
AtUGT78D1       -------LLDE---IVKV------------------------------------------
W1PTN8          -------LVEL---VVSL------------------------------------------
ZmUGT77A1       -------FVEI---VCRA------------------------------------------
AtUGT83A1       -------FVNW---IKSQVN----------------------------------------
W1NWE7          -------FIEA---MKMHV-----------------------------------------
W1NXD5          -------FMEA---MKRKS-----------------------------------------
AtUGT75B1       -------FVED---ICGESLIQNLCEAEEVKVK---------------------------
AtUGT84A1       -------FVEKLGAGVTKTKDNGY------------------------------------
W1PLB6          -------FVEE---VISLGRQEEVIEARLPSLNLKDHALTE-------------------
U5CXN6          -------FAEE---VMAMAMARGEEAIRACSPTLNEFEGPIT------------------
U5D2S8          -------FVEN---LMSLALEVQAIRVRLPEDQSPD------------------------
U5D817          -------FVED---LISLAREEEAIRVRLPNDQSPDD-----------------------
W1NFL7          -------FVED---LISLASEEEAIRLRLPKDQSPDD-----------------------
W1NFN9          -------FVED---LISLASEEEAIRLLLPKDQSPDD-----------------------
U5DBW3          -------FVDD---IMDNGPIHEPKPGNGVLC----------------------------
W1PE57          -------FVDD---VMKSGHSQGPG-----------------------------------
U5D3G1          -------LLEK---IVA-------------------------------------------
AtUGT74B1       -------FIES---LGK-------------------------------------------
U5DFQ6          -------FVGE---LLHT------------------------------------------
U5DI88          -------FVAA---VLENKDSGRDSLGVGGPNQA--------------------------
U5DCN3          -------FVAT---VCFRQVRHSCCIGH--------------------------------
U5D531          -------FITK---ISIHEGDGNI------------------------------------
U5CUG8          -------FVTK---VPFLLLDREK------------------------------------
W1PLP3          ------------------------------------------------------------
U5DI92          ------------------------------------------------------------
U5D3V6          -------FVAK---VVVFSLE---------------------------------------
U5DDJ3          -------FVAN---MSSSHLNSM-------------------------------------
U5DFQ3          -------FVAK---LSSSHLNSK-------------------------------------
U5DI97          -------LVAT---LSNFHLNNYHK-----------------------------------
U5DCP2          -------FVAM---VSRC------------------------------------------
U5DDK2          -------FVAM---VSRCRLKK--------------------------------------
OsUGT709A3      -------LVGFIGELATPIQHAKSESEV--------------------------------
SgUGT720A1      -------LIQD---IKKLNQIEGFINNPNFSDLLRV------------------------
U5CX27          -------LIKQ---VLPEKR----------------------------------------
U5D008          -------LIKE---VLLKKH----------------------------------------
W1PMY7          -------LVVE---VLLKKHGE--------------------------------------
AtUGT85A1       -------VVSK---FLLGQKSQD-------------------------------------
U5CVP6          -------VIKE---VMLKPCKQLGDAC---------------------------------
U5CNJ5          -------LVTQ---GQLKLS----------------------------------------
U5CNG3          -------LG---------------------------------------------------
U5CXJ9          -------LVND---VLFKKSRA--------------------------------------
AtUGT76B1       -------LIAY---ILSF------------------------------------------
W1PV81          -------LVNK---LKS-------------------------------------------
W1PVY0          -------LILQ---LMSC------------------------------------------
W1PX56          -------LILQ---LMSC------------------------------------------
AtUGT90A1       -------ILKE---LCKSRDSNGASE----------------------------------
OsUGT97A1       -------LVDE---LRSSYVVAGDGTAAASSANGGSGKC---------------------
W1PH20          -------LVED---LAGKRR----------------------------------------
W1PBB6          -------FIDE---FCKRKP----------------------------------------
W1PHN8          -------IV---------------------------------------------------
W1PRF4          -------AMEK---WSHH------------------------------------------
AtUGT71B1       -------FVQD---VVDNVP----------------------------------------
W1P1M0          -------VAEE---WRFGSPIWKDMKKVSFVVGNR-------------------------
AtUGT72B1       -------VALK-WKAHKKELEQNGNH----------------------------------
W1PMA9          -------ATQI---WRSTV-----------------------------------------
W1PP20          -------ATQK---WRSNV-----------------------------------------
AtUGT88A1       -------LLQS---WSPK------------------------------------------
U5DB27          -------VAGK---WVQEK-----------------------------------------
U5DBY1          -------VAGK-W-VQEK------------------------------------------
U5DBY4          -------AIHR---ILKG------------------------------------------
U5D276          -------AIHE---ILKAGVVSV-------------------------------------
U5DB32          -------AINE---MLKGGEA---------------------------------------
W1NHX5          -------AVEA---WLGAVQP---------------------------------------
W1NVU7          ------------------------------------------------------------
W1PAP0          -------LVNA---WLEGPPPPPPPPR---------------------------------
W1PD80          -------MVNA---GKHLPSSLETEA----------------------------------
W1PZW0          -------LVKA---WDQAPN----------------------------------------
AtUGT82A1       -------LKNE---LN--------------------------------------------
W1NUV1          -------FVVD---LKRKLGGPSKGEVISFDRDVYGE-----------------------
AtUGT86A1       -------FVSE---VRNRIETKLCNVNGLEISPSN-------------------------
AtUGT87A1       -------FIKD---ITKIV-----------------------------------------
W1P188          -------FLKS---LRS-------------------------------------------
W1P188        1 ------------------------------------------------------------
```
