# Supplementary material for: Exploring the Phytochemical Landscape of the Early-Diverging Flowering Plant Amborella trichopoda Baill
Source: Molecules. 2019 Oct 23;24(21):3814. doi: 10.3390/molecules24213814 (PMC6864642; doi:10.3390/molecules24213814)
Supplement: Supplementary file 1 [file molecules-24-03814-s001.zip › Figure S2.pdf]

```
AtIAMT        1 -----------MGSKGDNVAVCNMKLERLLSMKGGKGQDSYANNSQAQAMHA--------
AnmBAMT       1 ---------------------MKVMKKLLCMNIAGDGETSYANNSGLQKVMM--------
CbSAMT        1 ------------------MDVRQVLHMKGGAGENSYAMNSFIQRQVISITKP--------
AtJMT         1 -----------------------MEVMRVLHMNKGNGETSYAKNSTAQSNII--------
W1PM29        1 -------------------MAISAFSYIASASYSNRNHKLRCWRSSFSFSPPSRPSSRCH
PitCCoAOMT    1 ------------------MASTSVAAAEVKAQTTQAEEPVKVVRHQEVGHKS--------
ZeCCoAOMT     1 --------------------------------MATPTGETQPAKHQEVGHKS--------
PcCCoAOMT     1 ------------------------------------MASNGESKHSEVGHKS--------
MsCCoAOMT     1 ------------------------------MATNEDQKQTESGRHQEVGHKS--------
NtCCoAOMT     1 --------------------------------------MATNGRHQEVGHKS--------
U5CX90        1 -------------------------------MATKQEEQQQVGRHQEVGHKS--------
PotCCoAOMT    1 ------------------------------MATNGEEQQSQAGRHQEVGHKS--------
VvCCoAOMT     1 -----------------------------------MATNQEAGRHQEVGHKS--------
SlCCoAOMT     1 -----------------------------------MLTKTMGNFFTEVKDTG--------
McCCoAOMT     1 ------------------------------------MDFAVMKQVKNTG-----------
U5CZ55        1 -----------------------------------------MWSQLRVPSYG--------
U5D229        1 --------------------------------MPILPTQSLFMNSFMGYMST--------
U5D520        1 ------------------------------------------------------------
U5CZN4        1 ------------------------------------------------------------
U5D0E8        1 ------------------------------------------------------------
CjCoOMT       1 -----------------------MDTPNTFQNDDEIKAQAQVWKHMFGFAET--------
W1P9N8        1 ----------------------------MEQHSLAMEEQAQIWKYVFGFVDS--------
Hv7OMT        1 MQDTSSTQHKSLPNNIIEMDMVTSMPLEANSNGQILQAEAELFCHSFGYLKS--------
W1P041        1 -----------------------MEGVSNNGSNGASHTTWAMMELTNSIAVS--------
Cr4   OMT     1 --------------------------MDLQTA-EFREAQAKIWSQAFSFANC--------
CrOMT2        1 ---------------------------MELQSSEIRNAQAHFFTQVFSFTSM--------
ObCVOMT       1 --------------------MALQNMDISLSTEQLLQAQAHVWNHMYAFANS--------
ObEOMT        1 --------------------MALQKVDISLSTEQLLQAQVHVWNHMYAFANS--------
Mp8OMT        1 ----------------------MALPNGISSKQELLEAQAHVWNHIYSYINS--------
RhOOMT1       1 -----------MERLNSFRHLNQKWSNGEHSN-ELLHAQAHIWNHIFSFINS--------
RhOOMT2       1 ------------MERLNSFKHLNQKWSNGEHSNELLHAQAHIWNHIFSFINS--------
PsHMM         1 ----------------------MDFSTNGSEESELYHAQIHLYKHVYNFVSS--------
GeHI4   OMT   1 ----------------------MAFSTNGSEEIELYHAQIHLYKHVYNFVSS--------
LjHI4   OMT   1 ---------------------MDFSSSNGSEDTELSQAQIHLYKHVYNFVSS--------
MsI7OMT       1 ----------------------MASSINGRKPSEIFKAQALLYKHIYAFIDS--------
GeD7OMT       1 ----------------------MASSINGRKPSEIFQGQALLYRHIYAFIDS--------
Mp4   OMT     1 -----------------------------MVADEEVRVRAEAWNNAFGYIKP--------
Mp7OMTa       1 -----------------------------MAPEEDSLALAEAWNHGFGFIKT--------
Mp7OMTb       1 -----------------------------MAPKEDSLALAEAWNHGFGFIKT--------
AmmCOMT       1 ------------------------------MASHDQEAFLAAMQIVNSSTVD--------
Cj9OMT        1 MCTSLSELKCPVFSTKRKLLLEFALRTSVDMAAQEGVNYLSGLGLSRLICLP--------
Ms2   OMT     1 --------------MGNSYITKEDNQISATSEQTEDSACLSAMVLTTNLVYP--------
Cj6OMT        1 ----------------------------MEVKKDNLSSQAKLWNFIYGFAES--------
Cj4   OMT     1 ------------------------MAFHGKDDVLDIKAQAHVWKIIYGFADS--------
U5DBV9        1 --------------------MAFKETQHQQQGKEEVEAQAVIWEHMFGMAKT--------
Mp3   OMT     1 ----------------MEASFENGRKRSSSSSSEEESAFSFAMELAAGSVLP--------
RcPOMT        1 -------------MRSTSPQTIDSTPMSHPQQEEEEELGKQAIRLANVVILP--------
ZmCOMT        1 -------------MGSTAGDVA---------AVVDEEACMYAMQLASSSILP--------
TaOMT2        1 -----------------MGSIA---------AGADEDACMYALQLVSSSILP--------
AmmBMT        1 ------------------------MAEMKTSPSQDEEAGVVAMQLATSTVLP--------
ZeCOMT        1 -----------------MGSNQ------------DDQAFLFAMQLASASVLP--------
W1NLC7        1 -----------------MGSVG-------PEPLTEEDCCHYAMQLASSSILP--------
CbIEMT        1 -----------------MGSTGNAEIQIIPTHSSDEEANLFAMQLASAAVLP--------
At3   OMT     1 -----------------MGSTA--ETQLTPVQVTDDEAALFAMQLASASVLP--------
MsCOMT        1 -----------------MGSTG--ETQITPTHISDEEANLFAMQLASASVLP--------
Ca3   5   OMT 1 --------------------------------------MLFAMQLASASVLP--------
CaOMT2        1 --------------------------------------MLFAMQLACASVLP--------
W1PG58        1 ------------------------------------------------------------
PitAEOMT      1 -------MDSNMNGLAKSNGCEISRDGFFESEEEELQGQAEAWKCTFAFAES--------
W1P0L5        1 ---------------------------MGDEVQAQAQAQAEAWRLSLAFADS--------
W1P0K5        1 ------------------------------------------------------------
W1P6Z4        1 ------------------------------------------------------------
U5D1A9        1 ------------------------------------------------------------
U5CUU9        1 ------------------------------------------------------------
U5CZT3        1 ------------------------------------------------------------
W1P8Q1        1 ----------------------------MEMQDEDARGQAEFWQLTLAFADS--------
W1P633        1 ------------------MRLRNVQGLKMEMQDEDALGQAEFWQITLAFVDS--------
W1P8Q7        1 ------------------MRLRNVQGLKMEMQDEDALGQAEFWQITLAFVDS--------
W1NGT5        1 ----------------------------MELQDEDAEGQAEFWQLALAFVDS--------
W1NP75        1 ------------------------------------------------------------
W1P628        1 ------------------------------------------------------------
U5CKV5        1 ------------------------------------------------------------
```

AtIAMT	42	---R
AnmBAMT	32	---S
CbSAMT	35	---I
AtJMT	30	---S
W1PM29	42	GKIRAHKHGKVSSRSNGVTVLSSAFFRGKIRIRGYCMLSTSPNGVAVISSNENYGNKEVI
PitCCoAOMT	35	---L
ZeCCoAOMT	21	---L
PcCCoAOMT	17	---L
MsCCoAOMT	23	---L
NtCCoAOMT	15	---L
U5CX90	22	---L
PotCCoAOMT	23	---L
VvCCoAOMT	18	---L
SlCCoAOMT	18	---L
McCCoAOMT	14	---L
U5CZ55	12	---S
U5D229	21	--
U5D520	1	--
U5CZN4	1	--
U5D0E8	1	--
CjCoOMT	30	---I
W1P9N8	25	---A
Hv7OMT	53	---M
W1P041	30	---M
Cr4 OMT	26	---A
CrOMT2	26	---S
ObCVOMT	33	---M
ObEOMT	33	---M
Mp8OMT	31	---M
RhOOMT1	41	---M
RhOOMT2	41	---M
PsHMM	31	---M
GeHI4 OMT	31	---M
LjHI4 OMT	32	---M
MsI7OMT	31	---M
GeD7OMT	31	---M
Mp4 OMT	24	---T
Mp7OMTa	24	---S
Mp7OMTb	24	---S
AmmCOMT	23	---G
Cj9OMT	53	---M
Ms2 OMT	39	---A
Cj6OMT	25	---L
Cj4 OMT	29	---L
U5DBV9	33	---M
Mp3 OMT	37	---M
RcPOMT	40	---M
ZmCOMT	31	---M
TaOMT2	27	---M
AmmBMT	29	---M
ZeCOMT	24	---M
W1NLC7	29	---M
CbIEMT	36	---M
At3 OMT	34	---M
MsCOMT	34	---M
Ca3 5 OMT	15	---M
CaOMT2	15	---M
W1PG58	1	--
PitAEOMT	46	---L
W1P0L5	26	---L
W1P0K5	1	--
W1P6Z4	1	--
U5D1A9	1	--
U5CUU9	1	--
U5CZT3	1	--
W1P8Q1	25	---L
W1P633	35	---L
W1P8Q7	35	---L
W1NGT5	25	---L
W1NP75	1	--
W1P628	1	--
U5CKV5	1	--
U5CKV5	1	--

```
AtIAMT          43 SMLHLLEETLENVHLNSSASPP-PFTAVDLGCSSG-------ANTVHIIDFIVKHISKRF
AnmBAMT         33 KSLHVLDETLKDIIGDHVGFPK-CFKMMDMGCSSGPNALLVMSGIINTIEDLYTEKNIN-
CbSAMT          36 TEAAITALYSGDTVTT-------RLAIADLGCSSGPNALFAVTELIKTVEELRKKMGRE-
AtJMT           31 LGRRVMDEALKKLMMSNSEIS--SIGIADLGCSSGPNSLLSISNIVDTIHNLCPDLDR--
W1PM29         102 SVTPQLYEYILANVREPEI----LRELREETAGMQGSQMQVSPDQAQLLAMLVQILGAQ-
PitCCoAOMT      36 LQSDALYQYILETSVYPREPEP-MKELPRVTAKHPWNLMTTSADEGQFLGLLLKLINAK-
ZeCCoAOMT       22 LQSDALYQYILETSVYPREPQP-MKELRRITAKHPWNLMTTSADEGQFLNLLLKLINAK-
PcCCoAOMT       18 LQSDALYQYILETSVYPREPEA-MKELREVTAKHPWNLMTTSADEGQFLNMLLKLINAK-
MsCCoAOMT       24 LQSDALYQYILETSVFPREHEA-MKELREVTAKHPWNIMTTSADEGQFLSMLLKLINAK-
NtCCoAOMT       16 LQSDALYQYILETSVYPREPEP-MKELREITAKHPWNLMTTSADEGQFLSMLIKLINAK-
U5CX90          23 LQSDELYQYILETSVYPREPES-MKELREITAKHPWNIMTTSADEGQFLNLLLKLINAK-
PotCCoAOMT      24 LQSDALYQYILETSVYPREPEC-MKELREVTAKHPWNIMTTSADEGQFLNMLLKLVNAK-
VvCCoAOMT       19 LQSDALYQYILETSVYPREPES-MKELRELTAQHPWNIMTTSADEGQFLNMLLKLINAK-
SlCCoAOMT       19 LQSEQLHQYILDTSVFPRESEH-LKELRKATESHPMSFMGTSPLAGQLLSFMLKTVKPK-
McCCoAOMT       15 LQSEELCQYILRTSVYPREAGF-LKELREANESHPDSYMSTSPLAGQLMSFVLKLVNAK-
U5CZ55          13 GLTSV-----------YPREAEG-LKDLRDATEAHPWAIIATSPDGGQFISLLLKLINAK-
U5D229          21 -----------------------------------------APDGGQFFSLLLKLINAR-
U5D520           1 ------------------------------------------------------------
U5CZN4           1 -----MSRGVMGV----------------------------PPDEGQFIQMILKLVNAK-
U5D0E8           1 ------------------------------------------------------------
CjCoOMT         31 MLRSTVSLGIPDIIHNNG-----PVTLSQLVTHLPL-----KSTSIDRFHHFMRYLVHM-
W1P9N8          26 VAKCAIELGLVDILESHGS----PMTLSQLCASLHH-----PPKEPATLNRIMRFLAHK-
Hv7OMT          54 ALQSVVKLRIPDVLHRYGG----AASLPELLSTVPI-----HPNKLPYLPRLMKMLAAA-
W1P041          31 SLRAMAKLDVPKIIWQNGENT--PLTALEIATKLG------SEAGSSNLERMLRLLTAH-
Cr4   OMT       27 ALKCAVKLGIADAIDNH--DKK-ALTLSELTEELSI-----KPSKSPFLQRLMRQLVNA-
CrOMT2          27 SLKCAVQLGIPDAIHSHGK----PMALSDLTNSLPI-----NPSKAPYIYRLMRILVAA-
ObCVOMT         34 SLKCAIQLGIPDILHKHDH----PMTLSQLLKAIPI-----NKEKSQSFQRLMRALVNS-
ObEOMT          34 SLKCAIQLGIPDILHKHGR----PMTLSQLLQSIPI-----NKEKTQCFQRLMRALVNS-
Mp8OMT          32 SLKCAIQLGIPDAIHKHGN----PITLSQLADALNI-----NKAKSHGLFRLMRILVHS-
RhOOMT1         42 SLKSAIQLGIPDIINKHGY----PMTLSELTSALPI-----HPTKSHSVYRLMRILVHS-
RhOOMT2         42 SLKSAIQLGIPDIINKHG-----PMTLSELTSALPI-----HPTKSHSVYRLMRILVHS-
PsHMM           32 ALKSAMELGIADAIHNHGK----PMTLPELSSSLKL-----HPSKVNILYRFLRLLTHN-
GeHI4   OMT     32 ALKSAMELGIADVIHNHGK----PITLPELASALKL-----HPSKVGILYRFLRLLTHN-
LjHI4   OMT     33 ALKSAMELGIADVIHSHGK----PITLPELATALNL-----RPSKIGVLHRFLRLLTHN-
MsI7OMT         32 SLKWAVEMNIPNIIQNHGK----PISLSNLVSILQV-----PSSKIGNVRRLMRYLAHN-
GeD7OMT         32 CLKWIVELDIPNIIHNHGK----PITVSELVSILKV-----PQTKAGNVQRIMRYMAHN-
Mp4   OMT       25 AVATAVELGLPDILENHDG----PMSLLELSAA--------TDCPAEPLHRLMRFLVFH-
Mp7OMTa         25 IVKTAVELEIPDILESRGA----PVSIPELATA--------VDCSADRIYRVMRFLAYH-
Mp7OMTb         25 IVKTAVELGIPDILESRGA----PVSIPELAAA--------VDCSADRLYRVMRFLAYH-
AmmCOMT         24 VLNALIELNVFDIIMKKVGLDG-YLHPDEIALNLP-AKN--LEASD-MLDRMLRLLATH-
Cj9OMT          54 ALRAAIELNVFEIISQA-GPDA-QLSPSDIVAKIP-TKN--PSAAI-SLDRILRMLGAS-
Ms2   OMT       40 VLNAAIDLNLFEIIAKATPPGA-FMSPSEIASKLP-ASTQHSDLPN-RLDRMLRLLASY-
Cj6OMT          26 VLKCAVQLDLANIIHNSGT----SMTLSELSSRLPS-----QPVNEDALYRVMRYLVHM-
Cj4   OMT       30 VLRCAVELGIVDIIDNNNQ----PMALADLASKLPV-----SDVNCDNLYRILRYLVKM-
U5DBV9          34 ALNCAVRLGLADIIHAHGR----PITLFHLASLLPI-----QPVNPDRLHRLMRFLVHT-
Mp3   OMT       38 VIKSAIDLNLLELIKRGGEEGA---SAYELAAQIN-AEN--PKAAAEMIDRILQLLAAH-
RcPOMT          41 VLKSALELNVIDIIWGA-GDGE-SLSPSDIAAQLP-TKN--SNAPA-VLDRMLRLLASH-
ZmCOMT          32 TLKNAIELGLLEVLQKEAGGGKAALAPEEVVARMPAAPSDPAAAAA-MVDRMLRLLASY-
TaOMT2          28 TLKNAIELGLLETLM--AAGGK-FLTPAEVAAKLPSAAN--PEAPD-MVDRMLRLLASY-
AmmBMT          30 ILKSAIELDLLNTIAKA-GPGN-YLSPSDLASKLL-LSN--PDAPV-MLARILRVLATY-
ZeCOMT          25 VLKTAIELDLLETIAKA-GPHG-SVSSSELVAQLPKVNN--PEAPV-MIDRICSLLASY-
W1NLC7          30 TLKAAIELDVLEIIAAA-GPGA-LLSPKDVADKLP-TTN--PEAAT-MLDRMLRLLASY-
CbIEMT          37 ALKAAIELDVLEIMAKSVPPSG-YISPAEIAAQLP-TTN--PEAPV-MLDRVLRLLASY-
At3   OMT       35 ALKSALELDLLEIM--A-KNGS-PMSPTEIASKLP-TKN--PEAPV-MLDRILRLLTSY-
MsCOMT          35 ILKSALELDLLEIIAKA-GPGA-QISPIEIASQLP-TTN--PDAPV-MLDRMLRLLACY-
Ca3   5   OMT   16 VLKSAIELDLLEII--A-SQDT-CMSPTEIASHLP-TTN--PHAPT-MIDRILRLLSSY-
CaOMT2          16 VLKSAIELDLLEII--R-GQDT-CMSPTEIASHLP-TTN--PDAPA-MVDRILRLLSCY-
W1PG58           1 ------------------------------------------------------------
PitAEOMT        47 AVKCVVLLGIPDMIARE-GPRA-TLSLCEIVANLP-TES--PDAAC--LFRIMRFLVAK-
W1P0L5          27 AVKSAIELGIPDLLQES-D--T-PLSLPEIAMSLP-STS--PDTTT--LSRIMRFLVRK-
W1P0K5           1 ------------------------------------------------------------
W1P6Z4           1 ------------------------------------------------------------
U5D1A9           1 ------------------------------------------------------------
U5CUU9           1 ------------------------------------------------------------
U5CZT3           1 ------------------------------------------------------------
W1P8Q1          26 TLKCAVELGIPQLIHAKGQ----PLTLSEIITSLQA-----PSPDSWCLSRVMRFLVKK-
W1P633          36 TFKCAVELGIPQIIHAKGQ----PLTLLEIITSLQA-----PSPDSSCLSRVMRFLVKK-
W1P8Q7          36 TLKCAVELGIPQIIHAKGQ----PLTLSEIITSLQA-----PSPDSSCLSRVMRFLVKK-
W1NGT5          26 ALKCEVELGIARIIQEKGG----LVSLSEIVSSLQ--------APSSDTSRLMRFLVKK-
W1NP75           1 ------------------------------------------------------------
W1P628           1 ----------------------------------------------------MLFLVRK-
U5CKV5           1 ------------------------------------------------------------
U5CKV5           1 ------------------------------------------------------------
```

```
AtIAMT          95  DAAGIDPPEFTAFFSDLP---SNDFNTLFQLLPPLVSNTCMEECLAADGN----------
AnmBAMT         91  -----ELPEFEVFLNDLP---DNDFNNLFKLLS-----------HENGN-----------
CbSAMT          88  -----NSPEYQIFLNDLP---GNDFNAIFRSLP-------IENDV--DGV----------
AtJMT           87  -----PVPELRVSLNDLP---SNDFNYICASLP-------EFYDRVNNNKEGLGFGRGGG
W1PM29         157  -----RCI-------------EIGVYTGYSSLA-------IALVLPESGR----------
PitCCoAOMT      94  -----NTM-------------EIGVYTGYSLLS-------TALALPDDGK----------
ZeCCoAOMT       80  -----NTM-------------EIGVYTGYSLLS-------TALALPEDGK----------
PcCCoAOMT       76  -----NTM-------------EIGVYTGYSLLA-------TALALPDDGK----------
MsCCoAOMT       82  -----NTM-------------EIGVYTGYSLLA-------TALAIPEDGK----------
NtCCoAOMT       74  -----NTM-------------EIGVFTGYSLLA-------TAMALPDDGK----------
U5CX90          81  -----NTM-------------EIGVYTGYSLLA-------TALALPDDGK----------
PotCCoAOMT      82  -----NTM-------------EIGVYTGYSLLA-------TALAIPEDGK----------
VvCCoAOMT       77  -----NTM-------------EIGVYTGYSLLA-------TALALPDDGK----------
SlCCoAOMT       77  -----KTI-------------EVGVFTGYSLLA-------TALSIPDDGK----------
McCCoAOMT       73  -----KTI-------------EVGVFTGYSLLL-------TALSIPDDGK----------
U5CZ55          61  -----KTI-------------EIGVFTGYSLLA-------TALSLPDDGK----------
U5D229          39  -----KTL-------------EIGVFTGYSLLT-------TALSLPHDAH----------
U5D520           1  ------------------------------------------------------------
U5CZN4          27  -----KTI-------------EVGVFTGYSLLE-------TALALPEDGQ----------
U5D0E8           1  ------------------------------------------------------------
CjCoOMT         80  -----QLFTISTD--------QITKEDKYELTP-------ASKLLVHGHQ----KSLAPY
W1P9N8          76  ------------------------------------------------------------
Hv7OMT         104  -----GIFTAEDVPATVG---DGEPTTLYHLNA-------VSRLLVDDASVNGGASMSPC
W1P041          82  -----GVFNETML--------GPSEGRRFSLTG-------LGREIVDRSM-------GAF
Cr4   OMT       78  -----GFFTEAKQLRDDNK--DGRTTTAYALTP-------VSRLLLKNEQ----WNLRGI
CrOMT2          77  -----GYF-------------SEEEKNVYSLTP-------FTRLLLKNDP----LNSISM
ObCVOMT         84  -----NFFIEEN---------SNNQEVCYWLTP-------ASRLLLKGAP----LTVAPL
ObEOMT          84  -----NFFIEENN--------SNNQEVCYWLTP-------ASCLLLKEAP----LTVTPL
Mp8OMT          82  -----GFFDKVKVKVKVEGEDEEEEEDAYSLTP-------ASRLLLRSEP----LSVAPF
RhOOMT1         92  -----GFFAKKKL--------SKTDEEGYTLTD-------ASQLLLKDHP----LSLTPY
RhOOMT2         91  -----GFFAKKKL--------SKTDEEGYTLTD-------ASQLLLKDHP----LSLTPF
PsHMM           82  -----GFFAKTTVKSN-----EGEEETAYVLTP-------SSKLLVSGKS----TCLSSL
GeHI4   OMT     82  -----GFFAKTTVPSQNGKDGEEEEETAYALTP-------PSKLLVKGKP----TCLASI
LjHI4   OMT     83  -----GFFAKTTVSRGE----GAEEETAYALTP-------PSKLLVKSNS----TCLAPI
MsI7OMT         82  -----GFFEI-----------ITKEEESYALTV-------ASELLVRGSD----LCLAPM
GeD7OMT         82  -----GFFERVRIQE------EQEENEAYALTA-------ASELLVKGSE----LCLAPM
Mp4   OMT       72  -----GIFKKTAKPP------LSNEAVYYARTA-------LSRLFTRDEL-------GDF
Mp7OMTa         72  -----GIFKRTKPPPES----TEGGSVYYAQTP-------VSRRLTRENL---------G
Mp7OMTb         72  -----GIFKRTEPPPES----TGGGSVYYAQTL-------VSRRLTRENL---------G
AmmCOMT         78  -----SIIKCKLVKRSGG---THRLTRSYGLTS-------ISQYFVQAKD-G--PCLAPY
Cj9OMT         107  -----SIL-----SVS-----TTKSGRVYGLNE-------ESRCLVASED-K--VSVVPM
Ms2   OMT       96  -----SVLTSTTRTIE-----DGGAERVYGLSM-------VGKYLVPDES-R--GYLASF
Cj6OMT          76  -----KLFTKA----------SIDGELRYGLAP-------PAKYLVKGWD----KCMVGS
Cj4   OMT       80  -----EILRVEK---------SDDGQKKYALEP-------IATLLSRNAK----RSMVPM
U5DBV9          84  -----HLFAKTTG--------LNGDEETYWLTP-------SSQLLLRHAE----KSMSGF
Mp3   OMT       91  -----SVLTCRVETPP-----SRR--RRYSLAA-------VCKFLTRDED-G--ASLAPL
RcPOMT          94  -----SILKCSARTGS-----DGQVERLYSAGP-------ICKFLVKDQN-GGTRSVGPL
ZmCOMT          90  -----DVVRCQMED-R-----DGRYERRYSAAP-------VCKWLTPNED-G--VSMAAL
TaOMT2          81  -----NVVSCRTEDGK-----DGRLSRRYGAAP-------VCKYLTPNED-G--VSMSAL
AmmBMT          83  -----KVLGC-----K-----RGEVEWLYCWTP-------VCKYLSNNED-G--ASIAPI
ZeCOMT          79  -----SVLTCTLKETA-----DGCAERFYGLAP-------VCKFLIKNDA-G--VSLAPL
W1NLC7          83  -----SVLTSSVSLTP-----DGHPERLYGAAP-------VTKFLVKNQD-G--VSMAPL
CbIEMT          91  -----SVVTYTLRELP-----SGKVERLYGLAP-------VCKFLTKNED-G--VSLAPF
At3   OMT       86  -----SVLTCSNRKLS-----GDGVERIYGLGP-------VCKYLTKNED-G--VSIAAL
MsCOMT          88  -----IILTCSVRTQQ-----DGKVQRLYGLAT-------VAKYLVKNED-G--VSISAL
Ca3   5   OMT   67  -----SIVTCSVRSVD-----D---QRVYSPAP-------VCKYLTKNQD-G--VSIAAL
CaOMT2          67  -----SVVTCSVRSVD-----D---QRVYGLAP-------VCKYLTKNQD-G--VSIAAL
W1PG58           1  ------------------------------------------------------------
PitAEOMT        99  -----GIFPASKSA-R-----RRAFETRYGLTP-------ASKWLVK-GR-E--LSMAPM
W1P0L5          77  -----HVF----TS-Q-----EIEDETRYGPTR-------TSGWLTKQVH-R--PSLAPF
W1P0K5           1  ------------------------------------------------------------
W1P6Z4           1  ------------------------------------------------------------
U5D1A9           1  ------------------------------------------------------------
U5CUU9           1  ------------------------------------------------------------
U5CZT3           1  ------------------------------------------------------------
W1P8Q1          76  -----RVFSEE----------VKEGEAYYGLTR-------ASKWLVRPGT----SGLAPM
W1P633          86  -----QVFSEE----------VKEGEAYYRLTR-------ASKWLVRLGA----SGLAPM
W1P8Q7          86  -----QVFSEE----------VKVGEAYYRLTR-------ASKLLVRPGA----SGLAPM
W1NGT5          73  -----RVFSVE----------LKQGEAYYGLTR-------LSNWLVRPGE----SGLAPM
W1NP75           1  ------------------------------------------------------------
W1P628           8  -----RVFSVE----------VRQGEAYYGLTR-------MSKWLVGAKE----SGFAPM
U5CKV5           1  ------------------------------------------------------------
U5CKV5           1  ------------------------------------------------------------
```

```
AtIAMT          142 RSYFVAGVPGSFYRRLFPAR--------TIDFFHSA-FSLHWLSQVP-----ESVTDRRS
AnmBAMT         121 --CFVYGLPGSFYGRLLPKK--------SLHFAYSS-YSIHWLSQVP-----EGLE----
CbSAMT          121 --CFINGVPGSFYGRLFPRN--------TLHFIHSS-YSLMWLSQVP-----IGIE----
AtJMT           132 ESCFVSAVPGSFYGRLFPRR--------SLHFVHSS-SSLHWLSQVPCREAEKEDRTITA
W1PM29          182 --LVACDRDAKCLEV-------------AKRYYKRA-GVSHKVEVK------HALA----
PitCCoAOMT      119 --ILAMDINRENYDI-------------GLPIIEKA-GVAHKIDFR------EGPA---L
ZeCCoAOMT       105 --ILALDINRENYEI-------------GLPIIQKA-GVAHKIDFR------EGPA---L
PcCCoAOMT       101 --ILAMDINRENYEI-------------GLPIIEKA-GVGHKIDFR------EGPA---L
MsCCoAOMT       107 --ILAMDINKENYEL-------------GLPVIKKA-GVDHKIDFR------EGPA---L
NtCCoAOMT        99 --ILAMDINRENYEI-------------GLPVIEKA-GLAHKIEFK------EGPA---L
U5CX90          106 --IIAMDINKENYEL-------------GLPVIEKA-GVAHKIDFR------EGPA---L
PotCCoAOMT      107 --ILAMDINRENYEL-------------GLPVIQKA-GVAHKIDFK------EGPA---L
VvCCoAOMT       102 --ILAMDINKENYEL-------------GLPVIQKA-GVAHKIDFK------EGPA---L
SlCCoAOMT       102 --ITAVDIDREAYNV-------------GLALIKKA-GVESKISFI------VSDA---M
McCCoAOMT        98 --ITAIDFDREAYEI-------------GLPFIRKA-GVEHKINFI------ESDA---M
U5CZ55           86 --VVAIDIDKAVYDEY------------GLPIIKKV-GVAHKISFI------HSPA---M
U5D229           64 --ITAIDVDKDVYQEY------------ALPFIQKA-GVEHKINFI------HSPA---L
U5D520            1 --ITAIDVDKKAYEEI------------GLPIIRRA-GVEHKINFV------QSPA---L
U5CZN4           52 --VTAIDVDNKAYEEV------------GLPIIQKA-GVEHKINFI------HSPG---I
U5D0E8            1 ------------------------------------------------------------
CjCoOMT         116 VMLQTHPEEFSVWSHVINVLDG------KKPYWESN-DTSMYEKTE------GDPE----
W1P9N8           76 ----------------------------GL------------------------------
Hv7OMT          149 VLLGTVPLFLGASLKLHEWLQSEEQATTETPFMLAH-GGTLYGIGG------RDSE----
W1P041          115 ILYRLDDALLDAWRLLHEAVLEPS----SEPFIKAH-GKPAYHYHA------DDPV----
Cr4   OMT       120 VLTMLDPAELKAWSVLNDWFK-NDD---PTAFQTAH-EKNYWDYTA------ENTQ----
CrOMT2          108 VLGVNQIAELKAWNAMSEWFQNED----LTAFETAH-GKNFWDFGA------EDK-----
ObCVOMT         119 VQVVLDPTFTNPWHYMSEWFKHENH---ATQFEAAN-GCTFWEKLA------NKPS----
ObEOMT          120 VQVVLDPTFTNPWHHMSEWFT-HEK-H-ATQFEAAN-GCTFWEKLA------NEPS----
Mp8OMT          126 ALAMSDPVYTETWHHLSEWFR-NDA---VAAFDTKY-GMTFPEYAV------ADDR----
RhOOMT1         128 LTAMLDPVLTNPWNYLSTWFQ-NDD---PTPFDTAH-GMTFWDYGN------HQPS----
RhOOMT2         127 LTAMLDPVLTTPWNYLSTWFQNED----PTPFDTAH-GMTFWDYGN------HQPS----
PsHMM           121 VKGALHPSSLDMWGVSKKWFHEDKE---QTLFECAT-GENYWDFLN------KD------
GeHI4   OMT     126 VRGALHPSSLDMWRSSEKWFKEDK----ELTLFESATGESFWDFLN------KDSE----
LjHI4   OMT     123 VKGALHPSSLDMWRSSKKWFL-EDN-EELTLFESAT-GESFWEFLN------KETE----
MsI7OMT         115 VECVLDPTLSGSYHELKKWIYEEDLTLFGVTL-----GSGFWDFLD------KNPE----
GeD7OMT         120 VECVLDPTLSGSYHQLKKWIY-EED---LTLFGVSL-GSHFWEFLN------ENPE----
Mp4   OMT       107 MLLQTGPLSQHPAGLTASSLRTG-----KPQFIRSVNGEDSWTDPV------NGYH----
Mp7OMTa         107 PFVLLQGTMREPSGCVTAE---------TLRTSKRP-GVVNENESDHLY---EDPV----
Mp7OMTb         107 PFVLLQGTMREPSGCVTAE---------TLRMSKRP-GLVDDNDSDRLY---EDPV----
AmmCOMT         120 IRLIHHKEMQRSWSKVKDATI--EG---GVPFNKAHGGKNIFEYLE------KDKD----
Cj9OMT          142 LLFTSDKAVVESFYNIKDVVL-EEG---VIPFDRTH-GMDFFQYAG------KEER----
Ms2   OMT       136 TTFLCYPALLQVWMNFKEAVV-DED---IDLFKNVH-GVTKYEFMG------KDKK----
Cj6OMT          110 ILAITDKDFMAPWHYLKDGLSGES----GTAFEKAL-GTNIWGYMA------EHPE----
Cj4   OMT       115 ILGMTQKDFMTPWHSMKDGLSDN-----GTAFEKAM-GMTIWEYLE------GHPD----
U5DBV9          120 --ARVHEMTVSPWLSLGPCLEEGV----ASAFEKTY-GMSRWEYSA------SHPD----
Mp3   OMT       129 SLLVQDRVFMEPWYHLKDVIV-EG----GVAFERAY-GVHAFEYHA------KDPK----
RcPOMT          136 FLLHHDKVFMEGWFHLNDAIL-EG----GIPFNRAY-GMTAFEYPE------TDER----
ZmCOMT          129 ALMNQDKVLMESWYYLKDAVL-DG----GIPFNKAY-GMTAFEYHG------TDAR----
TaOMT2          121 ALMNQDKVLMESWYYLKDAVL-DG----GIPFNKAY-GMSAFEYHG------TDPR----
AmmBMT          118 LLVHQDKVTIKSWYHLTDAVR-DG----GTAFNKAH-DMSIFEYAS------QDPQ----
ZeCOMT          119 LLMNQDKVLMESWYYLKDPVL-DG----GIPFNKAY-GMSAFEYHG------KDQR----
W1NLC7          123 VLMNQDKVLMESWYHLKDAVL-EG----GVPFNRAY-GMTAFEYHG------KDPR----
CbIEMT          131 LLTATDKVLLEPWFYLKDAIL-EG----GIPFNKAY-GMNEFDYHG------TDHR----
At3   OMT       126 CLMNQDKVLMESWYHLKDAIL-DG----GIPFNKAY-GMSAFEYHG------TDPR----
MsCOMT          128 NLMNQDKVLMESWYHLKDAVL-DG----GIPFNKAY-GMTAFEYHG------TDPR----
Ca3   5   OMT   104 CVAAQDKVLMECWYHMKDAVL-DG----GIPFNKAY-GMPIFDYFA------KDLG----
CaOMT2          104 CLMNQDKVLMESWYHLKDAVL-DG----GIPFNKAY-GMSSFEYHG------TDPR----
W1PG58            1 -----------MW---------------SPRVYSDN-GTDVWEYSA--------------
PitAEOMT        137 LLMQNDETTLAPWHHFNECVL-EG----GVAFQKAN-GAEIWSYAS------DHPD----
W1P0L5          112 LLLETGVVSLKPWHYLSRCVL-EG----GEAFEKAH-GVEIWGYGA------KNRE----
W1P0K5            1 ------------------------------------------------------------
W1P6Z4            1 ------------------------------------------------------------
U5D1A9            1 ------------------------------------------------------------
U5CUU9            1 ------------------------------------------------------------
U5CZT3            1 ------------------------------------------------------------
W1P8Q1          110 LTMLNHPIFLAPWQCLSQCVVEG-----GNAFEKAH-GSKIFKYAL------NNPG----
W1P633          120 LTMLNHPIFLAPWHRFSRCVVEG-----GDAFEIAH------------------------
W1P8Q7          120 LTMLNHPIFLAPWHCFSRCVVER-----VDAFEKAH-GSKLWMYTA------NNTG----
W1NGT5          107 SLLLGHPTILAPWHYVSKCMSEG-----GKPFEKAH-HRDFFDHNS------DNLG----
W1NP75            1 ------------------------------------------------------------
W1P628           42 LMMLSHPTSIAPWYCLSRCVKEGE----SDAFKVAY-GSLLWEYAS------ENPG----
U5CKV5            1 ------------------------------------------------------------
U5CKV5            1 ------------------------------------------------------------
```

```
AtIAMT         188 AAYNRGRVFIHGAGEKT-TTAYKRQFQADL--AEFLRARAAEVKRGGAMFLVCLGR--TS
AnmBAMT        161 -DNNRQNIYMATESPPEVYKAYAKQYERDF--STFLKLRGEEIVPGGRMVLTFNGRSVED
CbSAMT         161 --SNKGNIYMANTCPQSVLNAYYKQFQEDH--ALFLRCRAQEVVPGGRMVLTILGRRSED
AtJMT          183 DLENMGKIYISKTSPKSAHKAYALQFQTDF--LVFLRSRSEELVPGGRMVLSFLGRRSLD
W1PM29         216 --INVLESLIETEEPGCFDFAFVDAEKRMY--KEYYELLLQLVRPGGVIVIDNVLW----
PitCCoAOMT     154 PVLDE--LLKNEDMHGSFDFVFVDRDKDNY--LNYHKRLIDLVKVGGLIAYDNTLW----
ZeCCoAOMT      140 PLLDQ--MLQDEKCHGSFDFIFVDADKDNY--LNYHKRLIDLVKFGGVIGYDNTLW----
PcCCoAOMT      136 PVLDH--MLEDGKYHGTFDFVFVDADKDNY--INYHKRLIDLVKIGGLIGYDNTLW----
MsCCoAOMT      142 PVLDE--MIKDEKNHGSYDFIFVDADKDNY--LNYHKRLIDLVKVGGVIGYDNTLW----
NtCCoAOMT      134 PVLDQ--MIEDGKYHGSYDFIFVDADKDNY--LNYHKRLIDLVKIGGLIGYDNTLW----
U5CX90         141 PVLDQ--MLEDEKYHGSFDFIFVDADKDNY--LNYHKRLIELVKVGGVIGYDNTLW----
PotCCoAOMT     142 PVLDQ--MIEDGKCHGSFDFIFVDADKDNY--INYHKRLIELVKVGGLIGYDNTLW----
VvCCoAOMT      137 PVLDQ--MIEDGKYHGSFDFIFVDADKDNY--LNYHKRLIDLVKVGGIIGYDNTLW----
SlCCoAOMT      137 TLLDD--LLADGRYQGSYDFAFVDADKTNY--VNYHERLIELVKVGGIIAYDNTLW----
McCCoAOMT      133 LALDN--LLQGQESEGSYDFGFVDADKPNY--IKYHERLMKLVKVGGIVAYDNTLW----
U5CZ55         122 PILDE--LLQDPKEEGSFDFAFVDADKPNY--ENYHERLLKLVKVGGLIMYDNTLW----
U5D229         100 PFLDN--LLEDPKEEGSFDFAFVDADKANY--INYHERLLKLVRVGGLILYDNTLWGGTV
U5D520          37 PFLDE--LLEDPKNEGSYDFAFVDADKNNY--SNYHERLLKLVKVGGVIVYDNTLW----
U5CZN4          88 PYLDE--LLQDPKVEGSFDFAFVDADKENY--LNYHERLLRLVKVGGLIMYDNTLW----
U5D0E8           1 ------------------------------------------------MYDNTLW----
CjCoOMT        159 --INE--ILNDAMTSHS--TFMLPALVSGLMKENVLDGVASIVDVGGNSGVVAKGI--VD
W1P9N8          78 -------------------------------------ISTRTDSNGATCYTLTRL----
Hv7OMT         198 --FNT--VFNKAMGASS--EFVAALAVREC--RDVFAGIKSLVDVAGGNGTTARTI--AE
W1P041         160 --FNE--RMMEAMSSSS--GAVMDWFLERY---DGFEGVRRVVDLGGSSGFCLKKI--LD
Cr4   OMT      165 --HCQ--IFEDAMANDS--VLVSKLLVTEY--KFLFEGLTSLIDLGGSTGTIAKAL--AK
CrOMT2         152 --YGK--NFDGVMAADS--ILVSKMLIPEF--NYLFEGLDSLVDVGGGTGTIAKAI--AK
ObCVOMT        165 --MGR--FFDEAMSCDS--RLVAHVLTKDY--KHVIDGIRTLVDVGGGNGTMAKAI--VE
ObEOMT         166 --KGR--FFDEAMSCDS--RLIAHVFTKDY--KHVIEGIRTLVDVGGGNGTMAKAI--VE
Mp8OMT         171 --LNV--LFNEAMACDA--GFVNSILTTEC--REIFDGLESMVDVGGGTGATAKGI--AA
RhOOMT1        173 --IAH--LFNDAMASDA--RLVTSVIINDC--KGVFEGLESLVDVGGGTGTLAKAI--AD
RhOOMT2        172 --IAH--LFNDAMASDA--RLVTSVIIDDC--KGVFEGLESLVDVGGGTGTVAKAI--AD
PsHMM          165 --SDSLSMFQDAMAADS--RLFKLAIQEN---KHVFEGLESLVDVAGGTGGVAKLI--HE
GeHI4   OMT    172 --SGTLSMFQEAMAADS---QMFKLALKEC--RHVFEGLESLVDVGGGTGGVTKLI--HE
LjHI4   OMT    170 --SDTLSMFQEAMAADS---HMFKLALKEC--KHVFEGLGSLVDVAGGRGGVTKLI--RE
MsI7OMT        160 --YNT--SFNDAMASDS---KLINLALRDC--DFVFDGLESIVDVGGGTGTTAKII--CE
GeD7OMT        165 --YNK--SFNDAMASDS---QMINLALRDC--NSGFEGVESIVDVGGGIGTTAKII--CD
Mp4   OMT      152 --MK---VFSDAMAAHA--RETTAAIVRYC--PAAFEGIGTVVDVGGRHGVALEKL--VA
Mp7OMTa        150 --FSM-KVFRDAMASHA--RMTTAAVIENY--GEGFQGVGSLVDVGGSYGMALSML--VK
Mp7OMTb        150 --FSM-KVFRDAMASHA--RMTTAAVIENY--GEGFLGVGSLVDVGGSYGMALSML--VK
AmmCOMT        165 --LAE--LLSQAMDKSI--ATSMNILLQMY--K-GLEGVQEVVDVGGAHGATLSCI--VS
Cj9OMT         187 --VNK--SFNQAMGAGS--TIAFDEVFKVY--K-GFDNLKELVDVGGGIGTSLSNI--VA
Ms2   OMT      181 --MNQ--IFNKSMVDVC--ATEMKRMLEIY--T-GFEGISTLVDVGGGSGRNLELI--IS
Cj6OMT         155 --KNQ--LFNEAMANDS--RLIMSALVKEC--GNIFNGITTLVDVGGGTGTAVRNI--AN
Cj4   OMT      159 --QSQ--LFNEGMAGET--RLLTSSLISGS--RDMFQGIDSLVDVGGGNGTTVKAI--SD
U5DBV9         163 --ENR--AFNEAMACLA--RLVMPAIVGGC--ERLLQGARSVVDVGGGTGTAVGAL--AK
Mp3   OMT      173 --FNK--IFNQAMHNQS--IIFMKRILEIY--K-GFEGVKSLVDVGGGTGASSKMI--VS
RcPOMT         180 --FNR--VFNQAMSNHT--TLILKKILDVY--R-GFEGINVLVDVGGGIGVTLNLI--TN
ZmCOMT         173 --FNR--VFNEGMKNHS--VIITKKLLDFY--T-GFEGVSTLVDVGGGVGATLHAI--TS
TaOMT2         165 --FNR--VFNEGMKNHS--IIITKKLLESY--K-GFEGLGTLVDVGGGVGATVAAI--TA
AmmBMT         162 --FNK--AFNRSMRGHS--TITMKKILETY--K-GFEGLKSIVDVGGGTGATLNMI--IS
ZeCOMT         163 --FNK--VFNSGMFNHS--TMTMKKIVELY--N-GFSGLKTLVDVGGGTGASLNMI--TS
W1NLC7         167 --FNK--VFNRGMSDHS--TITMKKILDDY--P-GFEGLNCIVDVGGGIGATLNMI--VG
CbIEMT         175 --FNK--VFNKGMSSNS--TITMKKILEMY--N-GFEGLTTIVDVGGGTGAVASMI--VA
At3   OMT      170 --FNK--VFNNGMSNHS--TITMKKILETY--K-GFEGLTSLVDVGGGIGATLKMI--VS
MsCOMT         172 --FNK--VFNKGMSDHS--TITMKKILETY--T-GFEGLKSLVDVGGGTGAVINTI--VS
Ca3    5    OMT 148 --SNK--VFNKGMSDFS--SMIIKKILETY--K-GFQGLTSLVDVGGGTGATLTKI--LS
CaOMT2         148 --FNK--VFNRGMSDHS--TITMKKVFQTY--Q-GFQGLTSLVDVGGGTGATLTMI--LS
W1PG58          20 ------------------------------EHEQLDVNSVVDVGGGTGTALRAT--IE
PitAEOMT       181 --FNN--LFNNAMACNA--RIVMKAILSKY--Q-GFHSLNSLVDVGGGTGTAVAEI--VR
W1P0L5         156 --YSR--LFNEAMACNA--QIVLEALLRGY--ENGFEGLGSLVDVGGGTGMTIGEI--VR
W1P0K5           1 ------------------------------------------------------------
W1P6Z4           1 ------------------------------------------------------------
U5D1A9           1 ------------MASNS--RVVLDALLLEY--KSEFAGFTSLVDVAGGTGTAISKI--VE
U5CUU9           1 ------------MASNS--RVVLDALLLEY--KSEFAGFSSLVDVAGGTGTAISKI--VE
U5CZT3           1 ------------MASNS--RVVLDALLLEY--KSEFAGFTSLVDVAGGTGTAISKI--VE
W1P8Q1         154 --SNE--IFNEAMASDA--RVVLDALLSQY--KSGFAGLTSLVDVGGGTGTAVIAI--VK
W1P633         151 ---------------------------------GFAGLTSLVDVAGGTGTVVSAI--VK
W1P8Q7         164 --AND--IFNEAMASDT--VVVLEALLSEY--KSGFAGLTSLVDVAGGTGTAVSAI--VK
W1NGT5         151 --FVH--LFNDAMASGA--RVVLEALLSEH--KSVFSGLTSLVDVGGGTGTALNEI--VK
W1NP75           1 ------------------------------------------------------------
W1P628          87 --YNG--LFNEAMTSNA--RVIFRALLSEY--ESGFSGVTSLVDVGGGTGTAVSEI--VK
U5CKV5           1 ----------------------ALLSEY--ESGFSGLTTLVDVGGGTGTTLSEI--VK
U5CKV5           1 ------------------------------------------------------------
```

```
AtIAMT       243 VD-------------PTDQGGAGLLFGTHFQDAWDDLVREGLVAAEKRDGFNIPVYAPSL
AnmBAMT      218 PS-------------SKDDLAIF----TLLAKTLVDMVAEGLVKMDDLYSFNIPIYSPCT
CbSAMT       217 RA-------------STECCLIW----QLLAMALNQMVSEGLIEEEKMDKFNIPQYTPSP
AtJMT        241 PT-------------TEESCYQW----ELLAQALMSMAKEGIIEEEKIDAFNAPYYAASS
W1PM29       268 -------------------------------------------------HGRVADPLVND
PitCCoAOMT   206 -------------------------------------------------NGSV---VAPP
ZeCCoAOMT    192 -------------------------------------------------NGSL---VAPA
PcCCoAOMT    188 -------------------------------------------------NGSV---AQPA
MsCCoAOMT    194 -------------------------------------------------NGSV---VAPP
NtCCoAOMT    186 -------------------------------------------------NGSV---VAPP
U5CX90       193 -------------------------------------------------NGSV---VAPP
PotCCoAOMT   194 -------------------------------------------------NGSV---VAPP
VvCCoAOMT    189 -------------------------------------------------NGSV---VAPP
SlCCoAOMT    189 -------------------------------------------------GGTV--ALPES
McCCoAOMT    185 -------------------------------------------------GGTV--AQPES
U5CZ55       174 -------------------------------------------------GGS----VAQD
U5D229       156 AEDEASVTEYMRPNRQHLMKLNQFLAVDPRIEISQVPGDSDVE------QGAMRFRAPLP
U5D520        89 -------------------------------------------------GGS----VAKD
U5CZN4       140 -------------------------------------------------GGSL----AMD
U5D0E8         8 -------------------------------------------------GGSL----AMD
CjCoOMT      211 AF-------------PHVKCSVM----DLNHVIERVIKNPKLDYV----AGDMFTSIPNA
W1P9N8        96 -------------------------------------------------SGCLSRNKEHS
Hv7OMT       248 AF-------------PYVKCSVL----DLPQVIQGISSHGTVEFV----AGDMMEFVPPA
W1P041       209 KH-------------KGLEGINF----DLPKVVEAAPPYLGIEHV----GGDMLEWVPQG
Cr4   OMT    215 SF-------------PNLKCTVF----DLPHVVANLESTKNLEFV----GGDMFEKLPPS
CrOMT2       202 SF-------------PDLKCTVF----DLPHVVANLESTENLEFV----GGDMFEKIPSA
ObCVOMT      215 AV-------------PTMKCTVL----DLPHVVAGLESTDKLSYI----GGDMFQSIPSA
ObEOMT       216 AM-------------PTIKCTVI----DLPHVVAGLESTDNLNYI----GGDMFQSIPSA
Mp8OMT       221 AF-------------PGMECTVL----DLPNVVGGLKGSENLSFV----SGDMFDFIPHA
RhOOMT1      223 AF-------------PHIECTVL----DLPHVVADLQGSKNLKYT----GGDMFEAVPPA
RhOOMT2      222 AF-------------PHIECTVL----DLPHVVADLQGSKNLKYT----GGDMFEAVPPA
PsHMM        216 AF-------------PHIKCTVF----DQPQVVGNLTGNENLNFV----GGDMFKSVPSA
GeHI4   OMT  223 EF-------------PHLKCTVF----DQPQVVGNLSGNENLKFV----GGDMFKSIPPA
LjHI4   OMT  221 AF-------------PHVKCTVF----DQPQVVANLTGDENLNFV----GGDMFKSVPPA
MsI7OMT      209 TF-------------PKLKCIVF----DRPQVVENLSGSNNLTYV----GGDMFTSIPNA
GeD7OMT      214 TF-------------PNLKCIVF----DRPKVVENLSGTNNLSYV----GGDMFQSVPKA
Mp4   OMT    201 AF-------------PWVRGISF----DLPEIVAKAPPRPGIEFV----GGSFFESVPKG
Mp7OMTa      201 AF-------------PWLRGICF----DLPEVVARASPLKGVEFV----GGTMFESIPKA
Mp7OMTb      201 AF-------------PWLRGICF----DLPEVVARASPLKGVEFV----AGSMFESIPKA
AmmCOMT      214 MN-------------PHLKGINF----DLPHVVKNAPSLPGIVHV----GGDMFESVPRG
Cj9OMT       236 KH-------------PHIRGINF----ELPHVIGDAPDYPGVEHV----PGDMFEGVPNA
Ms2   OMT    230 KY-------------PLIKGINF----DLPQVIENAPPLSGIEHV----GGDMFASVPQG
Cj6OMT       205 AF-------------PHIKCTVY----DLPHVIADSPGYSEVHCV----AGDMFKFIPKA
Cj4   OMT    209 AF-------------PHIKCTLF----DLPHVIANSYDLPNIERI----GGDMFKSVPSA
U5DBV9       213 AF-------------PSVKFTLY----DLPHVVATVGELNGVDTV----GGDMFSAIPKA
Mp3   OMT    222 KY-------------PLIKAINF----DLPHVIQDASPHPEVEHV----GGDMFVSVPKA
RcPOMT       229 KY-------------PHIKGINF----DLPHVLADAPSYPGVEHV----GGDMFKSVPQG
ZmCOMT       222 RH-------------PHISGVNF----DLPHVISEAPPFPGVRHV----GGDMFASVPAG
TaOMT2       214 HY-------------PTIKGINF----DLPHVISEAPPFPGVTHV----GGDMFQKVPSG
AmmBMT       211 KY-------------PTIKGINF----DLPHVVGDAPSLPGVEHV----GGNMFASVPKG
ZeCOMT       212 KH-------------KSLKGINF----DLPHVIADATTYQGIEHV----GGDMFESVPKG
W1NLC7       216 KY-------------GHIKGINF----DLPHVIADAPEFPGVTHV----GGDMFESVPTG
CbIEMT       224 KY-------------PSINAINF----DLPHVIQDAPAFSGVEHL----GGDMFDGVPKG
At3   OMT    219 KY-------------PNLKGINF----NLPHVIEDAPSHPGIEHV----GGDMFVSVPKG
MsCOMT       221 KY-------------PTIKGINF----DLPHVIEDAPSYPGVEHV----GGDMFVSIPKA
Ca3   5   OMT 197 KY-------------PTIRGINF----DLPHVIQDAPEYPGIEHV----GGDMFVSVPKG
CaOMT2       197 KY-------------PTIRCINF----DLPHVIEDAPEYPGIEHV----GGDMFVSVPKG
W1PG58        46 AY-------------PHIRGINF----DLPHVVDACVEQQGVVHM----GGGMFVAIPKA
PitAEOMT     230 AY-------------PFIRGINY----DLPHVVATASSLSGVQHV----GGDMFETVPTA
W1P0L5       206 AN-------------PHISGINF----DLPHVVATAPHYPGVVHIGVKPGQDNAKSLNQA
W1P0K5         1 ------------------------------------------------------------
W1P6Z4         1 ------------------------------------------------------------
U5D1A9        43 AN-------------PHISGINF----DLPHVVATAPNYPGVVNV----GGDMFSSIPSA
U5CUU9        43 AN-------------PHISGINF----DLPHVVATAPKYPGVVNV----GGDMFSSIPSA
U5CZT3        43 AN-------------PHISGINF----DLPLVVATAPKYPGVVNV----GGDMFSSIPSA
W1P8Q1       204 AH-------------PHISGINF----DLPHVVSSAPEHPGVVNV----SGDMFISIPHA
W1P633       175 AD-------------PHISGINF----DLPQVVSSAPEHPGVVHV----SGDMFISIPHA
W1P8Q7       214 AH-------------PHISRINF----DLPQVVSSAPEHPGVVHV----SGDMFISIPHA
W1NGT5       201 AN-------------PHISGINF----DLPDVVSTAPNYPGVLHV----GGDMFSSIPTA
W1NP75         1 ------------------------------------------------------------
W1P628       137 AH-------------PHISGINF----DLPHVVSTATEYPGVVHV----GGDMFASIPHA
U5CKV5        33 AH-------------PHISGINF----DLPHVVSTAQDYPGVVHV----GGDMFVSIPHA
U5CKV5         1 ------------------------------------------------------------
```

```
AtIAMT       290 QDFK----------------------------------------------EVVDANGSFA
AnmBAMT      261 REVE-------------------------------------------AAILSEGSFTLDR
CbSAMT       260 TEVE-------------------------------------------AEILKEGSFLIDH
AtJMT        284 EELK-------------------------------------------MVIEKEGSFSIDR
W1PM29       279 KKTM--------------------------------------------------------
PitCCoAOMT   214 DAPL--------------------------------------------------------
ZeCCoAOMT    200 DAPL--------------------------------------------------------
PcCCoAOMT    196 DAPM--------------------------------------------------------
MsCCoAOMT    202 DAPL--------------------------------------------------------
NtCCoAOMT    194 DAPL--------------------------------------------------------
U5CX90       201 DAPL--------------------------------------------------------
PotCCoAOMT   202 DAPM--------------------------------------------------------
VvCCoAOMT    197 DAPL--------------------------------------------------------
SlCCoAOMT    198 EVPD--------------------------------------------------------
McCCoAOMT    194 EVPD--------------------------------------------------------
U5CZ55       181 EALV--------------------------------------------------------
U5D229       210 RALS-----------------------LSVVEMIHIKLRGKVRLPLGVALLCEGMVRMLC
U5D520        96 ESLV--------------------------------------------------------
U5CZN4       147 ESSL--------------------------------------------------------
U5D0E8        15 ESSL--------------------------------------------------------
CjCoOMT      250 DAIL-------------------------------------------LKSTLHNYEDDDC
W1P9N8       107 MAAF--------------------------------------------------------
Hv7OMT       287 EAVL-------------------------------------------LKYVLHNWSDQDC
W1P041       248 DVIF-------------------------------------------MKGILCSWDDDEC
Cr4 OMT      254 NAIL-------------------------------------------LKWILHDWNDEDC
CrOMT2       241 NAIL-------------------------------------------LKWILHDWKDEEC
ObCVOMT      254 DAIL-------------------------------------------LKFIIHDWDDEEG
ObEOMT       255 DAIL-------------------------------------------LKSIIHDWDDVEG
Mp8OMT       260 DAIF-------------------------------------------MKFILHDWNDEEC
RhOOMT1      262 DTVL-------------------------------------------LKWILHDWSDEEC
RhOOMT2      261 DTVL-------------------------------------------LKWILHDWNDEEC
PsHMM        255 DAVL-------------------------------------------LKWVLHDWNDELS
GeHI4 OMT    262 DAVL-------------------------------------------LKWVLHDWNDELS
LjHI4 OMT    260 DAVL-------------------------------------------LKWVLHDWNDELS
MsI7OMT      248 DAVL-------------------------------------------LKYILHNWTDKDC
GeD7OMT      253 DAVL-------------------------------------------LKWILHNWTDNDC
Mp4 OMT      240 DLVL-------------------------------------------LMWILHDWSDESC
Mp7OMTa      240 DVVM-------------------------------------------LMFVLHNWSDEEC
Mp7OMTb      240 DVVM-------------------------------------------LMFVLHNWSDEEC
AmmCOMT      253 DVIL-------------------------------------------LQRLLHDWTDEES
Cj9OMT       275 QNIL-------------------------------------------LKWVLHDWDDDRS
Ms2 OMT      269 DAMI-------------------------------------------LKAVCHNWSDEKC
Cj6OMT       244 DAIM-------------------------------------------MKCILHDWDDKEC
Cj4 OMT      248 QAII-------------------------------------------LKLILHDWNDEDS
U5DBV9       252 DVVF-------------------------------------------LMRILHDWGDKEC
Mp3 OMT      261 DAIF-------------------------------------------LKWICHDWSDEHC
RcPOMT       268 DAIF-------------------------------------------MKWILHDWSDEHC
ZmCOMT       261 DAIL-------------------------------------------MKWILHDWSDAHC
TaOMT2       253 DAIL-------------------------------------------MKWILHDWSDEHC
AmmBMT       250 DAIF-------------------------------------------LKWIFHSWGDEEC
ZeCOMT       251 DAIF-------------------------------------------MKWILHDWSDAHC
W1NLC7       255 DAIF-------------------------------------------MKWILHDWGDEYC
CbIEMT       263 DAIF-------------------------------------------IKWICHDWSDEHC
At3 OMT      258 DAIF-------------------------------------------MKWICHDWSDEHC
MsCOMT       260 DAVF-------------------------------------------MKWICHDWSDEHC
Ca3 5 OMT    236 DAIF-------------------------------------------MKWICHDWNEEQC
CaOMT2       236 DAIF-------------------------------------------MKWICHDWSDEHC
W1PG58        85 DVAL-------------------------------------------LMRVLHDWGDEEC
PitAEOMT     269 DAIF-------------------------------------------MKWIMHDWNDEDC
W1P0L5       249 MALLDLTGAQWARTGPIGTRAQLAPGWDEPRPGPLPLLSCMNLRNLPLKWIMHDWGDEDC
W1P0K5         1 -------------------------------------------------SILHDWDDDDC
W1P6Z4         1 -------------------------------------------------WILHDWDDDDC
U5D1A9        82 DGVF-------------------------------------------MK-----------
U5CUU9        82 DGVF-------------------------------------------MKWILHDWKDEDC
U5CZT3        82 DGVF-------------------------------------------MK-----------
W1P8Q1       243 DAVF-------------------------------------------MKWILHDWKDEDC
W1P633       214 DAVF-------------------------------------------MK-----------
W1P8Q7       253 DAVF-------------------------------------------MKWILHDWNDEDC
W1NGT5       240 DAVF--------------------------------------------------------
W1NP75         1 -------------------------------------------------WILHNWEDEEC
W1P628       176 DAVF--------------------------------------------------------
U5CKV5        72 DAVL-------------------------------------------MKWIFHDWEDEEC
U5CKV5         1 ------------------------------------------------------------
```

```
AtIAMT        304 IDKLVV----------------------YKGGSPLVVNEPDDASEVGRAFASSCRSVAGV
AnmBAMT       278 LEVFRV----------------------CWDASD----YTDDDDQQDPSIFGKQRSGKFV
CbSAMT        277 IEASEI----------------------YWSSCTKDGDGGGSVEEEGYNVARCMRAVA--
AtJMT         301 LEISPI----------------------DWEGGSISEESYDLAIRSKPEALASGRRVSNT
W1PM29        283 -------------------------------SIR----DFNRSIFEDKRV----------
PitCCoAOMT    218 RKYVRY----------------------YRDFVM----ELNKALAVDPRI----------
ZeCCoAOMT     204 RKYVRY----------------------YRDFVL----ELNKALAVDPRV----------
PcCCoAOMT     200 RKYVRY----------------------YRDFVI----ELNKALAADPRI----------
MsCCoAOMT     206 RKYVRY----------------------YRDFVL----ELNKALAVDPRI----------
NtCCoAOMT     198 RKYVRY----------------------YRDFVL----ELNKALAADSRI----------
U5CX90        205 RKYVRY----------------------YRDFVI----ELNKALAVDPRI----------
PotCCoAOMT    206 RKYVRY----------------------YRDFVL----ELNKALAADPRI----------
VvCCoAOMT     201 RKYVRY----------------------YRDFVL----ELNKALAADPRI----------
SlCCoAOMT     202 --FMKN----------------------NWVCVT----KLNEILGSDARI----------
McCCoAOMT     198 --FMKE----------------------NREAVI----ELNKLLAADPRI----------
U5CZ55        185 PDFLKP----------------------NRHHLI----NFNRALAIDPRV----------
U5D229        247 TRHLRAVVFLVLPLLVLGLPLLRGRELLSNHFHPLGVLRGMEVLRLRMVF-RPLRTLTMM
U5D520        100 EEYMKY----------------------FRQFVM----KFNQILATDRRI----------
U5CZN4        151 SEHMKK----------------------TRPNLL----KLHETLAIDSRV----------
U5D0E8         19 SEHMKK----------------------TRPNLL----KLQETLAIDSRV----------
CjCoOMT       267 IKILNI----------------------AKEALP---STGGKVILVEIVV-DTENLPLFT
W1P9N8        111 -----------------------------------------------NTI----------
Hv7OMT        304 VKILTR----------------------CREAIS-HGEKAGKVIIIDTVV----GSPSQQ
W1P041        265 VGLLKN----------------------CYKALP----EKGKVIALEQVL-PMHTDTS-P
Cr4   OMT     271 VKILKN----------------------CKKAIQ-EKGNGGKVIIIDTVV-YSQKNEK-E
CrOMT2        258 VKVLKM----------------------CRKAIP-EKEKGGKVILIETVLMDSKKHENEE
ObCVOMT       271 LKILKR----------------------CKDAVG----IGGKVIIIDVVV-GVNHDVD-E
ObEOMT        272 LKILKK----------------------CKDAVV----MGGKVIIIDVVV-GVNHDID-E
Mp8OMT        277 VKILKK----------------------CKEAISRSNNSCRKIILVEIVM-EDEKETH-E
RhOOMT1       279 IKILER----------------------SRVAIT-GKEKKGKVIIIDMMM-ENQKGDE-E
RhOOMT2       278 IKILKR----------------------SRVAIT-SKDKKGKVIIIDMMM-ENQKGDE-E
PsHMM         272 LKILKN----------------------SKEAIS-HKGKDGKVIIIDISI--DENSDDRG
GeHI4   OMT   279 LKILKN----------------------SKEAIS-GKGKEGKVIIIDISI--DEASGDRE
LjHI4   OMT   277 LKILKN----------------------CKEAIS-GRGKEGKVIIIDISI-DETSDDR-E
MsI7OMT       265 LRILKK----------------------CKEAVT-NDGKRGKVTIIDMVI--DKKKDENQ
GeD7OMT       270 RRILEK----------------------CKEAVS-SDGEKGKVIIIEMVI-NENQDEH-E
Mp4   OMT     257 IEIMKK----------------------CKEAIP----TSGKVMIVDAIV--DEDGEGDD
Mp7OMTa       257 VEILKR----------------------CKDAVS---KDKGKVIIIDAVI--DEDGDGDE
Mp7OMTb       257 VEILKR----------------------CKDAVP---KNKGKVIIIDAVI-DEDGNGD-E
AmmCOMT       270 VKILKT----------------------CHEALP----DHGKVVIIEMMP-AELPEND-V
Cj9OMT        292 IKILKN----------------------CWKALP----ENGTVIVIEFVL-PQVLGNN-A
Ms2   OMT     286 IEFLSN----------------------CHKALS----PNGKVIIVEFIL-PEEPNTS-E
Cj6OMT        261 IEILKR----------------------CKEAVP---VKGGKVIIVDIVL---NVQSEHP
Cj4   OMT     265 IKILKQ----------------------CRNAVP---KDGGKVIIVDVAL-DEESDHELS
U5DBV9        269 LEILKQ----------------------IRKAIP----EEGKVIIVDVVL---KEEEEHV
Mp3   OMT     278 RKLLKN----------------------CYDAIL----GNGKVIIAESTL-PEDPNSG-P
RcPOMT        285 LTLLKN----------------------CCKSLP----SSGKVIFVESIL-PEVPDST-V
ZmCOMT        278 ATLLKN----------------------CYDALP----ENGKVIVVECVL-PVNTEAT-P
TaOMT2        270 ATLLKN----------------------CYDALP----AHGKVVLVECIL-PVNPEAT-P
AmmBMT        267 LKILKK----------------------CHQALG----DNKKVIVAEFIL-PEDPGGSDS
ZeCOMT        268 LQVLKN----------------------CYKSLP----ENGKVIVAECIL-PEAPDTT-P
W1NLC7        272 HKLLKN----------------------CYKALP----DSGKVIIAESIL-PIAAENS-L
CbIEMT        280 LKLLKN----------------------CYAALP----DHGKVIVAEYIL-PPSPDPS-I
At3   OMT     275 VKFLKN----------------------CYESLP----EDGKVILAECIL-PETPDSS-L
MsCOMT        277 LKFLKN----------------------CYEALP----DNGKVIVAECIL-PVAPDSS-L
Ca3   5   OMT 253 LKLLKN----------------------CYDALP----NNGKVIVAEYIL-PVVPDSS-L
CaOMT2        253 LKLLKN----------------------CYDALP----NNGKVILAECIL-PEVPDSS-L
W1PG58        102 IAILKN----------------------CREAIP---MERKKVVIVEIVI---REEEKQE
PitAEOMT      286 IKILKN----------------------CRKAIP----DTGKVIIVDVVL-DADQGDN-T
W1P0L5        309 VKILKQ----------------------CKRAIP---EKTGKVIIVDAVV-GGKEGV---
W1P0K5         12 VKILEQ----------------------CKKAIP---RNSGKLIIVDAVL-HPDDGTS-A
W1P6Z4         12 VKILDQ----------------------CKKAIP---KNSGKLIIVDAVL-RPEDDTS-A
U5D1A9            ------------------------------------------------------------
U5CUU9         99 VKILKQ----------------------CRKALP---SKSGKLIIVDVVL----------
U5CZT3            ------------------------------------------------------------
W1P8Q1        260 VKILKQ----------------------CRKALP---SKSGKLIIVDVVL--HLKEDTSA
W1P633            ------------------------------------------------------------
W1P8Q7        270 VKILKQ----------------------CRKALP---SKSGKLIIVDVVL-HLKEDTS-A
W1NGT5        244 MKVFH-------------------------------------------------------
W1NP75         12 VKILKQ----------------------CKKSIP---KNGGKLIIVDAVL-RPEEDTS-S
W1P628        180 MKV---------------------------------------------------------
U5CKV5         89 VKILKQ----------------------CKKSIP---KNGGKLIIVDAVL-RPEEDTS-S
U5CKV5          1 ------------------------------------------------------------
```

```
AtIAMT        342 LVEAHIG---------------------EELSNKLFSRVESRATSHAKDVLVNLQFFHIV
AnmBAMT       312 ADCVRAIT--------------------EPMLASHFGSTIMDLLFGKYAKKIVEHLS--V
CbSAMT        313 ----------------------------EPLLLDHFGEAIIEDVFHRYKLLIIERMS--K
AtJMT         339 IRAVV-----------------------EPMLEPTFGENVMDELFERYAKIVGEYF---Y
W1PM29        298 ----------------------------------SICVVPIGDGMTICCKR---------
PitCCoAOMT    242 ----------------------------------EISQIPVLDGVTLCRRVY--------
ZeCCoAOMT     228 ----------------------------------EICQLPVGDGITLCRRIS--------
PcCCoAOMT     224 ----------------------------------EICMLPVGDGVTLCRRIS--------
MsCCoAOMT     230 ----------------------------------EICMLPVGDGITICRRIK--------
NtCCoAOMT     222 ----------------------------------EICQLPVGDGITLCRRIS--------
U5CX90        229 ----------------------------------EICQLPVGDGITLCRRVS--------
PotCCoAOMT    230 ----------------------------------EICMLPVGDGITLCRRIQ--------
VvCCoAOMT     225 ----------------------------------EICMLPVGDGITLCRRLS--------
SlCCoAOMT     224 ----------------------------------DIAHLPVGDGITFCRRVY--------
McCCoAOMT     220 ----------------------------------EIVHLPLGDGITFCRRLY--------
U5CZ55        209 ----------------------------------EISQIPVGDGVTLCRRLY--------
U5D229        306 MMKALNT--------LGRSNATRTAKIPRNLSNTEEKKLYHGPGATQYVYSPLIGSMRLA
U5D520        124 ----------------------------------EISQIPVADGVTICRRLC--------
U5CZN4        175 ----------------------------------EISQIFIADGVTLCRRLY--------
U5D0E8         43 ----------------------------------EISQIFIAD-----------------
CjCoOMT       301 SARLSMG--------MDMMLMSG-----KERTKKEWEDLLRKANFTSHQVIPIMAIE---
W1P9N8            ------------------------------------------------------------
Hv7OMT        337 ILESQVT--------MDLSMMMLFNG--KVREEQNWHKIFLEAGFSHYKIHNVLGMR---
W1P041        297 STRALLQ--------ADVFLMAIYSPGSKRRTEDIFRRLSEAAGFRTLEIVLPGSF---Y
Cr4   OMT     306 LVDLQIS--------MDMAMVINFAA--KERTEEEWEHLIREAGFSGHKIFPMYDFR---
CrOMT2        295 AVKAQIS--------SDIDMMVFFTA--KERTEEEWATLFREAGFSGYKIFPMIDFR---
ObCVOMT       303 VLEDQLH--------FDMAMMSYFNA--KERTMNEWEKLISAAGFTSYKLTPAFGVR---
ObEOMT        304 VLEDQLH--------FDMAMMC-YFNA-KERTMSEWEKLIYDAGFKSYKLTPAFGVR--S
Mp8OMT        313 ATETKLF--------FDMQMLA-IITG-KERSEKEWGKLFFDAGFTNYKITRVLGLR--S
RhOOMT1       314 SIETQLF--------FDMLMMA-LVGG-KERNEKEWAKLFTDAGFSDYKITPISGLR--S
RhOOMT2       313 SIETQLF--------FDMLMMA-LVRG-QERNEKEWAKLFTDAGFSDYKITPILGLR--S
PsHMM         307 LTELQLE--------YDVVMLTMFLG--KERTKKEWEKLIYDAGFSRYKITPICGFK---
GeHI4   OMT   314 LTELQLD--------YDLVMLTMFNG--KEREKKEWEKLISDAGFSSYKITPICGFK---
LjHI4   OMT   312 LTELKLD--------YDLVMLT-MFNG-KEREKKEWEKLIYDAGFSSYKITPICGFK--S
MsI7OMT       300 VTQIKLL--------MDVNMACLNG---KERNEEEWKKLFIEAGFQHYKISPLTGFL---
GeD7OMT       305 ITGTKLL--------MDVNMAC--LNG-KERSEEEWKKLFIEAGFRDYKISPLTGFL--S
Mp4   OMT     289 FAGARLS--------LDLIMMAVLARG-KERTYREWEYLLREAGFTKFVVKNINTVE---
Mp7OMTa       290 FTGARLG--------LDVTMMATMFEG-RERTYVEWARIINEAGFRRHVVKNIKTLE---
Mp7OMTb       290 FTGARLG--------LDVTMMANMFEG-RERTYVEWAHIINEAGFRRHVVKNIKTLE--S
AmmCOMT       302 QAKNISQ--------VDIRMLIYTHGG-RERTAEEFQMLGKEAGFASSKFICGADLY--G
Cj9OMT        324 ESFNALT--------PDLLMMALNPGG-KERTTIEFDGLAKAAGFAETKFFPISQGL--H
Ms2   OMT     318 ESKLVST--------LDNLMFI-TVGG-RERTEKQYEKLSKLSGFSKFQVACRAFNS--L
Cj6OMT        293 YTKMRLT--------LDLDMML-NTGG-KERTEEEWKKLIHDAGYKGHKITQITAVQ---
Cj4   OMT     299 STRLILD--------IDML---VNTGG-KERTKEVWEKIVKSAGFSGCKIRHIAAIQ--S
U5DBV9        300 FRHARMG--------LDMVMMVQTGG--KERDEGEWAKLLHDAGFKKYEVVPIMAV---Y
Mp3   OMT     310 DTIHAIR--------GDVIMLTVNPGG-KERTEKEFRTLALQAGFKRLVKVCAAFHT--C
RcPOMT        317 TSNIVCE--------QDLLMFTQNPGG-KERTKKEYEALALKSGFSRLEVVCSAYNS--W
ZmCOMT        310 KAQGVFH--------VDMIMLAHNPGG-KERYEREFRELAKGAGFSGFKATYIYANA--W
TaOMT2        302 KAQGVFH--------VDMIMLAHNPGG-RERYEREFEALAKGAGFAAMKTTYIYANA--W
AmmBMT        300 ATKSAVH--------LDAIMLAYVPGG-KERTEKEFESLAKRAGFKSFTKVCCAFNT--W
ZeCOMT        300 ATQNVIH--------IDVIMLAHNPGG-KERTEKEFEALAKGAGFKGFNKAACALNT--W
W1NLC7        304 AAHGVFH--------VDCIMLAHNPGG-KERTEKEFEALAKGAGFAGIKVVCCAYGS--C
CbIEMT        312 ATKVVIH--------TDALMLAYNPGG-KERTEKEFQALAMASGFRGFKVASCAFNT--Y
At3   OMT     307 STKQVVH--------VDCIMLAHNPGG-KERTEKEFEALAKASGFKGIKVVCDAFGV--N
MsCOMT        309 ATKGVVH--------IDVIMLAHNPGG-KERTQKEFEDLAKGAGFQGFKVHCNAFNT--Y
Ca3   5   OMT 285 ASKLSVT--------ADVMIVTQNSGG-KERTEKEFEALAKAAGFQGFQVFCNAFTI--Y
CaOMT2        285 ATKGVVH--------IDVITVAHNPGG-KERTEKEFEALAKAAGFQGFQVFCNAFNT--Y
W1PG58        134 LSDTRLA--------LDMIMLAPTNTG-KEKTAQEWSHVLCEA-----------------
PitAEOMT      318 DKKRKKAVDPIVGTVFDLVMVAHSSGG-KERTEKEWKRILLEGGFSRYNIIEIPALQ--S
W1P0L5        340 FGDATLA--------FDLVMIAHSSGG-KERTAEEWGKLLKDGGFRRFNIISIPTIQ--S
W1P0K5         45 FADSKLA--------FDLLMLALAPGG-KERTEKEWRALLSQGGFSKCNIISLPTLQ--A
W1P6Z4         45 FADSRLA--------FDLLMLALAPGG-KERTEKEWRTLLSQGGFSKCNIISLPTLQ--A
U5D1A9            ------------------------------------------------------------
U5CUU9        124 ------------------------------HLKEDTSAFADS------------------
U5CZT3            ------------------------------------------------------------
W1P8Q1        293 FADSRLA--------FDMLMFAVTSGG-LERTEEEWRALLSKGGFSNCNIKPLPTLQ---
W1P633            ------------------------------------------------------------
W1P8Q7        303 FADSRLA--------FDMLMFAVTSGG-LERTEEEWRALLSKGGFSNCNIKPLPTLQ--S
W1NGT5            ------------------------------------------------------------
W1NP75         45 FADSRLA--------FDLVMVTLSTGG-KERTKKEWRDVLSQGGFGIRKIVSLPALQ--F
W1P628            ------------------------------------------------------------
U5CKV5        122 FADSRLA--------FDLVMVT-LSTG-EKNGQRKSGEMY--------------------
U5CKV5          1 ------------------------------------------------------------
```

```
AtIAMT            381 ASLSFT----------------------EELSNKLFSRVESRATSHAKDVLVNLQFFHIV
AnmBAMT           350 ENSSYFSIVVSLSRR-------------EPMLASHFGSTIMDLLFGKYAKKIVEHLS--V
CbSAMT            343 EKTKFINVIVSLIRKSD-----------EPLLLDHFGEAIIEDVFHRYKLLIIERMS--K
AtJMT             373 VSSPRYAIVILSLVRTG-----------EPMLEPTFGENVMDELFERYAKIVGEYF---Y
W1PM29                ----------------------------------SICVVPIGDGMTICCKR---------
PitCCoAOMT            ----------------------------------EISQIPVLDGVTLCRRVY--------
ZeCCoAOMT             ----------------------------------EICQLPVGDGITLCRRIS--------
PcCCoAOMT             ----------------------------------EICMLPVGDGVTLCRRIS--------
MsCCoAOMT             ----------------------------------EICMLPVGDGITICRRIK--------
NtCCoAOMT             ----------------------------------EICQLPVGDGITLCRRIS--------
U5CX90                ----------------------------------EICQLPVGDGITLCRRVS--------
PotCCoAOMT            ----------------------------------EICMLPVGDGITLCRRIQ--------
VvCCoAOMT             ----------------------------------EICMLPVGDGITLCRRLS--------
SlCCoAOMT             ----------------------------------DIAHLPVGDGITFCRRVY--------
McCCoAOMT             ----------------------------------EIVHLPLGDGITFCRRLY--------
U5CZ55                ----------------------------------EISQIPVGDGVTLCRRLY--------
U5D229            358 AAAAPLEIFHSQIKLD-RSNATRTAKIPRNLSNTEEKKLYHGPGATQYVYSPLIGSMRLA
U5D520                ----------------------------------EISQIPVADGVTICRRLC--------
U5CZN4                ----------------------------------EISQIFIADGVTLCRRLY--------
U5D0E8                ----------------------------------EISQIFIAD-----------------
CjCoOMT           345 SIIVAYS----------MMLMSG-----KERTKKEWEDLLRKANFTSHQVIPIMAIE---
W1P9N8                ------------------------------------------------------------
Hv7OMT            384 SLIEVQP----------LSMMMLFNG--KVREEQNWHKIFLEAGFSHYKIHNVLGMR---
W1P041            346 TALEFQK----------VFLMAIYSPGSKRRTEDIFRRLSEAAGFRTLEIVLPGSF---Y
Cr4   OMT         353 SIIEVYP----------MAMVINFAA--KERTEEEWEHLIREAGFSGHKIFPMYDFR---
CrOMT2            342 SPIEVYP----------IDMMVFFTA--KERTEEEWATLFREAGFSGYKIFPMIDFR---
ObCVOMT           350 SLIEAYP----------MAMMSYFNA--KERTMNEWEKLISAAGFTSYKLTPAFGVR---
ObEOMT            352 -LIEAYP----------MAMMC-YFNA-KERTMSEWEKLIYDAGFKSYKLTPAFGVR--S
Mp8OMT            361 -VIEVFP----------MQMLA-IITG-KERSEKEWGKLFFDAGFTNYKITRVLGLR--S
RhOOMT1           362 -LIEVYP----------MLMMA-LVGG-KERNEKEWAKLFTDAGFSDYKITPISGLR--S
RhOOMT2           361 -LIEVYP----------MLMMA-LVRG-QERNEKEWAKLFTDAGFSDYKITPILGLR--S
PsHMM             354 SLIEVYP----------VVMLTMFLG--KERTKKEWEKLIYDAGFSRYKITPICGFK---
GeHI4   OMT       361 SLIEVFP----------LVMLTMFNG--KEREKKEWEKLISDAGFSSYKITPICGFK---
LjHI4   OMT       360 -LIEVFP----------LVMLT-MFNG-KEREKKEWEKLIYDAGFSSYKITPICGFK--S
MsI7OMT           346 SLIEIYP----------VNMACLNG---KERNEEEWKKLFIEAGFQHYKISPLTGFL---
GeD7OMT           352 -LIEVYP----------VNMAC--LNG-KERSEEEWKKLFIEAGFRDYKISPLTGFL--S
Mp4   OMT         337 FVIEAYP----------LIMMAVLARG-KERTYREWEYLLREAGFTKFVVKNINTVE---
Mp7OMTa           338 SVIEAYP----------VTMMATMFEG-RERTYVEWARIINEAGFRRHVVKNIKTLE---
Mp7OMTb           339 -VIEAYP----------VTMMANMFEG-RERTYVEWAHIINEAGFRRHVVKNIKTLE--S
AmmCOMT           351 -VVELYKKM--------IRMLIYTHGG-RERTAEEFQMLGKEAGFASSKFICGADLY--G
Cj9OMT            373 -VMEFHKINC-------LLMMALNPGG-KERTTIEFDGLAKAAGFAETKFFPISQGL--H
Ms2   OMT         366 GVMEFYK----------NLMFI-TVGG-RERTEKQYEKLSKLSGFSKFQVACRAFNS--L
Cj6OMT            340 SVIEAYPY---------LDMML-NTGG-KERTEEEWKKLIHDAGYKGHKITQITAVQ---
Cj4   OMT         345 -VIEVFP----------ML---VNTGG-KERTKEVWEKIVKSAGFSGCKIRHIAAIQ--S
U5DBV9            347 SIIVAFP----------MVMMVQTGG--KERDEGEWAKLLHDAGFKKYEVVPIMAV---Y
Mp3   OMT         359 -IMECHK----------VIMLTVNPGG-KERTEKEFRTLALQAGFKRLVKVCAAFHT--C
RcPOMT            366 -VMEFHK----------LLMFTQNPGG-KERTKKEYEALALKSGFSRLEVVCSAYNS--W
ZmCOMT            359 -AIEFIK----------MIMLAHNPGG-KERYEREFRELAKGAGFSGFKATYIYANA--W
TaOMT2            351 -AIEFTK----------MIMLAHNPGG-RERYEREFEALAKGAGFAAMKTTYIYANA--W
AmmBMT            349 -IMEFSK----------AIMLAYVPGG-KERTEKEFESLAKRAGFKSFTKVCCAFNT--W
ZeCOMT            349 -VMEFCK----------VIMLAHNPGG-KERTEKEFEALAKGAGFKGFNKAACALNT--W
W1NLC7            353 -IIEFYK----------CIMLAHNPGG-KERTEKEFEALAKGAGFAGIKVVCCAYGS--C
CbIEMT            361 -VMEFLKTA--------ALMLAYNPGG-KERTEKEFQALAMASGFRGFKVASCAFNT--Y
At3   OMT         356 -LIELLKKL--------CIMLAHNPGG-KERTEKEFEALAKASGFKGIKVVCDAFGV--N
MsCOMT            358 -IMEFLKKV--------VIMLAHNPGG-KERTQKEFEDLAKGAGFQGFKVHCNAFNT--Y
Ca3   5   OMT     334 -IIEFSKNISN------VMIVTQNSGG-KERTEKEFEALAKAAGFQGFQVFCNAFTI--Y
CaOMT2            334 -IIEFSKKICN------VITVAHNPGG-KERTEKEFEALAKAAGFQGFQVFCNAFNT--Y
W1PG58                -----------------MIMLAPTNTG-KEKTAQEWSHVLCEA-----------------
PitAEOMT          375 -VIEAFPR---------LVMVAHSSGG-KERTEKEWKRILLEGGFSRYNIIEIPALQ--S
W1P0L5            389 -VIEAFPE---------LVMIAHSSGG-KERTAEEWGKLLKDGGFRRFNIISIPTIQ--S
W1P0K5             94 -IIESFILLCGLH----LLMLALAPGG-KERTEKEWRALLSQGGFSKCNIISLPTLQ--A
W1P6Z4             94 -IIEAFPDE--------LLMLALAPGG-KERTEKEWRTLLSQGGFSKCNIISLPTLQ--A
U5D1A9                ------------------------------------------------------------
U5CUU9                ------------------------------HLKEDTSAFADS------------------
U5CZT3                ------------------------------------------------------------
W1P8Q1            341 SIIEAYPDPKE------MLMFAVTSGG-LERTEEEWRALLSKGGFSNCNIKPLPTLQ---
W1P633                ------------------------------------------------------------
W1P8Q7            352 -IIEAYPDPNE------MLMFAVTSGG-LERTEEEWRALLSKGGFSNCNIKPLPTLQ--S
W1NGT5                ------------------------------------------------------------
W1NP75             94 -IIEAYPQEH-------LVMVTLSTGG-KERTKKEWRDVLSQGGFGIRKIVSLPALQ--F
W1P628                ------------------------------------------------------------
U5CKV5                -----------------LVMVT-LSTG-EKNGQRKSGEMY--------------------
U5CKV5              1 ------------------------------------------------------------
```

```
AtIAMT         381 ASLSFT----------------------EELSNKLFSRVESRATSHAKDVLVNLQFFHIV
AnmBAMT        350 ENSSYFSIVVSLSRR-------------EPMLASHFGSTIMDLLFGKYAKKIVEHLS--V
CbSAMT         343 EKTKFINVIVSLIRKSD-----------EPLLLDHFGEAIIEDVFHRYKLLIIERMS--K
AtJMT          373 VSSPRYAIVILSLVRTG-----------EPMLEPTFGENVMDELFERYAKIVGEYF---Y
W1PM29             ----------------------------------SICVVPIGDGMTICCKR---------
PitCCoAOMT         ----------------------------------EISQIPVLDGVTLCRRVY--------
ZeCCoAOMT          ----------------------------------EICQLPVGDGITLCRRIS--------
PcCCoAOMT          ----------------------------------EICMLPVGDGVTLCRRIS--------
MsCCoAOMT          ----------------------------------EICMLPVGDGITICRRIK--------
NtCCoAOMT          ----------------------------------EICQLPVGDGITLCRRIS--------
U5CX90             ----------------------------------EICQLPVGDGITLCRRVS--------
PotCCoAOMT         ----------------------------------EICMLPVGDGITLCRRIQ--------
VvCCoAOMT          ----------------------------------EICMLPVGDGITLCRRLS--------
SlCCoAOMT          ----------------------------------DIAHLPVGDGITFCRRVY--------
McCCoAOMT          ----------------------------------EIVHLPLGDGITFCRRLY--------
U5CZ55             ----------------------------------EISQIPVGDGVTLCRRLY--------
U5D229         358 AAAAPLEIFHSQIKLD-RSNATRTAKIPRNLSNTEEKKLYHGPGATQYVYSPLIGSMRLA
U5D520             ----------------------------------EISQIPVADGVTICRRLC--------
U5CZN4             ----------------------------------EISQIFIADGVTLCRRLY--------
U5D0E8             ----------------------------------EISQIFIAD-----------------
CjCoOMT        345 SIIVAYS----------MMLMSG-----KERTKKEWEDLLRKANFTSHQVIPIMAIE---
W1P9N8             ------------------------------------------------------------
Hv7OMT         384 SLIEVQP----------LSMMMLFNG--KVREEQNWHKIFLEAGFSHYKIHNVLGMR---
W1P041         346 TALEFQK----------VFLMAIYSPGSKRRTEDIFRRLSEAAGFRTLEIVLPGSF---Y
Cr4   OMT      353 SIIEVYP----------MAMVINFAA--KERTEEEWEHLIREAGFSGHKIFPMYDFR---
CrOMT2         342 SPIEVYP----------IDMMVFFTA--KERTEEEWATLFREAGFSGYKIFPMIDFR---
ObCVOMT        350 SLIEAYP----------MAMMSYFNA--KERTMNEWEKLISAAGFTSYKLTPAFGVR---
ObEOMT         352 -LIEAYP----------MAMMC-YFNA-KERTMSEWEKLIYDAGFKSYKLTPAFGVR--S
Mp8OMT         361 -VIEVFP----------MQMLA-IITG-KERSEKEWGKLFFDAGFTNYKITRVLGLR--S
RhOOMT1        362 -LIEVYP----------MLMMA-LVGG-KERNEKEWAKLFTDAGFSDYKITPISGLR--S
RhOOMT2        361 -LIEVYP----------MLMMA-LVRG-QERNEKEWAKLFTDAGFSDYKITPILGLR--S
PsHMM          354 SLIEVYP----------VVMLTMFLG--KERTKKEWEKLIYDAGFSRYKITPICGFK---
GeHI4   OMT    361 SLIEVFP----------LVMLTMFNG--KEREKKEWEKLISDAGFSSYKITPICGFK---
LjHI4   OMT    360 -LIEVFP----------LVMLT-MFNG-KEREKKEWEKLIYDAGFSSYKITPICGFK--S
MsI7OMT        346 SLIEIYP----------VNMACLNG---KERNEEEWKKLFIEAGFQHYKISPLTGFL---
GeD7OMT        352 -LIEVYP----------VNMAC--LNG-KERSEEEWKKLFIEAGFRDYKISPLTGFL--S
Mp4   OMT      337 FVIEAYP----------LIMMAVLARG-KERTYREWEYLLREAGFTKFVVKNINTVE---
Mp7OMTa        338 SVIEAYP----------VTMMATMFEG-RERTYVEWARIINEAGFRRHVVKNIKTLE---
Mp7OMTb        339 -VIEAYP----------VTMMANMFEG-RERTYVEWAHIINEAGFRRHVVKNIKTLE--S
AmmCOMT        351 -VVELYKKM--------IRMLIYTHGG-RERTAEEFQMLGKEAGFASSKFICGADLY--G
Cj9OMT         373 -VMEFHKINC-------LLMMALNPGG-KERTTIEFDGLAKAAGFAETKFFPISQGL--H
Ms2   OMT      366 GVMEFYK----------NLMFI-TVGG-RERTEKQYEKLSKLSGFSKFQVACRAFNS--L
Cj6OMT         340 SVIEAYPY---------LDMML-NTGG-KERTEEEWKKLIHDAGYKGHKITQITAVQ---
Cj4   OMT      345 -VIEVFP----------ML---VNTGG-KERTKEVWEKIVKSAGFSGCKIRHIAAIQ--S
U5DBV9         347 SIIVAFP----------MVMMVQTGG--KERDEGEWAKLLHDAGFKKYEVVPIMAV---Y
Mp3   OMT      359 -IMECHK----------VIMLTVNPGG-KERTEKEFRTLALQAGFKRLVKVCAAFHT--C
RcPOMT         366 -VMEFHK----------LLMFTQNPGG-KERTKKEYEALALKSGFSRLEVVCSAYNS--W
ZmCOMT         359 -AIEFIK----------MIMLAHNPGG-KERYEREFRELAKGAGFSGFKATYIYANA--W
TaOMT2         351 -AIEFTK----------MIMLAHNPGG-RERYEREFEALAKGAGFAAMKTTYIYANA--W
AmmBMT         349 -IMEFSK----------AIMLAYVPGG-KERTEKEFESLAKRAGFKSFTKVCCAFNT--W
ZeCOMT         349 -VMEFCK----------VIMLAHNPGG-KERTEKEFEALAKGAGFKGFNKAACALNT--W
W1NLC7         353 -IIEFYK----------CIMLAHNPGG-KERTEKEFEALAKGAGFAGIKVVCCAYGS--C
CbIEMT         361 -VMEFLKTA--------ALMLAYNPGG-KERTEKEFQALAMASGFRGFKVASCAFNT--Y
At3   OMT      356 -LIELLKKL--------CIMLAHNPGG-KERTEKEFEALAKASGFKGIKVVCDAFGV--N
MsCOMT         358 -IMEFLKKV--------VIMLAHNPGG-KERTQKEFEDLAKGAGFQGFKVHCNAFNT--Y
Ca3   5   OMT  334 -IIEFSKNISN------VMIVTQNSGG-KERTEKEFEALAKAAGFQGFQVFCNAFTI--Y
CaOMT2         334 -IIEFSKKICN------VITVAHNPGG-KERTEKEFEALAKAAGFQGFQVFCNAFNT--Y
W1PG58             -----------------MIMLAPTNTG-KEKTAQEWSHVLCEA-----------------
PitAEOMT       375 -VIEAFPR---------LVMVAHSSGG-KERTEKEWKRILLEGGFSRYNIIEIPALQ--S
W1P0L5         389 -VIEAFPE---------LVMIAHSSGG-KERTAEEWGKLLKDGGFRRFNIISIPTIQ--S
W1P0K5          94 -IIESFILLCGLH----LLMLALAPGG-KERTEKEWRALLSQGGFSKCNIISLPTLQ--A
W1P6Z4          94 -IIEAFPDE--------LLMLALAPGG-KERTEKEWRTLLSQGGFSKCNIISLPTLQ--A
U5D1A9             ------------------------------------------------------------
U5CUU9             ------------------------------HLKEDTSAFADS------------------
U5CZT3             ------------------------------------------------------------
W1P8Q1         341 SIIEAYPDPKE------MLMFAVTSGG-LERTEEEWRALLSKGGFSNCNIKPLPTLQ---
W1P633             ------------------------------------------------------------
W1P8Q7         352 -IIEAYPDPNE------MLMFAVTSGG-LERTEEEWRALLSKGGFSNCNIKPLPTLQ--S
W1NGT5             ------------------------------------------------------------
W1NP75          94 -IIEAYPQEH-------LVMVTLSTGG-KERTKKEWRDVLSQGGFGIRKIVSLPALQ--F
W1P628             ------------------------------------------------------------
U5CKV5             -----------------LVMVT-LSTG-EKNGQRKSGEMY--------------------
U5CKV5           1 ------------------------------------------------------------
```
